# Supplementary material for: Hydroxylamine-mediated C–C amination via an aza-hock rearrangement
Source: Nat Commun. 2021 Dec 2;12:7029. doi: 10.1038/s41467-021-27271-y (PMC8640056; doi:10.1038/s41467-021-27271-y)
Supplement: Supplementary file 1 — Supplementary Information [file 41467_2021_27271_MOESM1_ESM.docx]

**Supplementary Information**

**Hydroxylamine-Mediated C-C Amination via an Aza-Hock Rearrangement**

Tao Wang, Philipp M. Stein, Hongwei Shi, Chao Hu, Matthias Rudolph, and A. Stephen K. Hashmi

**Table of Contents**

**1. Supplementary Methods**

**2. Supplementary Discussion**

**2.1 Preparation of Aminating Reagents and Substrates**

**2.2 Substrate Scope for Secondary Anilines**

**2.3 Hydroxylamine Substrate for Secondary Anilines**

**2.4 Substrate Scope for Primary Anilines**

**2.5 Further Application**

**2.6 Large-Scale Reaction**

**2.7 Evaluation of MSH Storage Time**

**2.8 Control Experiments**

**2.9 Mechanistic Studies**

**2.10 NMR Spectra**

**3. Supplementary References**

**1.Supplementary Methods**

**Chemicals** were purchased from commercial suppliers (Sigma-Aldrich, Alfa Aesar and TCI) and used as delivered. Dry solvents were dispensed from solvent purification system MB SPS-800. HFIP was used directly without further purification. Deuterated solvents were bought from Euriso-Top. Unless otherwise stated, all reactions and manipulations were carried out under ambient atmosphere in new reaction vials or flasks.

**NMR Spectra** were recorded on a Bruker Avance-III-300, Bruker Avance-III-400, Bruker Avance-III-500, Bruker Avance-III-600. Chemical shifts are reported in ppm with the solvent resonance as the internal standard. For ^1^H NMR: CDCl_3_, 7.26; (CD_3_)_2_SO, 2.50. For ^13^C NMR: CDCl_3_, 77.16; (CD_3_)_2_SO, 39.52. Data is reported as follows: s = singlet, d = doublet, t = triplet, q = quartet, p = pentalet, hept = heptalet, m = multiplet, br = broad singlet, dd =doublet of doublets, td =triplet of doublets; coupling constants in Hz; integration.

**Mass spectra** (MS and HRMS) were determined in the chemistry department of the University Heidelberg under the direction of Dr. J. Gross. EI+ -spectra were measured on a JOEL JMS-700 spectrometer. For ESI+ -spectra a Bruker ApexQu FT-ICR-MS spectrometer was applied.

**Infrared Spectroscopy (IR)** was processed on an FT-IR Bruker (IF528), IR Perkin Elmer (283) or FT-IR Bruker Vector 22. The solvent or matrix is denoted in brackets. For the most significant bands the wave number ν (cm^-1^) is given.

**Melting points** were measured in open glass capillaries in a Büchi melting point apparatus (according to Dr. Tottoli) and were not corrected.

**Flash Column Chromatography** was accomplished using Silica gel 60 (0.04 – 0.063 mm / 230 – 400 mesh ASTM) purchased from Macherey-Nagel.

**Analytical / Preparative thin-layer chromatography** (TLC) was carried out on pre-coated aluminum sheets provided by Macherey-Nagel ALUGRAM® Xtra SIL G/UV254. Components were visualized by treatment with aqueous KMnO_4_ solution or by irradiation under UV light (254 nm).

**2.Supplementary Discussion**

**2.1 Preparation of Aminating Reagents and Substrates**

**General Procedure for Aminating Reagent Synthesis – GP1**

To a solution of *tert*-butyl hydroxycarbamate, sulfonyl chloride or acetyl chloride in THF or Et_2_O or DCM was slowly added Et_3_N at 0^o^C, then the reaction was warmed up to room temperature and stirred overnight or 24 h (monitored by TLC). During the process, white precipitation was developed. After the reaction was totally completed, it’s filtrated to remove white precipitation, diluted with EA or DCM, washed with 1N HCl aq., sat. brine, dried over anhydrous Na_2_SO_4_, filtrated, concentrated in vacuo. The residue was purified by silica gel chromatography with PE/EA to afford the desired product.

***tert*-Butyl methyl(tosyloxy)carbamate (TsONBocMe)** spectral data were in agreement with literature values^1^

To a solution of *N*-methyl hydroxylamine hydrochloride (10.02 g, 120 mmol) in THF/ H_2_O (1:1, 60 mL) was added K_2_CO_3_ (8.29g, 60 mmol) at 0^o^C. Then a solution of di-*tert*-butyl dicarbonate (26.19 g, 120 mmol) in 40 mL THF was added dropwise to the above mixture, and the reaction was stirred for 2 h at 0°C and 3 h at room temperature. The reaction was concentrated in vacuo and the residue was dissolved in 100 mL DCM, washed with water (3 x 40 mL), 40 mL sat. brine and dried over anhydrous Na_2_SO_4_, filtrated and concentrated in vacuo to afford crude *N*-Boc-*N*-methyl hydroxylamine (16.56 g) as a pale orange oil which was directly used for next step without further purification.

To a solution of crude *N*-Boc-*N*-methyl hydroxylamine (16.56 g, 112 mmol) in 250 mL DCM was added Et_3_N (16.4 mL, 118 mmol) and TsCl (21.87 g, 115 mmol) at 0^o^C. The reaction mixture was allowed to warm to room temperature and stirred for 18 h. The reaction was washed with 100 mL 1.0 M HCl, 100 mL sat. brine and dried over anhydrous Na_2_SO_4_, filtrated and concentrated in vacuo. The residue was recrystallized with PE (60^o^C – 80^o^C) to afford TsONBocMe as colorless solid (20.40 g, 56% for two steps) and the residue was concentrated to afford crude TsONBocMe (12.60 g, it still contained some TsCl, which was separated in the following step)

^1^H NMR (300 MHz, CDCl_3_) δ 7.86 (d, *J* = 8.2 Hz, 2H), 7.35 (d, *J* = 8.2 Hz, 2H), 3.24 (s, 3H), 2.45 (s, 3H), 1.22 (s, 9H). ^13^C NMR (75 MHz, CDCl_3_) δ 156.07, 145.67, 131.22, 129.71, 129.52, 83.29, 40.15, 27.57, 21.68.

***N*-Methyl-*O*-tosylhydroxylamine (TsONHMe, 2a)** spectral data were in agreement with literature values^1^

To a solution of TsONBocMe (3.01 g, 10 mmol) in 15 mL DCM was added trifluoroacetic acid (15 ml, 200 mmol) at 0^o^C. The reaction was stirred for 3 h at 0 °C and then it’s poured into 30 mL ice water and extracted with DCM (3 x 30 mL). The combined organic layers were dried over anhydrous Na_2_SO_4_, filtrated and concentrated in vacuo. The residue was purified by silica gel chromatography with PE/EA (5:1) as eluting solvent to afford TsONHMe **2a** as white solid (1.96 g, 98%).

^1^H NMR (300 MHz, CDCl_3_) δ 7.84 (d, *J* = 8.2 Hz, 2H), 7.34 (d, *J* = 8.2 Hz, 2H), 5.85 (s, 1H), 2.74 (s, 3H), 2.45 (s, 3H). ^13^C NMR (75 MHz, CDCl_3_) δ 144.93, 132.26, 129.50, 128.94, 40.10, 21.65.

***tert*-Butyl methyl((methylsulfonyl)oxy)carbamate (MsONBocMe)** spectral data were in agreement with literature values^1^

To a solution of *N*-methyl hydroxylamine hydrochloride (2.51 g, 30 mmol) in THF/ H_2_O (1:1, 20 mL) was added K_2_CO_3_ (2.07 g, 15 mmol) at 0^o^C. Then a solution of di-*tert*-butyl dicarbonate (6.55 g, 30 mmol) in 10 mL THF was added dropwise to the above mixture and stirred for 2 h at 0°C and then 3 h at room temperature. The reaction was concentrated in vacuo and the residue was dissolved in 20 mL DCM, washed with water (3 x 10 mL), 20 mL sat. brine and dried over anhydrous Na_2_SO_4_, filtrated and concentrated in vacuo to afford crude *N*-Boc-*N*-methyl hydroxylamine (3.82 g) as a pale orange oil which was directly used for next step without further purification.

To a solution of *N*-Boc-*N*-methyl hydroxylamine (1.47 g, 10 mmol) in 20 mL DCM was added Et_3_N (1.53 mL, 11 mmol) and MsCl (1.26 g, 11 mmol) at 0^o^C. The reaction mixture was warmed up to room temperature and stirred for 18 h. The reaction was washed with 20 mL 1.0 M HCl, 20 mL sat. brine and dried over anhydrous Na_2_SO_4_, filtrated and concentrated in vacuo. The residue was purified by silica gel chromatography with PE/EA (10:1) as eluting solvent to afford MsONBocMe as a colorless oil (1.58 g, 40% for two steps).

^1^H NMR (300 MHz, CDCl_3_) δ 3.32 (s, 3H), 3.14 (s, 3H), 1.52 (s, 9H). ^13^C NMR (75 MHz, CDCl_3_) δ 156.08, 84.30, 40.57, 36.63, 28.00.

***N*-Methyl-*O*-(methylsulfonyl)hydroxylamine (MsONHMe, 2b)** spectral data were in agreement with literature values^1^

To a solution of MsONBocMe (450 mg, 2 mmol) in 3 mL DCM was added trifluoroacetic acid (3.0 ml, 40 mmol) at 0^o^C. The reaction was stirred for 3 h at 0°C and then it’s poured into 10 mL ice water and extracted with DCM (3 x 10 mL). The combined organic layers were dried over anhydrous Na_2_SO_4_, filtrated and concentrated in vacuo. The residue was purified by silica gel chromatography with PE/EA (5:1) as eluting solvent to afford MsONHMe **2b** as a pale yellow oil (152 mg, 61%).

^1^H NMR (300 MHz, CDCl_3_) δ 6.31 (s, 1H), 3.09 (s, 3H), 2.98 (s, 3H). ^13^C NMR (75 MHz, CDCl_3_) δ 40.43, 34.98.

***tert*-Butyl (tosyloxy)carbamate (TsONHBoc, 5a)** spectral data were in agreement with literature values^1^

Following **GP 1**, *tert*-butyl hydroxycarbamate (13.98 g, 105 mmol), TsCl (19.06 g, 100 mmol), Et_3_N (10.63 g, 105 mmol) in 250 mL THF solution were stirred for 4 h at room temperature. Purification with cyclohexane afforded TsONHBoc **5a** as white solid (24.55 g, 85%).

^1^H NMR (300 MHz, CDCl_3_) δ 7.88 (d, *J* = 8.4 Hz, 2H), 7.67 (s, 1H), 7.36 (d, *J* = 8.4 Hz, 2H), 2.45 (s, 3H), 1.30 (s, 9H). ^13^C NMR (75 MHz, CDCl_3_) δ 154.08, 145.90, 130.58, 129.65, 129.60, 83.84, 27.69, 21.71.

***tert*-Butyl (((4-methoxyphenyl)sulfonyl)oxy)carbamate (5b)**

Following **GP1**, 4-methoxybenzenesulfonyl chloride (620 mg, 3 mmol), *tert*-butyl hydroxycarbamate (399 mg, 3 mmol) and Et_3_N (438 uL, 3.15 mmol) in 10 mL THF solution were stirred for 4 h at 0^o^C. Purification by silica gel chromatography with PE/EA 5:1 as eluting solvent afforded *tert*-butyl (((4-methoxyphenyl)sulfonyl)oxy)carbamate **5b** as white solid (780 mg, 86%).

^1^H NMR (300 MHz, CDCl_3_) δ 7.95 (d, *J* = 8.9 Hz, 2H), 7.68 (s, 1H), 7.04 (d, *J* = 8.9 Hz, 2H), 3.91 (s, 3H), 1.34 (s, 9H). ^13^C NMR (75 MHz, CDCl_3_) δ 164.59, 154.12, 131.94, 124.71, 114.23, 83.80, 55.78, 27.74.

***tert*-Butyl (((4-nitrophenyl)sulfonyl)oxy)carbamate (5c)**

Following **GP1**, 4-nitrobenzenesulfonyl chloride (2.21 g, 10 mmol), *tert*-butyl hydroxycarbamate (1.33 g, 10 mmol) and Et_3_N (1.53 mL, 11 mmol) in 80 mL Et_2_O solution were stirred for 6 h at 0^o^C. Purification with 30 mL hexane afforded *tert*-butyl (((4-nitrophenyl)sulfonyl)oxy)carbamate **5c** as white solid (2.86 g, 90%).

^1^H NMR (300 MHz, CDCl_3_) δ 8.41 (d, *J* = 8.9 Hz, 2H), 8.22 (d, *J* = 8.9 Hz, 2H), 7.78 (s, 1H), 1.32 (s, 9H). ^13^C NMR (75 MHz, CDCl_3_) δ 153.68, 151.26, 139.35, 131.06, 124.01, 84.78, 27.74.

***tert*-Butyl ((mesitylsulfonyl)oxy)carbamate (5d)** spectral data were in agreement with literature values^2^

Following **GP1**, 2,4,6-trimethylbenzenesulfonyl chloride (10.94 g, 50 mmol), *tert*-butyl hydroxycarbamate (6.66 g, 50 mmol) and Et_3_N (7.64 mL, 55 mmol) in 150 mL *tert*-butyl methyl ether solution were stirred overnight at room temperature. Purification with 50 mL hexane afforded *tert*-butyl ((mesitylsulfonyl)oxy)carbamate **5d** as white solid (13.05 g, 83%).

^1^H NMR (300 MHz, CDCl_3_) δ 7.62 (s, 1H), 6.99 (s, 2H), 2.67 (s, 6H), 2.32 (s, 3H), 1.32 (s, 9H). ^13^C NMR (75 MHz, CDCl_3_) δ 154.14, 144.38, 141.92, 131.62, 128.51, 83.79, 27.70, 23.09, 21.08.

***tert*-Butyl (((2,4,6-triisopropylphenyl)sulfonyl)oxy)carbamate (5e)**

Following **GP1**, 2,4,6-triisopropylbenzenesulfonyl chloride (3.03 g, 10 mmol), *tert*-butyl hydroxycarbamate (1.40 g, 10.5 mmol) and Et_3_N (1.46 mL, 10.5 mmol) in 50 mL THF solution were stirred overnight at room temperature. Purification by silica gel chromatography with PE/EA (20:1) as eluting solvent afforded *tert*-butyl (((2,4,6-triisopropylphenyl)sulfonyl)oxy)carbamate **5e** as white solid (3.28 g, 82%).

Mp: 87^o^C

^1^H NMR (300 MHz, CDCl_3_) δ 7.54 (s, 1H), 7.20 (s, 2H), 4.12 (hept, *J* = 6.8 Hz, 2H), 2.92 (hept, *J* = 6.8 Hz, 1H), 1.40 (s, 9H), 1.29 (s, 6H), 1.26 (s, 9H), 1.24 (s, 3H). ^13^C NMR (75 MHz, CDCl_3_) δ 154.68, 154.19, 152.34, 127.70, 123.90, 83.70, 34.30, 30.05, 27.88, 24.73, 23.47.

IR (EXTRACT): ṽ = 3219.13, 3127.94, 2962.11, 2873.81, 1697.68, 1598.27, 1565.16, 1461.19, 1425.33, 1381.81, 1368.59, 1352.88, 1320.96, 1259.76, 1185.53, 1158.30, 1108.07, 1072.81, 1037.40, 1008.33, 937.05, 885.56, 846.67, 801.44, 740.54, 719.22, 660.49, 633.73 cm^-1^

HRMS (DART+) m/z: [M+18]^+^ calcd 417.2418, found 417.2419.

***tert*-Butyl (((2,3,4,5,6-pentamethylphenyl)sulfonyl)oxy)carbamate (5f)**

Following **GP1**, 2,3,4,5,6-pentamethylbenzenesulfonyl chloride (1.23 g, 5 mmol), *tert*-butyl hydroxycarbamate (699 mg, 5.25 mmol) and Et_3_N (0.73 mL, 5.25 mmol) in 20 mL THF solution were stirred overnight at room temperature. Purification by silica gel chromatography with PE/EA (20:1) as eluting solvent afforded *tert*-butyl (((2,3,4,5,6-pentamethylphenyl)sulfonyl)oxy)carbamate **5f** as white solid (1.40 g, 81%).

Mp: 102^o^C

^1^H NMR (500 MHz, CDCl_3_) δ 7.62 (s, 1H), 2.61 (s, 6H), 2.30 (s, 3H), 2.25 (s, 6H), 1.34 (s, 9H). ^13^C NMR (125 MHz, CDCl_3_) δ 154.40, 141.74, 136.75, 134.93, 130.90, 83.70, 27.75, 19.16, 17.99, 17.06.

IR (ATR): ṽ = 3488, 3333, 3264, 2986, 2919, 1738, 1643, 1554, 1452, 1408, 1377, 1335, 1317, 1202, 1163, 1047, 1005, 940, 855, 823, 778, 746, 684, 634cm^-1^

HRMS (ESI+) m/z: [M+Na]^+^ calcd 366.1346, found 366.1345; [2M+Na]^+^ calcd 709.2799, found 709.2804.

***tert*-Butyl ((naphthalen-2-ylsulfonyl)oxy)carbamate (5g)**

Following **GP1**, naphthalene-2-sulfonyl chloride (1.13 g, 5 mmol), *tert*-butyl hydroxycarbamate (699 mg, 5.25 mmol) and Et_3_N (0.73 mL, 5.25 mmol) in 20 mL THF solution were stirred overnight at room temperature. Purification by silica gel chromatography with PE/EA (10:1) as eluting solvent afforded *tert*-butyl ((naphthalen-2-ylsulfonyl)oxy)carbamate **5g** as white solid (1.37 g, 85%).

^1^H NMR (300 MHz, CDCl_3_) δ 8.59 (s, 1H), 8.25 – 7.86 (m, 4H), 7.82 – 7.48 (m, 3H), 1.17 (s, 9H). ^13^C NMR (75 MHz, CDCl_3_) δ 154.08, 135.69, 131.88, 131.79, 130.42, 129.79, 129.49, 129.22, 127.93, 127.81, 123.71, 83.94, 27.56.

***tert*-Butyl ((2,4,6-trimethylbenzoyl)oxy)carbamate (5h)**

Following **GP1**, 2,4,6-trimethylbenzoyl chloride (1.82 g, 10 mmol), *tert*-butyl hydroxycarbamate (1.40 g, 10.5 mmol) and Et_3_N (1.46 mL, 10.5 mmol) in 50 mL DCM solution were stirred for 18 h at room temperature. Purification by silica gel chromatography with PE/EA (10:1) as eluting solvent afforded *tert*-butyl ((2,4,6-trimethylbenzoyl)oxy)carbamate **5h** as white solid (2.47 g, 89%).

^1^H NMR (300 MHz, CDCl_3_) δ 8.05 (s, 1H), 6.88 (s, 2H), 2.37 (s, 6H), 2.29 (s, 3H), 1.53 (s, 9H). ^13^C NMR (75 MHz, CDCl_3_) δ 169.22, 155.53, 140.74, 136.70, 128.63, 126.68, 83.30, 28.08, 21.19, 19.96.

***O*-(2,4,6-Trimethylbenzoyl)hydroxylamine (5i)**

To a solution of *tert*-butyl ((2,4,6-trimethylbenzoyl)oxy)carbamate (**5h**, 1.40 g, 5 mmol) in 7.4 mL DCM was added TFA (7.4 mL, 100 mml) at 0^o^C, and the reaction was stirred at 0 ^o^C for 3 h. The reaction mixture was quenched with 15 mL H_2_O, extracted with DCM (30 mL × 3), and the combined organic layers were concentrated in vacuo. The residue was purified by silica gel chromatography with PE/EA (5:1) as eluting solvent to afford *O*-(2,4,6-trimethylbenzoyl)hydroxylamine **5i** as off-white solid (864 mg, 96%).

^1^H NMR (300 MHz, CDCl_3_) δ 6.79 (s, 2H), 6.31 (br, 2H), 2.22 (s, 9H).^13^C NMR (75 MHz, CDCl_3_) δ 170.76, 140.20, 135.90, 128.48, 21.15, 19.76.

***O*-(4-Nitrobenzoyl)hydroxylamine (5j)** spectral data were in agreement with literature values^3^

To a solution of *tert*-butyl hydroxycarbamate (1.34 g, 10.1 mmol), Et_3_N (1.53 mL, 11 mmol) in 15 mL DCM was dropwise added 4-nitrobenzoyl chloride (1.85 g, 10 mmol) at 0^o^C, then the reaction was stirred at 0°C for 5 min and then warmed up to room temperature. The reaction was quenched with 5 mL H_2_O, washed with aqueous 8 mL 4% K_2_HPO_4_ aq., 10 mL sat. brine., dried over Na_2_SO_4_. Then methanesulfonic acid (1.44 g, 15 mmol) was added to the above DCM phase, and stirred at room temperature for 20 h. The mixture was treated with 15 mL 20% K_2_HPO_4_ aq. at room temperature for 10 min. Then 15 mL THF was added to the above mixture. The organic layer was separated, washed with 30 mL sat. brine and dried over Na_2_SO_4_, filtrated, concentrated in vacuo. 15 mL Heptane was slowly added to the residue for crystallization. The solid was collected by filtration, washed with THF-heptane (1:2, 6 mL), and dried in vacuo to afford *O*-(4-nitrobenzoyl)hydroxylamine **5j** as yellow solid (1.35 g, 74%)

^1^H NMR (300 MHz, CDCl_3_) δ 8.31 (d, *J* = 8.9 Hz, 2H), 8.19 (d, *J* = 8.9 Hz, 2H), 6.74 (br, 2H). ^13^C NMR (125 MHz, CDCl_3_) δ 165.48, 150.71, 133.30, 130.53, 123.72.

***O*-Mesitylenesulfonylhydroxylamine (MSH, 5m)** spectral data were in agreement with literature values^4^

To 70 mL TFA in a flask was added *tert*-butyl ((mesitylsulfonyl)oxy)carbamate (**5d**, 9.46 g, 30 mmol), and then the reaction was stirred for 3 h at 0^o^C. Then it’s poured into 150 mL ice-water and stirred for 30 min, during this time, white precipitate was developed, filtrated to afford the crude product. The crude product was dissolved in 80 mL Et_2_O at 0^o^C, then 150 mL cold PE was added to the above mixture, crystallized for 30 min, filtrated, dried under vacuum to afford *O*-mesitylenesulfonylhydroxylamine **5m** as white solid (4.80 g, 74%).

^1^H NMR (400 MHz, CDCl_3_) δ 7.02 (s, 2H), 4.35 (br, 2H), 2.66 (s, 6H), 2.35 (s, 3H). ^13^C NMR (100 MHz, CDCl_3_) δ 143.76, 140.95, 131.68, 129.11, 22.67, 21.03.

***O*-((2,4,6-Triisopropylphenyl)sulfonyl)hydroxylamine (5n)**

To a solution of *tert*-butyl (((2,4,6-triisopropylphenyl)sulfonyl)oxy)carbamate (**5e**, 2.00 g, 5 mmol) in 8 mL 1,4-dioxane was added 70% HClO_4_ (8.1 mL, 100 mmol) at 0^o^C, and stirred for 3 h at 0^o^C. Then it’s poured into 30 mL ice-water and stirred for 30 min, during this time, white precipitate was developed, filtrated to afford the crude *O*-((2,4,6-triisopropylphenyl)sulfonyl)hydroxylamine **5n** (1.41 g, 95%), which was directly used for next step without further purification.

***O*-((2,3,4,5,6-Pentamethylphenyl)sulfonyl)hydroxylamine (5o)**

To a solution of *tert*-butyl (((2,3,4,5,6-pentamethylphenyl)sulfonyl)oxy)carbamate (**5f**, 654 mg 2 mmol) in 5 mL DCM was added TFA (2.97 mL, 40 mmol) at 0^o^C, and stirred for 3 h at 0^o^C. Then it’s poured into 20 mL ice-water and stirred for 30 min, during this time, white precipitate was developed, filtrated to afford the crude *O*-((2,3,4,5,6-pentamethylphenyl)sulfonyl)hydroxylamine **5o** (378 mg, 78%), which was directly used for next step without further purification.

***tert*-Butyl (pivaloyloxy)carbamate (PivONHBoc, 5r)** spectral data were in agreement with literature values^5^

To a solution of *tert*-butyl hydroxycarbamate (2.66 g, 20 mmol) in 50 mL CHCl_3_ was added Piv_2_O (4.87 mL, 24 mmol) at room temperature, then the reaction was refluxed overnight. The reaction was cooled down to room temperature, washed with 50 mL sat.NaHCO_3_ aq., 50 mL sat.brine, dried over anhydrous Na_2_SO_4_, filtrated and concentrated in vacuo. The residue was purified by silica gel chromatography with PE/EA 20:1 as eluting solvent to afford PivONHBoc **5r** as white solid (1.96 g, 45%)

^1^H NMR (300 MHz, CDCl_3_) δ 7.79 (s, 1H), 1.48 (s, 9H), 1.29 (s, 9H). ^13^C NMR (75 MHz, CDCl_3_) δ 177.78, 155.65, 83.06, 38.15, 28.02, 26.94.

***tert*-Butyl ((methylsulfonyl)oxy)carbamate (MsONHBoc, 5s)** spectral data were in agreement with literature values^1^

Following **GP1**, MsCl (4.06 mL, 52.5 mmol), *tert*-butyl hydroxycarbamate (6.66 g, 50 mmol) and Et_3_N (7.3 mL, 52.5 mmol) in 150 mL Et_2_O solution were stirred at 0^o^C for 12 h. Purification by silica gel chromatography with PE/EA (5:1) as eluting solvent afforded MsONHBoc **5s** as white solid (9.46 g, 90%).

^1^H NMR (300 MHz, CDCl_3_) δ 7.88 (s, 1H), 3.17 (s, 3H), 1.52 (s, 9H). ^13^C NMR (75 MHz, CDCl_3_) δ 154.55, 84.75, 36.34, 27.97.

**MsONH_2_•HOTf (5t)** spectral data were in agreement with literature values^1^

To a solution of MsONHBoc **5s** (1.06 g, 5 mmol) in 15 mL Et_2_O was slowly added HOTf (463 uL, 5.25 mmol) at 0^o^C, then the reaction was stirred for 1 h at room temperature. 15 mL pentane was added to the reaction, and it’s stirred for 15 min, filtrated, washed with Et_2_O, dried under vacuum to afford MsONH_2_•HOTf **5t** as white solid (1.19 g, 91%)

^1^H NMR (300 MHz, DMSO) δ 2.46 (s, 3H), 4.36 (br, 3H). ^13^C NMR (75 MHz, DMSO) δ 120.79 (q, *J* = 322.2 Hz), 39.60. ^19^F NMR (283 MHz, DMSO) δ -77.74.

**TsONH_2_•HOTf (5u)** spectral data were in agreement with literature values^6^

To a solution of TsONHBoc (**5a**, 862 mg, 3 mmol) in 8 mL Et_2_O was slowly added HOTf (278 uL, 3.15 mmol) at 0^o^C, then the reaction was stirred for 1 h at room temperature. 8 mL pentane was added to the reaction, and it’s stirred for 15 min, filtrated, washed with Et_2_O, dried under vacuum to afford TsONH_2_•HOTf **5u** as white solid (952 mg, 94%)

^1^H NMR (400 MHz, DMSO) δ 7.51 (d, *J* = 8.4 Hz, 2H), 7.15 (d, *J* = 8.4 Hz, 2H), 4.37 (br, 3H), 2.29 (s, 3H). ^13^C NMR (100 MHz, DMSO) δ 144.54, 138.65, 128.45, 125.59, 120.77 (q, *J* = 320.1 Hz), 20.87. ^19^F NMR (283 MHz, DMSO) δ -77.74.

**General Procedure for the Mitsunobu Reaction – GP2**

To a solution of PPh_3_ (1.0 eq) in dry THF was added DIAD (1.0 eq) at 0^o^C under nitrogen atmosphere. A white solid was formed after 10 min, and the it’s stirred for 30 min at 0^o^C. Then the solution of alcohol (1.0 eq) in THF and TsONHBoc (1.0 eq) in THF were added to the above mixture successively. Then the reaction was stirred at 0^o^C for another 1 h, and then at room temperature overnight (monitored by TLC). The reaction was concentrated in vacuo and purified by silica gel chromatography with PE/EA to afford the desired product.

**General Procedure for the Removal of the Boc Group – GP3**

To a solution of *N*-Boc-*N*-alkyl-*O*-tosyl hydroxylamine (1.0 eq) in DCM was added TFA (20.0 eq) at 0^o^C, then it was stirred at 0^o^C for 3 h (monitored by TLC). The reaction was quenched with cold water at 0^o^C and then extracted with DCM. The combined organic layers were washed with sat. brine and concentrated in vacuo. The residue was purified by silica gel chromatography with PE/EA to afford the desired product or directly used for next step without further purification.

***tert*-Butyl ethyl(tosyloxy)carbamate** spectral data were in agreement with literature values^1^

Following **GP2**, TsONHBoc (862 mg, 3 mmol), ethanol (138 mg, 3 mmol), PPh_3_ (787 mg, 3 mmol), and DIAD (607 mg, 3 mmol) in 25 mL dry THF solution were stirred at room temperature overnight. Purification by silica gel chromatography with PE/EA (10:1) as eluting solvent afforded *tert*-butyl ethyl(tosyloxy)carbamate as white solid (744 mg, 79%).

^1^H NMR (500 MHz, CDCl_3_) δ 7.86 (d, *J* = 7.6 Hz, 2H), 7.34 (d, *J* = 7.6 Hz, 2H), 3.66 (br, 2H), 2.45 (s, 3H), 1.22 (s, 9H), 1.16 (t, *J* = 7.1 Hz, 3H). ^13^C NMR (125 MHz, CDCl_3_) δ 155.49, 145.59, 131.24, 129.69, 129.47, 83.18, 48.16, 27.58, 21.68, 10.66.

***tert*-Butyl butyl(tosyloxy)carbamate** spectral data were in agreement with literature values^1^

Following **GP2**, TsONHBoc (1.44 g, 5 mmol), n-butanol (370 mg, 5 mmol), PPh_3_ (1,31 g, 5 mmol), and DIAD (1.01 g, 5 mmol) in 35 mL dry THF solution were stirred at room temperature overnight. Purification by silica gel chromatography with PE/EA (20:1) as eluting solvent afforded *tert*-butyl butyl(tosyloxy)carbamate as white solid (1.60 g, 93%).

^1^H NMR (300 MHz, CDCl_3_) δ 7.85 (d, *J* = 8.2 Hz, 2H), 7.34 (d, *J* = 8.2 Hz, 2H), 3.60 (br, 2H), 2.45 (s, 3H), 1.66 – 1.52 (m, 2H), 1.32 – 1.22 (m, 2H), 1.22 (s, 9H), 0.90 (t, *J* = 7.3 Hz, 3H). ^13^C NMR (75 MHz, CDCl_3_) δ 155.52, 145.59, 131.32, 129.68, 129.48, 83.03, 52.67, 27.82, 27.58, 21.67, 19.73, 13.68.

***tert*-Butyl isopropyl(tosyloxy)carbamate** spectral data were in agreement with literature values^7^

Following **GP2**, TsONHBoc (1.14 g, 4 mmol), propan-2-ol (240 mg, 4 mmol), PPh_3_ (1048 mg, 4 mmol), and DIAD (808 mg, 4 mmol) in 35 mL dry THF solution were stirred at room temperature overnight. Purification by silica gel chromatography with PE/EA (10:1) as eluting solvent afforded *tert*-butyl isopropyl(tosyloxy)carbamate as colorless solid (1.04 g, 79%).

^1^H NMR (300 MHz, CDCl_3_) δ 7.86 (d, *J* = 8.2 Hz, 2H), 7.33 (d, *J* = 8.2 Hz, 2H), 4.13 (hept, *J* = 6.7 Hz, 1H), 2.44 (s, 3H), 1.28 (s, 9H), 1.18 (d, *J* = 6.7 Hz, 6H). ^13^C NMR (75 MHz, CDCl_3_) δ 156.36, 145.41, 131.87, 129.59, 129.43, 83.28, 56.57, 27.64, 21.65, 19.10.

***tert*-Butyl (2-(adamantan-1-yl)ethyl)(tosyloxy)carbamate** spectral data were in agreement with literature values^1^

Following **GP2**, TsONHBoc (574 mg, 2 mmol), 2-(adamantan-1-yl)ethan-1-ol (360 mg, 2 mmol), PPh_3_ (524 mg, 2 mmol), and DIAD (404 mg, 2 mmol) in 25 mL dry THF solution was stirred at room temperature overnight. Purification by silica gel chromatography with PE/EA (10:1) as eluting solvent afforded *tert*-butyl (2-(adamantan-1-yl)ethyl)(tosyloxy)carbamate as colorless oil (866 mg, 96%).

^1^H NMR (300 MHz, CDCl_3_) δ 7.85 (d, *J* = 8.4 Hz, 2H), 7.34 (d, *J* = 8.1 Hz, 2H), 3.60 (br, 2H), 2.44 (s, 3H), 1.95 – 1.89 (m, 2H), 1.72 – 1.54 (m, 6H), 1.45 (d, *J* = 2.8 Hz, 6H), 1.23 (s, 9H). ^13^C NMR (75 MHz, CDCl_3_) δ 155.49, 145.54, 131.35, 129.67, 129.44, 83.03, 48.53, 42.07, 39.02, 36.98, 31.41, 28.49, 27.62, 21.65.

***tert*-Butyl (cyclopropylmethyl)(tosyloxy)carbamate**

Following **GP2**, TsONHBoc (862 mg, 3 mmol), cyclopropylmethanol (216 mg, 3 mmol), PPh_3_ (787 mg, 3 mmol), and DIAD (607 mg, 3 mmol) in 25 mL dry THF solution were stirred at room temperature overnight. Purification by silica gel chromatography with PE/EA (10:1) as eluting solvent afforded *tert*-butyl (cyclopropylmethyl)(tosyloxy)carbamate as white solid (762 mg, 74%).

Mp: 63^o^C

^1^H NMR (300 MHz, CDCl_3_) δ 7.86 (d, *J* = 8.3 Hz,2H), 7.34 (d, *J* = 8.3 Hz, 2H), 3.50 (s, 2H), 2.45 (s, 3H), 1.23 (s, 9H), 1.18 – 1.06 (m, 1H), 0.50 (d, *J* = 7.8 Hz, 2H), 0.27 (d, *J* = 4.9 Hz, 2H). ^13^C NMR (75 MHz, CDCl_3_) δ 155.71, 145.55, 131.32, 129.69, 129.46, 83.10, 57.60, 27.57, 21.67, 8.02, 3.35.

IR (EXTRACT): ṽ = 3095.86, 3015.89, 2982.25, 2936.40, 1934.02, 1713.53, 1595.72, 1429.96, 1374.16, 1346.05, 1321.57, 1273.12, 1256.10, 1193.96, 1159.30, 1092.35, 1028.94, 975.88, 930.21, 892.77, 846.69, 815.70, 747.20, 704.21, 666.31, 651.02, 632.52 cm^-1^

HRMS (ESI+) m/z: [M+Na]^+^ calcd 364.1189, found 364.1190; [2M+Na]^+^ calcd 705.2486, found 705.2492.

***tert*-Butyl (2-methoxyethyl)(tosyloxy)carbamate** spectral data were in agreement with literature values^1^

Following **GP2**, TsONHBoc (862 mg, 3 mmol), 2-methoxyethan-1-ol (228 mg, 3 mmol), PPh_3_ (787 mg, 3 mmol), and DIAD (607 mg, 3 mmol) in 25 mL dry THF solution were stirred at room temperature overnight. Purification by silica gel chromatography with PE/EA (10:1) as eluting solvent afforded *tert*-butyl(2-methoxyethyl) (tosyloxy)carbamate as white solid (920 mg, 89%).

^1^H NMR (500 MHz, CDCl_3_) δ 7.86 (d, *J* = 8.3 Hz, 2H), 7.33 (d, *J* = 8.3 Hz, 2H), 3.63 (br, 4H), 3.29 (s, 3H), 2.44 (s, 3H), 1.20 (s, 9H).^13^C NMR (125 MHz, CDCl_3_) δ 155.74, 145.66, 131.00, 129.71, 129.46, 83.02, 67.18, 58.71, 51.73, 27.43, 21.66.

***tert*-Butyl (2-chloroethyl)(tosyloxy)carbamate** spectral data were in agreement with literature values^1^

Following **GP2**, TsONHBoc (862 mg, 3 mmol), 2-chloroethan-1-ol (241 mg, 3 mmol), PPh_3_ (787 mg, 3 mmol), and DIAD (607 mg, 3 mmol) in 25 mL dry THF solution were stirred at room temperature overnight. Purification by silica gel chromatography with PE/EA (10:1) as eluting solvent afforded *tert*-butyl(2-chloroethyl)(tosyloxy) carbamate as white solid (855 mg, 82%).

^1^H NMR (300 MHz, CDCl_3_) δ 7.86 (d, *J* = 8.2 Hz, 2H), 7.36 (d, *J* = 8.2 Hz, 2H), 3.99 (t, *J* = 6.5 Hz, 2H), 3.73 (t, *J* = 6.5 Hz, 2H), 2.46 (s, 3H), 1.21 (s, 9H). ^13^C NMR (75 MHz, CDCl_3_) δ 154.81, 146.01, 130.80, 129.78, 129.62, 83.78, 53.46, 39.01, 27.50, 21.70.

**Methyl (tosyloxy)carbamate (2j)**

To a solution of hydroxylamine hydrochloride (6.95 g, 100 mmol) in 80 mL 1.5 M NaOH aq. was slowly added methyl chloroformate (1.5 mL, 20 mmol) at 0^o^C. Then the reaction was warmed up to room temperature, and stirred for 4 h. The reaction mixture was adjusted to PH 4.5 with 6 M HCl aq., extracted with Et_2_O (100 mL × 3), and the combined organic layers were washed with 100 mL sat. brine, dried over Na_2_SO_4_, concentrated in vacuo to afford the crude methyl hydroxycarbamate as colorless oil (600 mg).

To a solution of crude methyl hydroxycarbamate (600 mg, 6.59 mmol) and TsCl (1256 mg, 6.59 mmol) in 30 mL Et_2_O was slowly added Et_3_N (0.96 mL, 6.59 mmol) at 0°C. The reaction mixture was warmed up to room temperature and stirred overnight. The reaction mixture was washed with 30 mL H_2_O, 30 mL sat. brine, dried over Na_2_SO_4_, concentrated in vacuo. The residue was purified by silica gel chromatography with PE/EA (3:1) as eluting solvent afforded methyl (tosyloxy)carbamate **2j** as white solid (1.32 g, 27% for two steps).

^1^H NMR (300 MHz, CDCl_3_) δ 7.91 (s, 1H), 7.87 (d, *J* = 8.2 Hz, 2H), 7.36 (d, *J* = 8.2 Hz, 2H), 3.64 (s, 3H), 2.46 (s, 3H). ^13^C NMR (75 MHz, CDCl_3_) δ 155.97, 146.19, 130.28, 129.73, 129.53, 53.66, 21.77.

***tert*-Butyl prop-2-yn-1-yl(tosyloxy)carbamate** spectral data were in agreement with literature values^1^

Following **GP2**, TsONHBoc (862 mg, 3 mmol), prop-2-yn-1-ol (168 mg, 3 mmol), PPh_3_ (787 mg, 3 mmol), and DIAD (607 mg, 3 mmol) in 25 mL dry THF solution were stirred at room temperature overnight. Purification by silica gel chromatography with PE/EA (10:1) as eluting solvent afforded *tert*-butyl prop-2-yn-1-yl(tosyloxy)carbamate as colorless solid (826 mg, 85%).

^1^H NMR (400 MHz, CDCl_3_) δ 7.86 (d, *J* = 8.3 Hz, 2H), 7.35 (d, *J* = 8.3 Hz, 2H), 4.30 (br, 2H), 2.45 (s, 3H), 2.25 (t, *J* = 2.4 Hz, 1H), 1.26 (s, 9H).^13^C NMR (100 MHz, CDCl_3_) δ 154.92, 145.85, 131.02, 129.72, 129.56, 84.33, 76.30, 73.16, 42.88, 27.55, 21.68.

***tert*-Butyl pent-4-en-1-yl(tosyloxy)carbamate** spectral data were in agreement with literature values^8^

Following **GP2**, TsONHBoc (574 mg, 2 mmol), pent-4-en-1-ol (172 mg, 2 mmol), PPh_3_ (524 mg, 2 mmol), and DIAD (404 mg, 2 mmol) in 15 mL dry THF solution were stirred at room temperature overnight. Purification by silica gel chromatography with PE/EA (10:1) as eluting solvent afforded *tert*-butyl pent-4-en-1-yl(tosyloxy)carbamate as white solid (576 mg, 81%).

^1^H NMR (300 MHz, CDCl_3_) δ 7.85 (d, *J* = 8.2 Hz, 2H), 7.34 (d, *J* = 8.1 Hz, 2H), 5.76 (ddt, *J* = 16.8, 10.2, 6.5 Hz, 1H), 5.08 – 4.92 (m, 2H), 3.62 (br, 2H), 2.45 (s, 3H), 2.03 (q, *J* = 7.8 Hz, 2H), 1.72 (p, *J* = 7.8 Hz, 2H), 1.22 (s, 9H). ^13^C NMR (75 MHz, CDCl_3_) δ 155.42, 145.64, 137.29, 131.27, 129.69, 129.49, 115.27, 83.15, 52.44, 30.58, 27.59, 24.89, 21.67.

***tert*-Butyl (3-phenylpropyl)(tosyloxy)carbamate** spectral data were in agreement with literature values^9^

Following **GP2**, TsONHBoc (574 mg, 2 mmol), 3-phenylpropan-1-ol (272 mg, 2 mmol), PPh_3_ (524 mg, 2 mmol), and DIAD (404 mg, 2 mmol) in 15 mL dry THF solution were stirred at room temperature overnight. Purification by silica gel chromatography with PE/EA (10:1) as eluting solvent afforded *tert*-butyl (3-phenylpropyl)(tosyloxy)carbamate as white solid (800 mg, 99%).

^1^H NMR (300 MHz, CDCl_3_) δ 7.85 (d, *J* = 8.4 Hz, 2H), 7.33 (d, *J* = 8.4 Hz, 2H), 7.30 – 7.25 (m, 2H), 7.23 – 7.13 (m, 3H), 3.64 (br, 2H), 2.60 (t, *J* = 7.8 Hz, 2H), 2.45 (s, 3H), 1.97 (p, *J* = 7.8Hz, 2H), 1.22 (s, 9H). ^13^C NMR (75 MHz, CDCl_3_) δ 155.46, 145.66, 141.09, 131.26, 129.68, 129.52, 128.42, 128.30, 126.00, 83.23, 52.64, 32.82, 27.61, 27.42, 21.69.

***N*,*N*-Dimethyl-*O*-tosylhydroxylamine** spectral data were in agreement with literature values^1^

To a solution of *N*, *N*-dimethylhydroxylamine hydrochloride (878 mg, 9 mmol) and Et_3_N (2.75 mL, 19.8 mmol) in 100 mL DCM was dropwise added TsCl (1.89 g, 9.9 mmol) in 10 mL DCM solution at 0^o^C. A white precipitate was formed after 20 min, and the reaction was stirred at 0^o^C for 2 h. The reaction mixture was filtrated, and concentrated in vacuo. The residue was purified by silica gel chromatography with PE/EA (10:1) to afford *N*, *N*-dimethyl-*O*-tosylhydroxylamine as white solid (702 mg, 36%).

^1^H NMR (500 MHz, CDCl_3_) δ 7.85 (d, *J* = 8.2 Hz, 2H), 7.33 (d, *J* = 8.2 Hz, 2H), 2.63 (s, 6H), 2.45 (s, 3H). ^13^C NMR (125 MHz, CDCl_3_) δ 144.79, 132.51, 129.41, 128.93, 48.91, 21.65.

**General Procedure for Alcohols Synthesis**

**GP4a**. To a mixture of ketone (1 eq.) in MeOH or THF/H_2_O (5:1) solution was portionwise added NaBH_4_ (1.2 – 2.0 eq.) at 0^o^C, then the reaction mixture was stirred for 3 h at 0^o^C. The reaction was quenched with 1N HCl aq., extracted with EA, and the combined organic layers were washed with sat. brine, dried over anhydrous Na_2_SO_4_, filtrated and concentrated in vacuo. The crude residue was purified by silica gel chromatography with PE/EA to afford the desired alcohol.

**GP4b**. To a solution of AlCl_3_ (2.0 – 3.0 eq.) in DCE or DCM was added acetyl chloride (2.0 – 3.0 eq.) at 0^o^C, and the mixture was stirred for 10 min at 0^o^C. Then the arene (1.0 eq) was added to the above mixture at 0^o^C, stirred for 3 h. The reaction was quenched with sat. NH_4_Cl aq. at 0^o^C, extracted with DCM, and the combined organic layers were washed with sat. brine, dried over anhydrous Na_2_SO_4_, filtrated and concentrated in vacuo to afford the crude ketone.

To a solution of the above crude ketone in MeOH or THF/H_2_O (5:1) was portionwise added NaBH_4_ (2.0 eq) at 0^o^C, and stirred for 3 h at 0^o^C. The reaction was quenched with 1N HCl aq., extracted with EA, and the combined organic layers were washed with sat. brine, dried over anhydrous Na_2_SO_4_, filtrated and concentrated in vacuo. The crude residue was purified by silica gel chromatography with PE/EA to afford the desired alcohol.

**GP4c**. To a solution of ketone in THF was portionwise added LiAlH_4_ (x mg) at 0^o^C, then the reaction was refluxed for some time. The reaction was cooled down to 0^o^C, then H_2_O (x mg) was added to the above mixture, stirred for 10 min, followed by 15% NaOH (x mg), stirred for 10 min, finally H_2_O (3x mg) was added, and stirred for 2 h at 0^o^C. The white precipitate was developed during this process, and it’s filtrated with celite, and concentrated in vacuo. The crude residue was purified by silica gel chromatography with PE/EA to afford the desired alcohol.

**GP4d**. To a solution of ketone (1.0 eq) in THF was slowly added Grignard reagent (1.5 – 2.0 eq.) at 0^o^C, then the reaction was stirred overnight at room temperature. The reaction was quenched with sat. NH_4_Cl aq., extracted with EA, and the combined organic layers were washed with sat. brine, dried over anhydrous Na_2_SO_4_, filtrated and concentrated in vacuo. The crude residue was purified by silica gel chromatography with PE/EA to afford the desired alcohol.

**1-(4-(*tert*-Butyl)phenyl)ethan-1-ol (1d)** spectral data were in agreement with literature values^10^

Following **GP4a**, 1-(4-(*tert*-butyl)phenyl)ethan-1-one (881 mg, 5 mmol), NaBH_4_ (227 mg, 6 mmol) in 10 mL MeOH solution were stirred for 3 h at 0^o^C. Purification by silica gel chromatography with PE/EA (10:1) as eluting solvent afforded 1-(4-(*tert*-butyl)phenyl)ethan-1-ol **1d** as colorless solid (873 mg, 98%).

^1^H NMR (500 MHz, CDCl_3_) δ 7.39 (d, *J* = 8.2 Hz, 2H), 7.32 (d, *J* = 8.2 Hz, 2H), 4.88 (q, *J* = 6.5 Hz, 1H), 1.82 (br, 1H), 1.51 (d, *J* = 6.5 Hz, 3H), 1.33 (s, 9H). ^13^C NMR (125 MHz, CDCl_3_) δ 150.44, 142.76, 125.39, 125.14, 70.17, 34.48, 31.34, 24.89.

**2-(4-Methoxyphenyl)propan-2-ol (1e)** spectral data were in agreement with literature values^11^

Following **GP4d**, 1-(4-methoxyphenyl)ethan-1-one (751 mg, 5 mmol), 3.0 M CH_3_MgBr (2.0 mL, 6 mmol) in 10 mL Et_2_O solution were stirred overnight at room temperature. Purification by silica gel chromatography with PE/EA (5:1) as eluting solvent afforded 2-(4-methoxyphenyl)propan-2-ol **1e** as colorless oil (471 mg, 57%).

^1^H NMR (300 MHz, CDCl_3_) δ 7.42 (d, *J* = 8.8 Hz, 1H), 6.87 (d, *J* = 8.8 Hz, 2H), 3.80 (s, 3H), 1. (br, 1H), 1.57 (s, 6H). ^13^C NMR (75 MHz, CDCl_3_) δ 158.30, 141.34, 125.58, 113.46, 72.16, 55.24, 31.75.

**1-(4-Methoxyphenyl)cyclohexan-1-ol (1f)** spectral data were in agreement with literature values^12^

Following **GP4d**, cyclohexanone (490 mg, 5 mmol), 0.5 M (4-methoxyphenyl)magnesium bromide (12.0 mL, 6 mmol) in 8 mL DCM solution were stirred for 3 h at room temperature. Purification by silica gel chromatography with PE/EA (10:1) as eluting solvent afforded 1-(4-methoxyphenyl)cyclohexan-1-ol **1f** as colorless oil (414 mg, 40%).

^1^H NMR (300 MHz, CDCl_3_) δ 7.43 (d, *J* = 8.8 Hz, 2H), 6.88 (d, *J* = 8.8 Hz, 2H), 3.80 (s, 3H), 1.86 – 1.52 (m, 11H). ^13^C NMR (75 MHz, CDCl_3_) δ 158.28, 141.59, 125.78, 113.46, 72.72, 55.22, 38.90, 25.50, 22.26.

**4-(1-Hydroxyethyl)phenol (1g)** spectral data were in agreement with literature values^13^

Following **GP4d**, 4-hydroxybenzaldehyde (733 mg, 6 mmol), 3.0 M CH_3_MgBr (6.0 mL, 18 mmol) in 20 mL THF solution were stirred overnight at room temperature. Purification by silica gel chromatography with PE/EA (5:1) as eluting solvent afforded 4-(1-hydroxyethyl)phenol **1g** as yellow solid (389 mg, 47%).

^1^H NMR (300 MHz, DMSO) δ 9.16 (s, 1H), 7.12 (d, *J* = 8.5 Hz, 2H), 6.68 (d, *J* = 8.5 Hz, 2H), 4.91 (d, *J* = 4.0 Hz, 1H), 4.60 (qd, *J* = 6.4, 4.0 Hz, 1H), 1.27 (d, *J* = 6.4 Hz, 3H). ^13^C NMR (75 MHz, DMSO) δ 155.95, 137.67, 126.41, 114.63, 67.76, 25.93.

**4-Acetylphenyl 4-methylbenzenesulfonate** was prepared according to a published procedure; spectral data were in agreement with literature values^14^

^1^H NMR (300 MHz, CDCl_3_) δ 7.89 (d, *J* = 8.8 Hz, 2H), 7.71 (d, *J* = 8.4 Hz, 2H), 7.32 (d, *J* = 8.4 Hz, 2H), 7.08 (d, *J* = 8.8 Hz, 2H), 2.57 (s, 3H), 2.45 (s, 3H). ^13^C NMR (75 MHz, CDCl_3_) δ 196.60, 152.96, 145.74, 135.67, 132.11, 130.01, 129.89, 128.44, 122.47, 26.57, 21.70.

**4-(1-Hydroxyethyl)phenyl 4-methylbenzenesulfonate (1i)**

Following **GP4a**, 4-acetylphenyl 4-methylbenzenesulfonate (871 mg, 3 mmol), NaBH_4_ (227 mg, 6 mmol) in 15 mL MeOH solution were stirred for 3 h at 0^o^C. Purification by silica gel chromatography with PE/EA (5:1) as eluting solvent afforded 4-(1-hydroxyethyl)phenyl 4-methylbenzenesulfonate **1i** as colorless liquid (843 mg, 96%).

^1^H NMR (300 MHz, CDCl_3_) δ 7.70 (d, *J* = 8.5 Hz, 2H), 7.31 (d, *J* = 6.8 Hz, 2H), 7.28 (d, *J* = 6.8 Hz, 2H), 6.95 (d, *J* = 8.5 Hz, 2H), 4.86 (q, *J* = 6.4 Hz, 1H), 2.45 (s, 3H), 1.89 (br, 1H), 1.45 (d, *J* = 6.4 Hz, 3H). ^13^C NMR (75 MHz, CDCl_3_) δ 148.69, 145.32, 144.68, 132.43, 129.73, 128.46, 126.59, 122.32, 69.62, 25.27, 21.68.

IR (EXTRACT): ṽ = 3549.68, 3388.69, 3065.92, 2971.44, 2946.75, 2927.26, 2866.93, 1909.46, 1596.63, 1501.03, 1453.96, 1369.39, 1292.22, 1196.71, 1174.85, 1153.67, 1108.85, 1090.67, 1016.28, 933.09, 863.24, 845.68, 814.47, 777.20, 741.31, 706.49, 682.31, 657.97cm^-1^

HRMS (EI+) m/z: [M]^+^ calcd 292.0764, found 292.0749.

**3-(1-Hydroxyethyl)-4-methoxybenzonitrile (1k)**

To a solution of 3-acetyl-4-hydroxybenzonitrile (483 mg, 3 mmol) in 10 mL acetone was added K_2_CO_3_ (1.24 g, 9 mmol), then the reaction mixture was stirred for 1h at room temperature. Then CH_3_I (554 mg, 3.9 mmol) was added to above mixture, and stirred for 24 h at room temperature. The reaction was diluted with 10 mL EA, washed with 20 mL H_2_O, 20 mL sat. brine, dried over Na_2_SO_4_, and concentrated in vacuo to afford crude 3-acetyl-4-methoxybenzonitrile, which was directly used for next step without further purification.

Following **GP4a**, crude 3-acetyl-4-methoxybenzonitrile, NaBH_4_ (227 mg, 6 mmol) in 15 mL MeOH solution were stirred for 3 h at 0^o^C. Purification by silica gel chromatography with PE/EA (2:1) as eluting solvent afforded 3-(1-hydroxyethyl)-4-methoxybenzonitrile **1k** as white solid (483 mg, 91%).

Mp: 95^o^C

^1^H NMR (300 MHz, CDCl_3_) δ 7.70 (d, *J* = 2.1 Hz, 1H), 7.55 (dd, *J* = 8.6, 2.1 Hz, 1H), 6.91 (d, *J* = 8.6 Hz, 1H), 5.11 (q, *J* = 6.4 Hz, 1H), 3.91 (s, 3H), 2.42 (br, 1H), 1.46 (d, *J* = 6.4 Hz, 3H). ^13^C NMR (75 MHz, CDCl_3_) δ 159.42, 135.33, 132.96, 129.86, 119.27, 110.76, 104.06, 64.98, 55.68, 23.01.

IR (EXTRACT): ṽ = 3234.74, 2977.50, 2929.08, 2849.15, 2223.54, 1602.48, 1493.21, 1456.64, 1439.44, 1369.01, 1347.42, 1320.92, 1294.92, 1258.01, 1188.21, 1161.28, 1134.83, 1072.76, 1018.51, 929.25, 911.05, 877.54, 830.79, 819.30, 766.65, 680.80, 614.10 cm^-1^

HRMS (EI+) m/z: [M]^+^ calcd 177.0784, found 177.0774.

**1-(2-methoxy-5-nitrophenyl)ethan-1-ol (1l)**

To a solution of 1-(2-hydroxy-5-nitrophenyl)ethan-1-one (362 mg, 2 mmol) in 10 mL acetone was added K_2_CO_3_ (829 mg, 6 mmol), then the reaction mixture was stirred for 1h at room temperature. Then CH_3_I (568 mg, 4 mmol) was added to above mixture, and stirred for 24 h at room temperature. The reaction was diluted with 10 mL EA, washed with 20 mL H_2_O, 20 mL sat. brine, dried over Na_2_SO_4_, and concentrated in vacuo. The crude residue was purified by silica gel chromatography with PE/EA (5:1) to afford 1-(2-methoxy-5-nitrophenyl)ethan-1-one as white solid (366 mg, 94%).

Following **GP4a**, 1-(2-methoxy-5-nitrophenyl)ethan-1-one (195 mg, 1 mmol), NaBH_4_ (75 mg, 2 mmol) in 15 mL MeOH solution were stirred for 3 h at 0^o^C. Purification by silica gel chromatography with PE/EA (2:1) as eluting solvent afforded 1-(2-methoxy-5-nitrophenyl)ethan-1-ol **1l** as white solid (194 mg, 98%).

^1^H NMR (300 MHz, CDCl_3_) δ 8.33 (d, *J* = 2.8 Hz, 1H), 8.16 (dd, *J* = 9.0, 2.8 Hz, 1H), 6.92 (d, *J* = 9.0 Hz, 1H), 5.16 (q, *J* = 6.4 Hz, 1H), 3.96 (s, 3H), 2.35 (br, 1H), 1.50 (d, *J* = 6.4 Hz, 3H). ^13^C NMR (75 MHz, CDCl_3_) δ 161.05, 141.63, 135.07, 124.62, 121.95, 110.04, 65.05, 56.12, 23.00.

**1,1'-(1,3-Phenylene)bis(ethan-1-ol) (1m)** spectral data were in agreement with literature values^15^

Following **GP4a**, 1,1'-(1,3-phenylene)bis(ethan-1-one) (487 mg, 3 mmol), NaBH_4_ (227 mg, 6 mmol) in 10 mL MeOH solution were stirred for 3 h at 0^o^C. Purification by silica gel chromatography with PE/EA (2:1) as eluting solvent afforded 1,1'-(1,3-phenylene)bis(ethan-1-ol) **1m** as colorless liquid (396 mg, 80%).

^1^H NMR (300 MHz, CDCl_3_) δ 7.41 – 7.34 (m, 1H), 7.33 – 7.29 (m, 1H), 7.26 – 7.22 (m, 2H), 4.86 (q, *J* = 6.4 Hz, 2H), 2.27 (br, 2H), 1.47 (d, *J* = 6.4 Hz, 6H). ^13^C NMR (75 MHz, CDCl_3_) δ 146.08, 146.06, 128.58, 124.50, 124.47, 122.41, 122.35, 70.31, 70.29, 25.12, 25.10.

**1-(2,4-Dimethylphenyl)ethan-1-ol (1n)** spectral data were in agreement with literature values^16^

Following **GP4a**, 1-(2,4-dimethylphenyl)ethan-1-one (741 mg, 5 mmol), NaBH_4_ (227 mg, 6 mmol) in 15 mL MeOH solution were stirred for 3 h at 0^o^C. Purification by silica gel chromatography with PE/EA (10:1) as eluting solvent afforded 1-(2,4-dimethylphenyl)ethan-1-ol **1n** as colorless liquid (746 mg, 99%).

^1^H NMR (300 MHz, CDCl_3_) δ 7.40 (d, *J* = 7.9 Hz, 1H), 7.05 (d, *J* = 7.9 Hz, 1H), 6.97 (s, 1H), 5.10 (q, *J* = 6.4 Hz, 1H), 2.33 (s, 3H), 2.32 (s, 3H), 1.73 (br, 1H), 1.47 (d, *J* = 6.4 Hz, 3H). ^13^C NMR (75 MHz, CDCl_3_) δ 140.85, 136.74, 134.16, 131.15, 126.96, 124.47, 66.69, 23.92, 20.90, 18.80.

**1-Mesitylethan-1-ol (1o)** spectral data were in agreement with literature values^17^

Following **GP4c**, 1-mesitylethan-1-one (811 mg, 5 mmol), LiAlH_4_ (949 mg, 25 mmol) in 20 mL THF solution were refluxed for 24 h. Purification by silica gel chromatography with PE/EA (10:1) as eluting solvent afforded 1-mesitylethan-1-ol **1o** as colorless solid (820 mg, 99%).

^1^H NMR (300 MHz, CDCl_3_) δ 6.82 (s, 2H), 5.37 (q, *J* = 6.7 Hz, 1H), 2.42 (s, 6H), 2.25 (s, 3H), 1.64 (br, 1H), 1.53 (d, *J* = 6.7 Hz, 3H). ^13^C NMR (75 MHz, CDCl_3_) δ 137.63, 136.40, 135.62, 130.11, 67.46, 21.57, 20.66, 20.48.

**1-(4-Phenoxyphenyl)ethan-1-ol (1q)** spectral data were in agreement with literature values^18^

Following **GP4a**, 1-(4-phenoxyphenyl)ethan-1-one (636 mg, 3 mmol), NaBH_4_ (227 mg, 6 mmol) in 10 mL MeOH solution were stirred for 3 h at 0^o^C. Purification by silica gel chromatography with PE/EA (10:1) as eluting solvent afforded 1-(4-phenoxyphenyl)ethan-1-ol **1q** as colorless liquid (640 mg, 99%).

^1^H NMR (300 MHz, CDCl_3_) δ 7.38 – 7.30 (m, 4H), 7.10 (t, *J* = 7.4 Hz, 1H), 7.03 – 6.97 (m, 4H), 4.90 (q, *J* = 6.4 Hz, 1H), 1.76 (br, 1H), 1.51 (d, *J* = 6.4 Hz, 3H). ^13^C NMR (75 MHz, CDCl_3_) δ 157.23, 156.54, 140.63, 129.71, 126.85, 123.22, 118.83, 118.81, 69.92, 25.13.

**Bis(4-bromophenyl)methanol (1s)** spectral data were in agreement with literature values^19^

Following **GP4a**, bis(4-bromophenyl)methanone (850 mg, 2.5 mmol), NaBH_4_ (113 mg, 3 mmol) in 10 mL MeOH solution were stirred for 3 h at 0^o^C. Purification by silica gel chromatography with PE/EA (10:1) as eluting solvent afforded bis(4-bromophenyl)methanol **1s** as colorless liquid (848 mg, 99%).

^1^H NMR (300 MHz, CDCl_3_) δ 7.46 (d, *J* = 8.4 Hz, 4H), 7.22 (d, *J* = 8.4 Hz, 4H), 5.75 (d, *J* = 2.9 Hz, 1H), 2.28 (d, *J* = 2.9 Hz, 1H). ^13^C NMR (75 MHz, CDCl_3_) δ 142.24, 131.70, 128.17, 121.74, 75.02.

**1-(Dibenzo[*b*,*d*]furan-2-yl)ethan-1-ol (1t)** spectral data were in agreement with literature values^20^

Following **GP4b**, dibenzo[*b*,*d*]furan (1.00 g, 6.0 mmol), acetyl chloride (498 uL, 6.6 mmol) and AlCl_3_ (880 mg, 6.6 mmol) in 15 mL CHCl_3_ solution were stirred for 4 h at room temperature. Simple workup afforded the crude 1-(dibenzo[*b*,*d*]furan-2-yl)ethan-1-one, which was directly used for next step without further purification.

The crude 1-(dibenzo[*b*,*d*]furan-2-yl)ethan-1-one, NaBH_4_ (272 mg, 7.2 mmol) in 20 mL MeOH solution were stirred for 3 h at 0^o^C. Purification by silica gel chromatography with PE/EA (10:1) as eluting solvent afforded 1-(dibenzo[*b*,*d*]furan-2-yl)ethan-1-ol **1t** as white solid (406 mg, 32%)

^1^H NMR (300 MHz, CDCl_3_) δ 7.98 (d, *J* = 1.8 Hz, 1H), 7.95 (dt, *J* = 7.6, 1.0 Hz, 1H), 7.59 – 7.51 (m, 2H), 7.50 – 7.42 (m, 2H), 7.35 (td, *J* = 7.6, 1.0 Hz, 1H), 5.07 (q, *J* = 6.4 Hz, 1H), 2.00 (br, 1H), 1.59 (d, *J* = 6.4 Hz, 3H). ^13^C NMR (75 MHz, CDCl_3_) δ 156.57, 155.58, 140.55, 127.18, 124.76, 124.27, 124.14, 122.70, 120.62, 117.43, 111.68, 111.48, 70.50, 25.64.

**9-Tosyl-9*H*-carbazole** was prepared according to a published procedure; spectral data were in agreement with literature values^1^

^1^H NMR (300 MHz, CDCl_3_) δ 8.34 (d, J = 8.4 Hz, 2H), 7.90 (dd, J = 7.7, 0.9 Hz, 2H), 7.70 (d, J = 8.4 Hz, 2H), 7.49 (td, J = 7.7, 0.9 Hz, 2H), 7.36 (td, J = 7.7, 0.9 Hz, 2H), 7.08 (d, J = 7.7 Hz, 2H), 2.25 (s, 3H). ^13^C NMR (75 MHz, CDCl_3_) δ 144.82, 138.38, 134.99, 129.61, 127.35, 126.45, 126.36, 123.85, 119.96, 115.14, 21.45.

**1-(9-Tosyl-9*H*-carbazol-3-yl)ethan-1-ol (1u)**

Following **GP4b**, 9-tosyl-9*H*-carbazole (321 mg, 1.0 mmol), acetyl chloride (90 uL, 1.2 mmol) and AlCl_3_ (160 mg, 1.2 mmol) in 5 mL DCE solution were stirred for 4 h at 0^o^C. Simple workup afforded the crude 1-(9-tosyl-9*H*-carbazol-3-yl)ethan-1-one, which was directly used for next step without further purification.

The crude 1-(9-tosyl-9*H*-carbazol-3-yl)ethan-1-one, NaBH_4_ (113 mg, 3 mmol) in 6 mL THF/H_2_O (5:1) solution were stirred for 3 h at 0^o^C. Purification by silica gel chromatography with PE/EA (2:1) as eluting solvent afforded 1-(9-tosyl-9*H*-carbazol-3-yl)ethan-1-ol **1u** as white solid (147 mg, 40%)

Mp: 59^o^C

^1^H NMR (300 MHz, CDCl_3_) δ 8.31 (d, *J* = 8.4 Hz, 1H), 8.27 (d, *J* = 8.4 Hz, 1H), 7.92 (d, *J* = 1.7 Hz, 1H), 7.89 (d, *J* = 7.6 Hz, 1H), 7.68 (d, *J* = 8.1 Hz, 2H), 7.51 – 7.44 (m, 2H), 7.35 (t, *J* = 7.6 Hz, 1H), 7.08 (d, *J* = 8.1 Hz, 2H), 5.05 (q, *J* = 6.5 Hz, 1H), 2.25 (s, 3H), 2.04 (br, 1H), 1.57 (d, *J* = 6.5 Hz, 3H).^13^C NMR (75 MHz, CDCl_3_) δ 144.84, 141.64, 138.67, 137.68, 134.93, 129.63, 127.40, 126.47, 126.45, 126.28, 124.99, 123.87, 119.98, 116.72, 115.10, 115.04, 70.29, 25.50, 21.46.

IR (EXTRACT): ṽ = 3546.22, 2972.78, 2926.28, 2872.96, 1911.69, 1798.83, 1711.58, 1597.97, 1484.33, 1444.30, 1366.10, 1305.90, 1227.75, 1207.33, 1170.69, 1154.28, 1117.55, 1089.33, 1032.29, 1020.23, 976.74, 898.56, 812.07, 771.31, 748.48, 703.29, 685.72, 659.77, 628.82 cm^-1^

HRMS (EI+) m/z: [M]^+^ calcd 365.1080, found 365.1062.

**5-(3,3-Dimethylbut-1-yn-1-yl)benzo[*d*][1,3]dioxole-4-carbaldehyde** was prepared according to a published procedure; spectral data were in agreement with literature values^21^

^1^H NMR (300 MHz, CDCl_3_) δ 10.42 (s, 1H), 6.99 (d, *J* = 8.0 Hz, 1H), 6.90 (d, *J* = 8.0 Hz, 1H), 6.13 (s, 2H), 1.32 (s, 9H). ^13^C NMR (75 MHz, CDCl_3_) δ 190.72, 148.29, 147.46, 126.90, 119.76, 119.26, 112.70, 103.67, 102.95, 74.72, 30.80, 28.21.

**1-(5-(3,3-Dimethylbut-1-yn-1-yl)benzo[*d*][1,3]dioxol-4-yl)ethan-1-ol (1v)**

Following **GP4d**, 5-(3,3-dimethylbut-1-yn-1-yl)benzo[*d*][1,3]dioxole-4-carbaldehyde (322 mg, 1.4 mmol), 3.0 M CH_3_MgBr (0.84 mL, 2.52 mmol) in 10 mL THF solution were stirred overnight at room temperature. Purification by silica gel chromatography with PE/EA (10:1) as eluting solvent afforded 1-(5-(3,3-dimethylbut-1-yn-1-yl)benzo[*d*][1,3]dioxol-4-yl)ethan-1-ol **1v** as yellow oil (342 mg, 100%).

^1^H NMR (300 MHz, CDCl_3_) δ 6.90 (d, *J* = 8.0 Hz, 1H), 6.64 (d, *J* = 8.0 Hz, 1H), 6.11 – 5.87 (m, 2H), 5.20 (dq, *J* = 9.1, 6.7 Hz, 1H), 2.88 (d, *J* = 9.1 Hz, 1H), 1.58 (d, *J* = 6.7 Hz, 3H), 1.31 (s, 9H). ^13^C NMR (75 MHz, CDCl_3_) δ 147.32, 144.15, 128.40, 126.67, 114.57, 107.13, 101.71, 101.25, 76.68, 67.00, 30.91, 28.11, 23.01.

IR (EXTRACT): ṽ = 3548.28, 3441.36, 2969.22, 2927.99, 2898.62, 2868.30, 2781.11, 1627.50, 1596.48, 1500.44, 1469.49, 1392.01, 1362.69, 1322.35, 1243.04, 1215.05, 1200.55, 1098.19, 1035.12, 990.45, 959.73, 931.32, 881.05, 865.82, 807.07, 775.27, 743.82, 640.13 cm^-1^

HRMS (EI+) m/z: [M]^+^ calcd 246.1251, found 246.1240.

**1-(2,3-Dihydrobenzofuran-5-yl)ethan-1-ol (1w)**

Following **GP4a**, 1-(2,3-dihydrobenzofuran-5-yl)ethan-1-one (800 mg, 5 mmol), NaBH_4_ (378 mg, 10 mmol) in 10 mL MeOH solution were stirred for 3 h at 0^o^C. Purification by silica gel chromatography with PE/EA (10:1) as eluting solvent afforded 1-(2,3-dihydrobenzofuran-5-yl)ethan-1-ol **1w** as colorless oil (640 mg, 78%).

^1^H NMR (300 MHz, CDCl_3_) δ 7.23 (d, *J* = 1.9 Hz, 1H), 7.09 (dd, *J* = 8.1, 1.9 Hz, 1H), 6.74 (d, *J* = 8.1 Hz, 1H), 4.83 (q, *J* = 6.4 Hz, 1H), 4.56 (t, *J* = 8.6 Hz, 2H), 3.20 (t, *J* = 8.6 Hz, 2H), 1.83 (br, 1H), 1.47 (d, *J* = 6.4 Hz, 3H). ^13^C NMR (75 MHz, CDCl_3_) δ 159.50, 138.04, 127.20, 125.38, 122.17, 108.95, 71.29, 70.24, 29.68, 25.09.

IR (EXTRACT): ṽ = 3270.02, 2968.76, 2894.62, 1612.88, 1490.11, 1434.95, 1410.68, 1367.91, 1347.25, 1320.98, 1304.89, 1288.00, 1231.04, 1134.72, 1103.48, 1070.58, 1011.37, 981.30, 945.46, 920.98, 896.57, 880.11, 825.91, 791.07, 733.33, 672.21, 607.21 cm^-1^

HRMS (EI+) m/z: [M]^+^ calcd 164.0832, found 164.0821.

**1-Tosylindoline** was prepared according to a published procedure; spectral data were in agreement with literature values^1^

^1^H NMR (300 MHz, CDCl_3_) δ 7.69 – 7.61 (m, 3H), 7.25 – 7.14 (m, 3H), 7.07 (d, *J* = 7.3 Hz, 1H), 6.96 (td, *J* = 7.4, 0.8 Hz, 1H), 3.91 (t, *J* = 8.4 Hz, 2H), 2.88 (t, *J* = 8.4 Hz, 2H), 2.36 (s, 3H). ^13^C NMR (75 MHz, CDCl_3_) δ 143.99, 141.98, 134.02, 131.72, 129.61, 127.67, 127.29, 125.06, 123.67, 114.99, 49.90, 27.85, 21.50.

**1-(1-Tosylindolin-5-yl)ethan-1-ol (1x)**

Following **GP4b**, 1-tosylindoline (273 mg, 1.0 mmol), acetyl chloride (90 uL, 1.2 mmol) and AlCl_3_ (160 mg, 1.2 mmol) in 5 mL DCE solution were stirred for 4 h at 0^o^C. Simple workup afforded the crude 1-(1-tosylindolin-5-yl)ethan-1-one, which was directly used for next step without further purification.

The crude 1-(1-tosylindolin-5-yl)ethan-1-one, NaBH_4_ (113 mg, 3 mmol) in 6 mL THF/H_2_O (5:1) solution were stirred for 3 h at 0^o^C. Purification by silica gel chromatography with PE/EA (2:1) as eluting solvent afforded 1-(1-tosylindolin-5-yl)ethan-1-ol **1x** as yellow oil (305 mg, 96%)

^1^H NMR (300 MHz, CDCl_3_) δ 7.67 (d, *J* = 8.2 Hz, 2H), 7.58 (d, *J* = 8.3 Hz, 1H), 7.22 (d, *J* = 8.2 Hz, 2H), 7.16 (dd, *J* = 8.3, 1.8 Hz, 1H), 7.11 (d, *J* = 1.8 Hz, 1H), 4.82 (q, *J* = 6.4 Hz, 1H), 3.90 (t, *J* = 8.4 Hz, 2H), 2.88 (t, *J* = 8.4 Hz, 2H), 2.37 (s, 3H), 1.83 (br, 1H), 1.44 (d, *J* = 6.4 Hz, 3H). ^13^C NMR (75 MHz, CDCl_3_) δ 144.02, 141.33, 141.31, 133.93, 132.03, 129.64, 127.30, 125.07, 122.24, 114.63, 69.97, 50.07, 27.81, 25.14, 21.50.

IR (EXTRACT): ṽ = 3539.14, 3064.62, 2971.59, 2924.92, 2873.82, 1920.31, 1709.98, 1612.42, 1597.09, 1485.19, 1443.45, 1399.44, 1357.43, 1305.26, 1293.56, 1248.09, 1185.15, 1169.60, 1110.01, 1092.48, 1017.42, 974.79, 923.69, 893.01, 831.20, 815.81, 735.55, 707.42, 663.26, 614.71cm^-1^

HRMS (EI+) m/z: [M]^+^ calcd 317.1080, found 317.1059.

**1-(3,5,5,6,8,8-Hexamethyl-5,6,7,8-tetrahydronaphthalen-2-yl)ethan-1-ol (1y)** spectral data were in agreement with literature values^22^

Following **GP4a**, 1-(3,5,5,6,8,8-hexamethyl-5,6,7,8-tetrahydronaphthalen-2-yl)ethan-1-one (517 mg, 2 mmol), NaBH_4_ (151 mg, 4 mmol) in 6 mL THF/MeOH (5:1) solution were stirred for 3 h at 0^o^C. Purification by silica gel chromatography with PE/EA (10:1) as eluting solvent afforded 1-(3,5,5,6,8,8-hexamethyl-5,6,7,8-tetrahydronaphthalen-2-yl)ethan-1-ol **1y** as colorless oil (520 mg, 100%).

major + minor diastereoisomers ^1^H NMR (300 MHz, CDCl_3_) δ 7.44 (d, *J* = 2.6 Hz, 1H), 7.12 (s, 1H), 5.08 (qd, *J* = 6.4, 2.6 Hz, 1H), 2.32 (s, 3H), 1.87 (dqd, *J* = 13.2, 6.6, 2.6 Hz, 1H), 1.70 (br, 1H), 1.64 (t, *J* = 13.2 Hz, 1H), 1.49 (dd, *J* = 6.4, 4.8 Hz, 3H), 1.37 (td,*J* = 13.2, 2.2 Hz, 1H), 1.33 (d, *J* = 3.9 Hz, 4H), 1.31 (s, 3H), 1.27 (d, *J* = 2.2 Hz, 3H), 1.07 (d, *J* = 6.6 Hz, 3H), 0.99 (d, *J* = 6.6 Hz, 3H). ^13^C NMR (75 MHz, CDCl_3_) δ 144.92, 142.76, 142.70, 140.90, 140.84, 131.46, 128.87, 128.80, 122.48, 122.38, 67.04, 43.77, 37.40, 34.60, 34.53, 34.23, 32.45, 32.37, 32.09, 32.06, 28.69, 28.44, 24.97, 24.92, 23.83, 23.72, 18.70, 16.81.

**3-(1-Hydroxyethyl)-2*H*-chromen-2-one (1z)** spectral data were in agreement with literature values^23^

To a mixture of 3-acetyl-2*H*-chromen-2-one (564 mg, 3mmol), CeCl_3_ (739 mg, 3 mmol) in 10 mL THF was added NaBH_4_ (113 mg, 3 mmol) at 0^o^C, and the reaction was stirred at 0^o^C for 4 h. The mixture was filtrated, concentrated in vacuo. The residue was purified by silica gel chromatography with PE/EA (3:1) as eluting solvent to afford 3-(1-hydroxyethyl)-2*H*-chromen-2-one **1z** as colorless liquid (285 mg, 50%)

^1^H NMR (300 MHz, CDCl_3_) δ 7.71 (s, 1H), 7.49 – 7.42 (m, 2H), 7.27 (d, *J* = 8.3 Hz, 1H), 7.24 – 7.18 (m, 1H), 4.87 (q, *J* = 6.5 Hz, 1H), 3.01 (br, 1H), 1.50 (d, *J* = 6.5 Hz, 3H). ^13^C NMR (75 MHz, CDCl_3_) δ 161.20, 153.03, 137.24, 132.01, 131.29, 127.93, 124.57, 119.09, 116.46, 66.05, 21.85.

**Methyl 5-(2,5-dimethyl phenoxy)-2,2-dimethyl pentanoate** spectral data were in agreement with literature values^1^

To a suspension of methyl 5-(2,5-dimethylphenoxy)-2,2-dimethylpentanoate (gemfibrozil, 300 mg, 1.2 mmol) and K_2_CO_3_ (216 mg, 1.56 mmol) in 3 mL DMF was added CH_3_I (276 mg, 2.4 mmol) at room temperature and the reaction was stirred overnight. Then the reaction was diluted with 10 mL EA and washed with sat. brine (10 mL × 3). The organic layer was dried over anhydrous Na_2_SO_4_, filtrated and concentrated in vacuo. The residue was purified by silica gel chromatography with PE/EA (30:1) as eluting solvent to afford the methyl 5-(2,5-dimethyl phenoxy)-2,2-dimethyl pentanoate as colorless liquid (298 mg, 94%).

^1^H NMR (300 MHz, CDCl_3_) δ 7.01 (d, *J* = 7.4 Hz, 1H), 6.66 (d, *J* = 7.4 Hz, 1H), 6.61 (s, 1H), 3.94 – 3.91(m, 2H), 3.67 (s, 3H), 2.31 (s, 3H), 2.18 (s, 3H), 1.74 – 1.71 (m, 4H), 1.23 (s, 6H). ^13^C NMR (75 MHz, CDCl_3_) δ 178.30, 156.92, 136.41, 130.26, 123.56, 120.65, 111.92, 67.86, 51.69, 42.08, 37.09, 25.17, 25.16, 21.37, 15.71.

**Methyl 5-(4-(1-hydroxyethyl)-2,5-dimethylphenoxy)-2,2-dimethylpentanoate (1aa)** spectral data were in agreement with literature values^24^

Following **GP4b**, methyl 5-(2,5-dimethyl phenoxy)-2,2-dimethyl pentanoate (205 mg, 0.78 mmol), acetyl chloride (0.17 mL, 2.4 mmol) and AlCl_3_ (320 mg, 2.4 mmol) in 5 mL DCE solution were stirred for 4 h at 0^o^C. Simple workup afforded the crude methyl 5-(4-acetyl-2,5-dimethylphenoxy)-2,2-dimethylpentanoate, which was directly used for next step without further purification.

The crude methyl 5-(4-acetyl-2,5-dimethylphenoxy)-2,2-dimethylpentanoate, NaBH_4_ (72 mg, 1.9 mmol) in 5 mL MeOH solution were stirred for 3 h at 0^o^C. Purification by silica gel chromatography with PE/EA (10:1) as eluting solvent afforded methyl 5-(4-(1-hydroxyethyl)-2,5-dimethylphenoxy)-2,2-dimethylpentanoate **1aa** as colorless oil (200 mg, 83%)

^1^H NMR (300 MHz, CDCl_3_) δ 7.24 (s, 1H), 6.55 (s, 1H), 5.06 (q, *J* = 6.4 Hz, 1H), 4.05 – 3.80 (m, 2H), 3.66 (s, 3H), 2.30 (s, 3H), 2.20 (s, 3H), 1.95 – 1.66 (m, 4H), 1.65 (br, 1H), 1.44 (d, *J* = 6.4 Hz, 3H), 1.22 (s, 6H). ^13^C NMR (75 MHz, CDCl_3_) δ 178.29, 155.99, 135.17, 132.69, 126.98, 124.42, 113.01, 68.05, 66.49, 51.70, 42.07, 37.06, 25.16, 25.14, 23.99, 18.84, 15.80.

**Methyl (*S*)-2-(6-methoxynaphthalen-2-yl)propanoate** spectral data were in agreement with literature values^1^

To 10 mL MeOH in a round bottom flask was added (*s*)-(+)-naproxen chloride (496 mg, 0.2 mmol) at room temperature, then it was stirred at room temperature for 2 h. The reaction was concentrated in vacuo, and the residue was purified by silica gel chromatography with PE/EA (10:1) as eluting solvent to afford methyl (*S*)-2-(6-methoxynaphthalen-2-yl)propanoate as white solid (463 mg, 95%)**.**

^1^H NMR (300 MHz, CDCl_3_) δ 7.71 (d, *J* = 8.5 Hz, 2H), 7.69 (s, 1H), 7.67 (d, *J* = 1.5 Hz, 1H), 7.41 (dd, *J* = 8.4, 1.5 Hz, 1H), 7.17 – 7.10 (m, 2H), 3.91 (s, 3H), 3.85 (q, *J* = 7.2 Hz, 1H), 3.67 (s, 3H), 1.59 (d, *J* = 7.2 Hz, 3H). ^13^C NMR (75 MHz, CDCl_3_) δ 175.11, 157.62, 135.65, 133.67, 129.24, 128.90, 127.15, 126.15, 125.90, 118.96, 105.57, 55.27, 52.00, 45.31, 18.56.

**Methyl (2*S*)-2-(5-(1-hydroxyethyl)-6-methoxynaphthalen-2-yl)propanoate (1ab)**

Following **GP4b**, methyl (*S*)-2-(6-methoxynaphthalen-2-yl)propanoate (244 mg, 1.0 mmol), acetyl chloride (0.21 mL, 3.0 mmol) and AlCl_3_ (400 mg, 3 mmol) in 5 mL DCE solution were stirred for 4 h at 0^o^C. Simple workup afforded the crude methyl (*S*)-2-(5-acetyl-6-methoxynaphthalen-2-yl)propanoate, which was directly used for next step without further purification.

The crude methyl (*S*)-2-(5-acetyl-6-methoxynaphthalen-2-yl)propanoate, NaBH_4_ (90 mg, 2.4 mmol) in 6 mL THF/MeOH (5:1) solution was stirred for 3 h at 0^o^C. Purification by silica gel chromatography with PE/EA (5:1) as eluting solvent afforded methyl (2*S*)-2-(5-(1-hydroxyethyl)-6-methoxynaphthalen-2-yl)propanoate **1ab** as yellow oil (181 mg, 63% for two steps)

major + minor diastereoisomers ^1^H NMR (300 MHz, CDCl_3_) δ 8.08 (d, *J* = 9.1 Hz, 1H), 7.75 (d, *J* = 9.1 Hz, 1H), 7.68 (d, *J* = 2.0 Hz, 1H), 7.44 (dd, *J* = 9.0, 2.0 Hz, 1H), 7.28 (d, *J* = 9.0 Hz, 1H), 5.77– 5.66 (m, 1H), 4.00 (s, 3H), 3.90 (br, 1H), 3.86 (q, *J* = 7.1 Hz, 1H), 3.67 (s, 3H), 1.65 (d, *J* = 6.8 Hz, 3H), 1.58 (d, *J* = 7.1 Hz, 3H). ^13^C NMR (75 MHz, CDCl_3_) δ 174.98, 154.22, 135.62, 135.60, 130.37, 129.37, 128.93, 126.73, 126.67, 126.52, 126.44, 125.56, 123.40, 123.38, 113.64, 66.04, 56.36, 52.04, 45.12, 45.10, 23.76, 18.45, 18.39.

IR (EXTRACT): ṽ = 3528.45, 3440.74, 2975.41, 2950.34, 2841.16, 1733.51, 1627.72, 1598.87, 1504.27, 1480.40, 1454.76, 1373.64, 1329.36, 1247.76, 1197.33, 1163.68, 1116.15, 1075.68, 1023.81, 995.91, 972.81, 921.29, 886.81, 830.13, 804.67, 761.08, 692.56, 662.89, 613.05 cm^-1^

HRMS (EI+) m/z: [M]^+^ calcd 288.1356, found 288.1347.

**Methyl 2-(4-(4-chlorobenzoyl)phenoxy)-2-methylpropanoate**

To a solution of 2-(4-(4-chlorobenzoyl)phenoxy)-2-methylpropanoic acid (956 mg, 3 mmol) in 30 mL MeOH was dropwise added SOCl_2_ (1.09 mL, 15 mmol) at 0^o^C, then the reaction was warmed up to room temperature and stirred overnight. The reaction was concentrated in vacuo, and the residue was purified by silica gel chromatography with PE/EA (10:1) as eluting solvent to afford methyl 2-(4-(4-chlorobenzoyl)phenoxy)-2-methylpropanoate as white solid (990 mg, 99%).

^1^H NMR (300 MHz, CDCl_3_) δ 7.73 (d, *J* = 8.9 Hz, 2H), 7.70 (s, d, *J* = 8.5 Hz, 2H), 7.44 (d, *J* = 8.5 Hz, 2H), 6.85 (d, *J* = 8.9 Hz, 2H), 3.77 (s, 3H), 1.67 (s, 6H). ^13^C NMR (75 MHz, CDCl_3_) δ 194.19, 174.20, 159.55, 138.38, 136.34, 132.02, 131.15, 130.48, 128.53, 117.30, 79.35, 52.68, 25.39.

**Methyl 2-(4-((4-chlorophenyl)(hydroxy)methyl)phenoxy)-2-methylpropanoate (1ac)**

Following **GP4a**, methyl 2-(4-(4-chlorobenzoyl)phenoxy)-2-methylpropanoate (665 mg, 2.0 mmol), NaBH_4_ (151 mg, 4.0 mmol) in 10 mL MeOH solution were stirred for 3 h at 0^o^C. Purification by silica gel chromatography with PE/EA (8:1) as eluting solvent afforded methyl 2-(4-((4-chlorophenyl)(hydroxy)methyl)phenoxy)-2-methylpropanoate **1ac** as colorless liquid (666 mg, 99%).

^1^H NMR (300 MHz, CDCl_3_) δ 7.29 (s, 4H), 7.19 (d, *J* = 8.6 Hz, 2H), 6.78 (d, *J* = 8.6 Hz, 2H), 5.75 (s, 1H), 3.75 (s, 3H), 2.25 (br, 1H), 1.58 (s, 6H). ^13^C NMR (75 MHz, CDCl_3_) δ 174.72, 154.98, 142.23, 137.14, 133.16, 128.52, 127.79, 127.62, 119.00, 79.09, 75.10, 52.50, 25.33, 25.31.

IR (EXTRACT): ṽ = 3505.56, 2993.62, 2950.71, 2874.03, 1904.78, 1736.44, 1608.44, 1583.70, 1507.48, 1489.22, 1466.18, 1436.05, 1404.33, 1383.97, 1365.82, 1289.67, 1239.64, 1175.95, 1145.83, 1089.37, 1038.61, 1013.31, 962.72, 891.23, 828.76, 800.92, 775.01, 735.29, 686.17, 619.33 cm^-1^

HRMS (EI+) m/z: [M]^+^ calcd 334.0966, found 334.0951.

**Ethyl 3-(3,4-dimethoxyphenyl)-3-hydroxy-2-(2-methoxyphenoxy)propanoate** spectral data were in agreement with literature values^25^

To a shrank flask with 15 mL THF was slowly added 2 M LDA (8.25 mL, 16.5 mmol) at -78^o^C under N_2_ atmosphere, then it’s stirred for 15 min at -78^o^C. Then ethyl 2-(2-methoxyphenoxy)acetate (3.15 g, 15 mmol) in 30 mL THF solution was dropwise added to the above mixture at -78^o^C, then the mixture was stirred for 10 min at -78^o^C. A solution of 3,4-dimethoxybenzaldehyde (2.49 g, 15 mmol) in 30 mL THF solution was dropwise added to the above mixture at -78^o^C and the reaction was stirred for 2 h. The reaction was warmed up to room temperature, and quenched with 20 mL H_2_O, extracted with EA (80 mL × 3), then the combined organic layers were washed with 100 mL 1N HCl aq., 100 mL sat. brine, concentrated in vacuo. the residue was purified by silica gel chromatography with PE/EA (2:1) as eluting solvent to afford ethyl 3-(3,4-dimethoxyphenyl)-3-hydroxy-2-(2-methoxyphenoxy)propanoate as white solid (3.70 g, 66%).

major + minor diastereoisomers ^1^H NMR (400 MHz, CDCl_3_) δ 7.15 – 6.66 (m, 7H), 5.14 and 5.06 (d, *J* = 5.0 Hz and d, *J* = 7.1 Hz, 1H), 4.74 and 4.49 (d, *J* = 5.0 Hz and d, *J* = 7.1 Hz, 1H), 4.13 (q, *J* = 7.1 Hz, 1H), 4.11 – 3.99 (m, 1H), 3.90 – 3.82 (m, 9H), 1.15 and 1.07 (t, *J* = 7.1 Hz and t, *J* = 7.1 Hz, 3H). ^13^C NMR (100 MHz, CDCl_3_) δ 169.38, 169.31, 150.59, 150.39, 149.12, 148.97, 148.81, 148.75, 147.34, 147.26, 131.74, 130.61, 123.89, 121.08, 121.04, 119.62, 119.31, 118.89, 118.28, 112.32, 112.29, 110.79, 110.76, 110.21, 110.08, 85.51, 83.89, 74.84, 73.81, 61.18, 61.16, 55.88, 55.86, 55.83, 55.79, 14.03, 13.89.

**1-(3,4-Dimethoxyphenyl)-2-(2-methoxyphenoxy)propane-1,3-diol (1ad)** spectral data were in agreement with literature values^26^

Following **GP4c**, ethyl 3-(3,4-dimethoxyphenyl)-3-hydroxy-2-(2-methoxyphenoxy)propanoate (1.88 g, 5 mmol), LiAlH_4_ (474 mg, 12.5 mmol) in 40 mL THF solution were refluxed for 2 h. Purification by silica gel chromatography with PE/EA (1:1) as eluting solvent afforded 1-(3,4-dimethoxyphenyl)-2-(2-methoxyphenoxy)propane-1,3-diol **1ad** as colorless oil (1.65 g, 99%).

major + minor diastereoisomers ^1^H NMR (300 MHz, CDCl_3_) δ 7.19 – 6.76 (m, 7H), 5.00 – 4.99 (m, 1H), 4.20 – 4.07 (m, 1H), 3.95 – 3.90 (m, 1H), 3.89 (s, 3H), 3.88 (s, 3H), 3.87 (s, 3H), 3.72 – 3.44 (m, 1H), 2.60 (br, 2H). ^13^C NMR (75 MHz, CDCl_3_) δ 151.59, 151.30, 149.08, 149.02, 148.90, 148.46, 147.58, 146.88, 132.45, 132.09, 124.25, 124.20, 121.67, 121.61, 121.04, 121.00, 119.60, 118.38, 112.16, 111.01, 109.89, 109.20, 89.47, 87.39, 73.90, 72.67, 61.02, 60.73, 55.88, 55.86.

**(8*R*,9*S*,13*S*,14*S*)-13-Methyl-17-oxo-7,8,9,11,12,13,14,15,16,17-decahydro-6*H*-cyclopenta[*a*]phenanthren-3-yl trifluoromethanesulfonate** spectral data were in agreement with literature values^27^

To a solution of estrone (1.0 g, 3.7 mmol), pyridine (0.6 mL, 7.4 mmol) in 20 mL DCM was dropwise added Tf_2_O (0.75 mL, 4.4 mmol) at 0^o^C, and the reaction was stirred at 0^o^C for 2 h. The reaction was quenched with 20 mL H_2_O, extracted with DCM (20 mL × 3), and the combined organic layers were washed with 30 mL sat. brine, dried over Na_2_SO_4_, concentrated in vacuo. The residue was purified by silica gel chromatography with PE/EA (10:1) as eluting solvent to afford (8*R*,9*S*,13*S*,14*S*)-13-methyl-17-oxo-7,8,9,11,12,13,14,15,16,17-decahydro-6*H*-cyclopenta[*a*]phenanthren-3-yl trifluoromethanesulfonate as white solid (1.48 g, 99%)

^1^H NMR (300 MHz, CDCl_3_) δ 7.34 (d, *J* = 8.6 Hz, 1H), 7.03 (dd, *J* = 8.6, 2.8 Hz, 2H), 6.99 (d, *J* = 2.8 Hz, 1H), 2.94 (dd, *J* = 8.9, 4.3 Hz, 3H), 2.52 (dd, *J* = 18.3, 8.4 Hz, 1H), 2.45 – 2.25 (m, 2H), 2.22 – 1.94 (m, 4H), 1.77 – 1.36 (m, 6H), 0.92 (s, 3H). ^13^C NMR (75 MHz, CDCl_3_) δ 220.36, 147.56, 140.25, 139.27, 127.16, 121.20, 118.72 (q, *J* = 320.7 Hz), 118.27, 50.35, 47.82, 44.06, 37.72, 35.76, 31.45, 29.35, 26.05, 25.65, 21.53, 13.76. ^19^F NMR (283 MHz, CDCl_3_) δ -72.96.

**(8*R*,9*S*,13*S*,14*S*)-3-Acetyl-13-methyl-6,7,8,9,11,12,13,14,15,16-decahydro-17*H*-cyclopenta[*a*]phenanthren-17-one** spectral data were in agreement with literature values^28^

To a mixture of (8*R*,9*S*,13*S*,14*S*)-13-methyl-17-oxo-7,8,9,11,12,13,14,15,16,17-decahydro-6*H*-cyclopenta[*a*]phenanthren-3-yl trifluoromethanesulfonate (603 mg, 1.5 mmol), 1-(vinyloxy)butane (775 uL, 6 mmol), Pd(OAc)_2_ (33.6 mg, 0.15 mmol), dppp (68 mg, 0.165 mmol) in 5 mL anhydrous DMF was added Et_3_N (417 uL, 3 mmol) under N_2_ atmosphere at room temperature. Then the reaction was warmed up to 80^o^C, and stirred overnight. The reaction was cooled down to room temperature, and was quenched with 5 mL 2 M HCl aq., and stirred for 30 min. The mixture was filtrated with celite, extracted with EA (10 mL × 3), and the combined organic layers were washed with sat. brine (20 mL × 3), dried over Na_2_SO_4_, concentrated in vacuo. The residue was purified by silica gel chromatography with PE/EA (5:1) as eluting solvent to afford (8*R*,9*S*,13*S*,14*S*)-3-acetyl-13-methyl-6,7,8,9,11,12,13,14,15,16-decahydro-17*H*-cyclopenta[*a*]phenanthren-17-one as white solid (381 mg, 86%)

^1^H NMR (400 MHz, CDCl_3_) δ 7.72 (dd, *J* = 8.0, 1.9 Hz, 1H), 7.69 (d, *J* = 1.9 Hz, 1H), 7.37 (d, *J* = 8.0 Hz, 1H), 2.96 (td, *J* = 6.9, 2.1 Hz, 2H), 2.57 (s, 3H), 2.55 – 2.47 (m, 1H), 2.47 – 2.28 (m, 2H), 2.22 – 1.90 (m, 4H), 1.73 – 1.40 (m, 6H), 0.91 (s, 3H). ^13^C NMR (100 MHz, CDCl_3_) δ 220.40, 198.03, 145.42, 136.88, 134.86, 128.88, 125.80, 125.53, 50.51, 47.83, 44.68, 37.81, 35.75, 31.51, 29.26, 26.50, 26.25, 25.54, 21.54, 13.76.

**(8*R*,9*S*,13*S*,14*S*)-3-(1-Hydroxyethyl)-13-methyl-7,8,9,11,12,13,14,15,16,17-decahydro-6*H*-cyclopenta[*a*]phenanthren-17-ol (1ae)**

Following **GP4a**, (8*R*,9*S*,13*S*,14*S*)-3-acetyl-13-methyl-6,7,8,9,11,12,13,14,15,16-decahydro-17*H*-cyclopenta[*a*]phenanthren-17-one (296 mg, 1 mmol), NaBH_4_ (91 mg, 2.5 mmol) in 6 mL THF/H_2_O (5:1) solution were stirred for 3 h at 0^o^C. Purification by silica gel chromatography with PE/EA (3:1) as eluting solvent afforded (8*R*,9*S*,13*S*,14*S*)-3-(1-hydroxyethyl)-13-methyl-7,8,9,11,12,13,14,15,16,17-decahydro-6*H*-cyclopenta[*a*]phenanthren-17-ol **1ae** as white solid (287 mg, 96%).

Mp: 145^o^C

major + minor diastereoisomers ^1^H NMR (400 MHz, CDCl_3_) δ 7.29 (d, *J* = 8.1 Hz, 1H), 7.15 (d, *J* = 8.1 Hz, 1H), 7.11 (s, 1H), 4.84 (q, *J* = 6.4 Hz, 1H), 3.73 (t, *J* = 8.5 Hz, 1H), 3.07 – 2.80 (m, 2H), 2.42 – 2.33 (m, 1H), 2.28 – 2.20 (m, 1H), 2.18 – 2.07 (m, 1H), 2.01 – 1.86 (m, 2H), 1.77 – 1.67 (m, 1H), 1.65 (br, 1H), 1.59 – 1.51 (m, 1H), 1.49 (d, *J* = 6.4 Hz, 3H), 1.48 – 1.16 (m, 6H), 0.78 (s, 3H). ^13^C NMR (100 MHz, CDCl_3_) δ 143.06, 139.65, 136.89, 126.00, 125.98, 125.53, 122.73, 122.71, 81.85, 70.20, 70.15, 50.15, 44.35, 43.21, 38.63, 36.72, 30.56, 29.60, 29.57, 27.19, 26.12, 24.93, 24.91, 23.11, 11.01.

IR (EXTRACT): ṽ = 3737.20, 3372.69, 2909.71, 2868.41, 2848.07, 2359.45, 1498.42, 1449.09, 1380.65, 1360.21, 1339.26, 1318.94, 1264.01, 1245.62, 1214.74, 1157.16, 1136.04, 1090.30, 1076.40, 1055.61, 1021.23, 1010.44, 962.98, 920.10, 897.04, 862.57, 849.70, 818.22, 784.07, 718.17, 655.01, 609.07 cm^-1^

HRMS (EI+) m/z: [M]^+^ calcd 300.2084, found 300.2085.

**1-(1-Cyclopropylvinyl)-4-methoxybenzene (1af)** was prepared according to a published procedure; spectral data were in agreement with literature values^29^

^1^H NMR (300 MHz, CDCl_3_) δ 7.55 (d, *J* = 8.8 Hz, 2H), 6.88 (d, *J* = 8.8 Hz, 2H), 5.20 (d, *J* = 1.1 Hz, 1H), 4.86 (t, *J* = 1.2 Hz, 1H), 3.82 (s, 3H), 1.68 – 1.57 (m, 1H), 1.00 – 0.75 (m, 2H), 0.63 – 0.49 (m, 2H).

**1-(4-Methoxyphenyl)propan-2-ol (1ag)** spectral data were in agreement with literature values^30^

Following **GP4a**, 1-(4-methoxyphenyl)propan-2-one (821 mg, 5 mmol), NaBH_4_ (227 mg, 6 mmol) in 10 mL MeOH solution were stirred for 3 h at 0^o^C. Purification by silica gel chromatography with PE/EA (10:1) as eluting solvent afforded 1-(4-methoxyphenyl)propan-2-ol **1ag** as colorless oil (820 mg, 99%).

^1^H NMR (300 MHz, CDCl_3_) δ 7.13 (d, *J* = 8.6 Hz, 2H), 6.86 (d, *J* = 8.6 Hz, 2H), 4.05 – 3.89 (m, 1H), 3.79 (s, 3H), 2.80 – 2.46 (m, 2H), 1.61 (br, 1H), 1.23 (d, *J* = 6.1 Hz, 3H). ^13^C NMR (75 MHz, CDCl_3_) δ 158.27, 130.44, 130.30, 113.95, 68.90, 55.22, 44.81, 22.65.

**1-(3,4-Dimethoxyphenyl)ethan-1-ol (1ah)** spectral data were in agreement with literature values^26^

Following **GP4d**, 3,4-dimethoxybenzaldehyde (831 mg, 5.0 mmol), 3.0 M CH_3_MgBr (2.0 mL, 6 mmol) in 10 mL THF solution were stirred overnight at room temperature. Purification by silica gel chromatography with PE/EA (3:1) as eluting solvent afforded 1-(3,4-dimethoxyphenyl)ethan-1-ol **1ah** as yellow oil (835 mg, 92%).

^1^H NMR (400 MHz, CDCl_3_) δ 6.94 (d, *J* = 2.0 Hz, 1H), 6.88 (dd, *J* = 8.2, 2.0 Hz, 1H), 6.83 (d, *J* = 8.2 Hz, 1H), 4.84 (q, *J* = 6.4 Hz, 1H), 3.89 (s, 3H), 3.86 (s, 3H), 1.87 (br, 1H), 1.48 (d, *J* = 6.4 Hz, 3H).^13^C NMR (100 MHz, CDCl_3_) δ 149.07, 148.37, 138.56, 117.50, 111.03, 108.69, 70.17, 55.92, 55.82, 25.04.

***N*-(4-(1-hydroxyethyl)phenyl)acetamide (1ak)** spectral data were in agreement with literature values^31^

Following **GP4a**, *N*-(4-acetylphenyl)acetamide (177 mg, 1 mmol), NaBH_4_ (76 mg, 2 mmol) in 5 mL MeOH solution were stirred for 3 h at 0^o^C. Purification by silica gel chromatography with PE/EA (1:1) as eluting solvent afforded *N*-(4-(1-hydroxyethyl)phenyl)acetamide **1ak** as colorless oil (167 mg, 93%).

^1^H NMR (300 MHz, CDCl_3_) δ 7.74 (br, 1H), 7.41 (d, *J* = 8.6 Hz, 2H), 7.26 (d, *J* = 8.6 Hz, 2H), 4.83 (q, *J* = 6.4 Hz, 1H), 2.11 (s, 3H), 1.44 (d, *J* = 6.4 Hz, 3H). ^13^C NMR (75 MHz, CDCl_3_) δ 168.74, 141.80, 137.01, 125.99, 120.17, 69.84, 25.02, 24.37.

**1-(2,3-Dihydro-1*H*-inden-5-yl)ethan-1-ol (1al)**

Following **GP4a**, 1-(2,3-dihydro-1*H*-inden-5-yl)ethan-1-one (800 mg, 5 mmol), NaBH_4_ (227 mg, 6 mmol) in 10 mL MeOH solution were stirred for 3 h at 0^o^C. Purification by silica gel chromatography with PE/EA (10:1) as eluting solvent afforded 1-(2,3-dihydro-1*H*-inden-5-yl)ethan-1-ol **1al** as colorless liquid (640 mg, 79%).

^1^H NMR (300 MHz, CDCl_3_) δ 7.26 (s, 1H), 7.21 (d, *J* = 7.7 Hz, 1H), 7.15 (d, *J* = 7.7 Hz, 1H), 4.88 (q, *J* = 6.4 Hz, 1H), 2.91 (td, *J* = 7.4, 3.6 Hz, 4H), 2.09 (p, *J* = 7.4 Hz, 2H), 1.84 (br, 1H), 1.50 (d, *J* = 6.4 Hz, 3H). ^13^C NMR (75 MHz, CDCl_3_) δ 144.58, 143.86, 143.57, 124.30, 123.37, 121.39, 70.53, 32.78, 32.52, 25.50, 25.15.

IR (EXTRACT): ṽ = 3348.20, 3006.72, 2966.29, 2888.90, 2866.10, 2843.17, 1889.66, 1764.66, 1613.50, 1581.67, 1490.88, 1438.72, 1367.50, 1338.47, 1293.16, 1262.40, 1232.27, 1193.14, 1141.37, 1072.03, 1009.50, 926.37, 876.04, 821.24, 780.34, 709.84 cm^-1^

HRMS (EI+) m/z: [M]^+^ calcd 162.1039 found 162.1034.

**1-(2,6-dimethoxypyridin-3-yl)ethan-1-ol (1am)** spectral data were in agreement with literature values^32^

Following **GP4d**, 2,6-dimethoxynicotinaldehyde (334 mg, 2 mmol), 3.0 M CH_3_MgBr (0.8 mL, 1.2 mmol) in 10 mL THF solution were stirred overnight at room temperature. Purification by silica gel chromatography with PE/EA (5:1) as eluting solvent afforded 1-(2,6-dimethoxypyridin-3-yl)ethan-1-ol **1am** as yellow oil (350 mg, 96%).

^1^H NMR (300 MHz, CDCl_3_) δ 7.53 (d, *J* = 8.0 Hz, 1H), 6.29 (d, *J* = 8.0 Hz, 1H), 4.97 (q, *J* = 6.5 Hz, 1H), 3.97 (s, 3H), 3.90 (s, 3H), 1.47 (d, *J* = 6.5 Hz, 3H). ^13^C NMR (125 MHz, CDCl_3_) δ 162.02, 159.32, 137.63, 118.44, 100.39, 65.28, 53.54, 53.24, 22.47.

**methyl 4-(1-hydroxyethyl)thiophene-2-carboxylate (1an)**

To a solution of methyl 4-acetylthiophene-2-carboxylate (631 mg, 5 mmol) in MeOH (1.62 mL, 40 mmol) and CCl_4_ (1.93 mL, 20 mmol) was added Fe(acac)_3_ (35 mg, 0.1 mmol) under nitrogen atmosphere, and then the reaction was stirred at 140^o^C for 2 days. The reaction was cooled down to room temperature, concentrated in vacuo. The crude residue was purified by silica gel chromatography with PE/EA (15:1) to afford methyl 4-acetylthiophene-2-carboxylate as white solid (222 mg, 24%).

Following **GP4a**, methyl 4-acetylthiophene-2-carboxylate (222 mg, 1.2 mmol), NaBH_4_ (91 mg, 2.4 mmol) in 5 mL MeOH solution were stirred for 3 h at 0^o^C. Purification by silica gel chromatography with PE/EA (10:1) as eluting solvent afforded methyl 4-(1-hydroxyethyl)thiophene-2-carboxylate **1an** as colorless solid (166 mg, 74%).

^1^H NMR (300 MHz, CDCl_3_) δ 7.76 (d, *J* = 1.5 Hz, 1H), 7.41 (dd, *J* = 1.5, 0.8 Hz, 1H), 4.93 (q, *J* = 6.4 Hz, 1H), 3.87 (s, 3H), 2.04 (br, 1H), 1.51 (d, *J* = 6.4 Hz, 3H). ^13^C NMR (75 MHz, CDCl_3_) δ 162.61, 148.05, 133.98, 131.80, 127.11, 66.28, 52.17, 24.58.

IR (Reflection): ṽ = 3414, 3095, 2973, 2953, 2929, 1693, 1545, 1439, 1249, 1191, 1155, 1067, 1025, 964, 923, 879, 846, 785, 748, 656

HRMS (EI+) m/z: [M]^+^ calcd 186.0345, found 163.1345186.0352

**1-([1,1'-Biphenyl]-2-yl)ethan-1-ol (1ao)** spectral data were in agreement with literature values^33^

Following **GP4d**, [1,1'-biphenyl]-2-carbaldehyde (547 mg, 3 mmol), 3.0 M CH_3_MgBr (1.5 mL, 4.5 mmol) in 15 mL THF solution were stirred overnight at room temperature. Purification by silica gel chromatography with PE/EA (5:1) as eluting solvent afforded 1-([1,1'-biphenyl]-2-yl)ethan-1-ol **1ao** as yellow oil (511 mg, 86%).

^1^H NMR (300 MHz, CDCl_3_) δ 7.68 (dd, *J* = 7.6, 1.4 Hz, 1H), 7.46 – 7.36 (m, 4H), 7.36 – 7.29 (m, 3H), 7.22 (dd, *J* = 7.6, 1.4 Hz, 1H), 4.99 (q, *J* = 6.4 Hz, 1H), 1.73 (br, 1H), 1.42 (d, *J* = 6.4 Hz, 3H). ^13^C NMR (75 MHz, CDCl_3_) δ 143.05, 140.90, 140.32, 129.93, 129.25, 128.14, 127.96, 127.10, 127.08, 125.32, 66.44, 24.87.

**1-(2,6-Dimethylphenyl)ethan-1-ol (1ap)** spectral data were in agreement with literature values^34^

Following **GP4c**, 1-mesitylethan-1-one (593 mg, 4 mmol), LiAlH_4_ (759 mg, 20 mmol) in 20 mL THF solution were refluxed for 36 h. Purification by silica gel chromatography with PE/EA (5:1) as eluting solvent afforded 1-(2,6-dimethylphenyl)ethan-1-ol **1ap** as colorless solid (586 mg, 98%).

^1^H NMR (300 MHz, CDCl_3_) δ 7.09 – 6.96 (m, 3H), 5.39 (q, *J* = 6.8 Hz, 1H), 2.46 (s, 6H), 1.78 (br, 1H), 1.54 (d, *J* = 6.8 Hz, 3H). ^13^C NMR (75 MHz, CDCl_3_) δ 140.52, 135.66, 129.38, 126.91, 67.64, 21.43, 20.60.

**Methyl 4-phenylbutanoate** spectral data were in agreement with literature values^35^

To a solution of 4-phenylbutanoic acid (1.64 g, 10 mmol) in 50 mL MeOH was dropwise added SOCl_2_ (3.63 mL, 50 mmol) at 0^o^C, and then the reaction was stirred overnight at room temperature. The reaction was concentrated in vacuo, and the residue was purified by silica gel chromatography with PE/EA (10:1) as eluting solvent to afford methyl 4-phenylbutanoate as colorless liquid (1.68 g, 94%).

^1^H NMR (300 MHz, CDCl_3_) δ 7.33 – 7.25 (m, 2H), 7.24 – 7.13 (m, 3H), 3.67 (s, 3H), 2.65 (t, *J* = 7.4 Hz, 2H), 2.34 (t, *J* = 7.4 Hz, 2H), 2.06 – 1.88 (m, 2H). ^13^C NMR (75 MHz, CDCl_3_) δ 173.91, 141.34, 128.46, 128.36, 125.96, 51.47, 35.10, 33.37, 26.45.

**Methyl 4-(4-(1-hydroxyethyl)phenyl)butanoate (1aq)**

Following **GP4b**, methyl 4-phenylbutanoate (1.07 g, 10 mmol), acetyl chloride (0.91 mL, 12 mmol) and AlCl_3_ (2.40 g, 18 mmol) in 30 mL DCM solution were stirred for overnight. Simple workup afforded the crude methyl 4-(4-acetylphenyl)butanoate, which was directly used for next step without further purification.

The crude methyl 4-(4-acetylphenyl)butanoate, NaBH_4_ (454 mg, 12 mmol) in 20 mL MeOH solution were stirred for 3 h at 0^o^C. Purification by silica gel chromatography with PE/EA (5:1) as eluting solvent afforded methyl 4-(4-(1-hydroxyethyl)phenyl)butanoate **1aq** as yellow oil (977 mg, 73% for two steps)

^1^H NMR (300 MHz, CDCl_3_) δ 7.29 (d, *J* = 8.1 Hz, 2H), 7.16 (d, *J* = 8.1 Hz, 2H), 4.87 (q, *J* = 6.5 Hz, 1H), 3.66 (s, 3H), 2.63 (t, *J* = 7.4 Hz, 2H), 2.32 (t, *J* = 7.4 Hz, 2H), 2.00 – 1.91 (m, 2H), 1.89 (br, 1H), 1.48 (d, *J* = 6.5 Hz, 3H). ^13^C NMR (75 MHz, CDCl_3_) δ 173.91, 143.50, 140.59, 128.56, 125.46, 70.17, 51.48, 34.72, 33.33, 26.43, 25.04.

IR (EXTRACT): ṽ = 3428.39, 2969.88, 2951.67, 2929.75, 2865.67, 1908.64, 1736.36, 1613.57, 1513.29, 1437.33, 1367.18, 1249.09, 1200.71, 1176.70, 1146.26, 1116.45, 1088.69, 1006.20, 897.20, 839.75, 818.32, 706.69 cm^-1^

HRMS (EI+) m/z: [M]^+^ calcd 222.1251, found 222.1241.

**1-Methoxy-4-(1-methoxyethyl)benzene (11)** spectral data were in agreement with literature values^36^

To a mixture of 1-(4-methoxyphenyl)ethan-1-ol (609 mg, 4.0 mmol) in 10 mL THF was slowly added 60% NaH (208 mg, 5.2 mmol) at 0^o^C, and the mixture was stirred for 30min 0^o^C. Then CH_3_I (373 uL, 6.0 mmol) was added to the above mixture at 0^o^C, and the reaction was warmed up to room temperature and stirred overnight. The reaction was quenched with 2 mL H_2_O, extracted with EA (10 mL × 3), and the combined organic layers were washed with 20 mL sat. brine, dried over Na_2_SO_4_, filtrated and concentrated in vacuo. The residue was purified by silica gel chromatography with PE/EA (20:1) as eluting solvent to afford the 1-methoxy-4-(1-methoxyethyl)benzene **11** as colorless liquid (538 mg, 81%).

^1^H NMR (300 MHz, CDCl_3_) δ 7.23 (d, *J* = 8.6 Hz, 2H), 6.89 (d, *J* = 8.6 Hz, 2H), 4.25 (q, *J* = 6.4 Hz, 1H), 3.81 (s, 3H), 3.20 (s, 3H), 1.42 (d, *J* = 6.4 Hz, 3H). ^13^C NMR (75 MHz, CDCl_3_) δ 158.98, 135.50, 127.39, 113.76, 79.09, 56.17, 55.23, 23.74.

**1-(4-Methoxyphenyl)ethyl acetate (12)** was prepared according to a published procedure; spectral data were in agreement with literature values^37^

^1^H NMR (300 MHz, CDCl_3_) δ 7.30 (d, *J* = 8.7 Hz, 2H), 6.88 (d, *J* = 8.7 Hz, 2H), 5.85 (q, *J* = 6.6 Hz, 1H), 3.80 (s, 3H), 2.05 (s, 3H), 1.52 (d, *J* = 6.6 Hz, 3H). ^13^C NMR (75 MHz, CDCl_3_) δ 170.36, 159.26, 133.73, 127.58, 113.81, 71.98, 55.25, 21.92, 21.38.

**1-(1-Bromoethyl)-4-chlorobenzene (16)** spectral data were in agreement with literature values^38^

To a solution of 1-(4-chlorophenyl)ethan-1-ol (470 mg, 3 mmol) in 10 mL DCM was slowly added PBr_3_ (1.22 g, 4.5 mmol) at 0^o^C, then it’s warmed up to room temperature and stirred for 2 h. The reaction was quenched with 5 mL ice/water, extracted with DCM (10 mL × 3), and the combined organic layers were washed with 20 mL sat. NaHCO_3_ aq., 20 mL sat. brine, dried over Na_2_SO_4_, filtrated and concentrated in vacuo to afford 1-(1-bromoethyl)-4-chlorobenzene **16** as colorless liquid (618 mg, 94%).

^1^H NMR (300 MHz, CDCl_3_) δ 7.38 (d, *J* = 8.6 Hz, 2H), 7.31 (d, *J* = 8.6 Hz, 2H), 5.17 (q, *J* = 6.9 Hz, 1H), 2.03 (d, *J* = 6.9 Hz, 3H). ^13^C NMR (75 MHz, CDCl_3_) δ 141.75, 134.01, 128.84, 128.17, 48.18, 26.72.

**2.2 Substrate Scope for Secondary Anilines**

**General Procedure for the Synthesis of Secondary Anilines – GP5**

To a solution of the alcohol (0.2 mmol) in 1.0 mL HFIP was added aminating reagent (0.22 mmol) at room temperature under ambient atmosphere, unless otherwise stated. The reaction was stirred at room temperature for 12 h (monitored by GCMS or TLC). The reaction was diluted with 1 mL DCM and basified with 1 mL saturated NaHCO_3_ aqueous solution. The aqueous layer was extracted with DCM (3 mL × 3), and the combined organic layers were washed with 5 mL sat. brine, dried over anhydrous Na_2_SO_4_, filtrated and concentrated in vacuo. The crude residue was purified by silica gel chromatography with PE/EA to afford the desired product.

**4-Methoxy-*N*-methylaniline (3a)** spectral data were in agreement with literature values^1^

Following **GP5**, 1-(4-methoxyphenyl)ethan-1-ol (**1a**, 30 mg, 0.2 mmol), TsONHMe (**2a**, 44 mg, 0.22 mmol) in 1.0 mL HFIP solution were stirred for 12 h at room temperature. Purification by silica gel chromatography with PE/EA (10:1) as eluting solvent afforded 4-methoxy-*N*-methylaniline **3a** as yellow oil (20 mg, 74%).

Following **GP5**, 2-(4-methoxyphenyl)propan-2-ol (**1e**, 33 mg, 0.2 mmol), TsONHMe (**2a**, 44 mg, 0.22 mmol) in 1.0 mL HFIP solution were stirred for 12 h at room temperature. Purification by silica gel chromatography with PE/EA (10:1) as eluting solvent afforded 4-methoxy-*N*-methylaniline **3a** as yellow oil (25 mg, 96%).

Following **GP5**, 1-(4-methoxyphenyl)cyclohexan-1-ol (**1f**, 41 mg, 0.2 mmol), TsONHMe (**2a**, 52 mg, 0.26 mmol) in 1.0 mL HFIP solution were stirred for 12 h at room temperature. Purification by silica gel chromatography with PE/EA (10:1) as eluting solvent afforded 4-methoxy-*N*-methylaniline **3a** as yellow oil (26 mg, 96%).

^1^H NMR (300 MHz, CDCl_3_) δ 6.81 (d, *J* = 8.9 Hz, 2H), 6.60 (d, *J* = 8.9 Hz, 2H), 3.76 (s, 3H), 3.31 (br, 1H), 2.81 (s, 3H). ^13^C NMR (75 MHz, CDCl_3_) δ 152.06, 143.68, 114.89, 113.61, 55.83, 55.81, 31.57.

***N*-Methylaniline (3b)** spectral data were in agreement with literature values^39^

Following **GP5**, 1-phenylethan-1-ol (**1b**, 24 mg, 0.2 mmol), TsONHMe (**2a**, 44 mg, 0.22 mmol) in 1.0 mL HFIP solution were stirred for 12 h at room temperature. Purification by silica gel chromatography with PE/EA (10:1) as eluting solvent afforded *N*-methylaniline **3b** as yellow oil (10 mg, 48%).

^1^H NMR (300 MHz, CDCl_3_) δ 7.27 – 7.14 (m, 2H), 6.78 – 6.66 (m, 1H), 6.63 (d, *J* = 8.7 Hz, 1H), 3.73 (br, 1H), 2.85 (s, 3H). ^13^C NMR (75 MHz, CDCl_3_) δ 149.32, 129.18, 117.24, 112.40, 30.71.

***N*,2-Dimethylaniline (3c)** spectral data were in agreement with literature values^39^

Following **GP5**, 1-(*o*-tolyl)ethan-1-ol (**1c**, 27 mg, 0.2 mmol), TsONHMe (**2a**, 44 mg, 0.22 mmol) in 1.0 mL HFIP solution were stirred for 12 h at room temperature. Purification by silica gel chromatography with PE/EA (20:1) as eluting solvent afforded *N*,2-dimethylaniline **3c** as yellow oil (13 mg, 54%).

^1^H NMR (400 MHz, CDCl_3_) δ 7.17 (td, *J* = 7.8, 1.4 Hz, 1H), 7.06 (d, *J* = 7.4 Hz, 1H), 6.68 (td, *J* = 7.4, 1.4 Hz, 1H), 6.63 (d, *J* = 7.8 Hz, 1H), 2.91 (s, 3H), 2.14 (s, 3H). ^13^C NMR (100 MHz, CDCl_3_) δ 147.22, 129.89, 127.17, 121.88, 116.85, 109.13, 30.75, 17.34.

**4-(*tert*-Butyl)-*N*-methylaniline (3d)** spectral data were in agreement with literature values^1^

Following **GP5**, 1-(4-(*tert*-butyl)phenyl)ethan-1-ol (**1d**, 36 mg, 0.2 mmol), TsONHMe (**2a**, 44 mg, 0.22 mmol) in 1.0 mL HFIP solution were stirred for 12 h at room temperature. Purification by silica gel chromatography with PE/EA (20:1) as eluting solvent afforded 4-(*tert*-butyl)-*N*-methylaniline **3d** as yellow oil (27 mg, 84%).

^1^H NMR (400 MHz, CDCl_3_) δ 7.24 (d, *J* = 8.7 Hz, 2H), 6.60 (d, *J* = 8.7 Hz, 2H), 3.60 (br, 1H), 2.84 (s, 3H), 1.30 (s, 9H). ^13^C NMR (100 MHz, CDCl_3_) δ 147.03, 140.06, 125.95, 112.20, 33.81, 31.54, 30.95.

**4-(Methylamino)phenol (3g)** spectral data were in agreement with literature values^40^

Following **GP5**, 4-(1-hydroxyethyl)phenol (**1g**, 28 mg, 0.2 mmol), MsONHMe (**2b**, 28 mg, 0.22 mmol) in 1.0 mL HFIP solution were stirred for 12 h at room temperature. Purification by silica gel chromatography with PE/EA (3:1) as eluting solvent afforded 4-(methylamino)phenol **3g** as yellow solid (16 mg, 59%).

^1^H NMR (300 MHz, CDCl_3_) δ 6.70 (d, *J* = 8.8 Hz, 2H), 6.55 (d, *J* = 8.8 Hz, 2H), 4.08 (br, 2H), 2.80 (s, 3H). ^13^C NMR (75 MHz, CDCl_3_) δ 147.88, 143.39, 116.18, 114.11, 31.78.

**4-Chloro-*N*-methylaniline (3h)** spectral data were in agreement with literature values^39^

Following **GP5**, 1-(4-chlorophenyl)ethan-1-ol (**1h**, 31 mg, 0.2 mmol), TsONHMe (44 mg, 0.22 mmol) in 1.0 mL HFIP solution were stirred for 12 h at room temperature. Purification by silica gel chromatography with PE/EA (10:1) as eluting solvent afforded 4-chloro-*N*-methylaniline **3h** as yellow oil (24 mg, 85%).

^1^H NMR (300 MHz, CDCl_3_) δ 7.14 (d, *J* = 8.8 Hz, 2H), 6.57 (d, *J* = 8.8 Hz, 2H), 4.02 (br, 1H), 2.82 (s, 3H). ^13^C NMR (75 MHz, CDCl_3_) δ 147.29, 129.04, 122.40, 113.88, 31.10.

**4-(Methylamino)phenyl 4-methylbenzenesulfonate (3i)**

Following **GP5**, 4-(1-hydroxyethyl)phenyl 4-methylbenzenesulfonate (**1i**, 58 mg, 0.2 mmol), TsONHMe (**2a**, 44 mg, 0.22 mmol) in 1.0 mL HFIP solution were stirred for 12 h at room temperature. Purification by silica gel chromatography with PE/EA (10:1) as eluting solvent afforded 4-(methylamino)phenyl 4-methylbenzenesulfonate **3i** as yellow solid (31 mg, 56%).

^1^H NMR (300 MHz, CDCl_3_) δ 7.68 (d, *J* = 8.2 Hz, 2H), 7.29 (d, *J* = 8.2 Hz, 2H), 6.76 (d, *J* = 9.0 Hz, 2H), 6.43 (d, *J* = 9.0 Hz, 2H), 2.78 (s, 3H), 2.44 (s, 3H). ^13^C NMR (75 MHz, CDCl_3_) δ 148.00, 144.95, 140.73, 132.55, 129.57, 128.57, 123.10, 112.39, 30.76, 21.66.

Mp: 88^o^C

IR (EXTRACT): ṽ = 3417.69, 2980.75, 2928.84, 2891.13, 2816.28, 1918.00, 1873.06, 1733.02, 1598.57, 1514.78, 1447.15, 1432.45, 1402.46, 1339.36, 1295.28, 1267.66, 1194.65, 1174.66, 1145.70, 1117.86, 1091.37, 1061.96, 1004.16, 852.41, 826.57, 807.28, 727.94, 705.43, 679.75, 633.95 cm^-1^

HRMS (EI+) m/z: [M]^+^ calcd 277.0767, found 277.0759.

**3-Bromo-*N*-methylaniline (3j)** spectral data were in agreement with literature values^41^

Following **GP5**, 1-(3-bromophenyl)ethan-1-ol (**1j**, 40 mg, 0.2 mmol), TsONHMe (**2a**, 44 mg, 0.22 mmol) in 1.0 mL HFIP solution were stirred for 12 h at room temperature. Purification by silica gel chromatography with PE/EA (10:1) as eluting solvent afforded 3-bromo-*N*-methylaniline **3j** as yellow oil (8 mg, 22%).

^1^H NMR (300 MHz, CDCl_3_) δ 7.02 (t, *J* = 8.1 Hz, 1H), 6.81 (d, *J* = 8.0 Hz, 1H), 6.73 (t, *J* = 2.1 Hz, 1H), 6.51 (dd, *J* = 8.1, 2.1 Hz, 1H), 2.81 (s, 3H). ^13^C NMR (75 MHz, CDCl_3_) δ 150.52, 130.38, 123.28, 119.90, 114.76, 111.22, 30.49.

**4-Methoxy-3-(methylamino)benzonitrile (3k)**

Following **GP5**, 3-(1-hydroxyethyl)-4-methoxybenzonitrile (**1k**, 35 mg, 0.2 mmol), TsONHMe (**2a**, 89 mg, 0.44 mmol) in 0.5 mL HFIP solution were stirred for 12 h at room temperature. Purification by silica gel chromatography with PE/EA (5:1) as eluting solvent afforded 4-methoxy-3-(methylamino)benzonitrile **3k** as white solid (19 mg, 59%).

^1^H NMR (600 MHz, CDCl_3_) δ 6.99 (dd, *J* = 8.2, 2.0 Hz, 1H), 6.73 (d, *J* = 8.2 Hz, 1H), 6.71 (d, *J* = 2.0 Hz, 1H), 4.43 (br, 1H), 3.88 (s, 3H), 2.85 (s, 3H). ^13^C NMR (150 MHz, CDCl_3_) δ 149.90, 139.64, 121.53, 120.22, 110.82, 108.78, 104.31, 55.57, 29.88.

Mp: 68^o^C

IR (EXTRACT): ṽ = 3422.41, 3026.82, 2996.21, 2915.97, 2847.20, 2798.57, 2220.87, 1819.25, 1592.66, 1521.00, 1479.62, 1458.53, 1445.40, 1431.88, 1407.66, 1350.01, 1290.54, 1266.49, 1233.89, 1164.24, 1121.96, 1019.51, 936.31, 837.27, 799.49, 768.86, 684.28, 622.08 cm^-1^

HRMS (EI+) m/z: [M]^+^ calcd 162.0788, found 162.0773.

**2-methoxy-*N*-methyl-5-nitroaniline (3l)** spectral data were in agreement with literature values^39^

Following **GP5**, 1-(2-methoxy-5-nitrophenyl)ethan-1-ol (**1l**, 39 mg, 0.2 mmol), TsONHMe (**2a**, 89 mg, 0.44 mmol) in 0.5 mL HFIP solution were stirred for 12 h at room temperature. Purification by silica gel chromatography with PE/EA (5:1) as eluting solvent afforded 2-methoxy-*N*-methyl-5-nitroaniline **3l** as yellow solid (28 mg, 78%).

^1^H NMR (300 MHz, CDCl_3_) δ 7.62 (dd, *J* = 8.8, 2.7 Hz, 1H), 7.35 (d, *J* = 2.7 Hz, 1H), 6.74 (d, *J* = 8.8 Hz, 1H), 4.47 (br, 1H), 3.93 (s, 3H), 2.92 (s, 3H). ^13^C NMR (75 MHz, CDCl_3_) δ 151.56, 142.54, 139.48, 113.01, 107.66, 103.11, 55.96, 30.00.

**1-(3-(Methylamino)phenyl)ethan-1-ol (3m)**

Following **GP5**, 1,1'-(1,3-phenylene)bis(ethan-1-ol) (**1m**, 33 mg, 0.2 mmol), TsONHMe (**2a**, 44 mg, 0.22 mmol) in 1.0 mL HFIP solution were stirred for 12 h at room temperature. Purification by silica gel chromatography with PE/EA (2:1) as eluting solvent afforded 1-(3-(methylamino)phenyl)ethan-1-ol **3m** as yellow solid (8 mg, 27%).

Following **GP5**, 1,1'-(1,3-phenylene)bis(ethan-1-ol) (**1m**, 33 mg, 0.2 mmol), TsONHMe (**2a**, 89 mg, 0.44 mmol) in 0.5 mL HFIP solution were stirred for 12 h at room temperature. Purification by silica gel chromatography with PE/EA (2:1) as eluting solvent afforded 1-(3-(methylamino)phenyl)ethan-1-ol **3m** as yellow solid (14 mg, 47%).

^1^H NMR (300 MHz, CDCl_3_) δ 7.17 (t, *J* = 7.8 Hz, 1H), 6.70 (d, *J* = 7.8 Hz, 1H), 6.64 (t, *J* = 1.9 Hz, 1H), 6.53 (dd, *J* = 7.8, 1.9 Hz, 1H), 4.82 (q, *J* = 6.4 Hz, 1H), 2.85 (s, 3H), 2.62 (br, 2H), 1.48 (d, *J* = 6.4 Hz, 3H). ^13^C NMR (75 MHz, CDCl_3_) δ 149.53, 147.08, 129.34, 114.31, 111.56, 109.27, 70.63, 30.75, 24.99.

***N*,2,4-Trimethylaniline (3n)** spectral data were in agreement with literature values^1^

Following **GP5**, 1-(2,4-dimethylphenyl)ethan-1-ol (**1n**, 30 mg, 0.2 mmol), TsONHMe (**2a**, 44 mg, 0.22 mmol) in 1.0 mL HFIP solution were stirred for 12 h at room temperature. Purification by silica gel chromatography with PE/EA (20:1) as eluting solvent afforded *N*,2,4-trimethylaniline **3n** as yellow oil (20 mg, 74%).

^1^H NMR (300 MHz, CDCl_3_) δ 6.98 (dd, *J* = 8.0, 2.1 Hz, 1H), 6.91 (d, *J* = 2.1 Hz, 1H), 6.57 (d, *J* = 8.0 Hz, 1H), 3.51 (br, 1H), 2.90 (s, 3H), 2.26 (s, 3H), 2.14 (s, 3H). ^13^C NMR (75 MHz, CDCl_3_) δ 144.77, 130.83, 127.35, 126.24, 122.22, 109.60, 31.16, 20.30, 17.30.

***N*,2,4,6-Tetramethylaniline (3o)** spectral data were in agreement with literature values^1^

Following **GP5**, 1-mesitylethan-1-ol (**1o**, 33 mg, 0.2 mmol), TsONHMe (**2a**, 44 mg, 0.22 mmol) in 1.0 mL HFIP solution were stirred for 12 h at room temperature. Purification by silica gel chromatography with PE/EA (10:1) as eluting solvent afforded *N*,2,4,6-tetramethylaniline **3o** as yellow oil (20 mg, 67%).

^1^H NMR (300 MHz, CDCl_3_) δ 6.84 (s, 2H), 2.82 (br, 1H), 2.75 (s, 3H), 2.28 (s, 6H), 2.24 (s, 3H). ^13^C NMR (75 MHz, CDCl_3_) δ 144.80, 131.31, 129.50, 129.42, 35.56, 20.50, 18.11.

***N*-Methyl-[1,1'-biphenyl]-4-amine (3p)** spectral data were in agreement with literature values^42^

Following **GP5**, 1-([1,1'-biphenyl]-4-yl)ethan-1-ol (**1p**, 40 mg, 0.2 mmol), TsONHMe (**2a**, 44 mg, 0.22 mmol) in 1.0 mL HFIP solution were stirred for 12 h at room temperature. Purification by silica gel chromatography with PE/EA (10:1) as eluting solvent afforded *N*-methyl-[1,1'-biphenyl]-4-amine **3p** as yellow oil (29 mg, 80%).

^1^H NMR (500 MHz, CDCl_3_) δ 7.57 (d, *J* = 7.6 Hz, 2H), 7.48 (d, *J* = 7.9 Hz, 2H), 7.41 (t, *J* = 7.6 Hz, 2H), 7.28 (t, *J* = 7.9 Hz, 1H), 6.70 (d, *J* = 7.9 Hz, 2H), 3.81 (br, 1H), 2.89 (s, 3H). ^13^C NMR (125 MHz, CDCl_3_) δ 148.71, 141.27, 130.09, 128.61, 127.87, 126.26, 125.98, 112.61, 30.73.

***N*-Methyl-4-phenoxyaniline (3q)** spectral data were in agreement with literature values^1^

Following **GP5**, 1-(4-phenoxyphenyl)ethan-1-ol (**1q**, 43 mg, 0.2 mmol), TsONHMe (**2a**, 52 mg, 0.26 mmol) in 1.0 mL HFIP solution were stirred for 12 h at room temperature. Purification by silica gel chromatography with PE/EA (10:1) as eluting solvent afforded *N*-methyl-4-phenoxyaniline **3q** as yellow oil (23 mg, 58%).

^1^H NMR (300 MHz, CDCl_3_) δ 7.32 – 7.26 (m, 2H), 7.04 – 6.96 (m, 1H), 6.93 (d, *J* = 8.8 Hz, 4H), 6.61 (d, *J* = 8.8 Hz, 2H), 3.60 (br, 1H), 2.85 (s, 3H). ^13^C NMR (75 MHz, CDCl_3_) δ 159.15, 147.52, 146.00, 129.46, 121.85, 121.24, 117.01, 113.31, 31.20.

***N*-Methylnaphthalen-2-amine (3r)** spectral data were in agreement with literature values^1^

Following **GP5**, 1-(naphthalen-2-yl)ethan-1-ol (**1r**, 35 mg, 0.2 mmol), TsONHMe (**2a**, 44 mg, 0.22 mmol) in 1.0 mL HFIP solution were stirred for 12 h at room temperature. Purification by silica gel chromatography with PE/EA (10:1) as eluting solvent afforded *N*-methylnaphthalen-2-amine **3r** as yellow solid (24 mg, 77%).

^1^H NMR (300 MHz, CDCl_3_) δ 7.74 – 7.59 (m, 3H), 7.38 (ddd, *J* = 8.2, 6.8, 1.3 Hz, 1H), 7.21 (ddd, *J* = 8.2, 6.8, 1.3 Hz, 1H), 6.89 (dd, *J* = 8.8, 2.4 Hz, 1H), 6.81 (d, *J* = 2.4 Hz, 1H), 3.73 (br, 1H), 2.95 (s, 3H). ^13^C NMR (75 MHz, CDCl_3_) δ 146.95, 135.26, 128.78, 127.61, 127.45, 126.27, 125.90, 121.86, 117.85, 103.73, 30.73.

**4-Bromo-*N*-methylaniline (3s) and 4-bromobenzaldehyde (3s’)**

Following **GP5**, bis(4-bromophenyl)methanol (**1s**, 68 mg, 0.2 mmol), TsONHMe (**2a**, 44 mg, 0.22 mmol) in 1.0 mL HFIP solution were stirred for 12 h at room temperature. Purification by silica gel chromatography with PE/EA (20:1) as eluting solvent afforded 4-bromo-*N*-methylaniline **3s** as yellow oil (32 mg, 86%) and 4-bromobenzaldehyde **3s’** as colorless solid (26 mg, 70%).

**4-Bromo-*N*-methylaniline (3s)** spectral data were in agreement with literature values^43^

^1^H NMR (300 MHz, CDCl_3_) δ 7.26 (d, *J* = 8.8 Hz, 2H), 6.48 (d, *J* = 8.8 Hz, 2H), 3.60 (br, 1H), 2.81 (s, 3H). ^13^C NMR (75 MHz, CDCl_3_) δ 148.24, 131.83, 113.90, 108.76, 30.67.

**4-Bromobenzaldehyde (3s’)** spectral data were in agreement with literature values^44^

^1^H NMR (300 MHz, CDCl_3_) δ 9.98 (s, 1H), 7.75 (d, *J* = 8.5 Hz, 2H), 7.68 (d, *J* = 8.5 Hz, 2H). ^13^C NMR (75 MHz, CDCl_3_) δ 191.03, 135.07, 132.43, 130.95, 129.77.

***N*-Methyldibenzo[*b,d*]furan-2-amine (3t)** spectral data were in agreement with literature values^1^

Following **GP5**, 1-(dibenzo[*b,d*]furan-2-yl)ethan-1-ol (**1t**, 42 mg, 0.2 mmol), TsONHMe (**2a**, 44 mg, 0.22 mmol) in 1.0 mL HFIP solution were stirred for 12 h at room temperature. Purification by silica gel chromatography with PE/EA (10:1) as eluting solvent afforded *N*-methyldibenzo[*b,d*]furan-2-amine **3t** as yellow solid (29 mg, 74%).

^1^H NMR (300 MHz, CDCl_3_) δ 7.90 (d, *J* = 7.6 Hz, 1H), 7.52 (d, *J* = 7.6 Hz, 1H), 7.42 (td, *J* = 7.6, 1.2 Hz, 1H), 7.39 (d, *J* = 8.7 Hz, 1H), 7.30 (td, *J* = 7.6, 1.2 Hz, 1H), 7.13 (d, *J* = 2.5 Hz, 1H), 6.78 (dd, *J* = 8.7, 2.5 Hz, 1H), 3.47 (br, 1H), 2.94 (s, 3H). ^13^C NMR (75 MHz, CDCl_3_) δ 156.68, 149.73, 145.64, 126.70, 124.77, 124.58, 122.10, 120.46, 114.13, 111.89, 111.56, 102.06, 31.68.

***N*-Methyl-9-tosyl-9*H*-carbazol-3-amine (3u)** spectral data were in agreement with literature values^1^

Following **GP5**, 1-(9-tosyl-9*H*-carbazol-3-yl)ethan-1-ol (**1u**, 73 mg, 0.2 mmol), TsONHMe (**2a**, 44 mg, 0.22 mmol) in 1.0 mL HFIP solution were stirred for 12 h at room temperature. Purification by silica gel chromatography with PE/EA (2:1) as eluting solvent afforded *N*-methyl-9-tosyl-9*H*-carbazol-3-amine **3u** as yellow solid (58 mg, 83%).

^1^H NMR (300 MHz, CDCl_3_) δ 8.28 (d, *J* = 8.4 Hz, 1H), 8.11 (d, *J* = 8.9 Hz, 1H), 7.80 (d, *J* = 7.6 Hz, 1H), 7.61 (d, *J* = 8.2 Hz, 2H), 7.43 (ddd, *J* = 8.4, 7.6, 1.2 Hz, 1H), 7.30 (td, *J* = 7.6, 1.2 Hz, 1H), 7.04 (d, *J* = 8.2 Hz, 2H), 7.00 (d, *J* = 2.4 Hz, 1H), 6.78 (dd, *J* = 8.9, 2.4 Hz, 1H), 2.91 (s, 3H), 2.23 (s, 3H). ^13^C NMR (75 MHz, CDCl_3_) δ 146.62, 144.43, 139.01, 134.73, 130.94, 129.43, 127.77, 127.01, 126.98, 126.43, 123.65, 119.80, 116.26, 115.51, 114.30, 101.18, 31.21, 21.43.

**5-(3,3-Dimethylbut-1-yn-1-yl)-*N*-methylbenzo[*d*][1,3]dioxol-4-amine (3v)**

Following **GP5**, 1-(5-(3,3-dimethylbut-1-yn-1-yl)benzo[*d*][1,3]dioxol-4-yl)ethan-1-ol (**1v**, 49 mg, 0.2 mmol), TsONHMe (**2a**, 44 mg, 0.22 mmol) in 1.0 mL HFIP solution were stirred for 12 h at room temperature. Purification by silica gel chromatography with PE/EA (10:1) as eluting solvent afforded 5-(3,3-dimethylbut-1-yn-1-yl)-*N*-methylbenzo[*d*][1,3]dioxol-4-amine **3v** as yellow oil (14 mg, 30%).

Following **GP5**, 1-(5-(3,3-dimethylbut-1-yn-1-yl)benzo[*d*][1,3]dioxol-4-yl)ethan-1-ol (**1v**, 49 mg, 0.2 mmol), TsONHMe (**2a**, 88 mg, 0.44 mmol) in 0.5 mL HFIP solution were stirred for 12 h at room temperature. Purification by silica gel chromatography with PE/EA (10:1) as eluting solvent afforded 5-(3,3-dimethylbut-1-yn-1-yl)-*N*-methylbenzo[*d*][1,3]dioxol-4-amine **3v** as yellow oil (19 mg, 41%).

^1^H NMR (300 MHz, CDCl_3_) δ 6.81 (d, *J* = 8.0 Hz, 1H), 6.22 (d, *J* = 8.0 Hz, 1H), 5.86 (s, 2H), 4.13 (br, 1H), 3.09 (s, 3H), 1.32 (s, 9H). ^13^C NMR (75 MHz, CDCl_3_) δ 148.36, 135.03, 133.34, 126.14, 104.88, 102.54, 100.26, 98.75, 75.19, 32.67, 31.26, 28.23.

IR (EXTRACT): ṽ = 3397.69, 2967.84, 2926.91, 2897.97, 2867.42, 2817.03, 2774.18, 1733.52, 1631.59, 1600.89, 1511.74, 1478.85, 1435.66, 1392.62, 1361.76, 1329.25, 1291.98, 1267.19, 1242.04, 1221.32, 1171.84, 1128.64, 1092.82, 1052.82, 994.17, 937.04, 868.52, 784.30, 736.79, 688.25, 652.88, 624.22 cm^-1^

HRMS (EI+) m/z: [M]^+^ calcd 231.1254, found 231.1236.

***N*-Methyl-2,3-dihydrobenzofuran-5-amine (3w)** spectral data were in agreement with literature values^1^

Following **GP5**, 1-(2,3-dihydrobenzofuran-5-yl)ethan-1-ol (**1w**, 33 mg, 0.2 mmol), TsONHMe (**2a**, 44 mg, 0.22 mmol) in 1.0 mL HFIP solution were stirred for 12 h at room temperature. Purification by silica gel chromatography with PE/EA (10:1) as eluting solvent afforded *N*-methyl-2,3-dihydrobenzofuran-5-amine **3w** as yellow oil (19 mg, 64%).

^1^H NMR (300 MHz, CDCl_3_) δ 6.66 (d, *J* = 8.4 Hz, 1H), 6.56 (d, *J* = 2.5 Hz, 1H), 6.41 (dd, *J* = 8.4, 2.5 Hz, 1H), 4.50 (t, *J* = 8.5 Hz, 2H), 3.15 (t, *J* = 8.5 Hz, 2H), 2.80 (s, 3H). ^13^C NMR (75 MHz, CDCl_3_) δ 152.45, 143.84, 127.71, 111.96, 109.96, 109.30, 70.83, 31.91, 30.36.

***N*-Methyl-1-tosylindolin-5-amine (3x)** spectral data were in agreement with literature values^1^

Following **GP5**, 1-(1-tosylindolin-5-yl)ethan-1-ol (**1x**, 64 mg, 0.2 mmol), TsONHMe (**2a**, 44 mg, 0.22 mmol) in 1.0 mL HFIP solution were stirred for 12 h at room temperature. Purification by silica gel chromatography with PE/EA (2:1) as eluting solvent afforded *N*-methyl-1-tosylindolin-5-amine **3x** as yellow solid (49 mg, 82%).

^1^H NMR (300 MHz, CDCl_3_) δ 7.57 (d, *J* = 8.2 Hz, 2H), 7.48 (d, *J* = 8.6 Hz, 1H), 7.18 (d, *J* = 8.2 Hz, 2H), 6.46 (dd, *J* = 8.6, 2.6 Hz, 1H), 6.33 (d, *J* = 2.6 Hz, 1H), 3.86 (t, *J* = 8.2 Hz, 2H), 3.60 (br, 1H), 2.78 (s, 3H), 2.64 (t, *J* = 8.2 Hz, 2H), 2.35 (s, 3H). ^13^C NMR (75 MHz, CDCl_3_) δ 146.83, 143.59, 134.02, 133.99, 132.82, 129.44, 127.37, 117.35, 111.66, 108.75, 50.27, 31.11, 28.44, 21.48.

***N*,3,5,5,6,8,8-Heptamethyl-5,6,7,8-tetrahydronaphthalen-2-amine (3y)**

Following **GP5**, 1-(3,5,5,6,8,8-hexamethyl-5,6,7,8-tetrahydronaphthalen-2-yl)ethan-1-ol (**1y**, 52 mg, 0.2 mmol), TsONHMe (**2a**, 44 mg, 0.22 mmol) in 1.0 mL HFIP solution were stirred for 12 h at room temperature. Purification by silica gel chromatography with PE/EA (20:1) as eluting solvent afforded methyl *N*,3,5,5,6,8,8-heptamethyl-5,6,7,8-tetrahydronaphthalen-2-amine **3y** as yellow solid (39 mg, 80%).

^1^H NMR (300 MHz, CDCl_3_) δ 7.06 (s, 1H), 6.53 (s, 1H), 3.29 (br, 1H), 2.91 (s, 3H) ,2.13 (s, 3H), 1.94 – 1.81 (m, 1H), 1.66 (t, *J* = 13.1 Hz, 1H), 1.39 – 1.36 (m, 1H), 1.33 (s, 3H), 1.31 (s, 6H), 1.06 (s, 3H), 0.99 (d, *J* = 6.7 Hz, 3H). ^13^C NMR (75 MHz, CDCl_3_) δ 144.77, 143.28, 134.53, 128.48, 120.27, 106.56, 44.01, 36.83, 34.73, 34.32, 32.32, 32.19, 30.90, 28.82, 24.99, 17.20, 16.85.

Mp: 54^o^C

IR (EXTRACT): ṽ = 3420.82, 3023.66, 2962.92, 2930.04, 2906.60, 2806.59, 1734.64, 1618.78, 1571.82, 1527.33, 1512.74, 1469.80, 1433.17, 1396.26, 1360.13, 1340.96, 1325.36, 1298.38, 1267.67, 1241.50, 1174.41, 1140.82, 1113.79, 1080.38, 1031.90, 1004.83, 883.60, 849.74, 732.07, 671.78, 644.33 cm^-1^

HRMS (EI+) m/z: [M]^+^ calcd 245.2138, found 245.2113.

**3-(Methylamino)-2*H*-chromen-2-one (3z) and 3-(1-((1,1,1,3,3,3-hexafluoropropan-2-yl)oxy)ethyl)-2*H*-chromen-2-one (3z’)**

Following **GP5**, 3-(1-hydroxyethyl)-2*H*-chromen-2-one (**1z**, 38 mg, 0.2 mmol), TsONHMe (**2a**, 44 mg, 0.22 mmol) in 1.0 mL HFIP solution were stirred for 12 h at room temperature. Purification by silica gel chromatography with PE/EA (5:1) as eluting solvent afforded 3-(methylamino)-2*H*-chromen-2-one **3z** as yellow solid (4 mg, 11%) and 3-(1-((1,1,1,3,3,3-hexafluoropropan-2-yl)oxy)ethyl)-2*H*-chromen-2-one **3z’** as white solid (33 mg, 49%).

Following **GP5**, 3-(1-hydroxyethyl)-2*H*-chromen-2-one (**1z**, 38 mg, 0.2 mmol), TsONHMe (**2a**, 88 mg, 0.44 mmol) in 0.5 mL HFIP solution were stirred for 12 h at room temperature. Purification by silica gel chromatography with PE/EA (5:1) as eluting solvent afforded 3-(methylamino)-2*H*-chromen-2-one **3z** as yellow solid (17 mg, 49%) and 3-(1-((1,1,1,3,3,3-hexafluoropropan-2-yl)oxy)ethyl)-2*H*-chromen-2-one **3z’** as white solid (14 mg, 21%)

**3-(Methylamino)-2*H*-chromen-2-one (3z)**

^1^H NMR (300 MHz, CDCl_3_) δ 7.40 – 7.30 (m, 1H), 7.29 – 7.23 (m, 1H), 7.23 – 7.16 (m, 2H), 6.30 (s, 1H), 4.91 (br, 1H), 2.91 (d, *J* = 4.9 Hz, 3H). ^13^C NMR (75 MHz, CDCl_3_) δ 159.58, 147.86, 134.11, 125.56, 124.94, 124.60, 121.81, 116.04, 104.29, 29.75.

**3-(1-((1,1,1,3,3,3-Hexafluoropropan-2-yl)oxy)ethyl)-2*H*-chromen-2-one (3z’)**

^1^H NMR (400 MHz, CDCl_3_) δ 7.86 (s, 1H), 7.58 – 7.52 (m, 2H), 7.38 – 7.28 (m, 2H), 5.05 (q, *J* = 6.4 Hz, 1H), 4.40 (hept, *J* = 5.9 Hz, 1H), 1.59 (d, *J* = 6.4 Hz, 3H). ^13^C NMR (100 MHz, CDCl_3_) δ 160.54, 153.29, 138.88, 131.97, 128.61, 128.27, 124.74, 121.55 (qq, *J* = 282.6, 2.7 Hz), 121.22 (qq, *J* = 282.6, 2.7 Hz), 118.81, 116.61, 76.65, 75.30 (hept, *J* = 32.5 Hz), 20.58. ^19^F NMR (283 MHz, CDCl_3_) δ -73.80 (dm, *J* = 42.3 Hz).

Mp: 92^o^C

IR (EXTRACT): ṽ = 2993.76, 2932.75, 1691.86, 1634.80, 1607.92, 1575.31, 1453.10, 1410.03, 1387.45, 1375.41, 1293.72, 1262.54, 1226.71, 1181.39, 1141.94, 1116.94, 1103.11, 1033.70, 1002.66, 951.35, 932.42, 913.63, 891.61, 863.02, 846.28, 783.22, 754.76, 734.88, 692.92, 678.36, 610.98 cm^-1^

HRMS (EI+) m/z: [M]^+^ calcd 340.0529, found 340.0496.

**Methyl 5-(2,5-dimethyl-4-(methylamino)phenoxy)-2,2-dimethylpentanoate (3aa)** spectral data were in agreement with literature values^1^

Following **GP5**, methyl 5-(4-(1-hydroxyethyl)-2,5-dimethylphenoxy)-2,2-dimethylpentanoate (**1aa**, 61 mg, 0.2 mmol), TsONHMe (**2a**, 44 mg, 0.22 mmol) in 1.0 mL HFIP solution were stirred for 12 h at room temperature. Purification by silica gel chromatography with PE/EA (3:1) as eluting solvent afforded methyl 5-(2,5-dimethyl-4-(methylamino)phenoxy)-2,2-dimethylpentanoate **3aa** as brown oil (35 mg, 60%).

^1^H NMR (300 MHz, CDCl_3_) δ 6.61 (s, 1H), 6.44 (s, 1H), 3.95 – 3.80 (m, 2H), 3.67 (s, 3H), 2.99 (br, 1H), 2.86 (s, 3H), 2.22 (s, 3H), 2.10 (s, 3H), 1.72 – 1.68 (m, 4H), 1.22 (s, 6H). ^13^C NMR (75 MHz, CDCl_3_) δ 178.32, 148.92, 141.25, 125.30, 120.15, 115.73, 112.56, 69.61, 51.65, 42.06, 37.13, 31.48, 25.40, 25.13, 17.26, 16.03.

**Methyl (*S*)-2-(6-methoxy-5-(methylamino)naphthalen-2-yl)propanoate (3ab)** spectral data were in agreement with literature values^1^

Following **GP5**, methyl (2*S*)-2-(5-(1-hydroxyethyl)-6-methoxynaphthalen-2-yl)propanoate (**1ab**, 58 mg, 0.2 mmol), TsONHMe (**2a**, 44 mg, 0.22 mmol) in 1.0 mL HFIP solution were stirred for 12 h at room temperature. Purification by silica gel chromatography with PE/EA (3:1) as eluting solvent afforded methyl (*S*)-2-(6-methoxy-5-(methylamino)naphthalen-2-yl)propanoate **3ab** as brown oil (44 mg, 81%).

^1^H NMR (300 MHz, CDCl_3_) δ 8.05 (d, *J* = 8.9 Hz, 1H), 7.65 (d, *J* = 1.9 Hz, 1H), 7.46 (d, *J* = 8.9 Hz, 1H), 7.40 (dd, *J* = 8.9, 1.9 Hz, 1H), 7.24 (d, *J* = 8.9 Hz, 1H), 3.94 (s, 3H), 3.86 (q, *J* = 7.1 Hz, 1H), 3.67 (s, 3H), 3.60 (br, 1H), 2.97 (s, 3H), 1.58 (d, *J* = 7.1 Hz, 3H). ^13^C NMR (75 MHz, CDCl_3_) δ 175.11, 147.41, 135.55, 133.92, 129.94, 127.25, 126.40, 125.10, 123.38, 122.30, 113.83, 56.86, 52.00, 45.25, 37.10, 18.46.

**Methyl 2-methyl-2-(4-(methylamino)phenoxy)propanoate (3ac) and 4-chlorobenzaldehyde (3ac’)**

Following **GP5**, methyl 2-(4-((4-chlorophenyl)(hydroxy)methyl)phenoxy)-2-methylpropanoate (**1ac**, 67 mg, 0.2 mmol), TsONHMe (**2a**, 44 mg, 0.22 mmol) in 1 mL HFIP solution were stirred for 12 h at room temperature. Purification by silica gel chromatography with PE/EA (10:1) as eluting solvent afforded methyl 2-methyl-2-(4-(methylamino)phenoxy)propanoate **3ac** as yellow oil (30 mg, 68%) and 4-chlorobenzaldehyde **3ac’** as white solid (15 mg, 54%)

**Methyl 2-methyl-2-(4-(methylamino)phenoxy)propanoate (3ac)**

^1^H NMR (300 MHz, CDCl_3_) δ 6.77 (d, *J* = 8.9 Hz, 2H), 6.49 (d, *J* = 8.9 Hz, 2H), 3.77 (s, 3H), 2.79 (s, 3H), 1.51 (s, 6H). ^13^C NMR (75 MHz, CDCl_3_) δ 174.94, 146.50, 145.40, 122.27, 112.78, 79.78, 52.25, 31.17, 25.20.

IR (EXTRACT): ṽ = 3409.11, 2989.74, 2949.52, 2882.14, 2810.77, 1735.25, 1612.61, 1512.88, 1465.85, 1434.38, 1381.95, 1364.61, 1284.40, 1224.36, 1191.66, 1170.62, 1136.29, 1062.18, 1008.85, 949.63, 884.76, 827.13, 770.26, 748.92, 643.54 cm^-1^

HRMS (EI+) m/z: [M]^+^ calcd 223.1203, found 223.1196

**4-Chlorobenzaldehyde (3ac’)** spectral data were in agreement with literature values^45^

^1^H NMR (300 MHz, CDCl_3_) δ 9.98 (s, 1H), 7.82 (d, *J* = 8.5 Hz, 2H), 7.51 (d, *J* = 8.5 Hz, 2H). ^13^C NMR (75 MHz, CDCl_3_) δ 190.83, 140.95, 134.71, 130.89, 129.44.

**3,4-Dimethoxy-*N*-methylaniline (3ad)** spectral data were in agreement with literature values^1^

Following **GP5**, 1-(3,4-dimethoxyphenyl)-2-(2-methoxyphenoxy)propane-1,3-diol (**1ad**, 67 mg, 0.2 mmol), TsONHMe (**2a**, 44 mg, 0.22 mmol) in 1 mL HFIP solution were stirred for 12 h at room temperature. Purification by silica gel chromatography with PE/EA (10:1) as eluting solvent afforded 3,4-dimethoxy-*N*-methylaniline **3ad** as yellow oil (17 mg, 52%).

^1^H NMR (300 MHz, CDCl_3_) δ 6.76 (d, *J* = 8.5 Hz, 1H), 6.24 (d, *J* = 2.6 Hz, 1H), 6.15 (dd, *J* = 8.5, 2.6 Hz, 1H), 3.84 (s, 3H), 3.80 (s, 3H), 2.81 (s, 3H). ^13^C NMR (75 MHz, CDCl_3_) δ 150.01, 144.34, 141.48, 113.33, 102.99, 98.53, 56.73, 55.67, 31.44.

**(8*R*,9*S*,13*S*,14*S*)-3-(1-Hydroxyethyl)-13-methyl-7,8,9,11,12,13,14,15,16,17-decahydro-6*H*-cyclopenta[*a*]phenanthren-17-ol (3ae)**

Following **GP5**, (8*R*,9*S*,13*S*,14*S*)-13-methyl-3-(methylamino)-7,8,9,11,12,13,14,15,16,17-decahydro-6*H*-cyclopenta[*a*]phenanthren-17-ol (**1ae**, 60 mg, 0.2 mmol), TsONHMe (**2a**, 44 mg, 0.22 mmol) in 1 mL HFIP solution were stirred for 12 h at room temperature. Purification by silica gel chromatography with PE/EA (3:1) as eluting solvent afforded (8*R*,9*S*,13*S*,14*S*)-3-(1-hydroxyethyl)-13-methyl-7,8,9,11,12,13,14,15,16,17-decahydro-6*H*-cyclopenta[*a*]phenanthren-17-ol **3ae** as white solid (34 mg, 60%).

^1^H NMR (400 MHz, CDCl_3_) δ 7.13 (d, *J* = 8.4, 1H), 6.47 (dd, *J* = 8.4, 2.6 Hz, 1H), 6.37 (d, *J* = 2.6 Hz, 1H), 3.73 (t, *J* = 8.4 Hz, 1H), 2.91 – 2.74 (m, 2H), 2.82 (s, 3H), 2.33 – 2.26(m, 1H), 2.22 – 2.07 (m, 2H), 1.99 – 1.81 (m, 2H), 1.74 – 1.65 (m, 1H), 1.57 – 1.13 (m, 7H), 0.78 (s, 3H). ^13^C NMR (100 MHz, CDCl_3_) δ 147.25, 137.46, 129.49, 126.08, 112.56, 110.65, 81.94, 50.03, 43.93, 43.28, 39.06, 36.73, 30.98, 30.58, 29.82, 27.37, 26.34, 23.10, 11.04.

Mp: 162^o^C

IR (EXTRACT): ṽ = 3401.43, 3285.92, 2917.03, 2862.89, 2239.99, 1734.63, 1664.84, 1611.43, 1500.77, 1469.52, 1446.43, 1431.77, 1381.48, 1352.27, 1332.17, 1304.77, 1289.12, 1263.39, 1203.61, 1169.58, 1135.81, 1075.51, 1057.02, 1029.35, 1009.82, 980.35, 961.53, 949.20, 908.49, 869.00, 812.24, 776.68, 730.61, 710.21, 648.24, 610.08 cm^-1^

HRMS (EI+) m/z: [M]^+^ calcd 285.2087, found 285.2080.

**Special examples**

**4-Methoxy-*N*-methylaniline (3a)**

Following **GP5**, 1-(1-cyclopropylvinyl)-4-methoxybenzene (**1af**, 35 mg, 0.2 mmol), TsONHMe (**2a**, 81 mg, 0.4 mmol) in 0.5 mL HFIP solution were stirred for 12 h at room temperature. Purification by silica gel chromatography with PE/EA (10:1) as eluting solvent afforded 4-methoxy-*N*-methylaniline **3a** as yellow oil (15 mg, 56%).

**1-(4-Methoxy-3-(methylamino)phenyl)propan-2-ol (3ag)**

Following **GP5**, 1-(4-methoxyphenyl)propan-2-ol (**1ag**, 33 mg, 0.2 mmol), TsONHMe (**2a**, 80 mg, 0.4 mmol) in 1 mL HFIP solution were stirred for 36 h at room temperature. Purification by silica gel chromatography with PE/EA (2:1) as eluting solvent afforded 1-(4-methoxy-3-(methylamino)phenyl)propan-2-ol **3ag** as yellow oil (23 mg, 59%).

^1^H NMR (300 MHz, CDCl_3_) δ 6.70 (d, *J* = .0 Hz, 1H), 6.50 (dd, *J* = 8.0, 2.0 Hz, 1H), 6.43 (d, *J* = 2.0 Hz, 1H), 4.17 – 3.89 (m, 1H), 3.82 (s, 3H), 2.86 (s, 3H), 2.74 (dd, *J* = 13.5, 4.5 Hz, 1H), 2.56 (dd, *J* = 13.5, 8.4 Hz, 1H), 1.25 (d, *J* = 6.2 Hz, 3H). ^13^C NMR (75 MHz, CDCl_3_) δ 145.68, 139.40, 131.20, 116.70, 110.25, 109.25, 68.86, 55.46, 45.64, 30.30, 22.58.

IR (EXTRACT): ṽ = 3397.69, 2967.84, 2926.91, 2897.97, 2867.42, 2817.03, 2774.18, 1733.52, 1631.59, 1600.89, 1511.74, 1478.85, 1435.66, 1392.62, 1361.76, 1329.25, 1291.98, 1267.19, 1242.04, 1221.32, 1171.84, 1128.64, 1092.82, 1052.82, 994.17, 937.04, 868.52, 784.30, 736.79, 688.25, 652.88, 624.22 cm^-1^

HRMS (EI+) m/z: [M]^+^ calcd 195.1254, found 195.1259.

**2.3 Hydroxylamine Substrate for Secondary Anilines**

**4-Methoxy-*N*-methylaniline (3a)**

Following **GP5**, 1-(4-methoxyphenyl)ethan-1-ol (**1a**, 30 mg, 0.2 mmol), *N*-methyl-*O*-(methylsulfonyl)hydroxylamine (**2b**, 28 mg, 0.22 mmol) in 1.0 mL HFIP solution were stirred for 12 h at room temperature. Purification by silica gel chromatography with PE/EA (10:1) as eluting solvent afforded 4-methoxy-*N*-methylaniline **3a** as yellow oil (20 mg, 74%).

***N*-Ethyl-4-methoxyaniline (4c)** spectral data were in agreement with literature values^46^

Following **GP3**, *tert*-butyl ethyl(tosyloxy)carbamate (95 mg, 0.3 mmol), TFA (0.45 mL, 6 mmol) in 0.5 mL DCM solution were to give crude *N*-ethyl-*O*-tosylhydroxylamine **2c** which was directly used for next step without further purification.

Following **GP5**, 1-(4-methoxyphenyl)ethan-1-ol (**1a**, 30 mg, 0.2 mmol), crude *N*-ethyl-*O*-tosylhydroxylamine (**2c**, 0.3 mmol) in 1.0 mL HFIP solution were stirred for 12 h at room temperature. Purification by silica gel chromatography with PE/EA (10:1) as eluting solvent afforded *N*-ethyl-4-methoxyaniline **4c** as yellow oil (52 mg, 91%).

^1^H NMR (300 MHz, CDCl_3_) δ 6.79 (d, *J* = 8.9 Hz, 2H), 6.59 (d, *J* = 8.9 Hz, 2H), 3.75 (s, 3H), 3.22 (br, 1H), 3.12 (q, *J* = 7.1 Hz, 2H), 1.25 (t, *J* = 7.1 Hz, 3H). ^13^C NMR (75 MHz, CDCl_3_) δ 152.05, 142.71, 114.86, 114.10, 55.79, 39.43, 14.95.

***N*-Butyl-4-methoxyaniline (4d)** spectral data were in agreement with literature values^47^

Following **GP3**, *tert*-butyl butyl(tosyloxy)carbamate (103 mg, 0.3 mmol), TFA (0.45 mL, 6 mmol) in 0.5 mL DCM solution were to give crude *N*-butyl-*O*-tosylhydroxylamine **2d**, which was directly used for next step without further purification.

Following **GP5**, 1-(4-methoxyphenyl)ethan-1-ol (**1a**, 30 mg, 0.2 mmol), crude *N*-butyl-*O*-tosylhydroxylamine (**2d**, 0.3 mmol) in 1.0 mL HFIP solution were stirred for 12 h at room temperature. Purification by silica gel chromatography with PE/EA (10:1) as eluting solvent afforded *N*-butyl-4-methoxyaniline as yellow oil (**4d**, 35 mg, 97%).

^1^H NMR (300 MHz, CDCl_3_) δ 6.79 (d, *J* = 8.9 Hz, 2H), 6.59 (d, *J* = 8.9 Hz, 2H), 3.75 (s, 3H), 3.18 (br, 1H), 3.07 (t, *J* = 7.2 Hz, 2H), 1.69 – 1.51 (m, 2H), 1.50 – 1.34 (m, 2H), 0.96 (t, *J* = 7.2 Hz, 3H). ^13^C NMR (75 MHz, CDCl_3_) δ 151.96, 142.81, 114.88, 114.02, 55.81, 44.70, 31.76, 20.29, 13.90.

***N*-Isopropyl-3,4-dimethoxyaniline (4e)** spectral data were in agreement with literature values^48^

Following **GP3**, *tert*-butyl isopropyl(tosyloxy)carbamate (135 mg, 0.3 mmol), TFA (0.45 mL, 6 mmol) in 0.5 mL DCM solution were to give crude *N*-isopropyl-*O*-tosylhydroxylamine **2e**, which was directly used for next step without further purification.

Following **GP5**, 1-(3,4-dimethoxyphenyl)ethan-1-ol (**1ah**, 36 mg, 0.2 mmol), crude *N*-isopropyl-*O*-tosylhydroxylamine (**2e**, 0.3 mmol) in 1.0 mL HFIP solution were stirred for 12 h at room temperature. Purification by silica gel chromatography with PE/EA (4:1) as eluting solvent afforded *N*-isopropyl-3,4-dimethoxyaniline **4e** as brown oil (35 mg, 90%).

^1^H NMR (300 MHz, CDCl_3_) δ 6.74 (d, *J* = 8.5 Hz, 1H), 6.23 (d, *J* = 2.6 Hz, 1H), 6.14 (dd, *J* = 8.5, 2.6 Hz, 1H), 3.83 (s, 3H), 3.80 (s, 3H), 3.55 (hept, *J* = 6.2 Hz, 1H), 3.16 (br, 1H), 1.19 (d, *J* = 6.2 Hz, 6H). ^13^C NMR (75 MHz, CDCl_3_) δ 150.00, 142.38, 141.31, 113.30, 104.36, 99.56, 56.68, 55.66, 45.06, 23.04.

***N*-(2-(Adamantan-1-yl)ethyl)-4-methoxyaniline (4f)** spectral data were in agreement with literature values^49^

Following **GP3**, *tert*-butyl (2-(adamantan-1-yl)ethyl)(tosyloxy)carbamate (135 mg, 0.3 mmol), TFA (0.45 mL, 6 mmol) in 0.5 mL DCM solution were to give crude *N*-(2-(adamantan-1-yl)ethyl)-*O*-tosylhydroxylamine **2f**, which was directly used for next step without further purification.

Following **GP5**, 1-(4-methoxyphenyl)ethan-1-ol (**1a**, 30 mg, 0.2 mmol), crude *N*-(2-(adamantan-1-yl)ethyl)-*O*-tosylhydroxylamine (**2f**, 0.3 mmol) in 1.0 mL HFIP solution were stirred for 12 h at room temperature. Purification by silica gel chromatography with PE/EA (10:1) as eluting solvent afforded *N*-(2-(adamantan-1-yl)ethyl)-4-methoxyaniline **4f** as yellow oil (52 mg, 91%).

^1^H NMR (500 MHz, CDCl_3_) δ 6.79 (d, *J* = 9.0 Hz, 2H), 6.58 (d, *J* = 9.0 Hz, 2H), 3.75 (s, 3H), 3.24 – 2.93 (m, 2H), 1.99 – 1.95 (m, 3H), 1.75 – 1.61 (m, 6H), 1.56 (d, *J* = 2.9 Hz, 6H), 1.41 – 1.35 (m, 2H). ^13^C NMR (125 MHz, CDCl_3_) δ 151.91, 142.88, 114.83, 114.01, 55.80, 44.14, 42.58, 39.53, 37.08, 31.96, 28.61.

***N*-(Cyclopropylmethyl)-4-methoxyaniline (4g)** spectral data were in agreement with literature values^50^

Following **GP3**, *tert*-butyl (cyclopropylmethyl)(tosyloxy)carbamate (102 mg, 0.3 mmol), TFA (0.45 mL, 6 mmol) in 0.5 mL DCM solution were to give crude *N*-(cyclopropylmethyl)-*O*-tosylhydroxylamine **2g**, which was directly used for next step without further purification.

Following **GP5**, 1-(4-methoxyphenyl)ethan-1-ol (**1a**, 30 mg, 0.2 mmol), crude *N*-(cyclopropylmethyl)-*O*-tosylhydroxylamine (**2g**, 0.3 mmol) in 1.0 mL HFIP solution were stirred for 12 h at room temperature. Purification by silica gel chromatography with PE/EA (10:1) as eluting solvent afforded *N*-(cyclopropylmethyl)-4-methoxyaniline **4g** as yellow oil (32 mg, 91%).

^1^H NMR (300 MHz, CDCl_3_) δ 6.79 (d, *J* = 8.9 Hz, 2H), 6.60 (d, *J* = 8.9 Hz, 2H), 3.75 (s, 3H), 3.19 (br, 1H), 2.92 (d, *J* = 6.9 Hz, 2H), 1.19 – 1.03 (m, 1H), 0.67 – 0.48 (m, 2H), 0.26 – 0.20 (m, 2H). ^13^C NMR (75 MHz, CDCl_3_) δ 152.09, 142.76, 114.87, 114.16, 55.80, 50.14, 10.97, 3.38.

**4-Methoxy-*N*-(2-methoxyethyl)aniline (4h)**

Following **GP3**, *tert*-butyl (2-methoxyethyl)(tosyloxy)carbamate (102 mg, 0.3 mmol), TFA (0.45 mL, 6 mmol) in 0.5 mL DCM solution were to give crude *N*-(2-methoxyethyl)-*O*-tosylhydroxylamine **2h**, which was directly used for next step without further purification.

Following **GP5**, 1-(4-methoxyphenyl)ethan-1-ol (**1a**, 30 mg, 0.2 mmol), crude *N*-(2-methoxyethyl)-*O*-tosylhydroxylamine (**2h**, 0.3 mmol) in 1.0 mL HFIP solution were stirred for 12 h at room temperature. Purification by silica gel chromatography with PE/EA (10:1) as eluting solvent afforded 4-methoxy-*N*-(2-methoxyethyl)aniline **4h** as yellow oil (35 mg, 97%).

^1^H NMR (300 MHz, CDCl_3_) δ 6.79 (d, *J* = 8.9 Hz, 2H), 6.61 (d, *J* = 8.9 Hz, 2H), 3.75 (s, 3H), 3.60 (d, *J* = 5.2 Hz, 2H), 3.39 (s, 3H), 3.24 (d, *J* = 5.2 Hz, 2H). ^13^C NMR (75 MHz, CDCl_3_) δ 152.28, 142.41, 114.82, 114.49, 71.10, 58.72, 55.75, 44.53.

***N*-(2-Chloroethyl)-4-methoxyaniline (4i)**

Following **GP3**, *tert*-butyl (2-chloroethyl)(tosyloxy)carbamate (105 mg, 0.3 mmol), TFA (0.45 mL, 6 mmol) in 0.5 mL DCM solution were to give crude *N*-(2-chloroethyl)-*O*-tosylhydroxylamine **2i**, which was directly used for next step without further purification.

Following **GP5**, 1-(4-methoxyphenyl)ethan-1-ol (**1a**, 30 mg, 0.2 mmol), crude *N*-(2-chloroethyl)-*O*-tosylhydroxylamine (**2i**, 0.3 mmol) in 1.0 mL HFIP solution were stirred for 12 h at room temperature. Purification by silica gel chromatography with PE/EA (10:1) as eluting solvent afforded *N*-(2-chloroethyl)-4-methoxyaniline **4i** as yellow oil (35 mg, 95%).

^1^H NMR (400 MHz, CDCl_3_) δ 6.80 (d, *J* = 8.9 Hz, 2H), 6.64 (d, *J* = 8.9 Hz, 2H), 3. (s, 3H), 3.70 (t, *J* = 5.9 Hz, 2H), 3.46 (t, *J* = 5.9 Hz, 2H). ^13^C NMR (100 MHz, CDCl_3_) δ 152.88, 140.87, 115.02, 115.00, 55.74, 46.65, 43.53.

IR (EXTRACT): ṽ = 3385.95, 2997.65, 2956.31, 2830.63, 1617.85, 1513.23, 1461.62, 1441.78, 1363.94, 1296.58, 1272.20, 1238.62, 1179.26, 1149.10, 1121.67, 1032.35, 944.48, 820.76, 731.99, 661.53 cm^-1^

HRMS (EI+) m/z: [M]^+^ calcd 185.0602, found 185.0581.

**Methyl (4-methoxyphenyl)carbamate (4j)** spectral data were in agreement with literature values^51^

Following **GP5**, 1-(4-methoxyphenyl)ethan-1-ol (**1a**, 30 mg, 0.2 mmol), methyl (tosyloxy)carbamate (**2j**, 54 mg, 0.22 mmol) in 1.0 mL HFIP solution were stirred for 12 h at room temperature. Purification by silica gel chromatography with PE/EA (3:1) as eluting solvent afforded methyl (4-methoxyphenyl)carbamate **4j** as white solid (8 mg, 22%).

Following **GP5**, 1-(4-methoxyphenyl)ethan-1-ol (**1a**, 30 mg, 0.2 mmol), methyl (tosyloxy)carbamate (**2j**, 54 mg, 0.22 mmol) in 1.0 mL TFE solution were stirred for 12 h at room temperature. Purification by silica gel chromatography with PE/EA (3:1) as eluting solvent afforded methyl (4-methoxyphenyl)carbamate **4j** as white solid (16 mg, 44%).

^1^H NMR (300 MHz, DMSO) δ 9.41 (br, 1H), 7.34 (d, *J* = 8.8 Hz, 2H), 6.85 (d, *J* = 8.8 Hz, 2H), 3.70 (s, 3H), 3.63 (s, 3H). ^13^C NMR (75 MHz, DMSO) δ 154.75, 154.15, 132.17, 119.82, 113.94, 55.15, 51.46.

**4-Methoxy-*N*-(prop-2-yn-1-yl)aniline (4k)** spectral data were in agreement with literature values^52^

Following **GP3**, *tert*-butyl prop-2-yn-1-yl(tosyloxy)carbamate (98 mg, 0.3 mmol), TFA (0.45 mL, 6 mmol) in 0.5 mL DCM solution were to give crude *N*-(prop-2-yn-1-yl)-*O*-tosylhydroxylamine **2k**, which was directly used for next step without further purification.

Following **GP5**, 1-(4-methoxyphenyl)ethan-1-ol (**1a**, 30 mg, 0.2 mmol), crude *N*-(prop-2-yn-1-yl)-*O*-tosylhydroxylamine (**2k**, 0.3 mmol) in 1.0 mL HFIP solution were stirred for 12 h at room temperature. Purification by silica gel chromatography with PE/EA (5:1) as eluting solvent afforded 4-methoxy-*N*-(prop-2-yn-1-yl)aniline **4k** as yellow oil (30 mg, 94%).

^1^H NMR (500 MHz, CDCl_3_) δ 6.82 (d, *J* = 8.9 Hz, 2H), 6.68 (d, *J* = 8.9 Hz, 2H), 3.90 (d, *J* = 2.4 Hz, 2H), 3.76 (s, 3H), 3.65 (br, 1H), 2.21 (t, *J* = 2.4 Hz, 1H). ^13^C NMR (125 MHz, CDCl_3_) δ 152.90, 140.83, 115.05, 114.73, 81.30, 71.18, 55.65, 34.51.

**4-Methoxy-*N*-(pent-4-en-1-yl)aniline (4l)** spectral data were in agreement with literature values^53^

Following **GP3**, *tert*-butyl pent-4-en-1-yl(tosyloxy)carbamate (107 mg, 0.3 mmol), TFA (0.45 mL, 6 mmol) in 0.5 mL DCM solution were to give crude *N*-(pent-4-en-1-yl)-*O*-tosylhydroxylamine **2l**, which was directly used for next step without further purification.

Following **GP5**, 1-(4-methoxyphenyl)ethan-1-ol (**1a**, 30 mg, 0.2 mmol), crude *N*-(pent-4-en-1-yl)-*O*-tosylhydroxylamine (**2l**, 0.3 mmol) in 1.0 mL HFIP solution were stirred for 12 h at room temperature. Purification by silica gel chromatography with PE/EA (10:1) as eluting solvent afforded 4-methoxy-*N*-(pent-4-en-1-yl)aniline **4l** as brown oil (33 mg, 87%).

^1^H NMR (500 MHz, CDCl_3_) δ 6.79 (d, *J* = 9.1 Hz, 2H), 6.59 (d, *J* = 9.1 Hz, 2H), 5.85 (ddt, *J* = 17.0, 10.0, 6.4 Hz, 1H), 5.06 (dq, *J* = 17.0, 1.9 Hz, 1H), 5.00 (dd, *J* = 10.0, 1.9 Hz, 1H), 3.75 (s, 3H), 3.10 (t, *J* = 7.2 Hz, 2H), 2.17 (q, *J* = 6.4 Hz, 2H), 1.71 (p, *J* = 7.2 Hz, 2H). ^13^C NMR (125 MHz, CDCl_3_) δ 151.95, 142.63, 138.09, 114.99, 114.85, 114.02, 55.79, 44.39, 31.31, 28.70.

**4-Methoxy-*N*-(3-phenylpropyl)aniline (4m)** spectral data were in agreement with literature values^54^

Following **GP3**, *tert*-butyl (3-phenylpropyl)(tosyloxy)carbamate (122 mg, 0.3 mmol), TFA (0.45 mL, 6 mmol) in 0.5 mL DCM solution were to give crude *N*-(3-phenylpropyl)-*O*-tosylhydroxylamine **2m**, which was directly used for next step without further purification.

Following **GP5**, 1-(4-methoxyphenyl)ethan-1-ol (**1a**, 30 mg, 0.2 mmol), crude *N*-(3-phenylpropyl)-*O*-tosylhydroxylamine (**2m**, 0.3 mmol) in 1.0 mL HFIP solution were stirred for 12 h at room temperature. Purification by silica gel chromatography with PE/EA (10:1) as eluting solvent afforded 4-methoxy-*N*-(3-phenylpropyl)aniline **4m** as yellow oil (44 mg, 92%).

^1^H NMR (500 MHz, CDCl_3_) δ 7.33 – 7.28 (m, 2H), 7.26 – 7.19 (m, 3H), 6.79 (d, *J* = 9.1 Hz, 2H), 6.57 (d, *J* = 9.1 Hz, 2H), 3.76 (s, 3H), 3.12 (t, *J* = 7.3 Hz, 2H), 2.75 (t, *J* = 7.3 Hz, 2H), 1.95 (p, *J* = 7.3 Hz, 2H). ^13^C NMR (125 MHz, CDCl_3_) δ 151.97, 142.58, 141.70, 128.38, 128.36, 125.88, 114.83, 114.04, 55.77, 44.40, 33.40, 31.15.

**2.4 Substrate Scope for Primary Anilines**

**General Procedure for the Synthesis of Primary Anilines – GP6**

To a solution of the alcohol (0.2 mmol) in 1.0 mL TFE was added MSH (0.3 mmol) under ambient atmosphere at room temperature, unless otherwise stated. The reaction was stirred at room temperature for 12 h (monitored by GCMS or TLC). Then the reaction was diluted with 1mL DCM and basified with 1mL saturated NaHCO_3_ aqueous solution. The aqueous layer was extracted with DCM (3 mL × 3), and the combined organic layers were washed with 5 mL sat. brine, dried over anhydrous Na_2_SO_4_, filtrated and concentrated in vacuo. The crude residue was purified by silica gel chromatography with PE/EA to afford the desired product.

**4-Methoxyaniline (6a)** spectral data were in agreement with literature values^1^

Following **GP6**, 1-(4-methoxyphenyl)ethan-1-ol (**1a**, 30 mg, 0.2 mmol), MSH (**5m**, 64 mg, 0.3 mmol) in 1.0 mL TFE solution were stirred for 12 h at room temperature. Purification by silica gel chromatography with PE/EA (5:1) as eluting solvent afforded 4-methoxyaniline **6a** as brown solid (19 mg, 79%).

^1^H NMR (300 MHz, CDCl_3_) δ 6.75 (d, *J* = 8.8 Hz, 2H), 6.65 (d, *J* = 8.8 Hz, 2H), 3.75 (s, 3H), 3.42 (br, 2H). ^13^C NMR (75 MHz, CDCl_3_) δ 152.78, 139.88, 116.39, 114.77, 55.70.

**4-(*tert*-Butyl)aniline (6d)** spectral data were in agreement with literature values^55^

Following **GP6**, 1-(4-(*tert*-butyl)phenyl)ethan-1-ol (**1d**, 36 mg, 0.2 mmol), MSH (**5m**, 64 mg, 0.3 mmol) in 1.0 mL TFE solution were stirred for 12 h at room temperature. Purification by silica gel chromatography with PE/EA (10:1) as eluting solvent afforded 4-(*tert*-butyl)aniline **6d** as yellow oil (19 mg, 63%).

^1^H NMR (300 MHz, CDCl_3_) δ 7.20 (d, *J* = 8.6 Hz, 2H), 6.66 (d, *J* = 8.6 Hz, 2H), 3.48 (br, 2H), 1.29 (s, 9H). ^13^C NMR (75 MHz, CDCl_3_) δ 143.75, 141.40, 126.02, 114.90, 33.88, 31.50.

***p*-Toluidine (6ai)** spectral data were in agreement with literature values^24^

Following **GP6**, 1-(*p*-tolyl)ethan-1-ol (**1ai**, 27 mg, 0.2 mmol), MSH (**5m**, 64 mg, 0.3 mmol) in 1.0 mL TFE solution were stirred for 12 h at room temperature. Purification by silica gel chromatography with PE/EA (10:1) as eluting solvent afforded *p*-toluidine **6ai** as brown oil (5 mg, 24%).

^1^H NMR (300 MHz, CDCl_3_) δ 6.98 (d, *J* = 8.1 Hz, 2H), 6.62 (d, *J* = 8.1 Hz, 2H), 3.53 (br, 2H), 2.26 (s, 3H). ^13^C NMR (75 MHz, CDCl_3_) δ 143.79, 129.71, 127.72, 115.21, 20.40.

**4-fluoroaniline (6aj)** spectral data were in agreement with literature values^56^

Following **GP6**, 1-(4-fluorophenyl)ethan-1-ol (**1aj**, 28 mg, 0.2 mmol), MSH (**5m**, 129 mg, 0.6 mmol) in 1.0 mL HFIP solution were stirred for 12 h at room temperature. Purification by silica gel chromatography with PE/EA (10:1) as eluting solvent afforded 4-fluoroaniline **6aj** as yellow oil (12 mg, 55%).

^1^H NMR (300 MHz, CDCl_3_) δ 6.92 – 6.80 (m, 2H), 6.65 – 6.51 (m, 2H), 3.53 (br, 2H). ^13^C NMR (75 MHz, CDCl_3_) δ 156.41 (d, *J* = 235.6 Hz), 142.37 (d, *J* = 2.1 Hz), 116.02 (d, *J* = 7.6 Hz), 115.64 (d, *J* = 22.4 Hz). ^19^F NMR (283 MHz, CDCl_3_) δ -126.84.

***N*-(4-aminophenyl)acetamide (6ak)** spectral data were in agreement with literature values^57^

Following **GP6**, 1-(4-fluorophenyl)ethan-1-ol (**1ak**, 36 mg, 0.2 mmol), MSH (**5m**, 64 mg, 0.3 mmol) in 1.0 mL HFIP solution were stirred for 12 h at room temperature. Purification by silica gel chromatography with PE/EA (1:2) as eluting solvent afforded *N*-(4-aminophenyl)acetamide **6ak** as yellow oil (21 mg, 70%).

^1^H NMR (500 MHz, MeOD) δ 7.23 (d, *J* = 8.8 Hz, 2H), 6.68 (d, *J* = 8.7 Hz, 2H), 2.07 (s, 3H). ^13^C NMR (125 MHz, MeOD) δ 171.27, 145.55, 130.71, 123.16, 116.67, 23.46.

**2,4-Dimethylaniline (6n)** spectral data were in agreement with literature values^58^

Following **GP6**, 1-(2,4-dimethylphenyl)ethan-1-ol (**1n**, 30 mg, 0.2 mmol), MSH (**5m**, 64 mg, 0.3 mmol) in 1.0 mL TFE solution were stirred for 12 h at room temperature. Purification by silica gel chromatography with PE/EA (10:1) as eluting solvent afforded 2,4-dimethylaniline **6n** as brown oil (12 mg, 50%).

^1^H NMR (300 MHz, CDCl_3_) δ 6.88 (s, 1H), 6.86 (d, *J* = 7.8 Hz, 1H), 6.60 (d, *J* = 7.8 Hz, 1H), 3.43 (br, 2H), 2.24 (s, 3H), 2.15 (s, 3H). ^13^C NMR (75 MHz, CDCl_3_) δ 141.94, 131.09, 127.82, 127.29, 122.42, 115.07, 20.38, 17.28.

**2,3-Dihydro-1*H*-inden-5-amine (6al)** spectral data were in agreement with literature values^59^

Following **GP6**, 1-(2,3-dihydro-1*H*-inden-5-yl)ethan-1-ol (**1al**, 32 mg, 0.2 mmol), MSH (**5m**, 64 mg, 0.3 mmol) in 1.0 mL TFE solution were stirred for 12 h at room temperature. Purification by silica gel chromatography with PE/EA (5:1) as eluting solvent afforded 2,3-dihydro-1*H*-inden-5-amine **6al** as yellow solid (18 mg, 67%).

^1^H NMR (300 MHz, CDCl_3_) δ 7.02 (d, *J* = 7.8 Hz, 1H), 6.61 (d, *J* = 2.1 Hz, 1H), 6.51 (dd, *J* = 7.8, 2.1 Hz, 1H), 3.51 (br, 2H), 2.84 (t, *J* = 7.2 Hz, 2H), 2.81 (t, *J* = 7.2 Hz, 2H), 2.05 (p, *J* = 7.2 Hz, 2H). ^13^C NMR (75 MHz, CDCl_3_) δ 145.47, 144.74, 134.22, 124.71, 113.25, 111.45, 32.95, 31.91, 25.64.

**2,4,6-Trimethylaniline (6o)** spectral data were in agreement with literature values^1^

Following **GP6**, 1-mesitylethan-1-ol (**1o**, 33 mg, 0.2 mmol), MSH (**5m**, 64 mg, 0.3 mmol) in 1.0 mL TFE solution were stirred for 12 h at room temperature. Purification by silica gel chromatography with PE/EA (5:1) as eluting solvent afforded 2,4,6-trimethylaniline **6o** as brown oil (20 mg, 74%).

^1^H NMR (300 MHz, CDCl_3_) δ 6.80 (s, 2H), 3.40 (br, 2H), 2.24 (s, 3H), 2.18 (s, 6H). ^13^C NMR (75 MHz, CDCl_3_) δ 140.05, 128.80, 127.12, 121.83, 20.31, 17.53.

**[1,1'-Biphenyl]-4-amine (6p)** spectral data were in agreement with literature values^24^

Following **GP6**, 1-([1,1'-biphenyl]-4-yl)ethan-1-ol (**1p**, 40 mg, 0.2 mmol), MSH (**5m**, 64 mg, 0.3 mmol) in 1.0 mL TFE solution were stirred for 12 h at room temperature. Purification by silica gel chromatography with PE/EA (5:1) as eluting solvent afforded [1,1'-biphenyl]-4-amine **6p** as yellow solid (26 mg, 77%).

^1^H NMR (300 MHz, CDCl_3_) δ 7.56 (dd, *J* = 8.3, 1.3 Hz, 2H), 7.49 – 7.37 (m, 4H), 7.28 (t, *J* = 7.3 Hz, 1H), 6.77 (d, *J* = 8.5 Hz, 2H), 3.73 (br, 2H). ^13^C NMR (75 MHz, CDCl_3_) δ 145.80, 141.12, 131.54, 128.62, 127.97, 126.36, 126.21, 115.35.

**4-Phenoxyaniline (6q)** spectral data were in agreement with literature values^55^

Following **GP6**, 1-(4-phenoxyphenyl)ethan-1-ol (**1q**, 43 mg, 0.2 mmol), MSH (**5m**, 64 mg, 0.3 mmol) in 1.0 mL TFE solution were stirred for 12 h at room temperature. Purification by silica gel chromatography with PE/EA (5:1) as eluting solvent afforded 4-phenoxyaniline **6q** as yellow solid (33 mg, 89%).

^1^H NMR (300 MHz, CDCl_3_) δ 7.29 (dd, *J* = 8.6, 7.3 Hz, 2H), 7.01 (tt, *J* = 7.3, 1.2 Hz, 1H), 6.94 (dd, *J* = 8.6, 1.2 Hz, 2H), 6.88 (d, *J* = 8.7 Hz, 2H), 6.68 (d, *J* = 8.7 Hz, 2H), 3.59 (br, 2H). ^13^C NMR (75 MHz, CDCl_3_) δ 158.87, 148.57, 142.65, 129.48, 122.03, 121.09, 117.19, 116.21.

**Naphthalen-2-amine (6r)** spectral data were in agreement with literature values^55^

Following **GP6**, 1-(naphthalen-2-yl)ethan-1-ol (**1r**, 34 mg, 0.2 mmol), MSH (**5m**, 64 mg, 0.3 mmol) in 1.0 mL TFE solution were stirred for 12 h at room temperature. Purification by silica gel chromatography with PE/EA (5:1) as eluting solvent afforded naphthalen-2-amine **6r** as yellow solid (22 mg, 78%).

^1^H NMR (300 MHz, CDCl_3_) δ 7.71 (d, *J* = 8.2 Hz, 1H), 7.68 (d, *J* = 8.6 Hz, 1H), 7.61 (d, *J* = 8.2 Hz, 1H), 7.39 (ddd, *J* = 8.2, 6.7, 1.2 Hz, 1H), 7.25 (ddd, *J* = 8.2, 6.7, 1.2 Hz, 1H), 6.99 (d, *J* = 2.3 Hz, 1H), 6.95 (dd, *J* = 8.6, 2.3 Hz, 1H), 3.83 (br, 2H). ^13^C NMR (75 MHz, CDCl_3_) δ 144.06, 134.87, 129.16, 127.93, 127.67, 126.30, 125.75, 122.42, 118.19, 108.54.

**4-Bromoaniline (6s)** spectral data were in agreement with literature values^24^

Following **GP6**, bis(4-bromophenyl)methanol (**1s**, 68 mg, 0.2 mmol), MSH (**5m**, 64 mg, 0.3 mmol) in 1.0 mL TFE solution were stirred for 12 h at room temperature. Purification by silica gel chromatography with PE/EA (5:1) as eluting solvent afforded 4-bromoaniline **6s** as yellow solid (19 mg, 56%).

^1^H NMR (300 MHz, CDCl_3_) δ 7.23 (d, *J* = 8.7 Hz, 2H), 6.56 (d, *J* = 8.7 Hz, 2H), 3.66 (br, 2H). ^13^C NMR (75 MHz, CDCl_3_) δ 145.39, 131.98, 116.67, 110.17.

**Dibenzo[*b*,*d*]furan-2-amine (6t)** spectral data were in agreement with literature values^55^

Following **GP6**, 1-(dibenzo[*b*,*d*]furan-2-yl)ethan-1-ol (**1t**, 42 mg, 0.2 mmol), MSH (**5m**, 64 mg, 0.3 mmol) in 1.0 mL TFE solution were stirred for 12 h at room temperature. Purification by silica gel chromatography with PE/EA (5:1) as eluting solvent afforded dibenzo[*b*,*d*]furan-2-amine **6t** as yellow solid (29 mg, 80%).

^1^H NMR (300 MHz, CDCl_3_) δ 7.86 (d, *J* = 7.4 Hz, 1H), 7.52 (d, *J* = 8.3 Hz, 1H), 7.42 (td, *J* = 8.3, 1.2 Hz, 1H), 7.37 (d, *J* = 8.7 Hz, 1H), 7.29 (td, *J* = 7.4, 1.2 Hz, 1H), 7.23 (d, *J* = 2.4 Hz, 1H), 6.82 (dd, *J* = 8.7, 2.4 Hz, 1H), 3.59 (br, 2H). ^13^C NMR (75 MHz, CDCl_3_) δ 156.73, 150.33, 142.01, 126.89, 124.84, 124.28, 122.19, 120.51, 115.72, 111.89, 111.59, 105.94.

**2,3-Dihydrobenzofuran-5-amine (6w)** spectral data were in agreement with literature values^55^

Following **GP6**, 1-(2,3-dihydrobenzofuran-5-yl)ethan-1-ol (**1w**, 32 mg, 0.2 mmol), MSH (**5m**, 64 mg, 0.3 mmol) in 1.0 mL TFE solution were stirred for 12 h at room temperature. Purification by silica gel chromatography with PE/EA (3:1) as eluting solvent afforded 2,3-dihydrobenzofuran-5-amine **6w** as yellow solid (17 mg, 63%).

^1^H NMR (300 MHz, CDCl_3_) δ 6.60 (d, *J* = 8.3 Hz, 1H), 6.59 (d, *J* = 2.4 Hz, 1H), 6.46 (dd, *J* = 8.3, 2.4 Hz, 1H), 4.49 (t, *J* = 8.6 Hz, 2H), 3.30 (br, 2H), 3.12 (t, *J* = 8.6 Hz, 2H). ^13^C NMR (75 MHz, CDCl_3_) δ 153.14, 139.81, 127.76, 114.60, 112.68, 109.26, 70.86, 30.18.

**1-Tosylindolin-5-amine (6x)** spectral data were in agreement with literature values^60^

Following **GP6**, 1-(1-tosylindolin-5-yl)ethan-1-ol (**1x**, 64 mg, 0.2 mmol), MSH (**5m**, 64 mg, 0.3 mmol) in 1.0 mL TFE solution were stirred for 12 h at room temperature. Purification by silica gel chromatography with PE/EA (2:1) as eluting solvent afforded1-tosylindolin-5-amine **6x** as brown solid (22 mg, 38%).

^1^H NMR (300 MHz, CDCl_3_) δ 7.58 (d, *J* = 8.2 Hz, 2H), 7.44 (d, *J* = 8.5 Hz, 1H), 7.18 (d, *J* = 8.2 Hz, 2H), 6.53 (dd, *J* = 8.5, 2.5 Hz, 1H), 6.41 (d, *J* = 2.5 Hz, 1H), 3.86 (t, *J* = 8.2 Hz, 2H), 3.54 (br, 2H), 2.63 (t, *J* = 8.2 Hz, 2H), 2.36 (s, 3H). ^13^C NMR (75 MHz, CDCl_3_) δ 143.69, 143.43, 134.01, 133.93, 133.91, 129.47, 127.37, 117.22, 114.26, 111.84, 50.23, 28.25, 21.49.

**2,6-dimethoxypyridin-3-amine (6am)** spectral data were in agreement with literature values^6^

Following **GP6**, 1-(2,6-dimethoxypyridin-3-yl)ethan-1-ol (**1am**, 36 mg, 0.2 mmol), MSH (**5m**, 64 mg, 0.3 mmol) in 1.0 mL HFIP solution were stirred for 12 h at room temperature. Purification by silica gel chromatography with PE/EA (5:1) as eluting solvent afforded 2,6-dimethoxypyridin-3-amine **6am** as brown oil (20 mg, 65%).

^1^H NMR (500 MHz, CDCl_3_) δ 6.94 (d, *J* = 8.0 Hz, 1H), 6.16 (d, *J* = 8.0 Hz, 1H), 3.96 (s, 3H), 3.84 (s, 3H), 3.40 (br, 2H). ^13^C NMR (125 MHz, CDCl_3_) δ 155.45, 150.99, 125.67, 123.28, 99.21, 53.72, 53.26.

**Methyl 4-aminothiophene-2-carboxylate (6an)**

Following **GP6**, methyl 4-(1-hydroxyethyl)thiophene-2-carboxylate (**1an**, 37 mg, 0.2 mmol), MSH (**5m**, 64 mg, 0.3 mmol) in 1.0 mL HFIP solution were stirred for 12 h at room temperature. Purification by silica gel chromatography with PE/EA (1:2) as eluting solvent afforded methyl 4-aminothiophene-2-carboxylate **6an** as yellow oil (21 mg, 68%).

^1^H NMR (300 MHz, CDCl_3_) δ 7.30 (d, *J* = 1.9 Hz, 1H), 6.39 (d, *J* = 1.8 Hz, 1H), 3.84 (s, 3H), 3.66 (br, 2H). ^13^C NMR (75 MHz, CDCl_3_) δ 162.58, 145.40, 132.29, 126.06, 107.87, 52.04.

IR (ATR): ṽ = 3453, 3365, 3102, 2955, 1693, 1617, 1570, 1450, 1420, 1259, 1187, 1072, 985, 940, 873, 859, 787, 756, 709, 614

HRMS (EI+) m/z: [M]+ calcd 157.0192, found 163.1345157.0182

**3,4-Dimethoxyaniline (6ah)** spectral data were in agreement with literature values^24^

Following **GP6**, 1-(3,4-dimethoxyphenyl)ethan-1-ol (**1ah**, 36 mg, 0.2 mmol), MSH (**5m**, 64 mg, 0.3 mmol) in 1.0 mL TFE solution were stirred for 12 h at room temperature. Purification by silica gel chromatography with PE/EA (2:1) as eluting solvent afforded 3,4-dimethoxyaniline **6ah** as brown solid (22 mg, 72%).

^1^H NMR (400 MHz, CDCl_3_) δ 6.69 (d, *J* = 8.4 Hz, 1H), 6.30 (d, *J* = 2.6 Hz, 1H), 6.22 (dd, *J* = 8.4, 2.6 Hz, 1H), 3.82 (s, 3H), 3.79 (s, 3H), 3.40 (br, 2H). ^13^C NMR (100 MHz, CDCl_3_) δ 149.88, 142.17, 140.64, 113.17, 106.36, 100.74, 56.60, 55.67.

**3,5,5,6,8,8-Hexamethyl-5,6,7,8-tetrahydronaphthalen-2-amine (6y)**

Following **GP6**, 1-(3,5,5,6,8,8-hexamethyl-5,6,7,8-tetrahydronaphthalen-2-yl)ethan-1-ol (**1y**, 52 mg, 0.2 mmol), MSH (**5m**, 64 mg, 0.3 mmol) in 1.0 mL TFE solution were stirred for 12 h at room temperature. Purification by silica gel chromatography with PE/EA (10:1) as eluting solvent afforded 3,5,5,6,8,8-hexamethyl-5,6,7,8-tetrahydronaphthalen-2-amine **6y** as yellow solid (37 mg, 80%).

^1^H NMR (300 MHz, CDCl_3_) δ 7.04 (s, 1H), 6.61 (s, 1H), 3.44 (br, 2H), 2.15 (s, 3H), 1.85 (dqd, *J* = 13.2, 6.7, 2.5 Hz, 1H), 1.62 (t, *J* = 13.2 Hz, 1H), 1.32 (dd, *J* = 13.2, 2.5 Hz, 1H), 1.30 (s, 3H), 1.27 (s, 3H), 1.24 (s, 3H), 1.04 (s, 3H), 0.97 (d, *J* = 6.7 Hz, 3H). ^13^C NMR (75 MHz, CDCl_3_) δ 143.45, 141.96, 136.55, 128.86, 120.63, 112.47, 43.83, 36.91, 34.71, 33.98, 32.37, 32.09, 28.83, 25.07, 17.22, 16.84.

**5-(4-Amino-2,5-dimethylphenoxy)-2,2-dimethylpentanoate (6aa)** spectral data were in agreement with literature values^24^

Following **GP6**, methyl 5-(4-(1-hydroxyethyl)-2,5-dimethylphenoxy)-2,2-dimethylpentanoate (**1aa**, 62 mg, 0.2 mmol), MSH (**5m**, 64 mg, 0.3 mmol) in 1.0 mL TFE solution were stirred for 12 h at room temperature. Purification by silica gel chromatography with PE/EA (3:1) as eluting solvent afforded methyl 5-(4-amino-2,5-dimethylphenoxy)-2,2-dimethylpentanoate **6aa** as brown oil (38 mg, 68%).

^1^H NMR (300 MHz, CDCl_3_) δ 6.55 (s, 1H), 6.50 (s, 1H), 3.87 – 3.75 (m, 2H), 3.66 (s, 3H), 3.29 (br, 2H), 2.14 (s, 3H), 2.13 (s, 3H), 1.77 – 1.57 (m, 4H), 1.21 (s, 6H). ^13^C NMR (75 MHz, CDCl_3_) δ 178.33, 150.15, 137.64, 125.39, 120.39, 118.05, 114.90, 69.17, 51.67, 42.07, 37.12, 25.33, 25.14, 17.34, 15.67.

**Methyl (*S*)-2-(5-amino-6-methoxynaphthalen-2-yl)propanoate (6ab)** spectral data were in agreement with literature values^6^

Following **GP6**, methyl (2*S*)-2-(5-(1-hydroxyethyl)-6-methoxynaphthalen-2-yl)propanoate (**1ab**, 58 mg, 0.2 mmol), MSH (**5m**, 64 mg, 0.3 mmol) in 1.0 mL TFE solution were stirred for 12 h at room temperature. Purification by silica gel chromatography with PE/EA (3:1) as eluting solvent afforded methyl (*S*)-2-(5-amino-6-methoxynaphthalen-2-yl)propanoate **6ab** as brown oil (39 mg, 75%).

^1^H NMR (300 MHz, CDCl_3_) δ 7.79 (d, *J* = 7.9 Hz, 1H), 7.70 (d, *J* = 1.9 Hz, 1H), 7.43 (dd, *J* = 8.8, 1.9 Hz, 1H), 7.34 (d, *J* = 8.9 Hz, 1H), 7.30 (d, *J* = 7.9 Hz, 1H), 4.27 (br, 2H), 4.01 (s, 3H), 3.91 (q, *J* = 7.1 Hz, 1H), 3.72 (s, 3H), 1.63 (d, *J* = 7.1 Hz, 3H). ^13^C NMR (75 MHz, CDCl_3_) δ 175.11, 142.56, 135.54, 129.56, 129.43, 126.53, 124.78, 123.02, 120.87, 118.27, 113.87, 56.71, 52.01, 45.27, 18.44.

**Methyl 2-(4-aminophenoxy)-2-methylpropanoate (6ac)**

Following **GP6**, methyl 2-(4-((4-chlorophenyl)(hydroxy)methyl)phenoxy)-2-methylpropanoate (**1ac**, 67 mg, 0.2 mmol), MSH (**5m**, 64 mg, 0.3 mmol) in 1.0 mL TFE solution were stirred for 12 h at room temperature. Purification by silica gel chromatography with PE/EA (3:1) as eluting solvent afforded methyl 2-(4-aminophenoxy)-2-methylpropanoate **6ac** as brown oil (23 mg, 56%).

^1^H NMR (500 MHz, CDCl_3_) δ 6.72 (d, *J* = 8.8 Hz, 2H), 6.56 (d, *J* = 8.8 Hz, 2H), 3.76 (s, 3H), 3.50 (br, 2H), 1.50 (s, 6H). ^13^C NMR (125 MHz, CDCl_3_) δ 174.90, 147.36, 141.97, 122.04, 115.69, 79.68, 52.30, 25.17.

IR (REFLEXION): ṽ = 3446, 3369, 3225, 3036, 2991, 2952, 2850, 1875, 1740, 1627, 1512, 1465, 1436, 1383, 1365, 1284, 1224, 1172, 1146, 1011, 963, 885, 831, 773, 751, 646 cm^-1^

HRMS (EI+) m/z: [M]^+^ calcd 209.1046, found 209.1047.

**(8*R*,9*S*,13*S*,14*S*)-3-Amino-13-methyl-7,8,9,11,12,13,14,15,16,17-decahydro-6*H*-cyclopenta[*a*]phenanthren-17-ol (6ae)**

Following **GP6**, (8*R*,9*S*,13*S*,14*S*)-3-(1-hydroxyethyl)-13-methyl-7,8,9,11,12,13,14,15,16,17-decahydro-6*H*-cyclopenta[*a*]phenanthren-17-ol (**1ae**, 60 mg, 0.2 mmol), MSH (**5m**, 64 mg, 0.3 mmol) in 1.0 mL TFE solution were stirred for 12 h at room temperature. Purification by silica gel chromatography with PE/EA (2:1) as eluting solvent afforded (8*R*,9*S*,13*S*,14*S*)-3-amino-13-methyl-7,8,9,11,12,13,14,15,16,17-decahydro-6*H*-cyclopenta[*a*]phenanthren-17-ol **6ae** as white solid (36 mg, 67%).

^1^H NMR (500 MHz, CDCl_3_) δ 7.09 (d, *J* = 8.3 Hz, 1H), 6.52 (dd, *J* = 8.3, 2.6 Hz, 1H), 6.45 (d, *J* = 2.6 Hz, 1H), 3.73 (t, *J* = 8.5 Hz, 1H), 2.95 – 2.62 (m, 2H), 2.32 – 2.26 (m, 1H), 2.20 – 2.05 (m, 2H), 1.93 (dt, *J* = 12.6, 3.4 Hz, 1H), 1.87 – 1.80 (m, 1H), 1.73 – 1.63 (m, 1H), 1.58 – 1.24 (m, 6H), 1.22 – 1.15 (m, 1H), 0.77 (s, 3H). ^13^C NMR (125 MHz, CDCl_3_) δ 143.91, 137.60, 130.70, 126.18, 115.40, 112.97, 81.89, 49.95, 43.88, 43.22, 38.91, 36.66, 30.53, 29.58, 27.25, 26.27, 23.07, 11.03.

**2.5 Further Application**

***N*-Methyl-[1,1'-biphenyl]-2-amine (7)** spectral data were in agreement with literature values^61^

Following **GP5**, 1-([1,1'-biphenyl]-2-yl)ethan-1-ol (**1ao**, 40 mg, 0.2 mmol), TsONHMe (**2a**, 44 mg, 0.22 mmol) in 1 mL HFIP solution were stirred for 12 h at room temperature. Purification by silica gel chromatography with PE/EA (10:1) as eluting solvent afforded *N*-methyl-[1,1'-biphenyl]-2-amine **7** as yellow oil (22 mg, 61%).

^1^H NMR (300 MHz, CDCl_3_) δ 7.51 – 7.42 (m, 4H), 7.40 – 7.34 (m, 1H), 7.30 (td, *J* = 8.0, 1.7 Hz, 1H), 7.12 (dd, *J* = 7.4, 1.7 Hz, 1H), 6.80 (td, *J* = 7.4, 1.7 Hz, 1H), 6.72 (d, *J* = 8.0 Hz, 1H), 3.99 (br, 1H), 2.82 (s, 3H). ^13^C NMR (75 MHz, CDCl_3_) δ 146.14, 139.48, 129.99, 129.38, 128.82, 128.73, 127.54, 127.14, 116.76, 109.75, 30.73.

**[1,1'-Biphenyl]-2-amine (8)** spectral data were in agreement with literature values^62^

Following **GP6**, 1-([1,1'-biphenyl]-2-yl)ethan-1-ol (**1ao**, 40 mg, 0.2 mmol), MSH (**5m**, 64 mg, 0.3 mmol) in 1.0 mL TFE solution were stirred for 12 h at room temperature. Purification by silica gel chromatography with PE/EA (10:1) as eluting solvent afforded [1,1'-biphenyl]-2-amine **8** as yellow solid (21 mg, 62%).

^1^H NMR (300 MHz, CDCl_3_) δ 7.58 – 7.41 (m, 4H), 7.44 – 7.30 (m, 1H), 7.23 – 7.07 (m, 2H), 6.85 (td, *J* = 7.6, 1.2 Hz, 1H), 6.78 (d, *J* = 7.6 Hz, 1H), 3.77 (br, 2H). ^13^C NMR (75 MHz, CDCl_3_) δ 143.45, 139.49, 130.41, 129.05, 128.76, 128.45, 127.60, 127.11, 118.60, 115.56.

**2,6-Dimethylaniline (9)** spectral data were in agreement with literature values^63^

Following **GP6**, 1-(2,6-dimethylphenyl)ethan-1-ol (**1ap**, 150 mg, 1 mmol), MSH (**5m**, 322 mg, 1.5 mmol) in 5.0 mL TFE solution were stirred for 12 h at room temperature. Purification by silica gel chromatography with PE/EA (10:1) as eluting solvent afforded 2,6-dimethylaniline **9** as yellow oil (45 mg, 37%).

^1^H NMR (300 MHz, CDCl_3_) δ 6.97 (d, *J* = 7.4 Hz, 2H), 6.67 (t, *J* = 7.4 Hz, 1H), 3.50 (br, 2H), 2.21 (s, 6H). ^13^C NMR (75 MHz, CDCl_3_) δ 142.66, 128.19, 121.64, 117.94, 17.55.

**Methyl 4-(4-aminophenyl)butanoate (10)** spectral data were in agreement with literature values^64^

Following **GP6**, methyl 4-(4-(1-hydroxyethyl)phenyl)butanoate (**1aq**, 44 mg, 0.2 mmol), MSH (**5m**, 64 mg, 0.3 mmol) in 1.0 mL TFE solution were stirred for 12 h at room temperature. Purification by silica gel chromatography with PE/EA (4:1) as eluting solvent afforded methyl 4-(4-aminophenyl)butanoate **10** as yellow solid (21 mg, 55%).

^1^H NMR (300 MHz, CDCl_3_) δ 6.96 (d, *J* = 8.3 Hz, 2H), 6.62 (d, *J* = 8.3 Hz, 2H), 3.66 (s, 3H), 3.55 (br, 2H), 2.54 (t, *J* = 7.5 Hz, 2H), 2.31 (t, *J* = 7.5 Hz, 2H), 1.90 (p, *J* = 7.9 Hz, 2H). ^13^C NMR (75 MHz, CDCl_3_) δ 174.06, 144.36, 131.34, 129.21, 115.20, 51.41, 34.20, 33.32, 26.74.

**4-methoxy-*N*-methylaniline (3a)**

Following **GP5**, 1-methoxy-4-(1-methoxyethyl)benzene (**11**, 33 mg, 0.2 mmol) and TsONHMe (**2a**, 44 mg, 0.22 mmol) in 1 mL HFIP were stirred for 12 h at room temperature. Purification by silica gel chromatography with PE/EA (10:1) as eluting solvent afforded 4-methoxy-*N*-methylaniline **3a** as yellow oil (18 mg, 67%).

Following **GP5**, 1-(4-methoxyphenyl)ethyl acetate (**12**, 39 mg, 0.2 mmol) and TsONHMe (**2a**, 44 mg, 0.22 mmol) in 1 mL HFIP were stirred for 12 h at room temperature. Purification by silica gel chromatography with PE/EA (10:1) as eluting solvent afforded 4-methoxy-*N*-methylaniline **3a** as yellow oil (17 mg, 63%).

***N*-ethyl-4-methoxy-*N*-methylaniline (13)** spectral data were in agreement with literature values^65^

To a solution of 1-(4-methoxyphenyl)ethan-1-ol (**2a**, 30 mg, 0.2 mmol) in 1.0 mL HFIP was added TsONHMe (**2a**, 44 mg, 0.22 mmol) at room temperature, and the reaction was stirred for 12 h at room temperature. Then NaBH_3_CN (64 mmol, 1.0 mmol) was added to the above reaction mixture and the reaction was stirred for 3 h at room temperature. The reaction was quenched with 1mL saturated NaHCO_3_ aq., extracted with DCM (3 mL × 3), and the combined organic layers were washed with 5 mL sat. brine, dried over anhydrous Na_2_SO_4_, filtrated and concentrated in vacuo. The crude residue was purified by silica gel chromatography with PE/EA (10:1) as eluting solvent to afford *N*-ethyl-4-methoxy-*N*-methylaniline **13** as yellow oil (18 mg, 55%)

^1^H NMR (300 MHz, CDCl_3_) δ 6.84 (d, *J* = 9.1 Hz, 2H), 6.73 (d, *J* = 9.1 Hz, 2H), 3.77 (s, 3H), 3.31 (q, *J* = 7.1 Hz, 2H), 2.84 (s, 3H), 1.09 (t, *J* = 7.1 Hz, 3H). ^13^C NMR (75 MHz, CDCl_3_) δ 151.68, 144.23, 115.00, 114.71, 55.75, 47.99, 38.26, 11.03.

**4-Methoxyphenol (14)** spectral data were in agreement with literature values^66^

To a solution of 1-(4-methoxyphenyl)ethan-1-ol (**1a**, 30 mg, 0.2 mmol) in 1.0 mL HFIP was added 35% H_2_O_2_ (29 mg, 0.3 mmol) at room temperature, and the reaction was stirred for 12 h at room temperature. Then the reaction was diluted with 1mL DCM and basified with 1mL saturated NaHCO_3_ aq., extracted with DCM (3 mL × 3), and the combined organic layers were washed with 5 mL sat. brine, dried over anhydrous Na_2_SO_4_, filtrated and concentrated in vacuo. The crude residue was purified by silica gel chromatography with PE/EA (10:1) as eluting solvent to afford 4-methoxyphenol **14** as yellow solid (16 mg, 64%)

^1^H NMR (300 MHz, CDCl_3_) δ 6.83 – 6.74 (m, 4H), 4.95 (br, 1H), 3.77 (s, 3H). ^13^C NMR (75 MHz, CDCl_3_) δ 153.68, 149.48, 116.04, 114.86, 55.80.

**1-Bromo-4-methoxybenzene (15)** spectral data were in agreement with literature values^67^

To a solution of 1-(4-methoxyphenyl)ethan-1-ol (**1a**, 30 mg, 0.2 mmol) in 1.0 mL TFE was added NBS (35 mg, 0.2 mmol) under nitrogen atmosphere. Then the reaction was stirred under UV light for 12 h at room temperature. The reaction was concentrated in vacuo, and the residue was purified by silica gel chromatography with PE/EA (20:1) as eluting solvent to afford 1-bromo-4-methoxybenzene **15** as colorless liquid (20 mg, 54%).

^1^H NMR (300 MHz, CDCl_3_) δ 7.38 (d, *J* = 9.0 Hz, 2H), 6.78 (d, *J* = 9.0 Hz, 2H), 3.78 (s, 3H). ^13^C NMR (75 MHz, CDCl_3_) δ 158.68, 132.22, 115.71, 112.80, 55.42.

**2.6 Large-Scale Reaction**

Following **GP5**, 1-(4-chlorophenyl)ethan-1-ol (**1h**, 626 mg, 4 mmol), TsONHMe (**2a**, 885 mg, 4.4 mmol) in 20 mL HFIP solution were stirred for 12 h at room temperature. Purification by silica gel chromatography with PE/EA (10:1) as eluting solvent afforded 4-chloro-*N*-methylaniline **3h** as yellow oil (478 mg, 84%).

Following **GP6**, 1-(4-methoxyphenyl)ethan-1-ol (**1a**, 608 mg, 4 mmol), MSH (**5m**, 1676 mg, 6 mmol) in 20 mL TFE solution were stirred for 12 h. Purification by silica gel chromatography with PE/EA (5:1) as eluting solvent afforded 4-methoxyaniline **6a** as brown solid (388 mg, 79%).

**2.7 Evaluation of MSH Storage Time**

New prepared MSH (*O*-(mesitylsulfonyl)hydroxylamine) was stored at -20^o^C, and NMR was used to measure how MSH was decomposed at different time. From the figure below, we can see: new prepared MSH was 100% pure; around 5% decomposition after 10 days storage at -20^o^C; but around 20% decomposition after 40 days storage at -20^o^C.

Besides, A competitive reaction was done to check the efficiency of MSH, which was stored at -20^o^C after 40 days: MSH (**5m**, 1.67 g) reacted with 1-(4-methoxyphenyl)ethan-1-ol (**1a**, 608 mg), delivering 4-methoxylaniline (**6a**, 388 mg, 79%), almost without any weight loss compared to small-scale reaction. (details see **Large-Scale Reaction** above), i.e. MSH, stored for 40 days at -20^o^C, doesn’t have any negative effect on the yield.


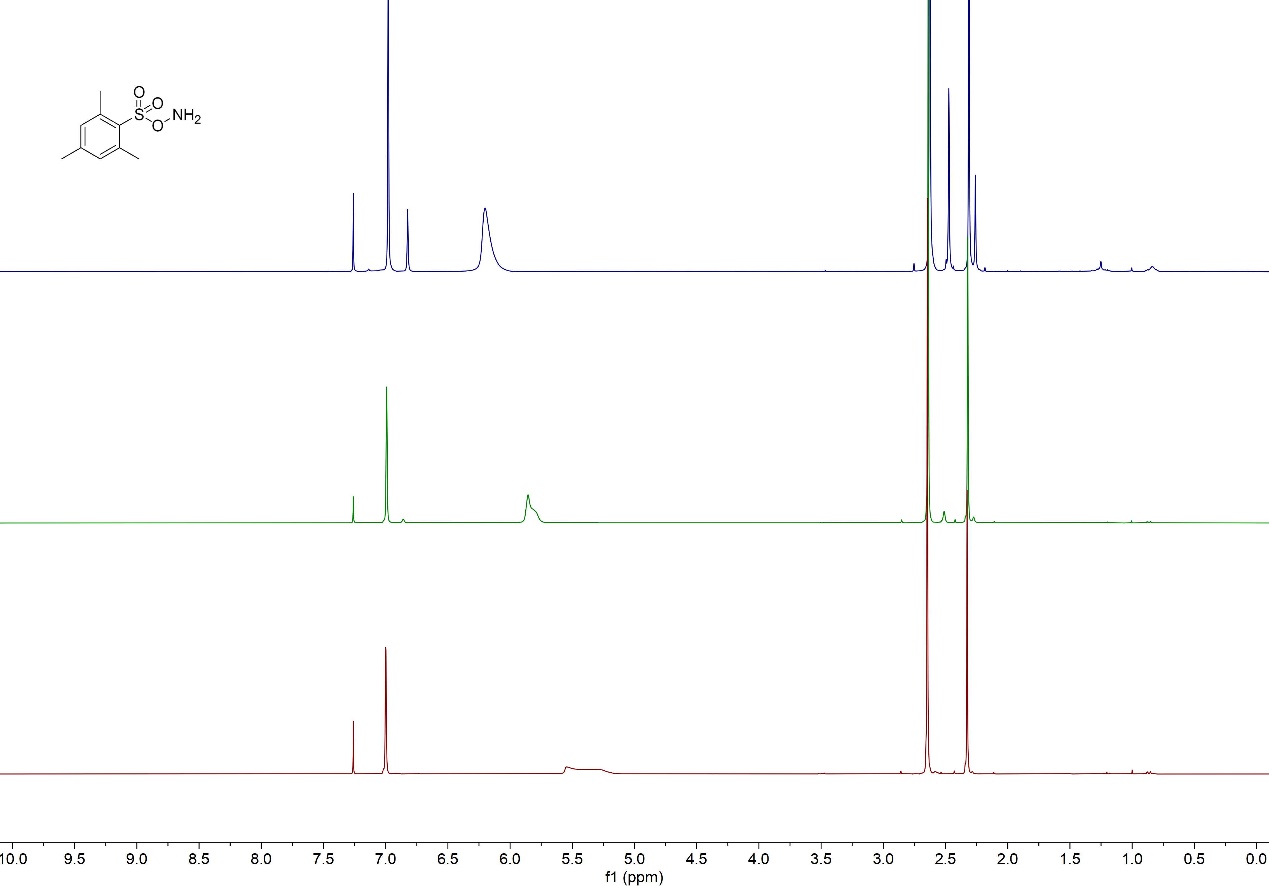


newly prepared MSH

40 days after storage at -20^o^C

10 days after storage at -20^o^C

**Supplementary Fig 1.** NMR monitoring of the stability of MSH over a period of 40 days

**2.8 Control Experiments**

The yield was decreased when BHT or 1,1-diphenylethylene as an additive in the standard reaction (1-(4-methoxyphenyl)ethan-1-ol **1a** and TsONHMe **2a** in HFIP), which, however, doesn’t support a radical pathway involved in the reaction. And this can be explained: simple BHT could also react with TsONHMe via stepwise or concerted pathway,^1^ affording the same adduct 2,6-di-*tert*-butyl-4-methyl-4-(methylamino)cyclohexa-2,5-dien-1-one as in the below (reaction **a**, figure 1); ethylene might be not compatible with TsONHMe.^68^ That’s the reason of BHT or 1,1-diphenylethylene inhibiting the reaction. More proof from reaction **c** in Scheme 1: no radical was involved in the reaction (no signal was detected on EPR), and same yield was acquired even with the DMPO as an additive compared to the standard reaction.

**Supplementary Fig 2.** Mechanistic Experiments

**a.** To a solution of 1-(4-methoxyphenyl)ethan-1-ol (**1a**, 30 mg, 0.2 mmol) and BHT (48 mg, 0.2 mmol) in 1 mL HFIP was slowly added TsONHMe (**2a**, 44 mg, 0.22 mmol), and the reaction was stirred for 12 h at room temperature. The reaction was quenched with 1 mL sat. NaHCO_3_ aq., extracted with DCM (3 × 3 mL), and the combined organic layers were washed with 5 mL sat. brine, dried over anhydrous Na_2_SO_4_, filtrated and concentrated in vacuo. The residue was purified by silica gel chromatography with PE/EA (10:1) as eluting solvent afforded 4-methoxy-*N*-methylaniline **3a** as yellow oil (18 mg, 67%) and 2,6-di-*tert*-butyl-4-methyl-4-(methylamino)cyclohexa-2,5-dien-1-one (7 mg, 13 %).

**2,6-Di-*tert*-butyl-4-methyl-4-(methylamino)cyclohexa-2,5-dien-1-one** spectral data were in agreement with literature values^1^

^1^H NMR (300 MHz, CDCl_3_) δ 6.37 (s, 2H), 2.18 (s, 3H), 2.03 (s, 1H), 1.29 (s, 3H), 1.23 (s, 18H). ^13^C NMR (75 MHz, CDCl_3_) δ 186.32, 148.05, 144.57, 54.32, 34.79, 30.38, 29.62, 27.58.

**b.** To a solution of 1-(4-methoxyphenyl)ethan-1-ol (**1a**, 30 mg, 0.2 mmol) and 1,1-diphenylethylene (40 mg, 0.2 mmol) in 1 mL HFIP was slowly added TsONHMe (**2a**, 44 mg, 0.22 mmol), and the reaction was stirred for 12 h at room temperature. The reaction was quenched with 1 mL sat. NaHCO_3_, extracted with DCM (3 × 3 mL), and the combined organic layers were washed with 5 mL sat. brine, dried over anhydrous Na_2_SO_4_, filtrated and concentrated in vacuo. The residue was purified by silica gel chromatography with PE/EA (10:1) as eluting solvent afforded 4-methoxy-*N*-methylaniline **3a** as yellow oil (12 mg, 44%).

**c.** To a solution of 1-(4-methoxyphenyl)ethan-1-ol (**1a**, 30 mg, 0.2 mmol) and DMPO (44 mg, 0.2 mmol) in 1 mL HFIP was slowly added TsONHMe (**2a**, 44 mg, 0.22 mmol), and the reaction was stirred for 12 h under argon atmosphere at room temperature. The reaction was quenched with 1 mL sat. NaHCO_3_, extracted with DCM (3 × 3 mL), and the combined organic layers were washed with 5 mL sat. brine, dried over anhydrous Na_2_SO_4_, filtrated and concentrated in vacuo. The residue was purified by silica gel chromatography with PE/EA (10:1) as eluting solvent afforded 4-methoxy-*N*-methylaniline **3a** as yellow oil (20 mg, 74%). And DMPO was also detected with GC-MS (m/z 113.1). No radicals were detected by EPR machine when the same reaction was prepared under argon atmosphere at room temperature and.

**2.9 Mechanistic Studies**

**Supplementary Fig 3.** Mechanistic studies

**a.** Following **GP5**, 1-(1-bromoethyl)-4-chlorobenzene (**16**, 44 mg, 0.2 mmol) and TsONHMe (**2a**, 44 mg, 0.22 mmol) in 1 mL HFIP were stirred for 12 h at room temperature. Purification by silica gel chromatography with PE/EA (10:1) as eluting solvent afforded 4-chloro-*N*-methylaniline **3h** as yellow oil (12 mg, 43%)

**b.** Following **GP6**, 4-(1-hydroxyethyl)phenyl 4-methylbenzenesulfonate (**1i**, 59 mg, 0.2 mmol), MSH (**5m**, 84 mg, 0.3 mmol) in 1 mL TFE solution were stirred for 12 h. Purification by silica gel chromatography with PE/EA (10:1) as eluting solvent afforded 4-(1-(2,2,2-trifluoroethoxy)ethyl)phenyl 4-methylbenzenesulfonate **17** as colorless oil (56 mg, 75%).

**4-(1-(2,2,2-Trifluoroethoxy)ethyl)phenyl 4-methylbenzenesulfonate (17)**

^1^H NMR (300 MHz, CDCl_3_) δ 7.72 (d, *J* = 8.2 Hz, 2H), 7.32 (d, *J* = 8.2 Hz, 2H), 7.24 (d, *J* = 8.6 Hz, 2H), 6.99 (d, *J* = 8.6 Hz, 2H), 4.54 (q, *J* = 6.5 Hz, 1H), 3.62 (q, *J* = 8.7 Hz, 2H), 2.45 (s, 3H), 1.45 (d, *J* = 6.5 Hz, 3H). ^13^C NMR (75 MHz, CDCl_3_) δ 149.20, 145.46, 140.84, 132.43, 129.76, 128.43, 127.39, 123.90 (q, *J* = 278.7 Hz), 122.64, 78.73, 65.83 (q, *J* = 34.2 Hz), 23.59, 21.66. ^19^F NMR (283 MHz, CDCl_3_) δ -74.17.

IR (EXTRACT): ṽ = 3069.15, 2981.11, 2932.91, 2106.42, 1914.34, 1731.83, 1598.22, 1502.87, 1434.99, 1372.87, 1275.81, 1197.92, 1152.36, 1130.84, 1091.38, 1016.90, 964.54, 862.22, 814.53, 779.09, 741.92, 707.89, 684.29, 657.77, 608.58 cm^-1^

HRMS (EI+) m/z: [M]^+^ calcd 374.0794, found 374.0790.

**c.** To a solution of 1-isopropyl-4-methoxybenzene (**18**, 30 mg, 0.2 mmol) in 1 mL HFIP or TFA was slowly added DDQ (50 mg, 0.22 mmol) and TsONHMe (**2a**, 44 mg, 0.22 mmol), and the reaction was stirred for 12 h at room temperature. The reaction was quenched with 1 mL sat. NaHCO_3_, extracted with DCM (3 × 3 mL), and the combined organic layers were washed with 5 mL sat. brine, dried over anhydrous Na_2_SO_4_, filtrated and concentrated in vacuo. The residue was purified by silica gel chromatography with PE/EA (10:1) as eluting solvent afforded desired products: HFIP as solvent: 4-methoxy-*N*-methylaniline **3a** as yellow oil (5 mg, 18%) and 5-isopropyl-2-methoxy-*N*-methylaniline **19** as yellow oil (12 mg, 34%); TFA as solvent: 4-methoxy-*N*-methylaniline **3a** as yellow oil (13 mg, 48%) and 5-isopropyl-2-methoxy-*N*-methylaniline **19** as yellow oil (2 mg, 6%).

**5-Isopropyl-2-methoxy-*N*-methylaniline (19)** spectral data were in agreement with literature values^1^

^1^H NMR (300 MHz, CDCl_3_) δ 6.71 (d, *J* = 8.1 Hz, 1H), 6.55 (dd, *J* = 8.1, 2.0 Hz, 1H), 6.50 (d, *J* = 2.0 Hz, 1H), 4.21 (s, 1H), 3.83 (s, 3H), 2.89 (s, 3H), 2.88 – 2.78 (m, 1H), 1.26 (d, *J* = 6.9 Hz, 6H). ^13^C NMR (75 MHz, CDCl_3_) δ 145.10, 141.97, 139.11, 113.42, 109.08, 107.92, 55.47, 33.88, 30.39, 24.26.

**d.** See **Substrate Scope for Secondary Anilines**

**e.** Following **GP5**, 1-(4-methoxyphenyl)ethan-1-one (**20**, 30 mg, 0.2 mmol) and TsONHMe (**2a**, 44 mg, 0.22 mmol) in 1 mL HFIP were stirred for 12 h at room temperature. The reaction mixture was detected with GC-MS: 4-methoxy-*N*-methylaniline **3a** was formed

**2.10 NMR Spectra**


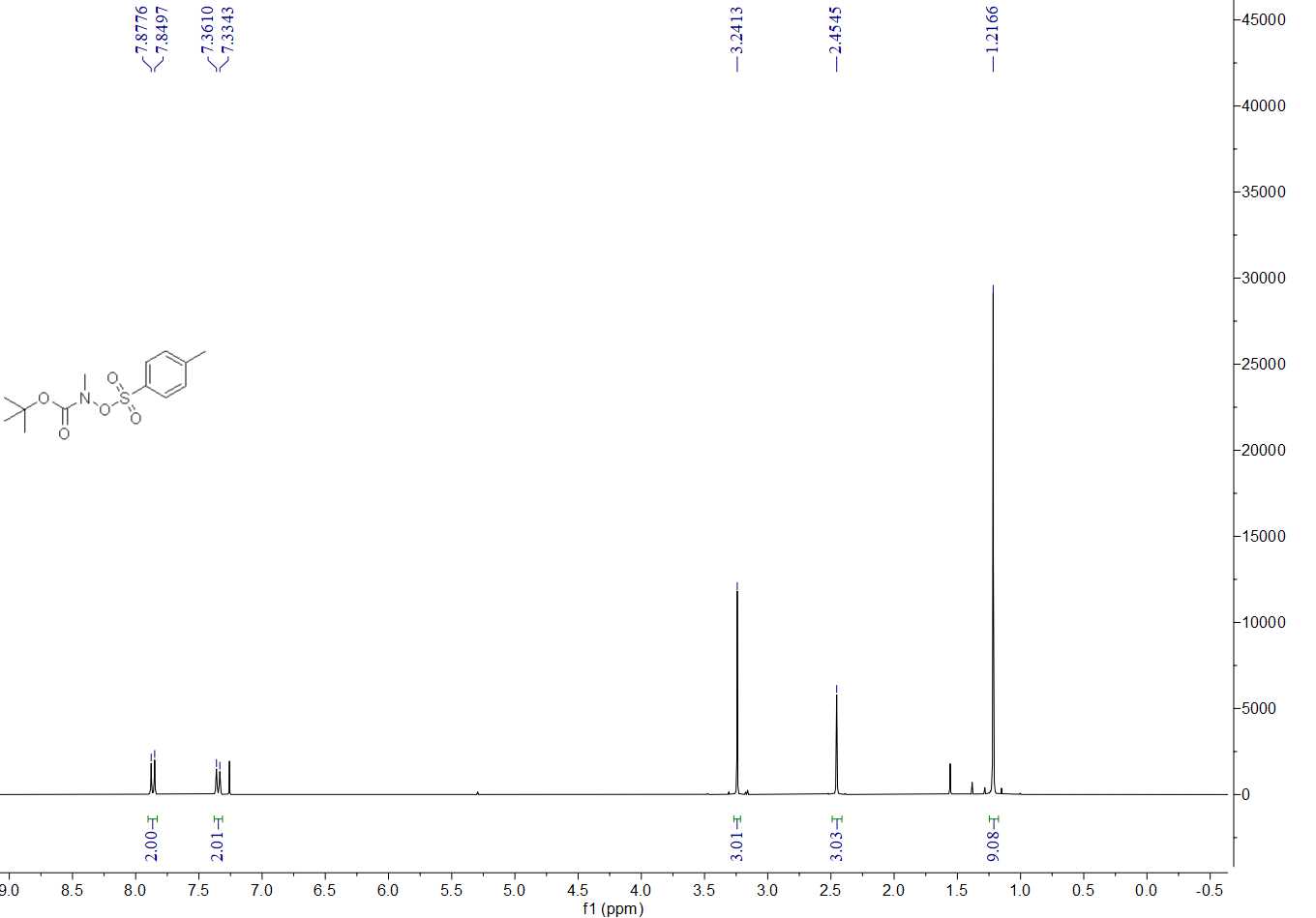


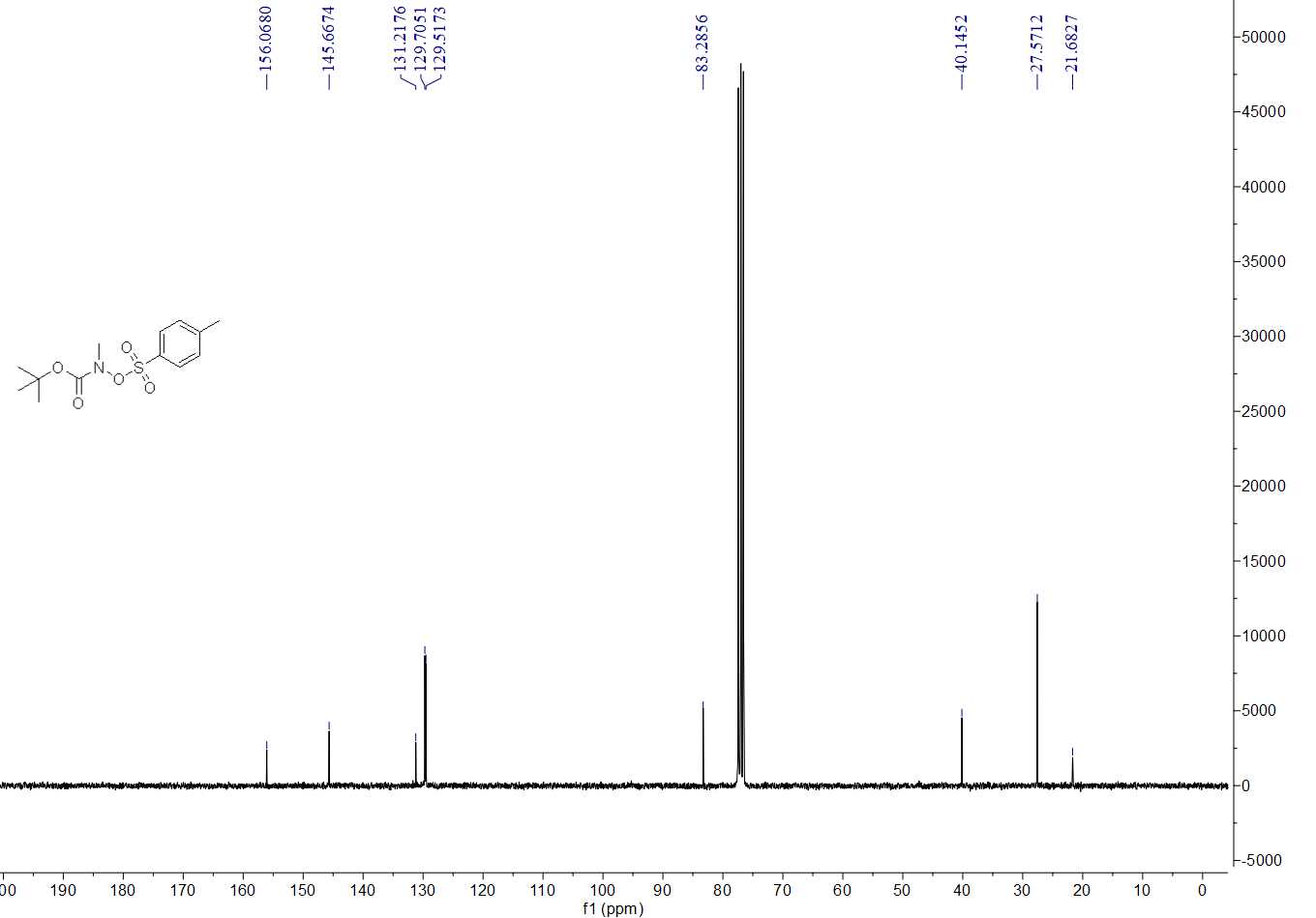


**Supplementary Fig 4.** ^1^H (upper part) and ^13^C NMR (lower part) of MsONBocMe


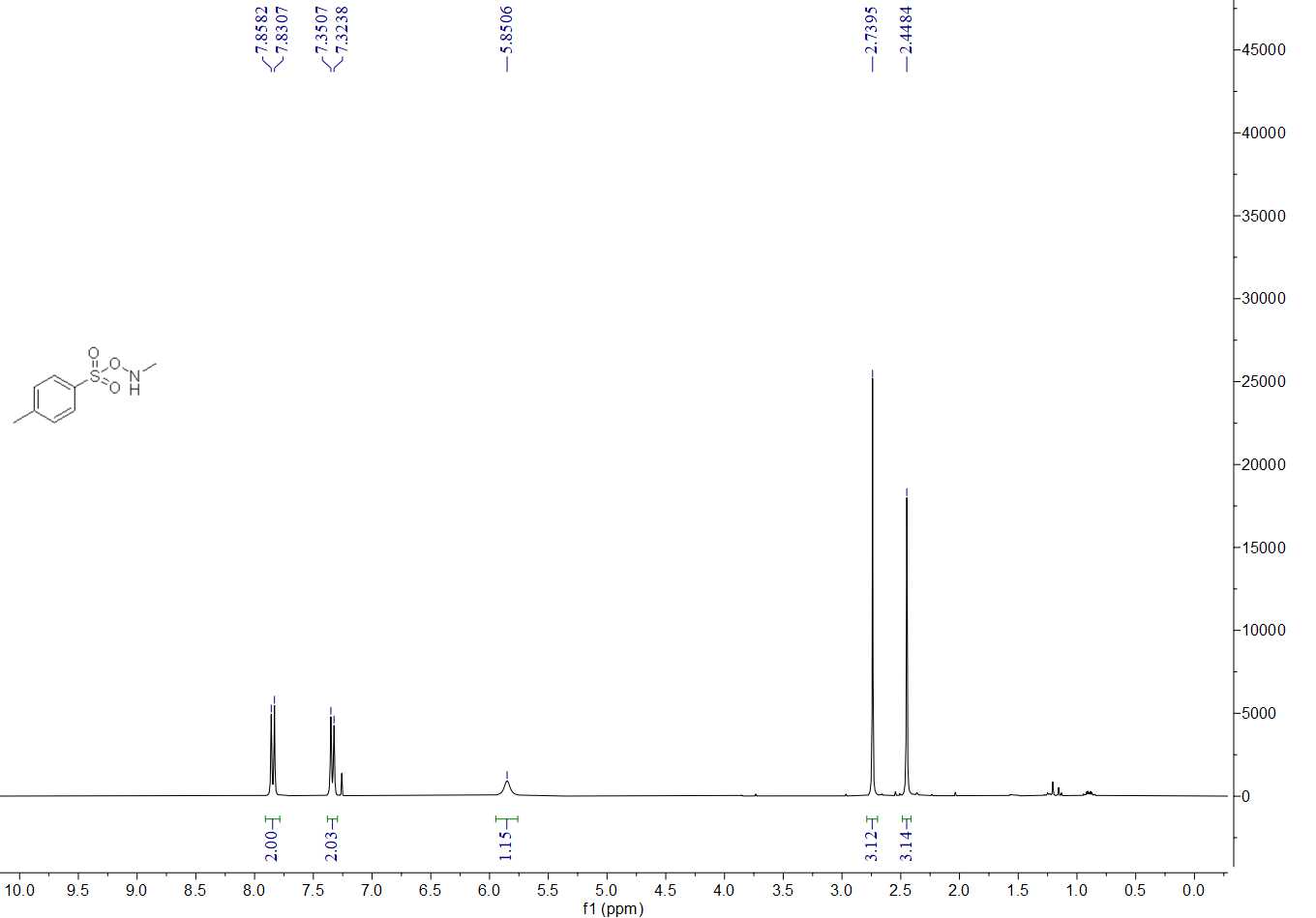


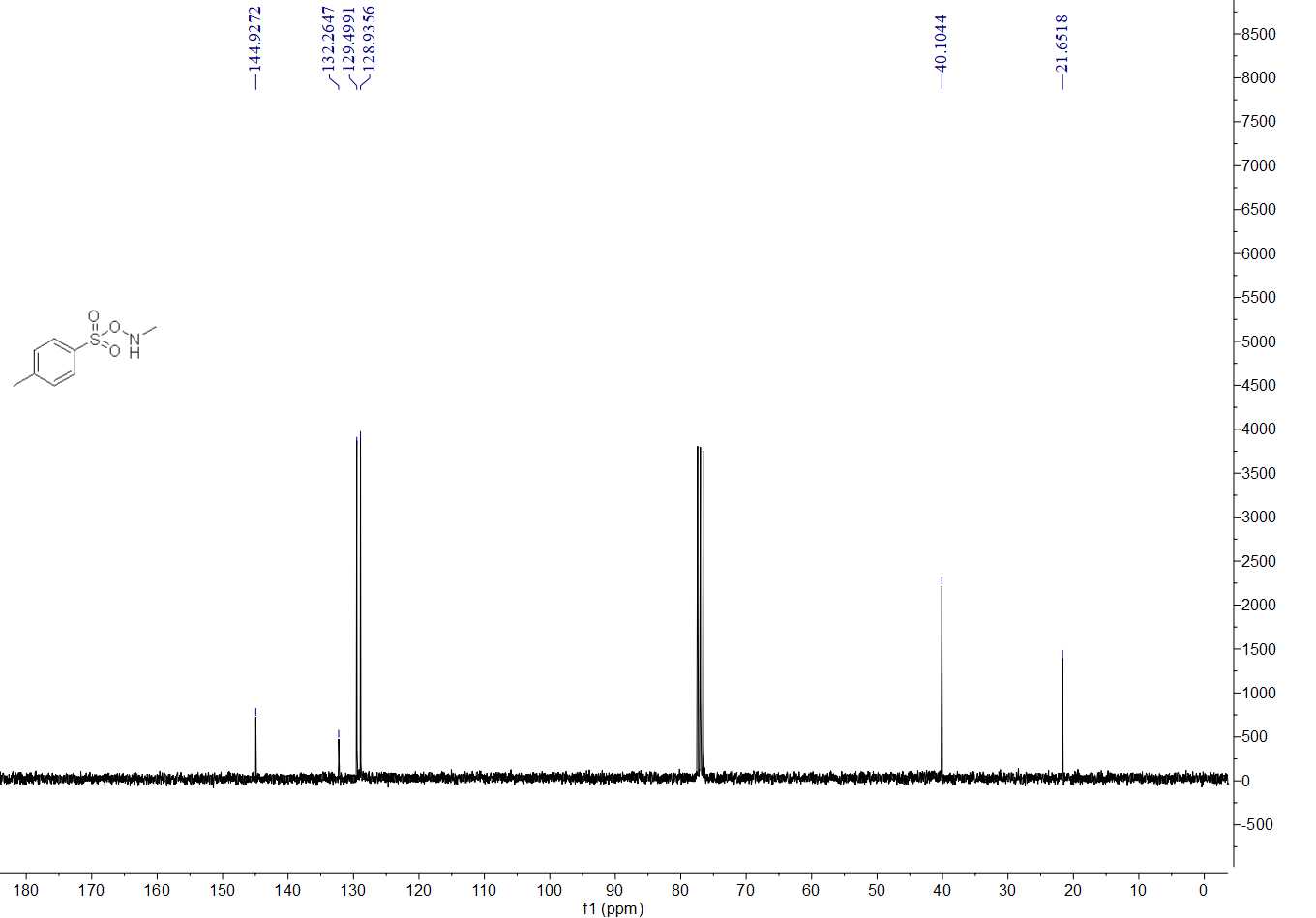


**Supplementary Fig 5.** ^1^H (upper part) and ^13^C NMR (lower part) of **2a**


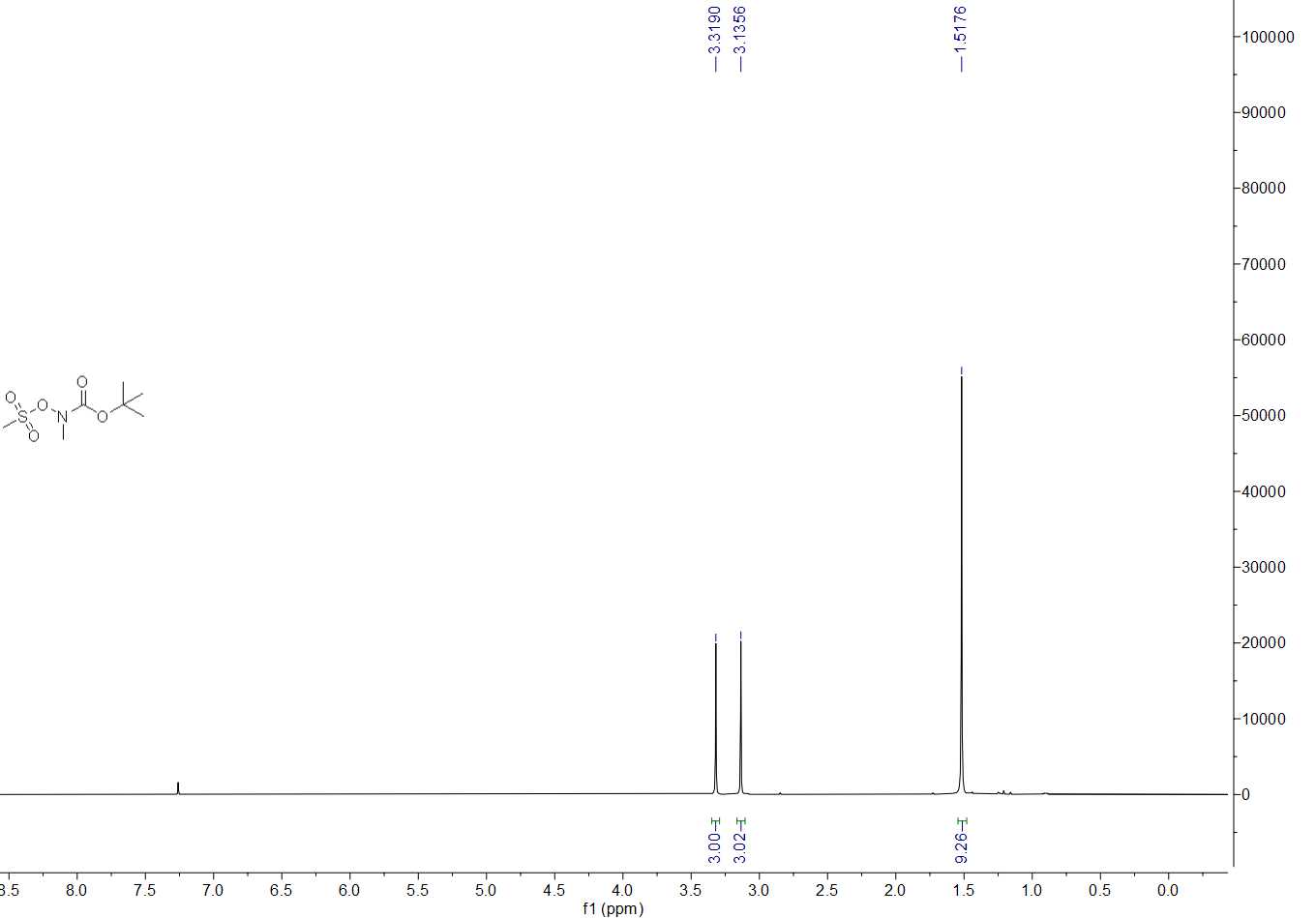


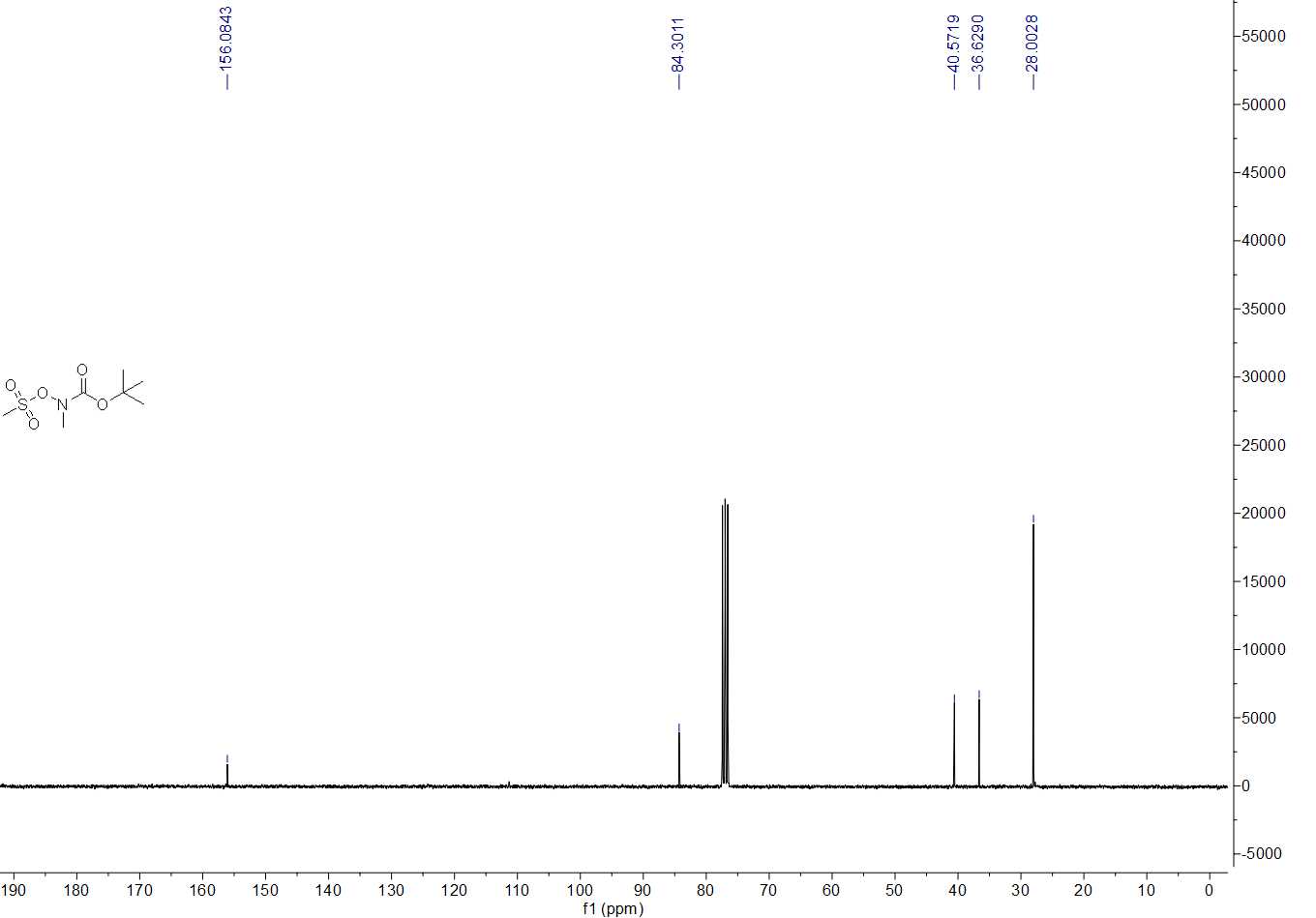


**Supplementary Fig 6.** ^1^H (upper part) and ^13^C NMR (lower part) of MsONBocMe


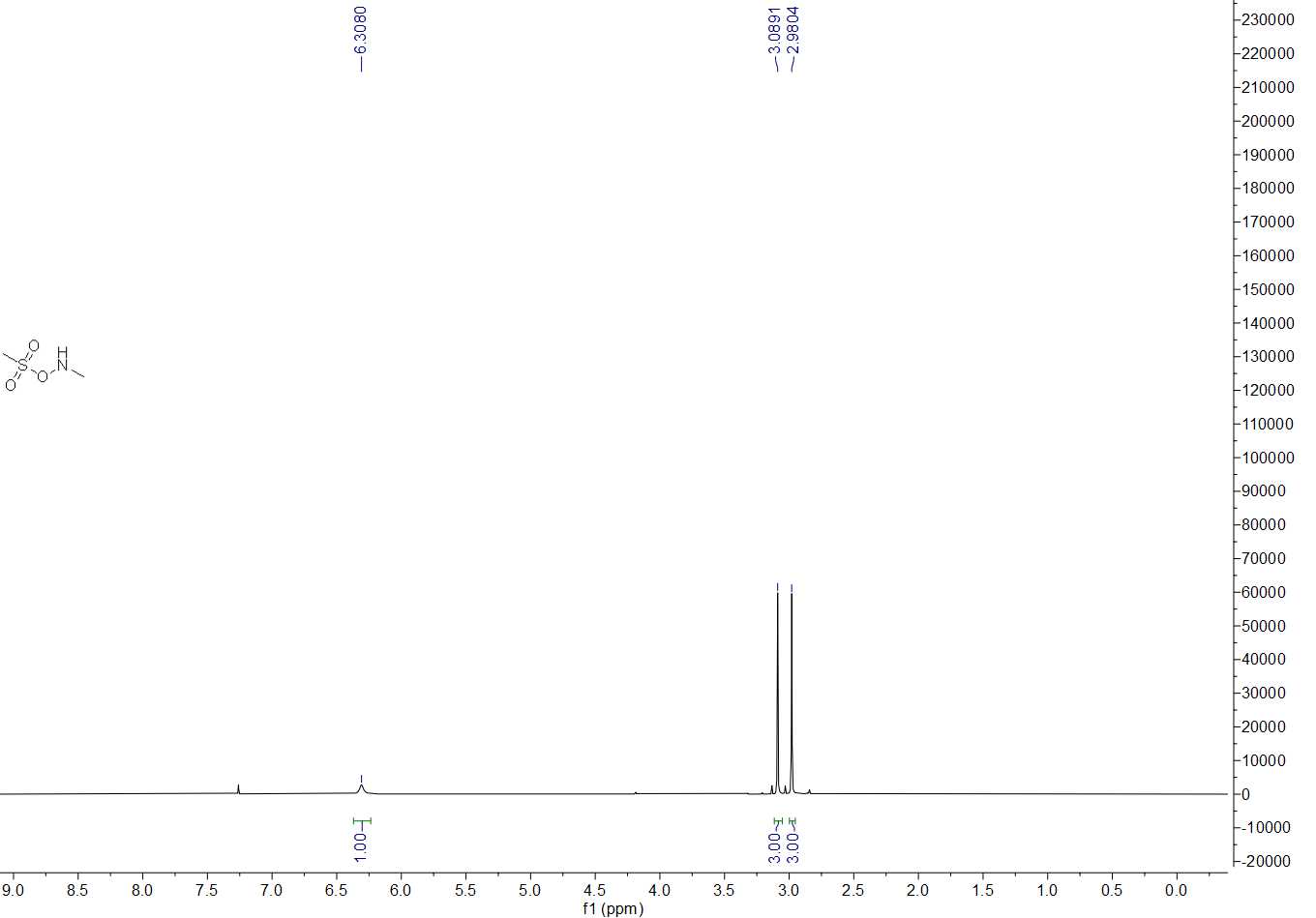


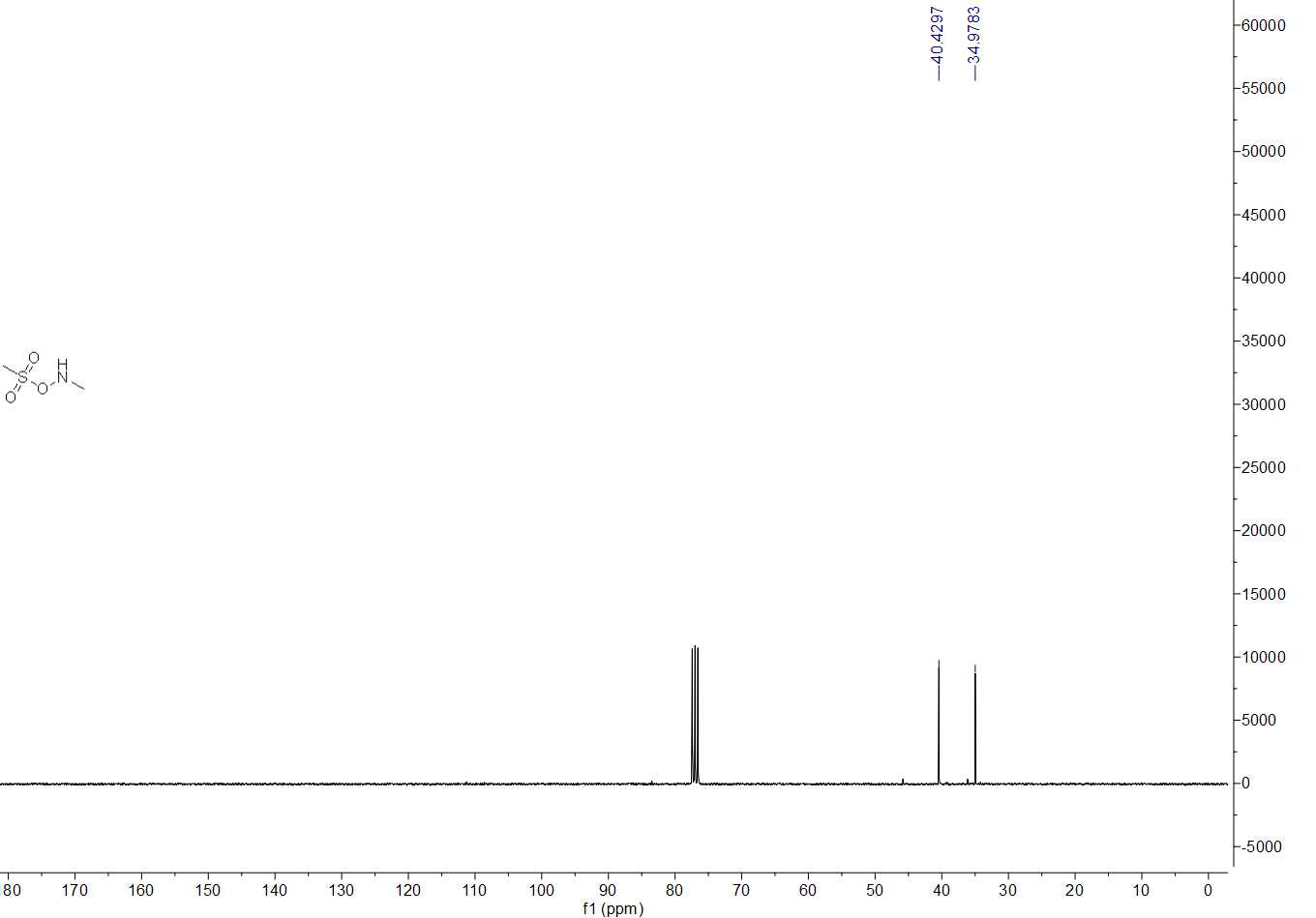


**Supplementary Fig 7.** ^1^H (upper part) and ^13^C NMR (lower part) of **2b**


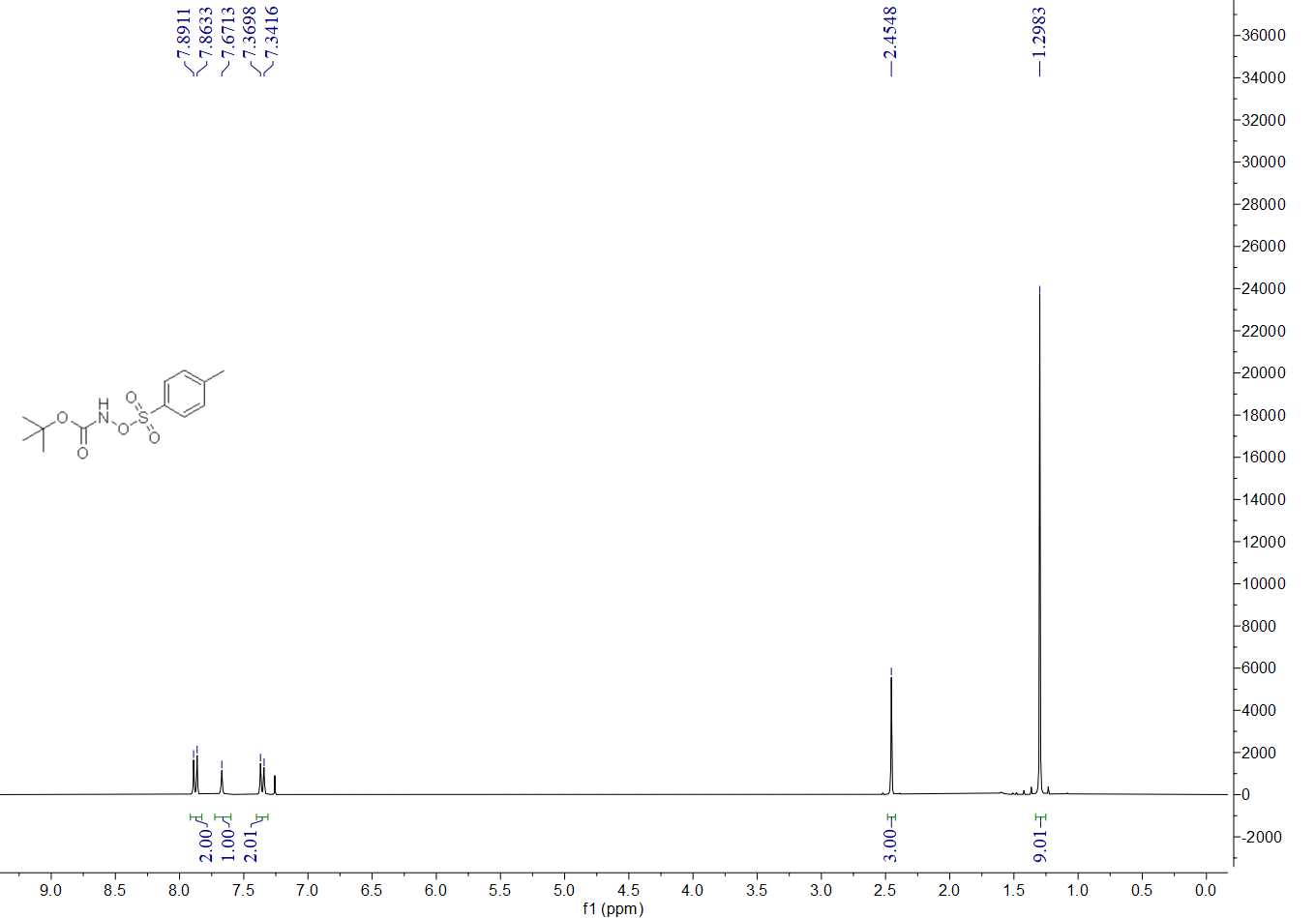


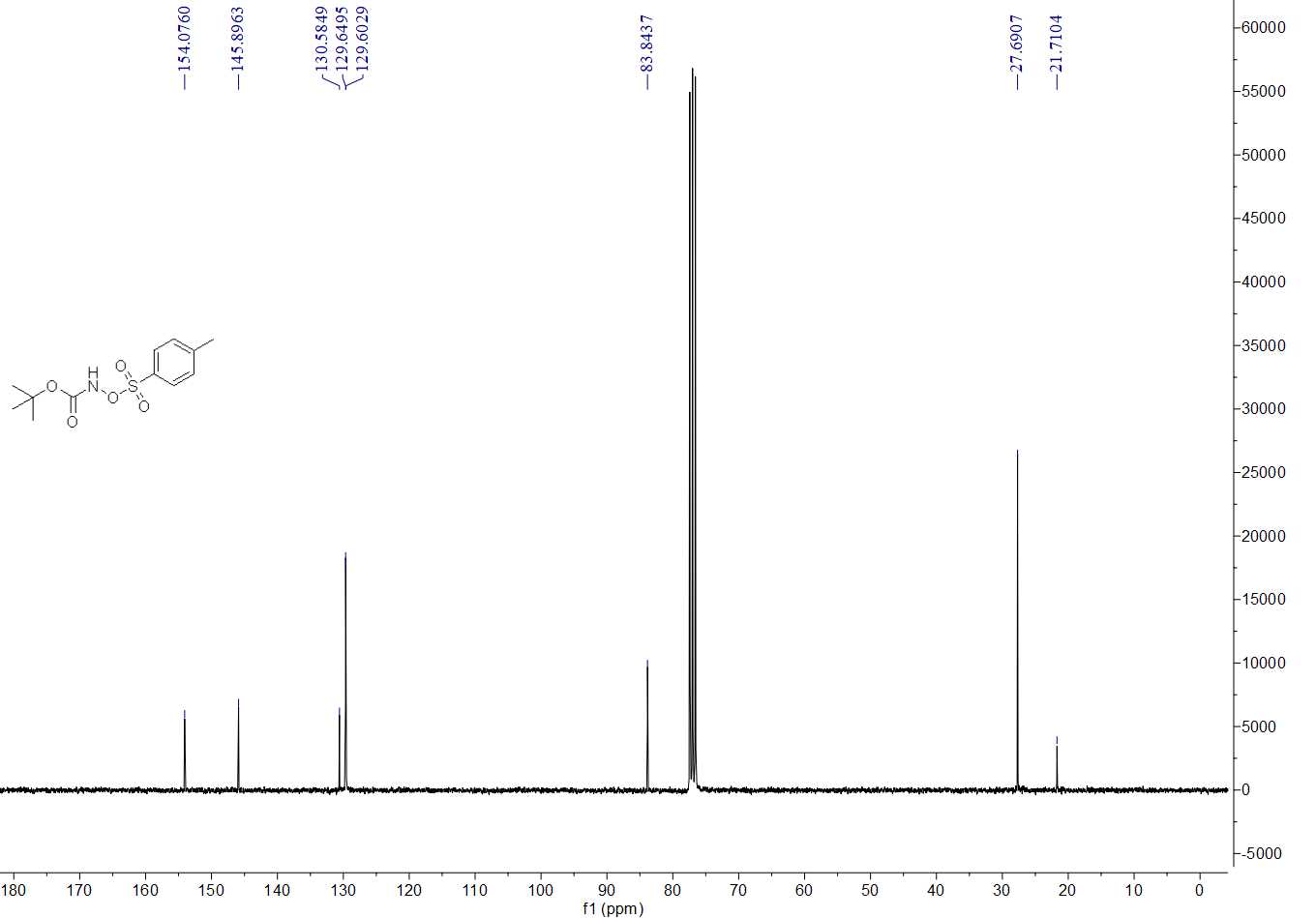


**Supplementary Fig 8.** ^1^H (upper part) and ^13^C NMR (lower part) of **5a**.


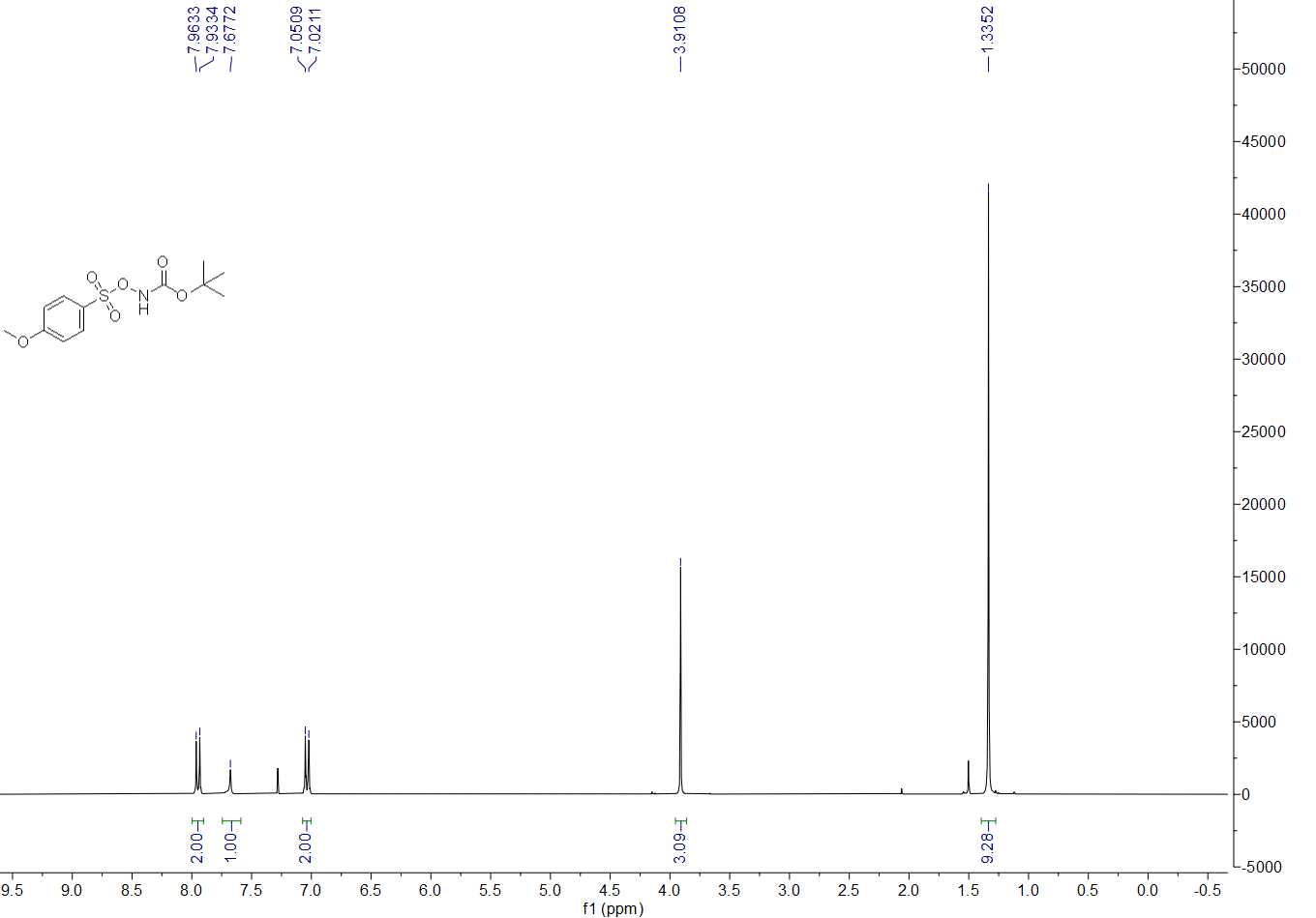


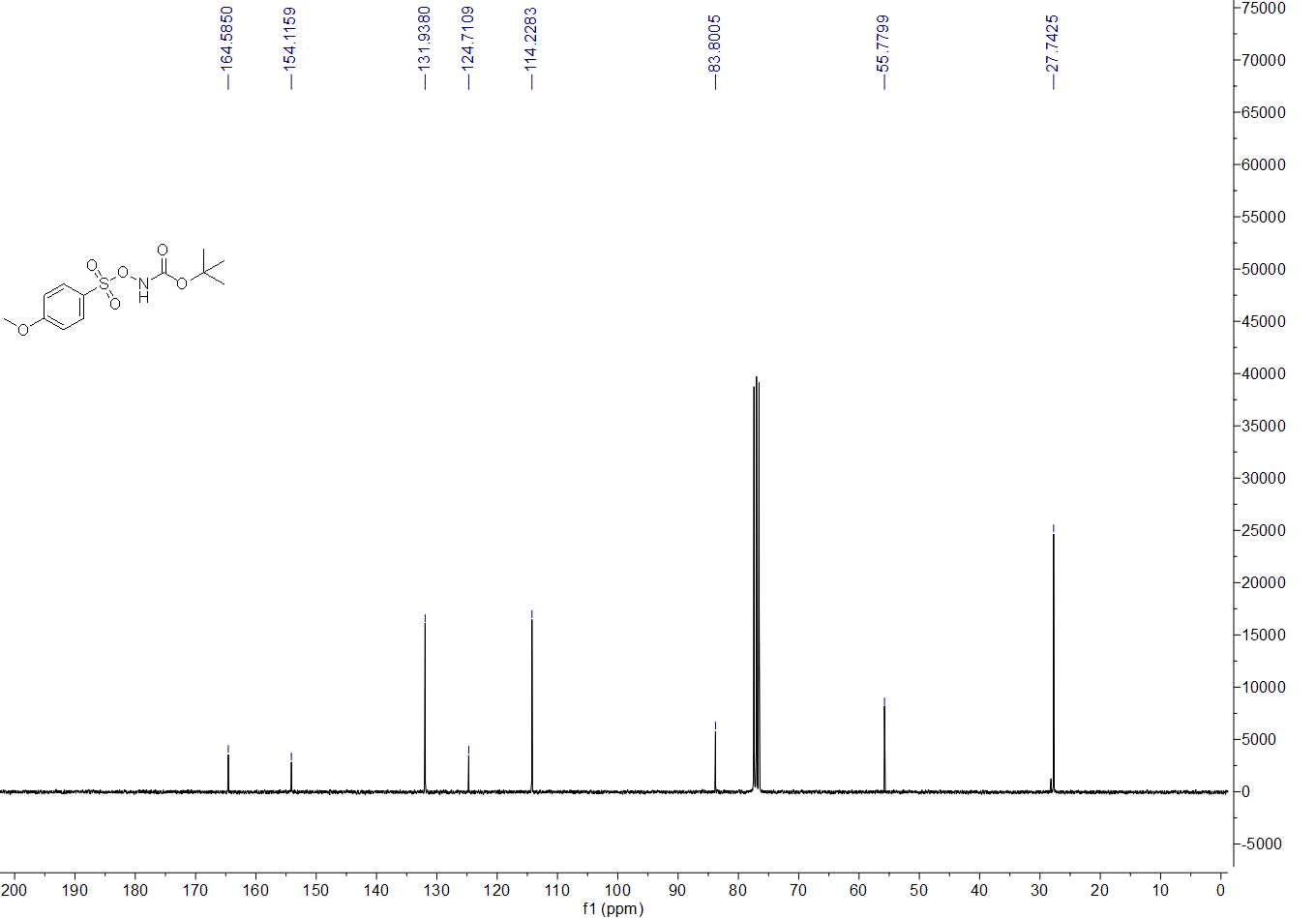


**Supplementary Fig 9.** ^1^H (upper part) and ^13^C NMR (lower part) of **5b**.


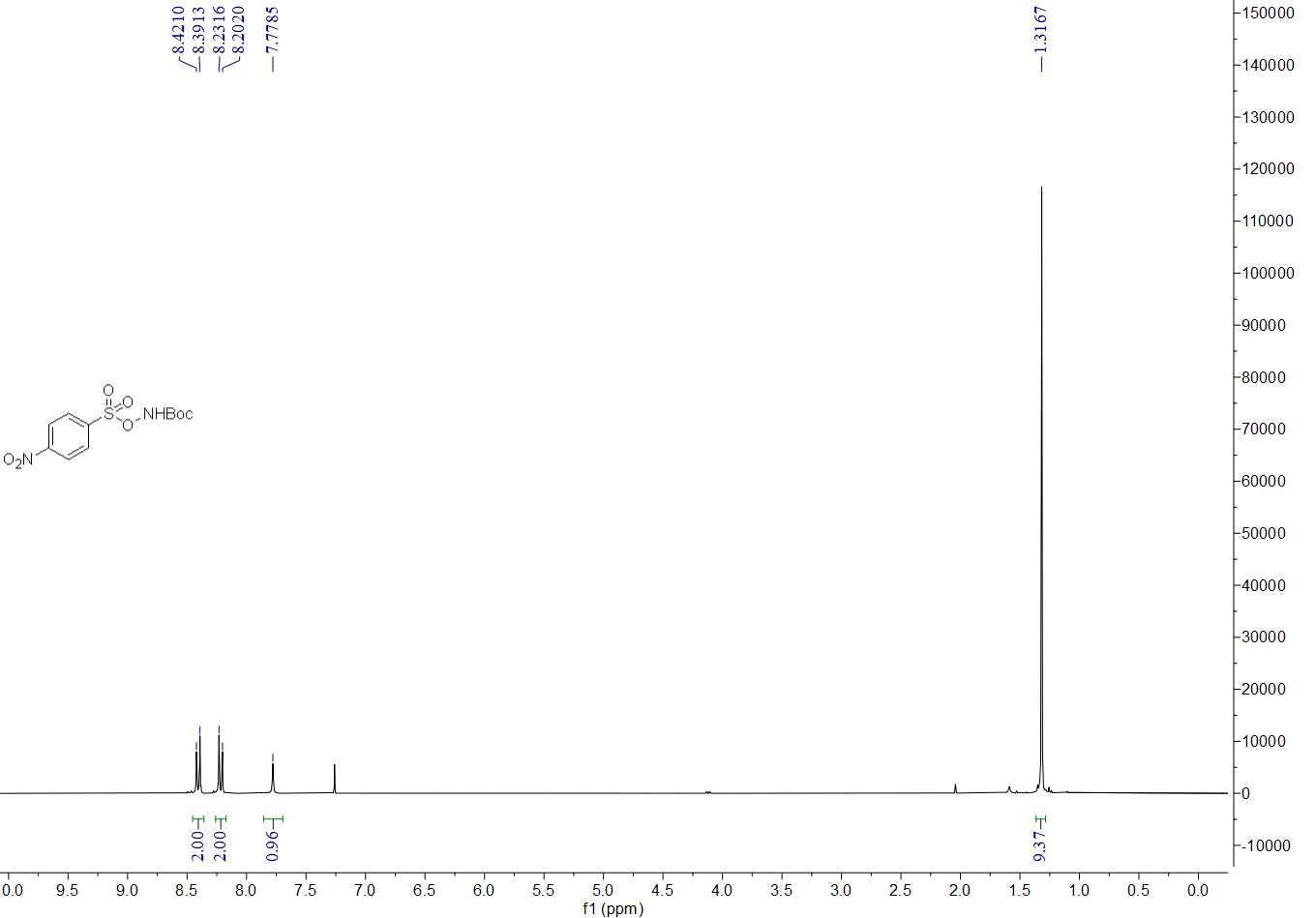


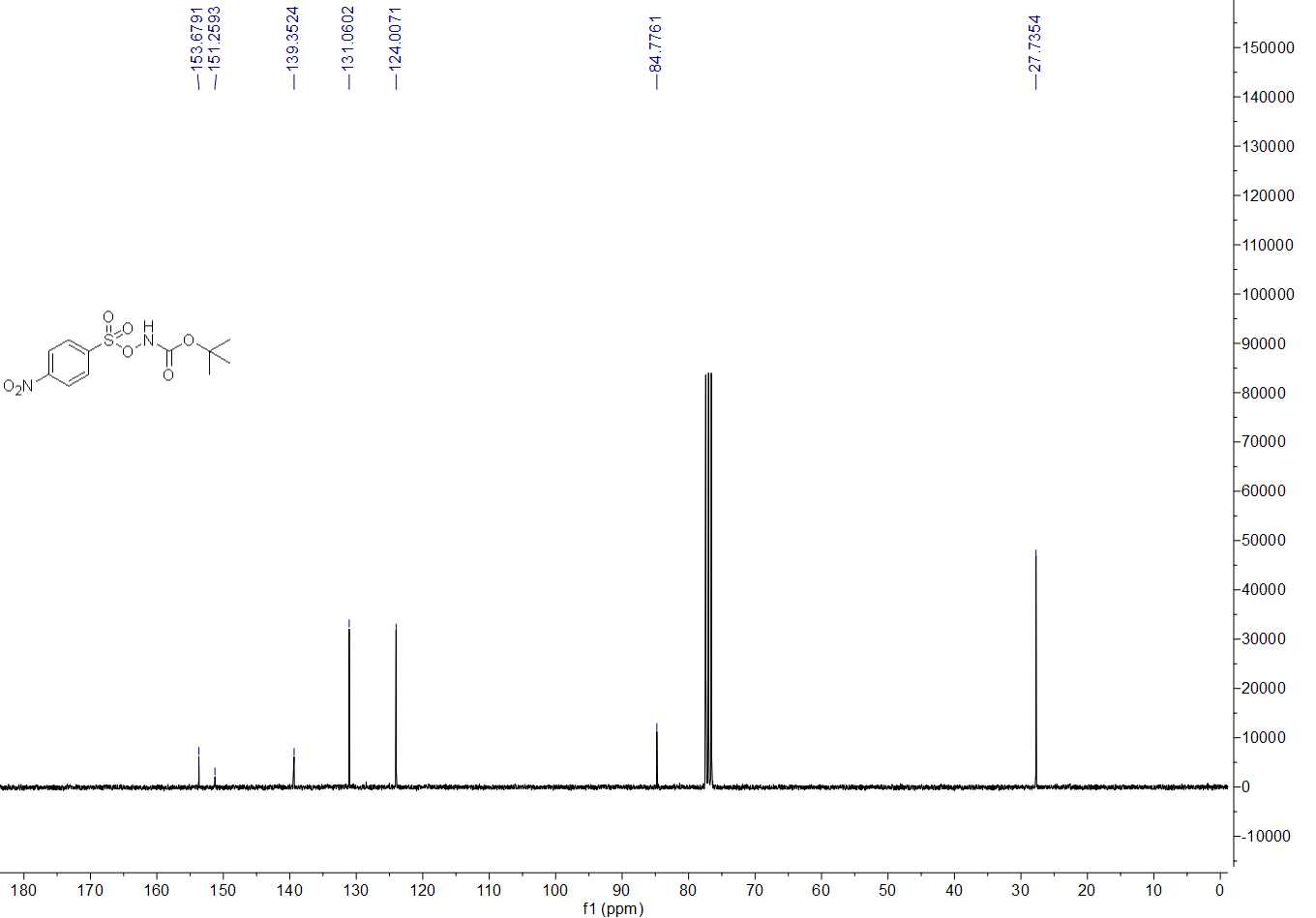


**Supplementary Fig 10.** ^1^H (upper part) and ^13^C NMR (lower part) of **5c**.


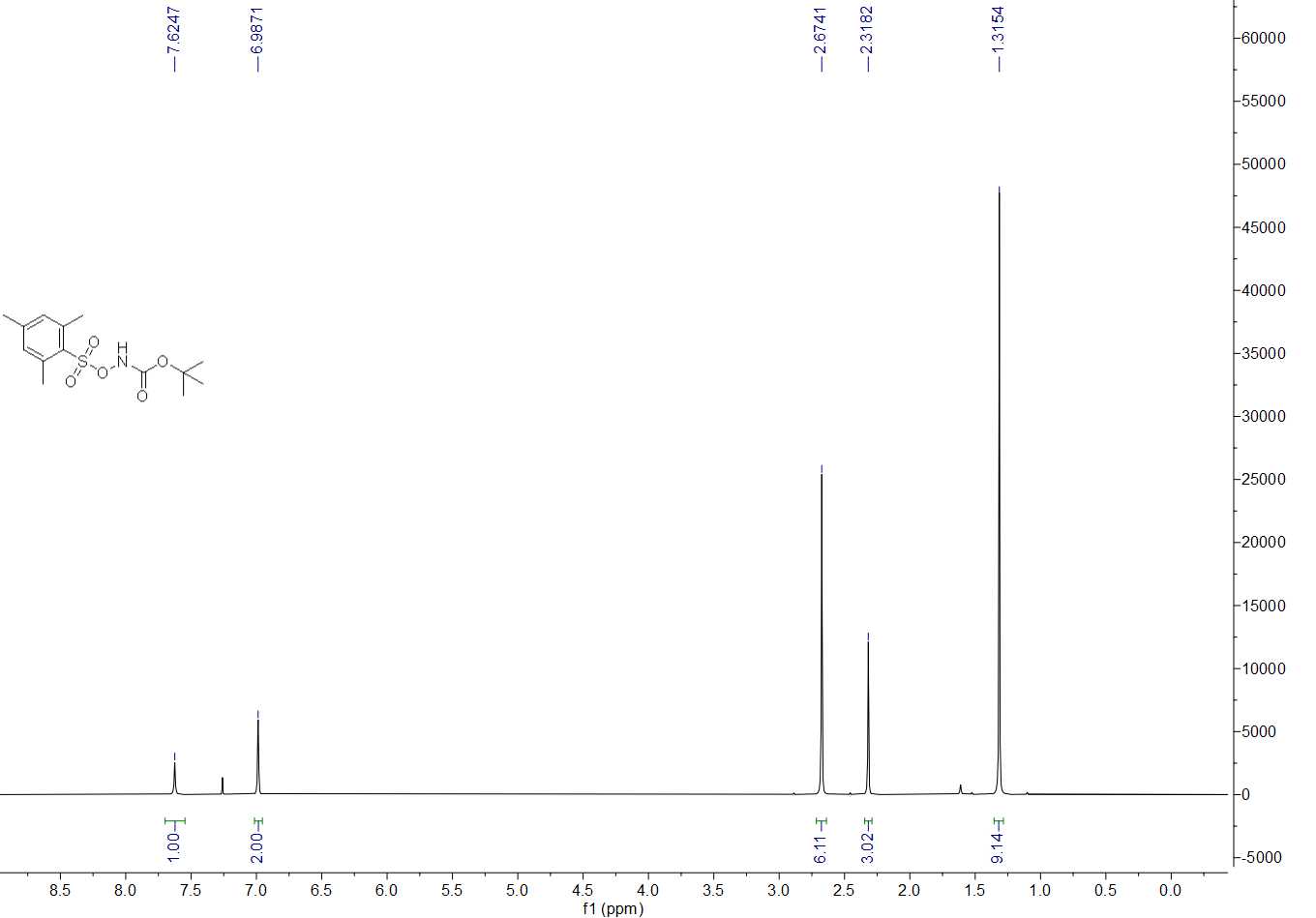


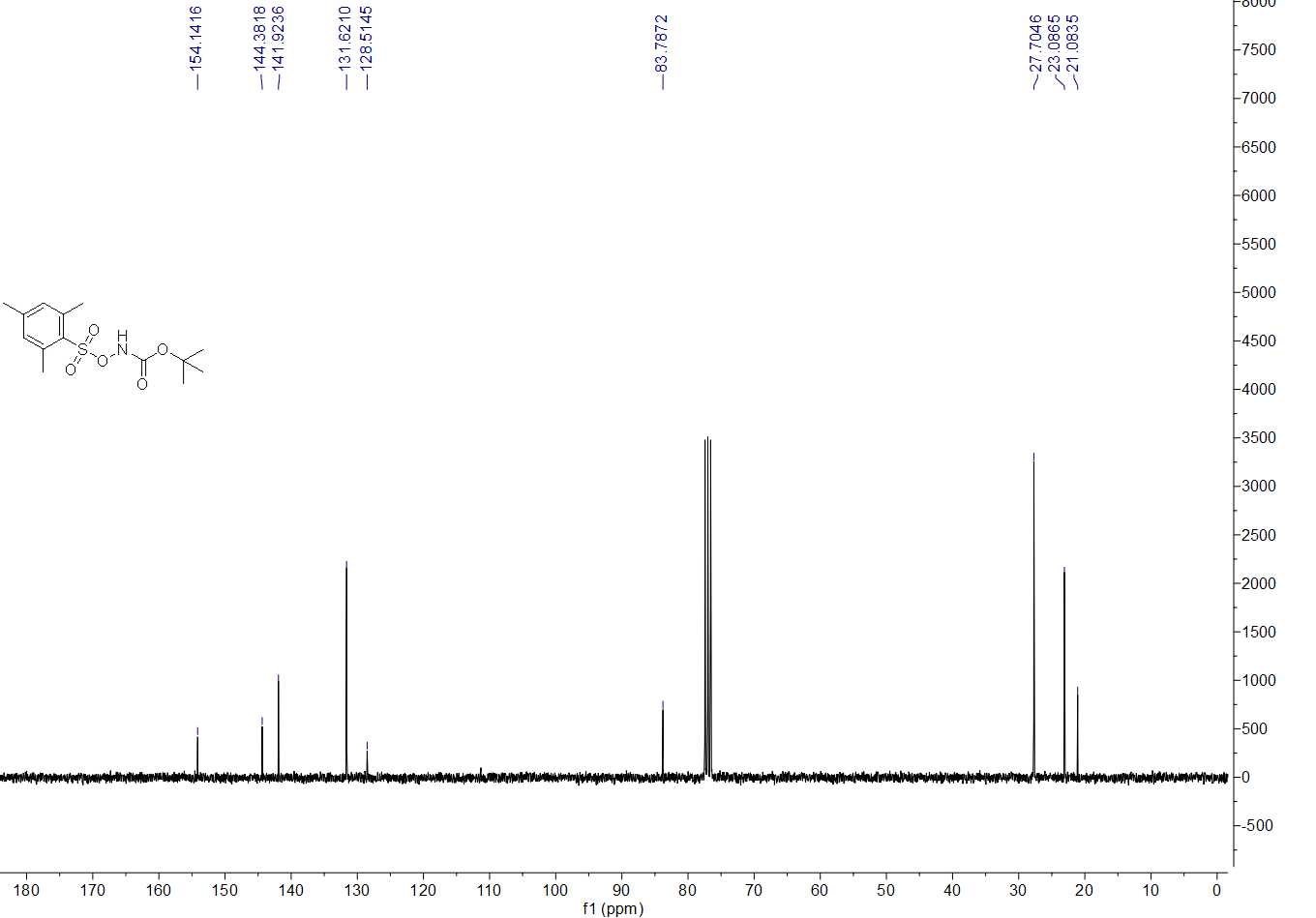


**Supplementary Fig 11.** ^1^H (upper part) and ^13^C NMR (lower part) of **5d**.


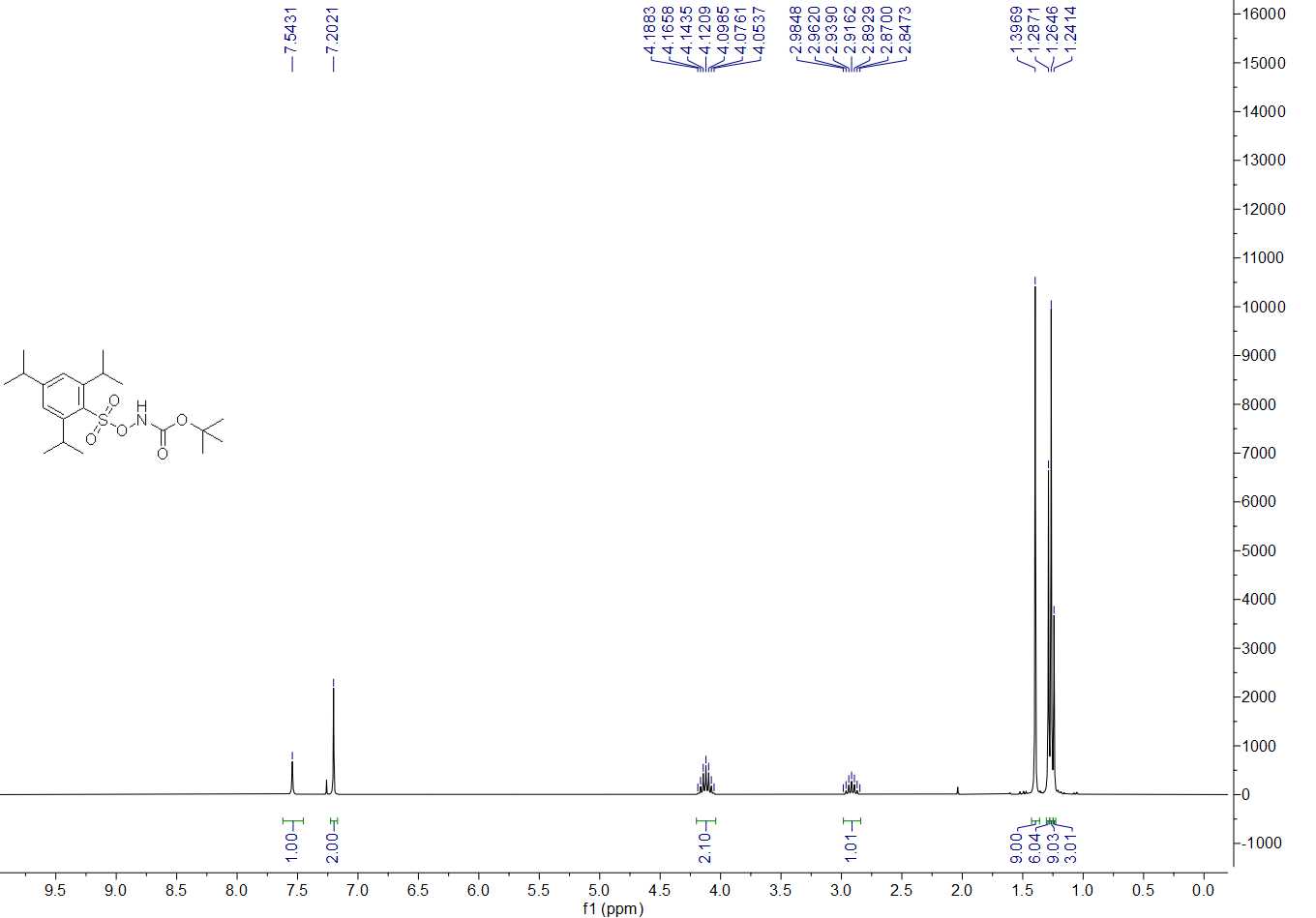


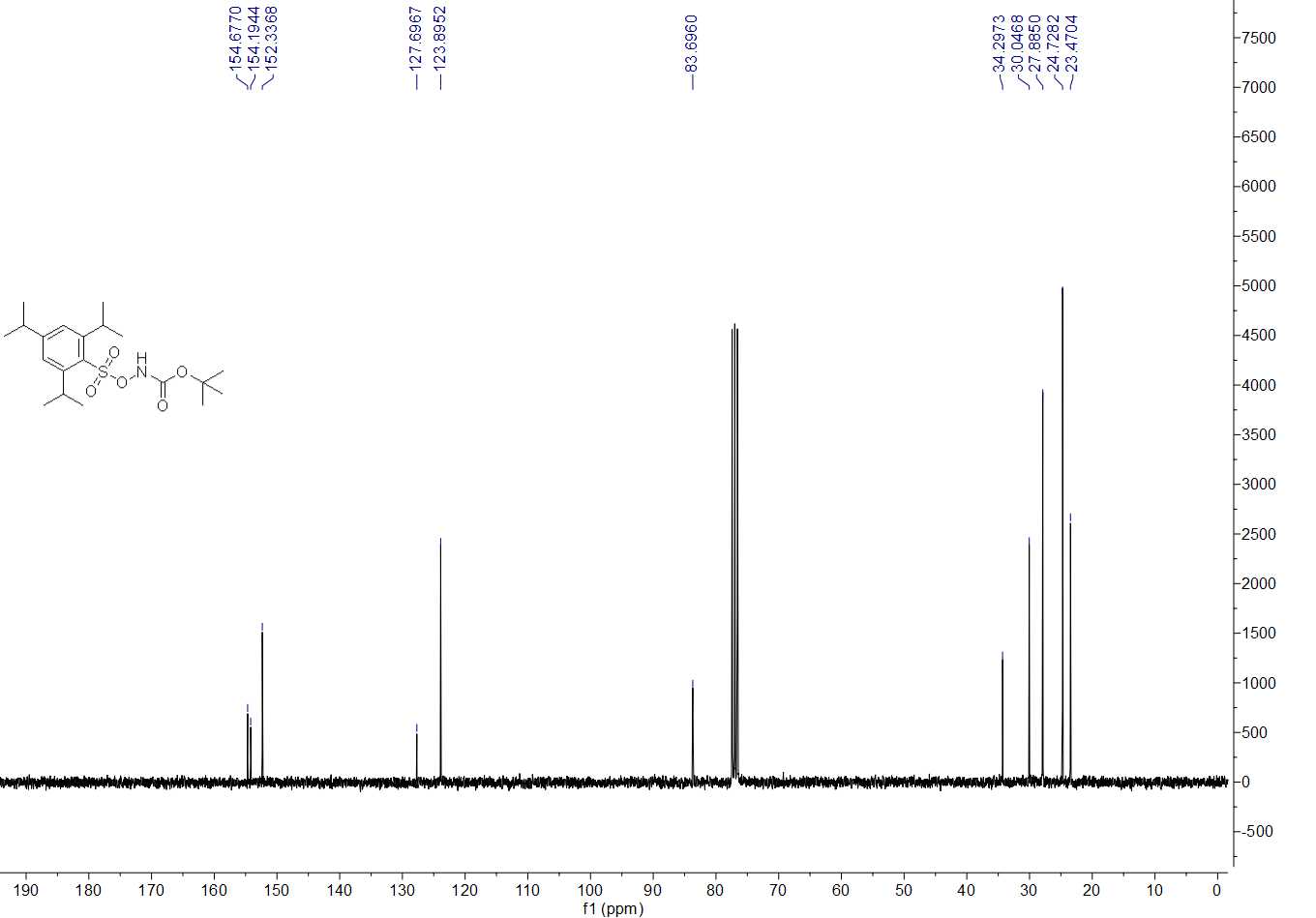


**Supplementary Fig 12.** ^1^H (upper part) and ^13^C NMR (lower part) of **5e**.


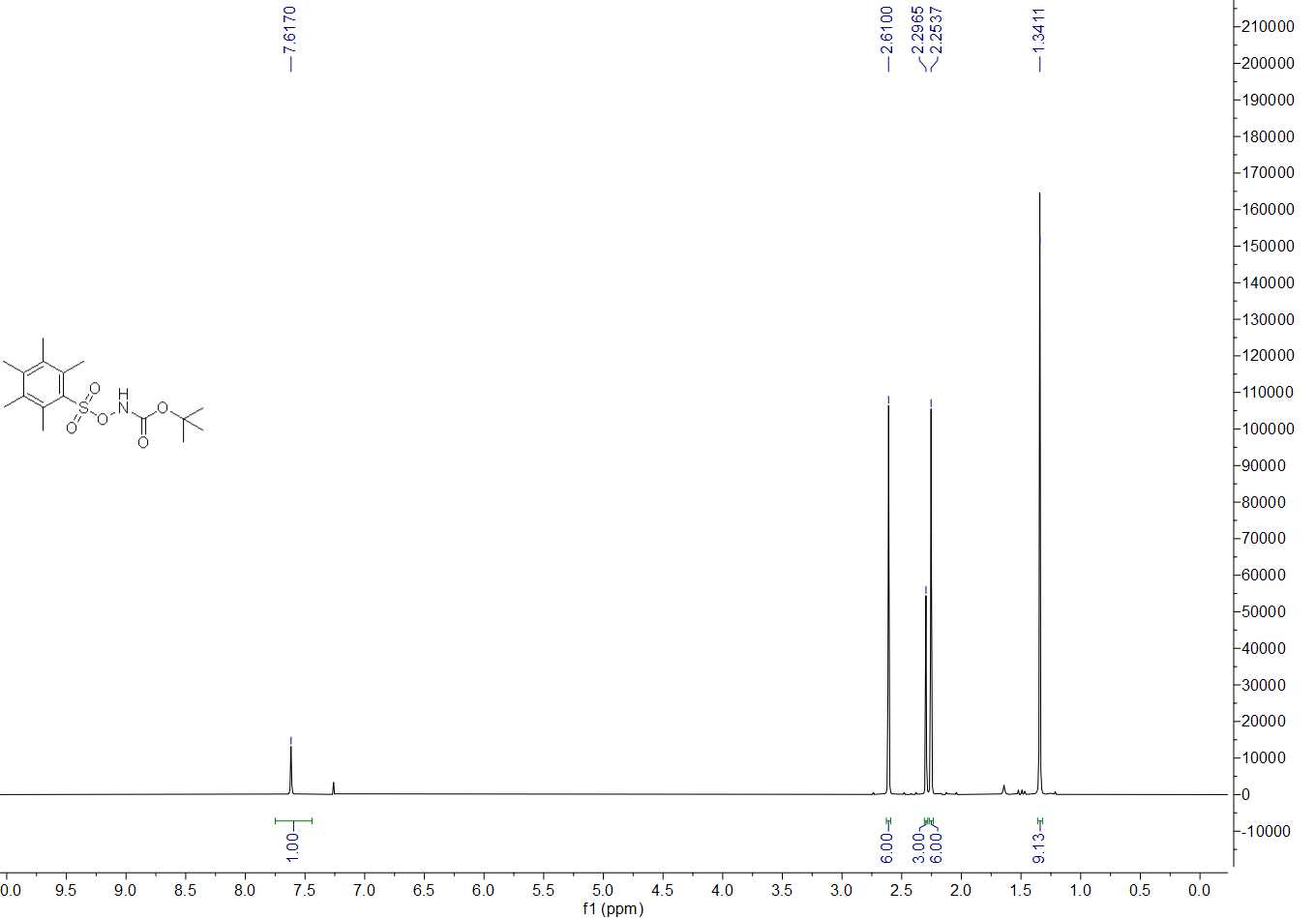


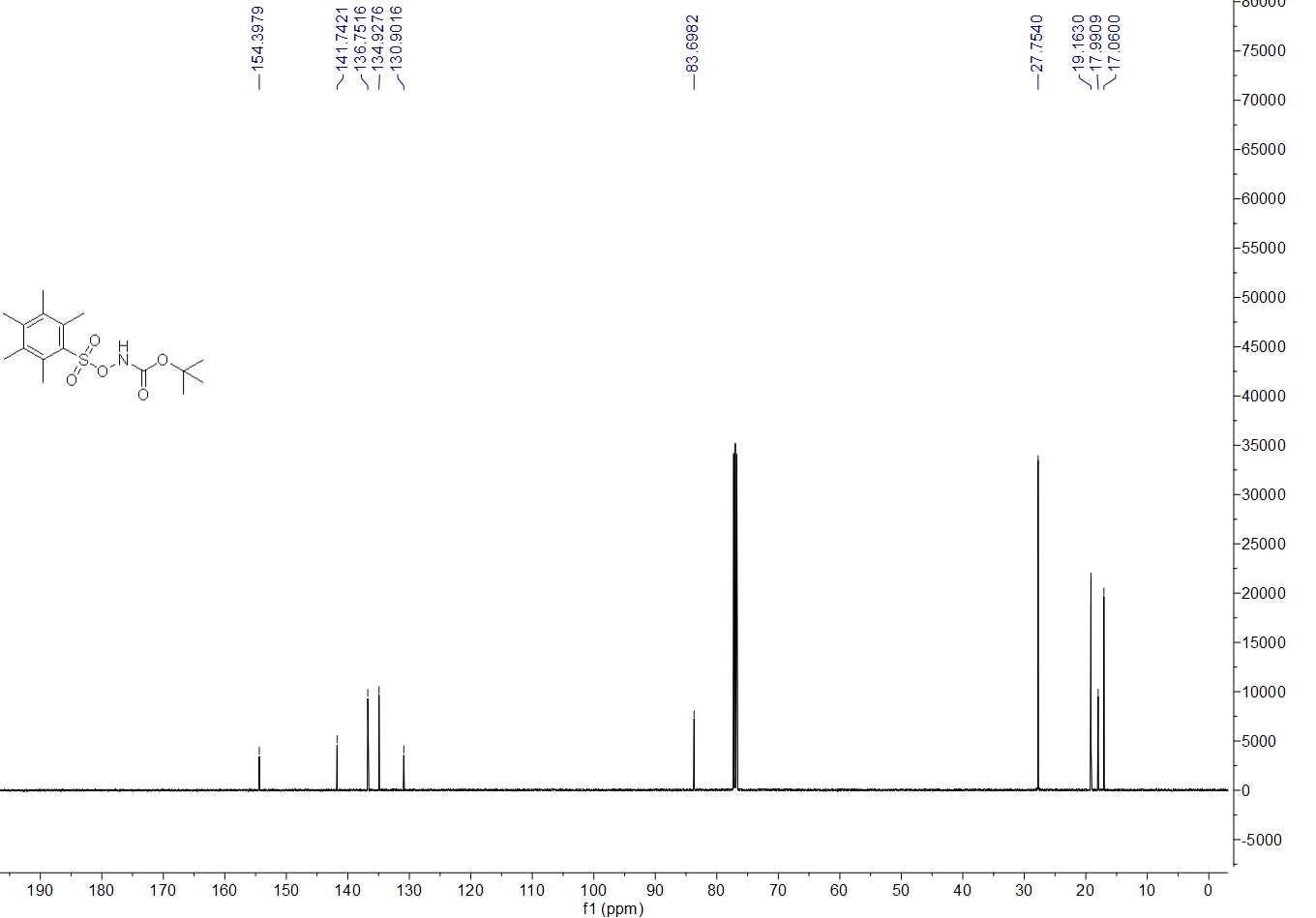


**Supplementary Fig 13.** ^1^H (upper part) and ^13^C NMR (lower part) of **5f**.


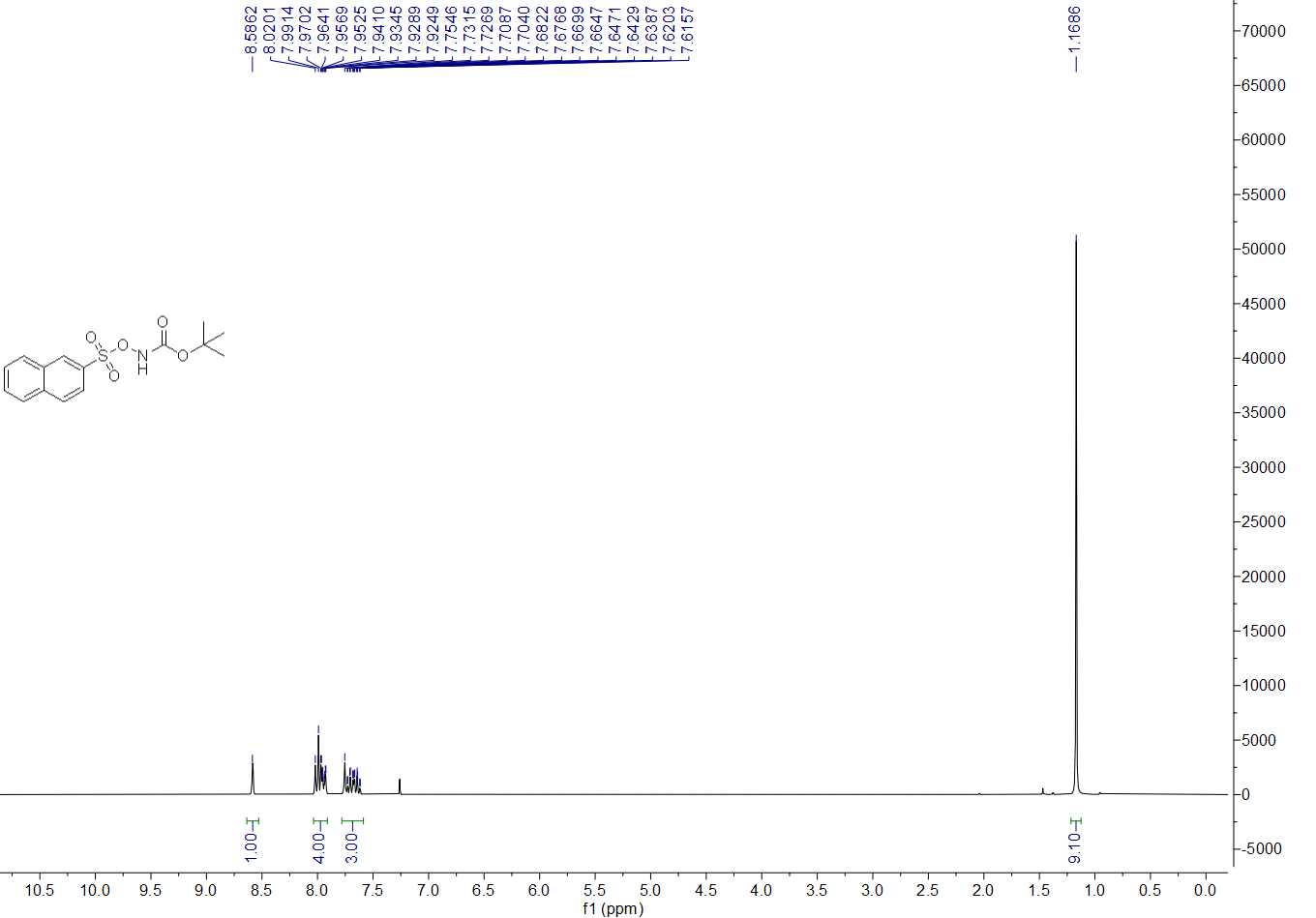


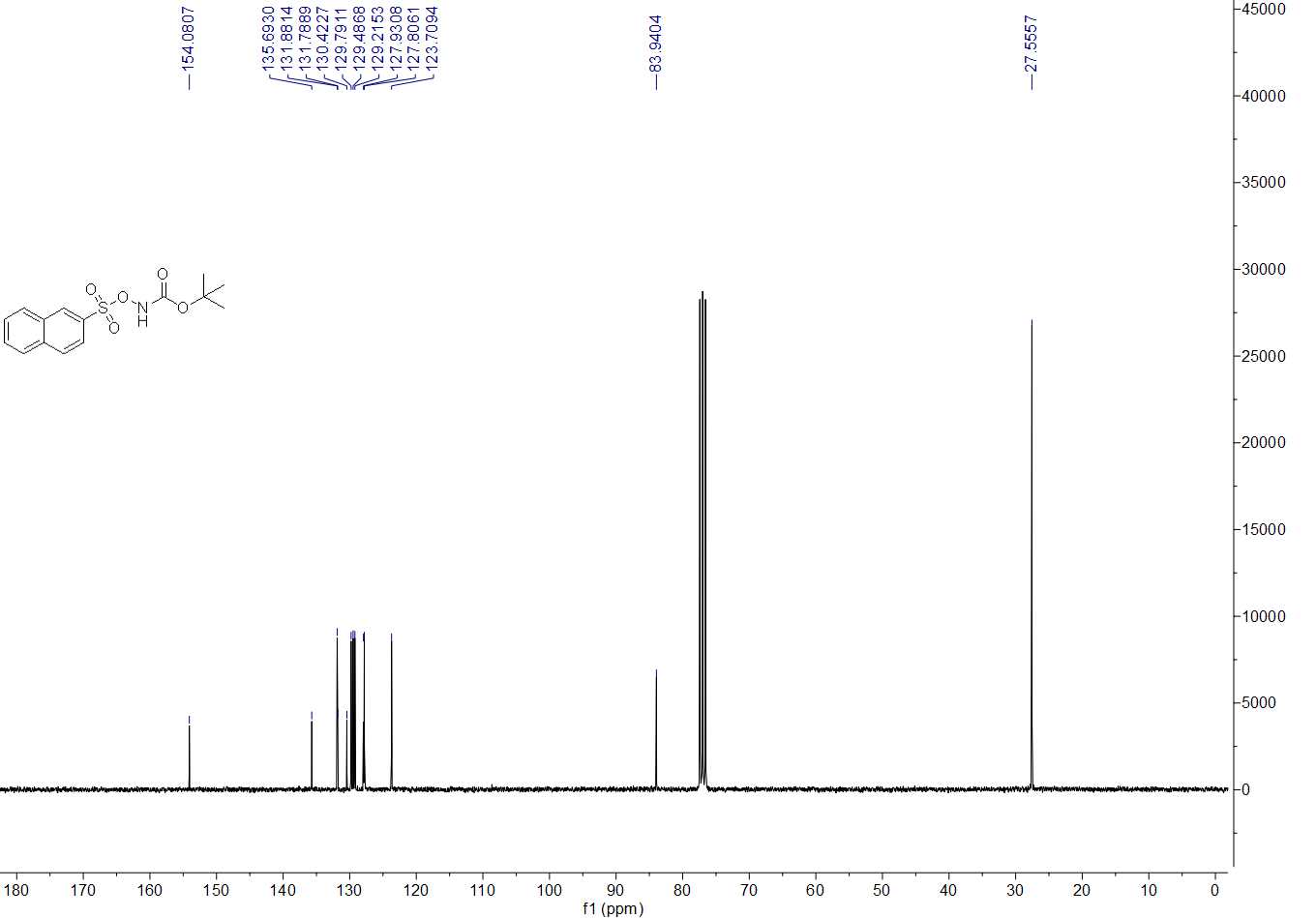


**Supplementary Fig 14.** ^1^H (upper part) and ^13^C NMR (lower part) of **5g**.


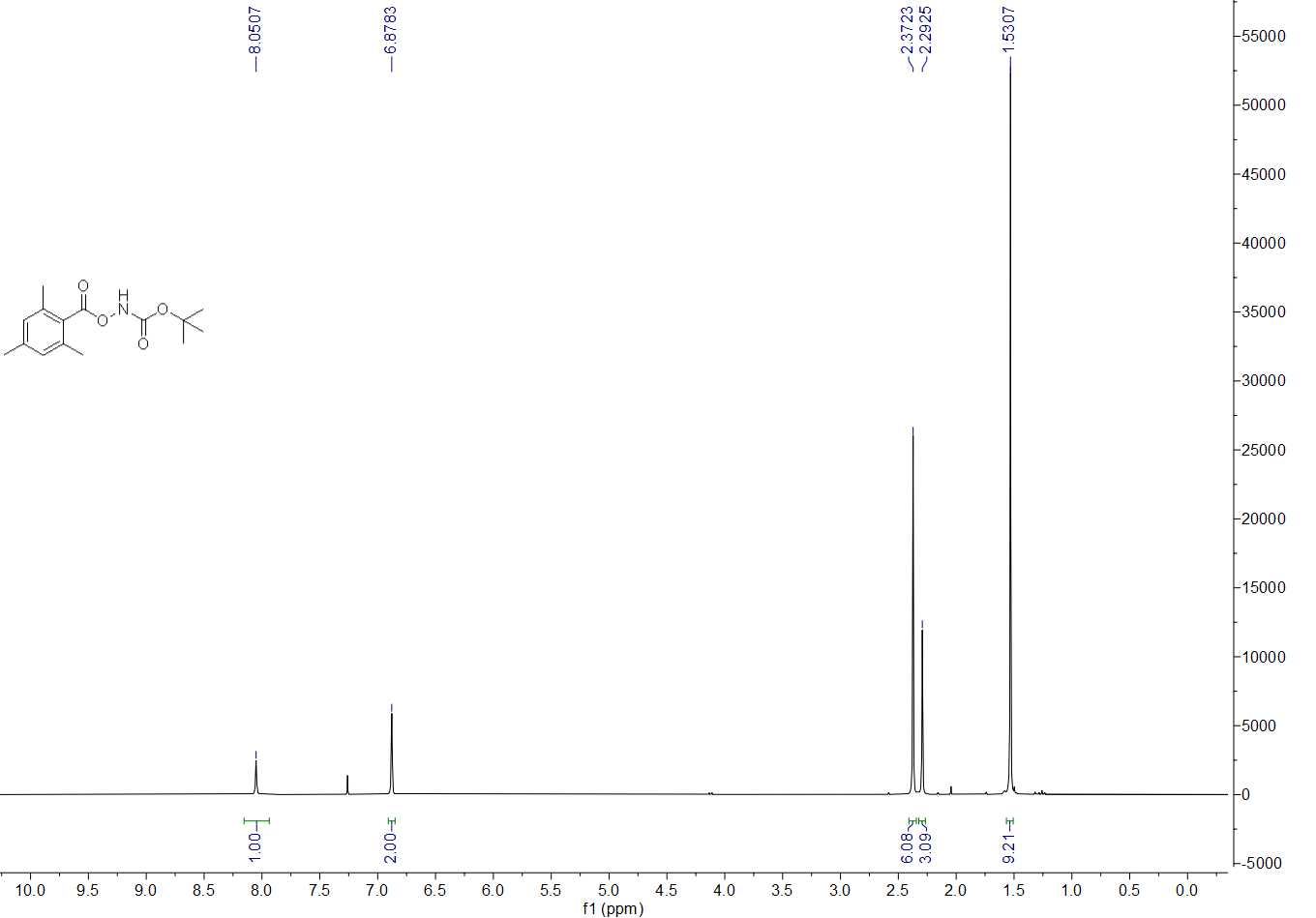


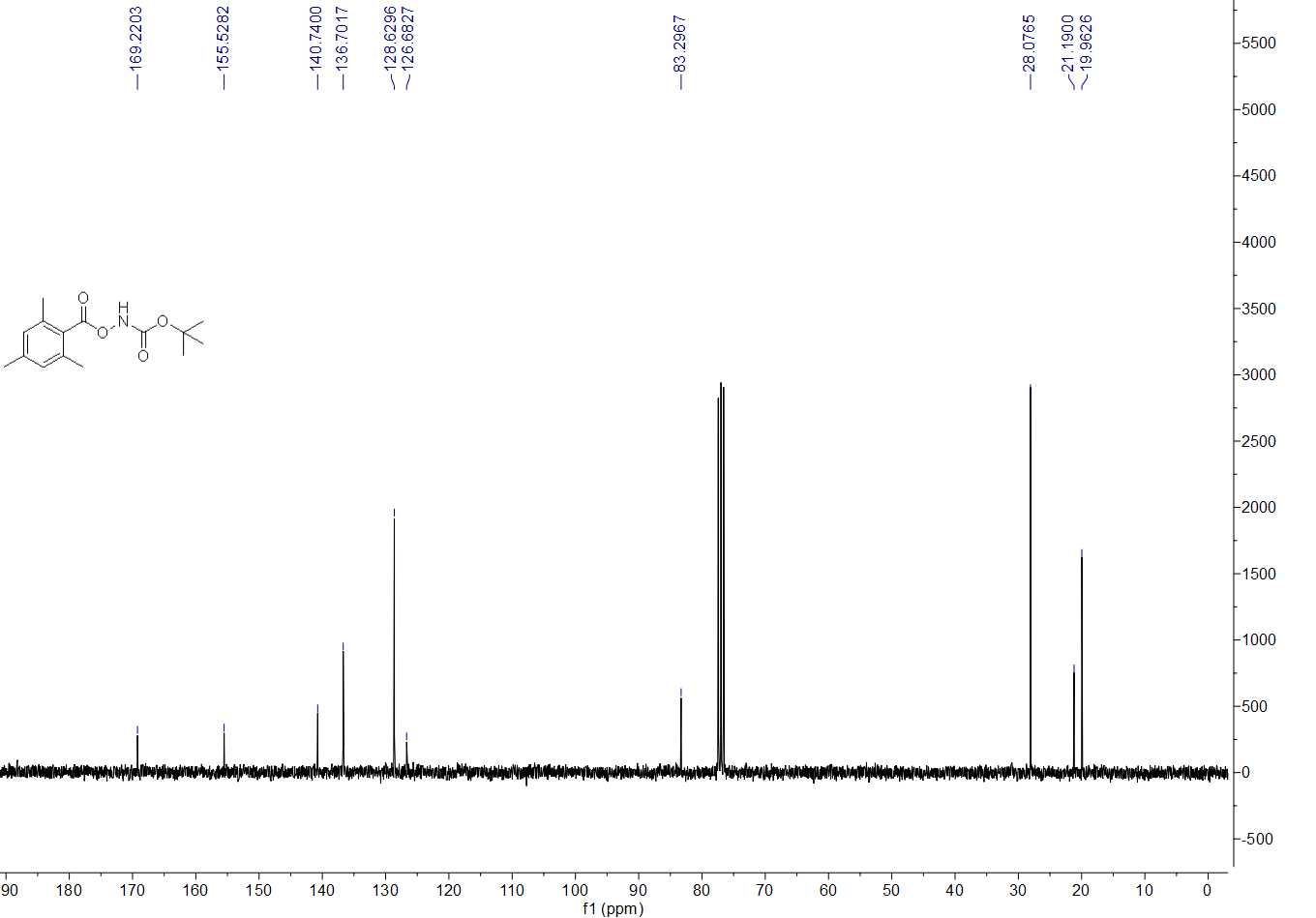


**Supplementary Fig 15.** ^1^H (upper part) and ^13^C NMR (lower part) of **5h**.


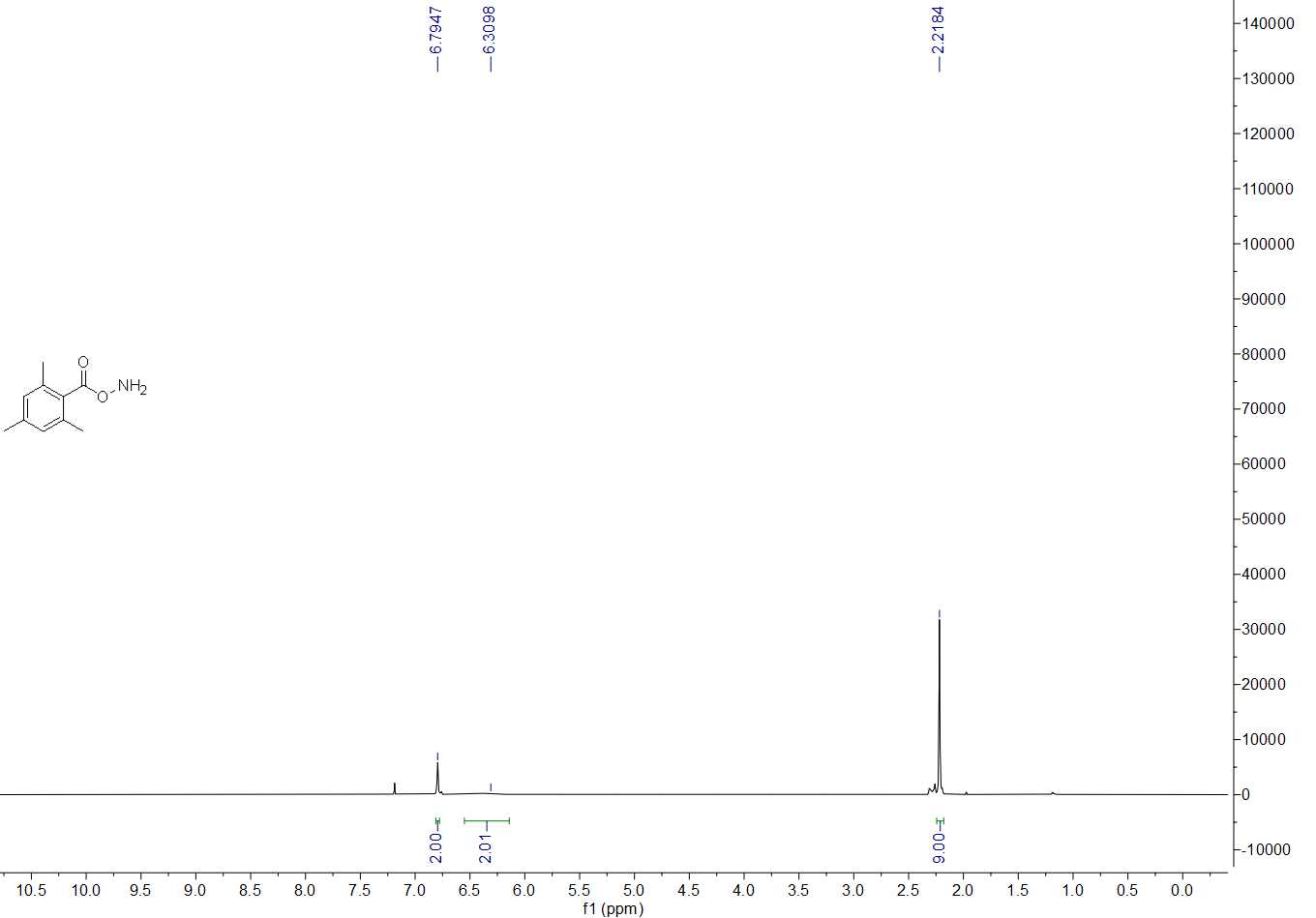


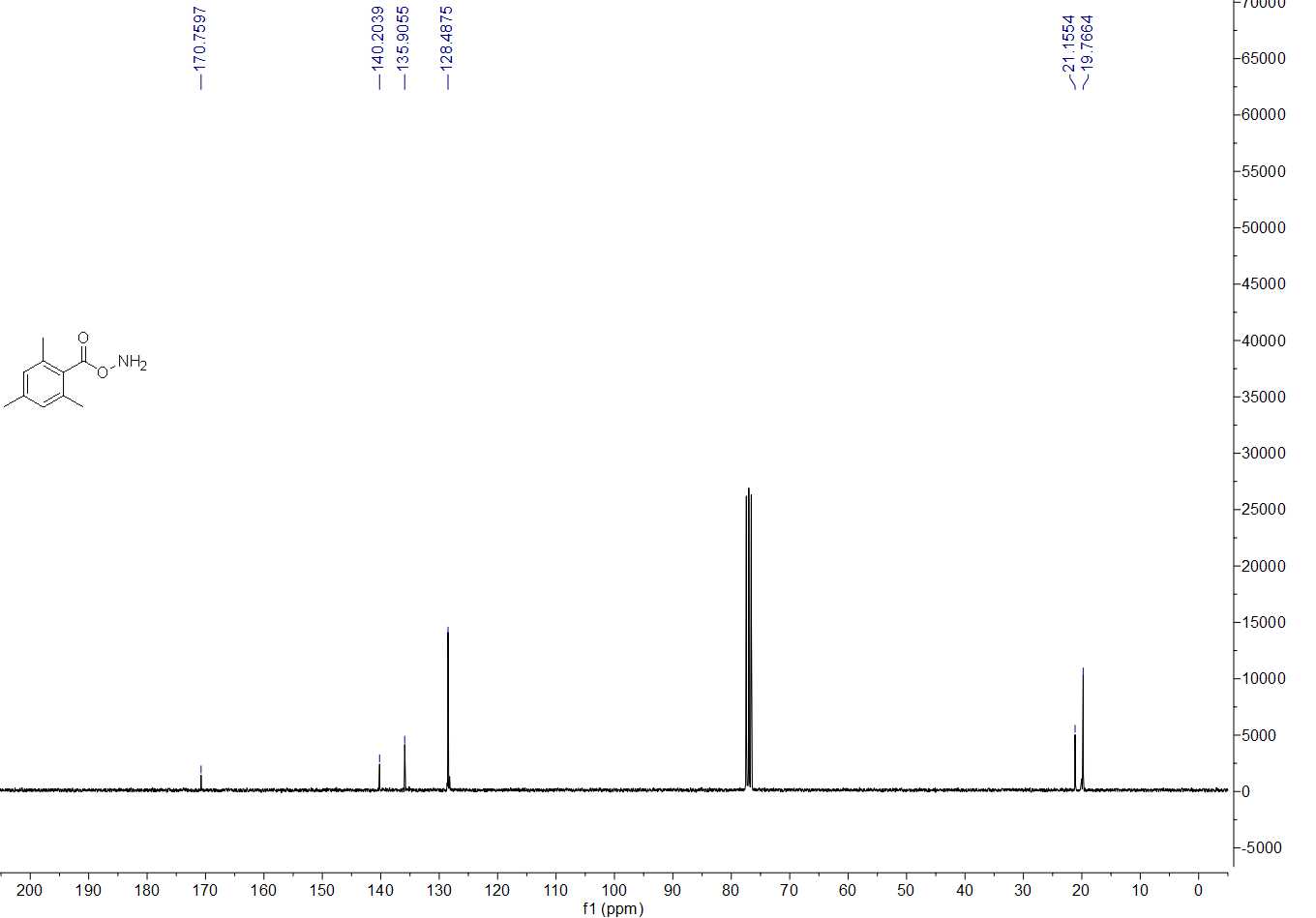


**Supplementary Fig 16.** ^1^H (upper part) and ^13^C NMR (lower part) of **5i**.


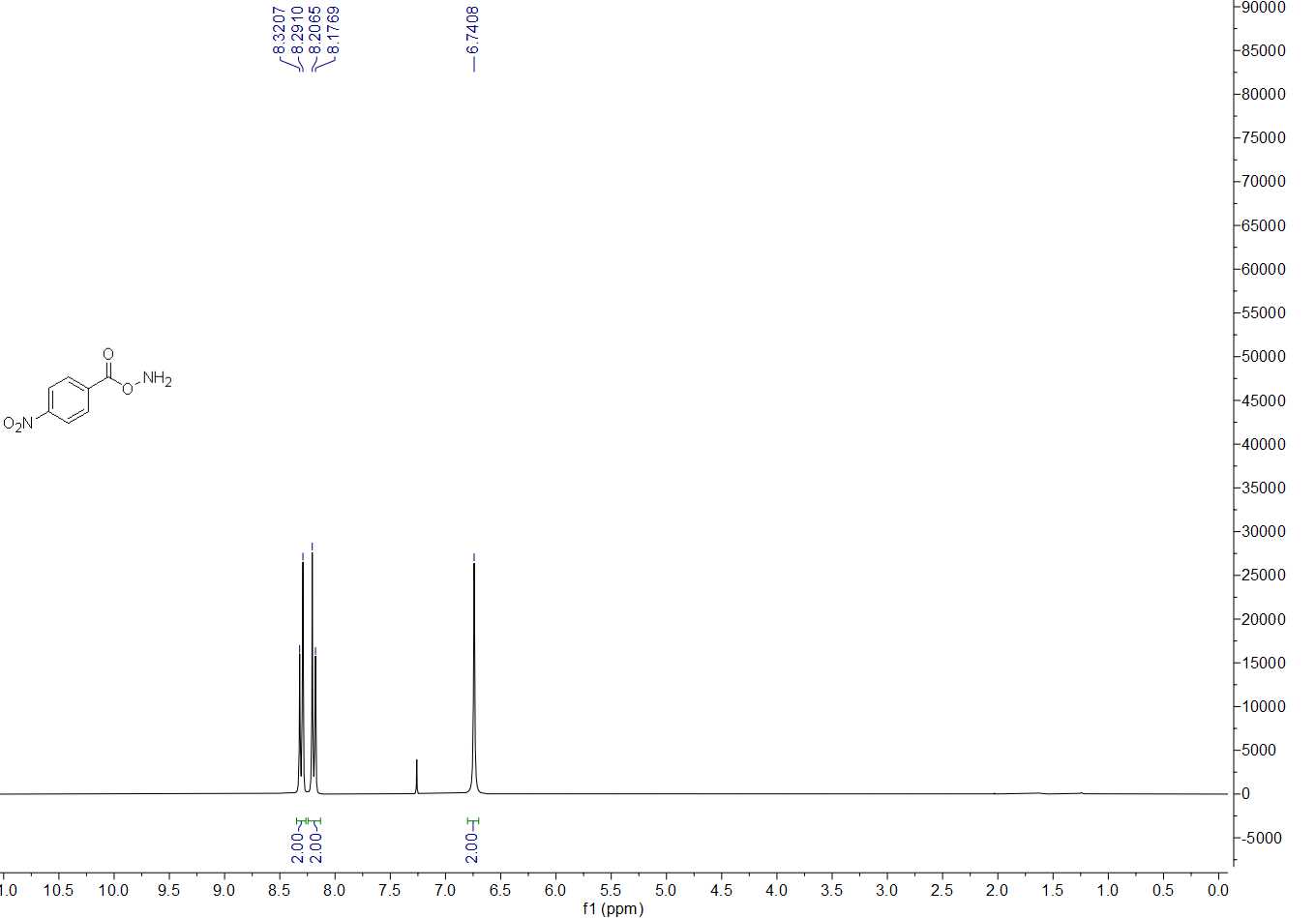


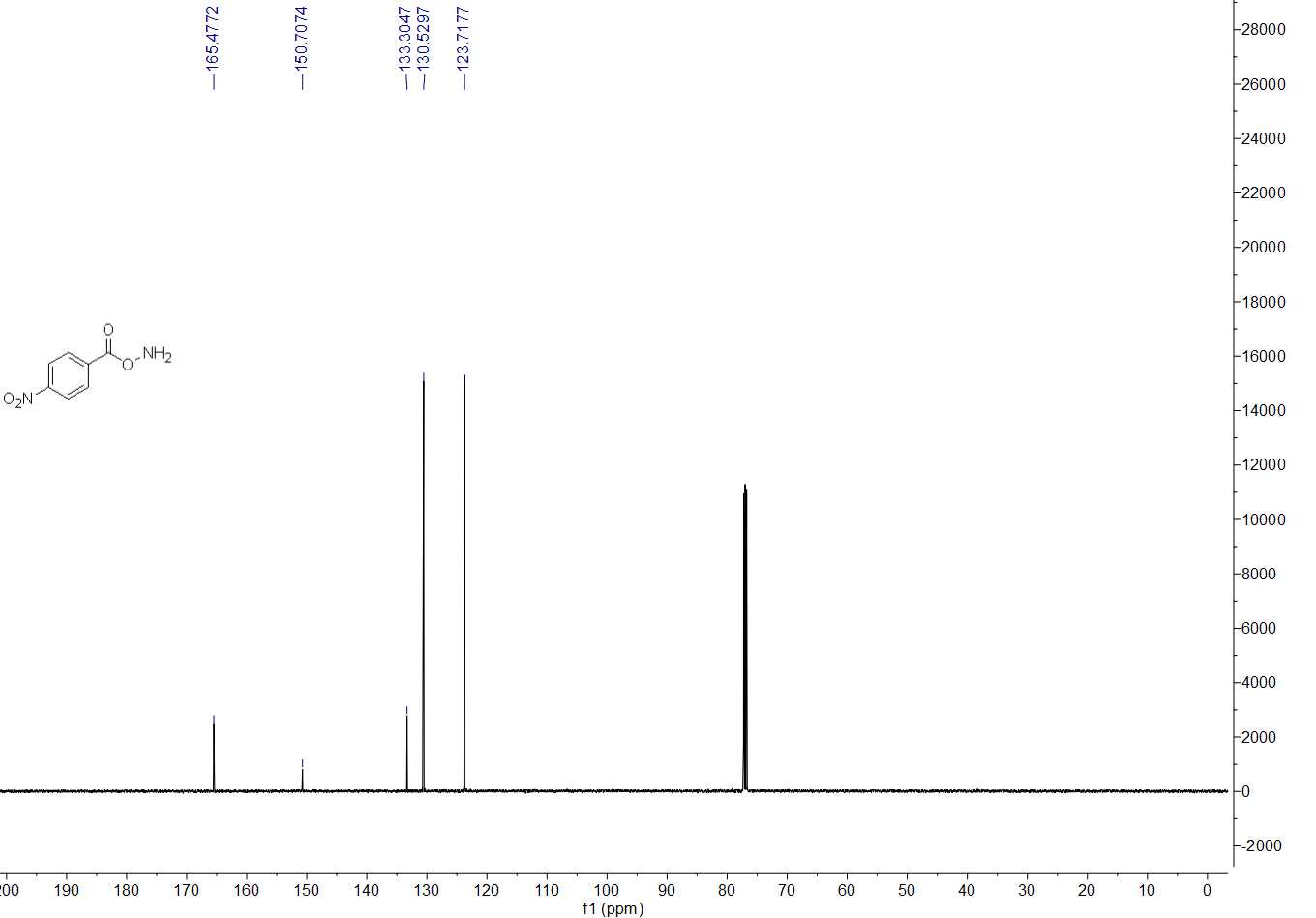


**Supplementary Fig 17.** ^1^H (upper part) and ^13^C NMR (lower part) of **5j**.


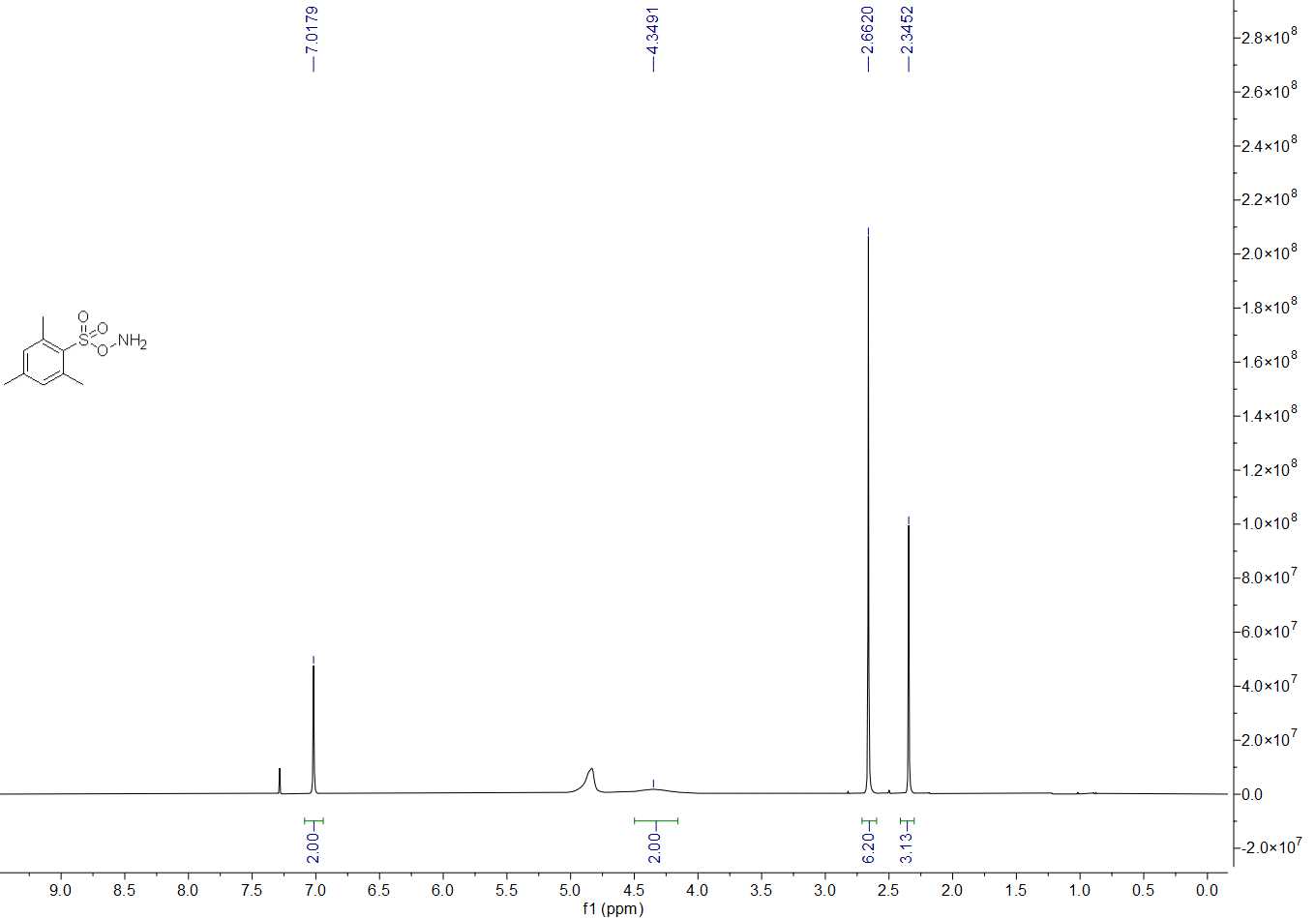


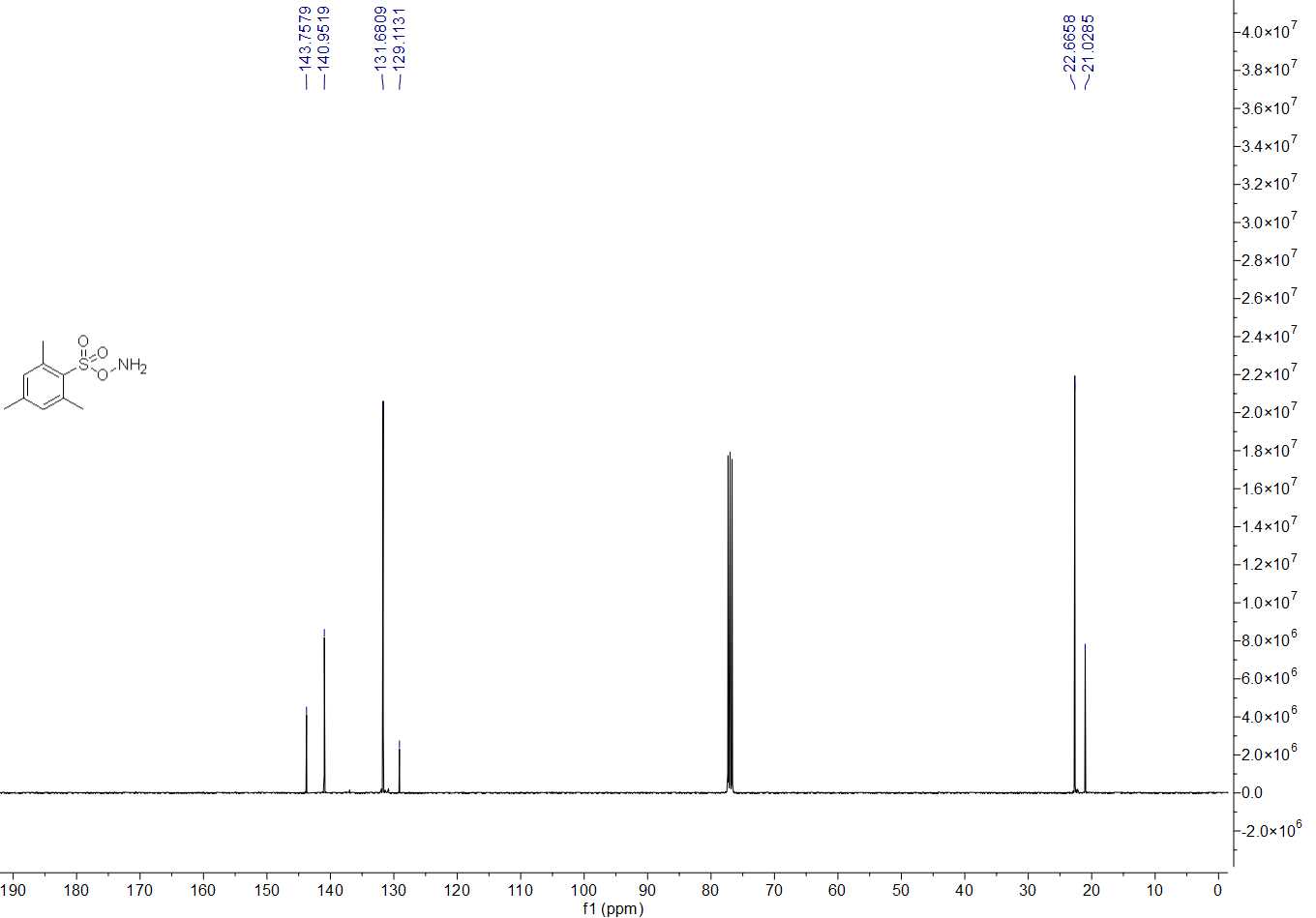


**Supplementary Fig 18.** ^1^H (upper part) and ^13^C NMR (lower part) of MSH (**5m**).


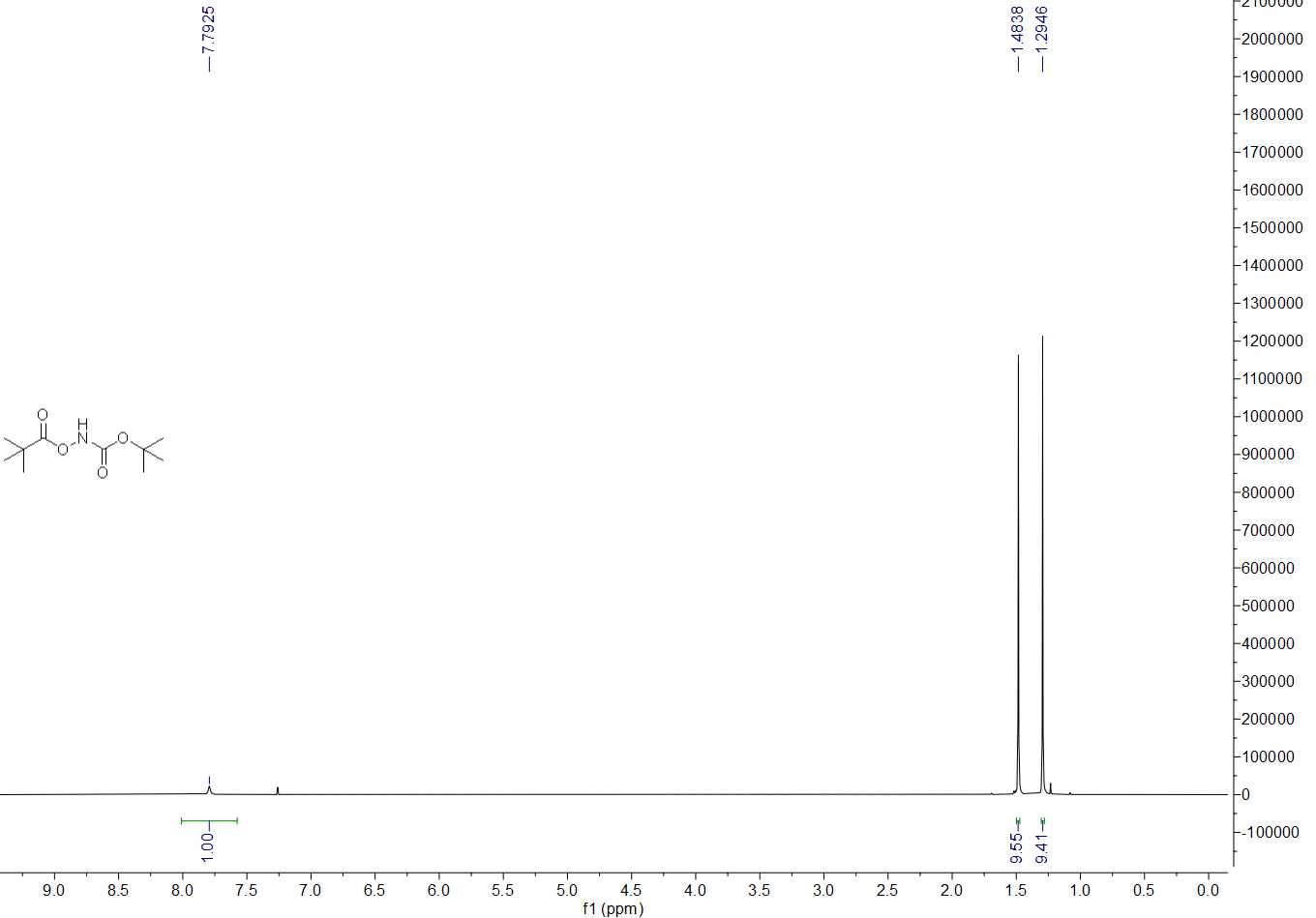


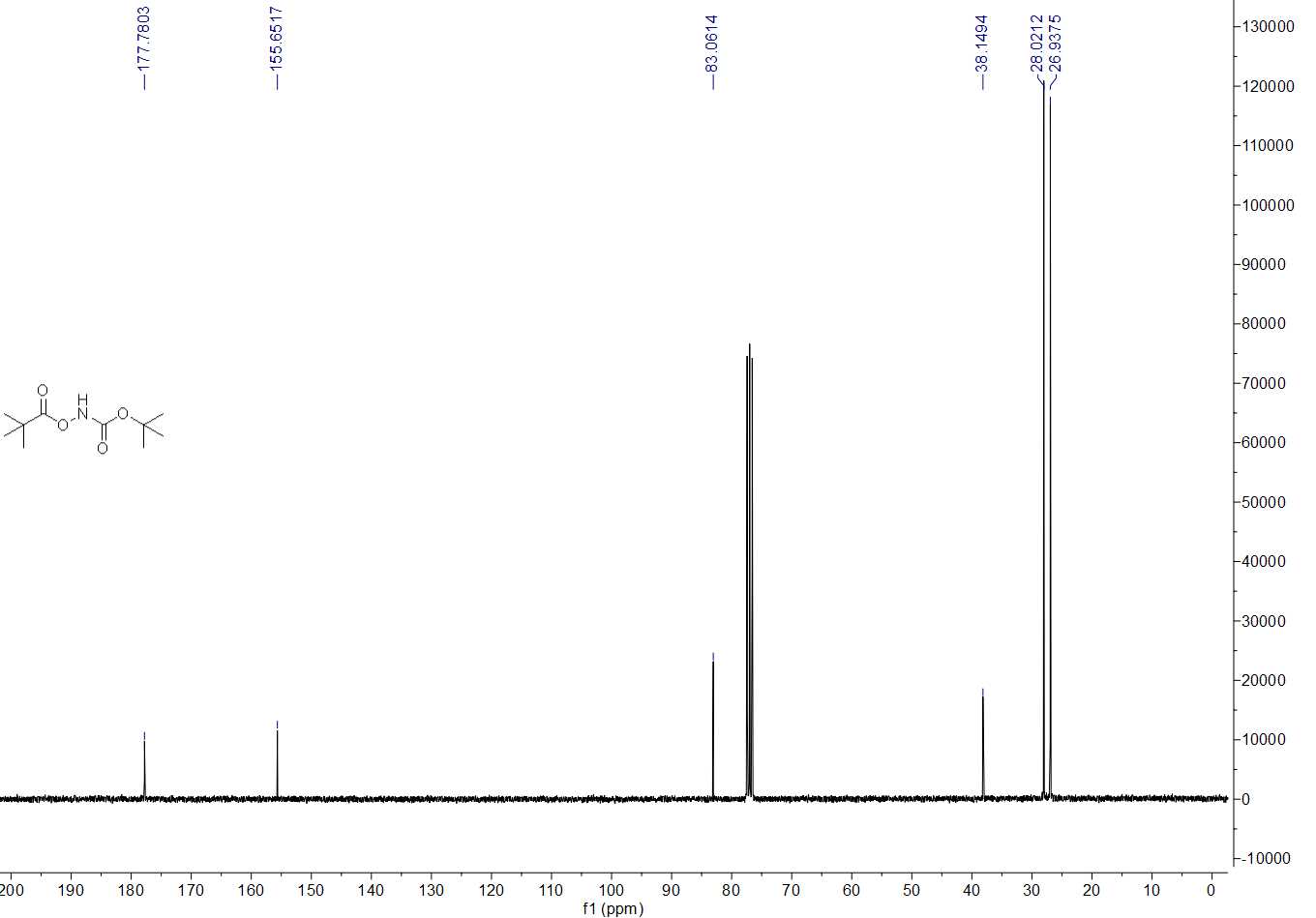


**Supplementary Fig 19.** ^1^H (upper part) and ^13^C NMR (lower part) of PivONHBoc (**5r**).


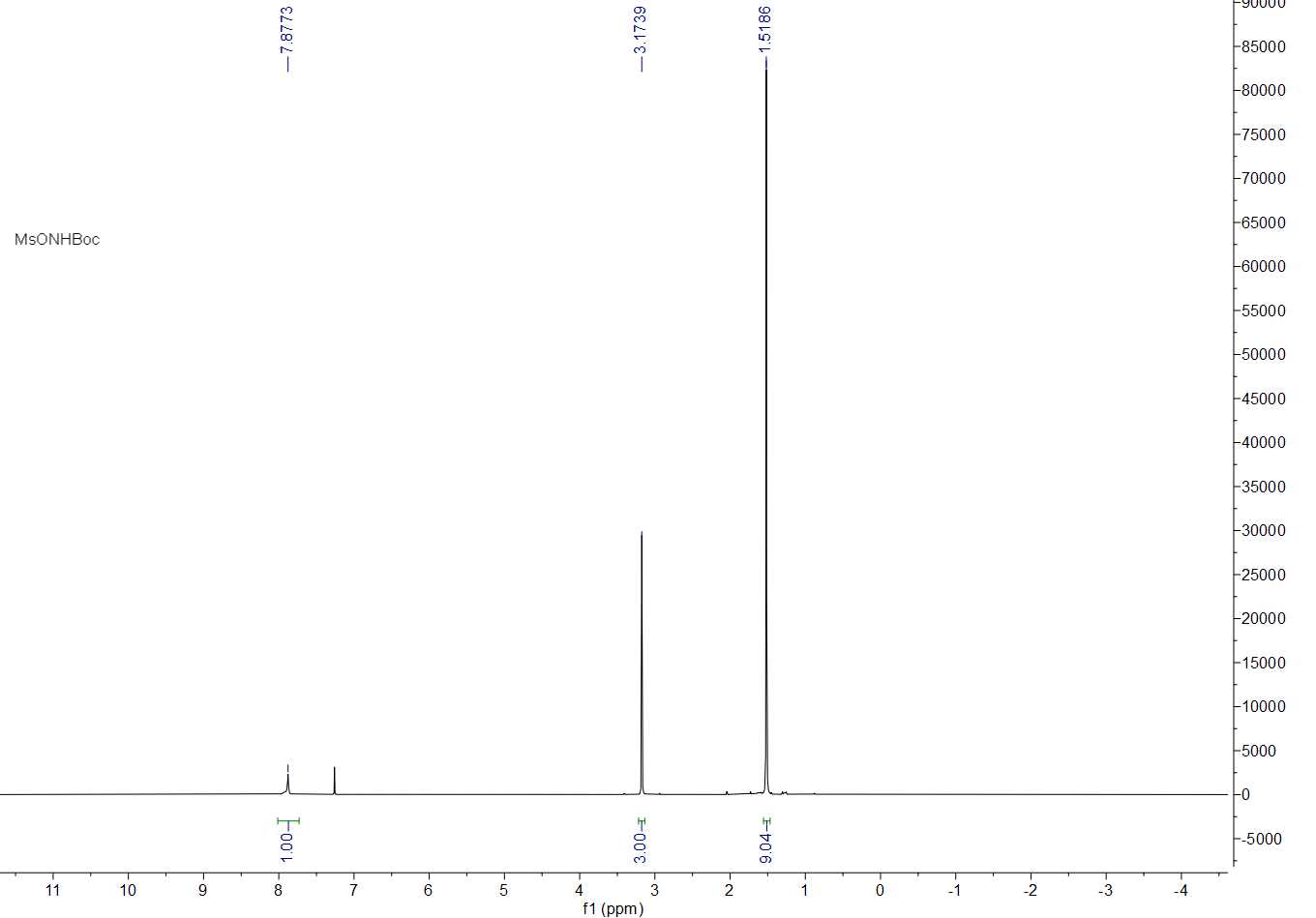


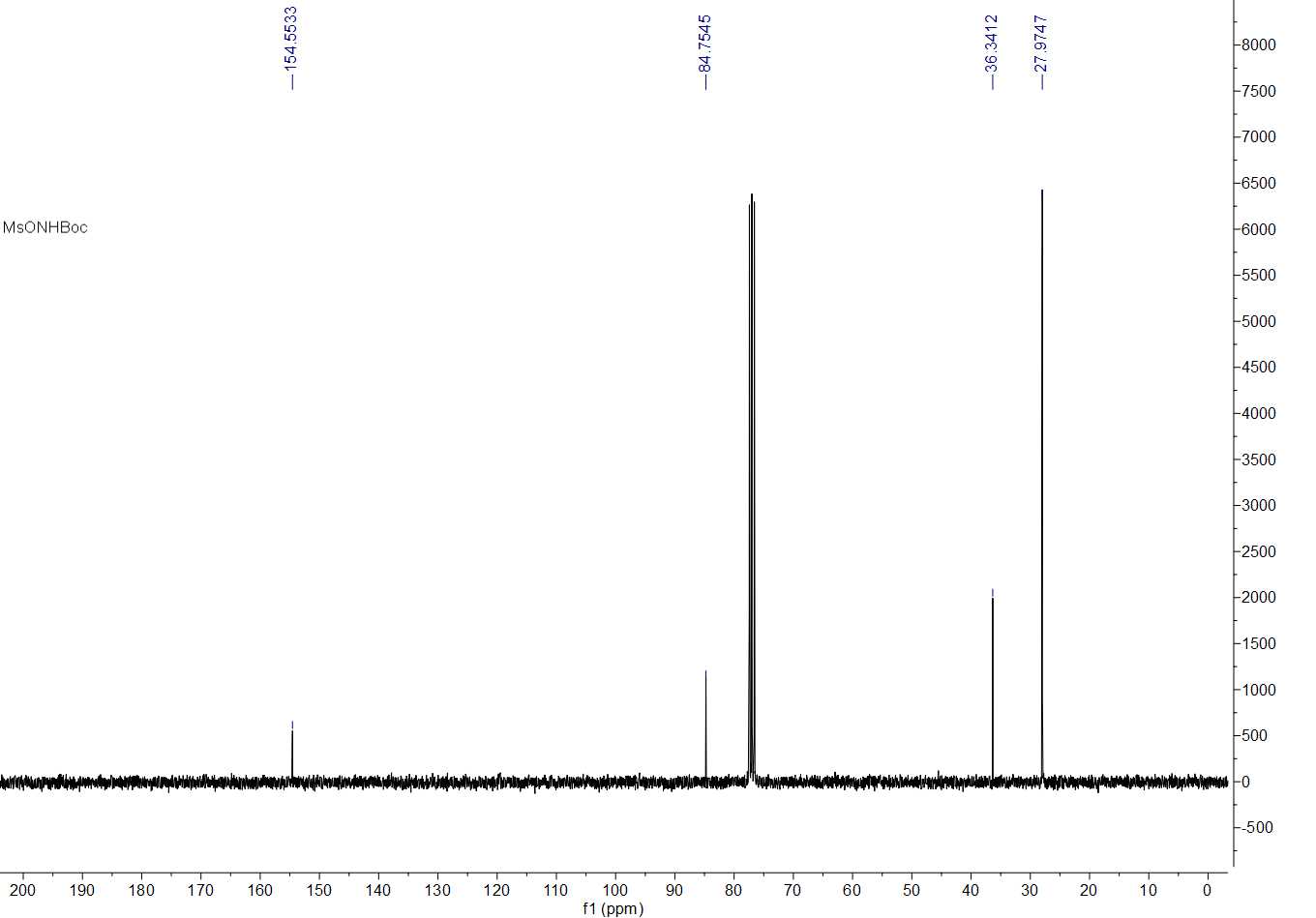


**Supplementary Fig 20.** ^1^H (upper part) and ^13^C NMR (lower part) of MsONHBoc.


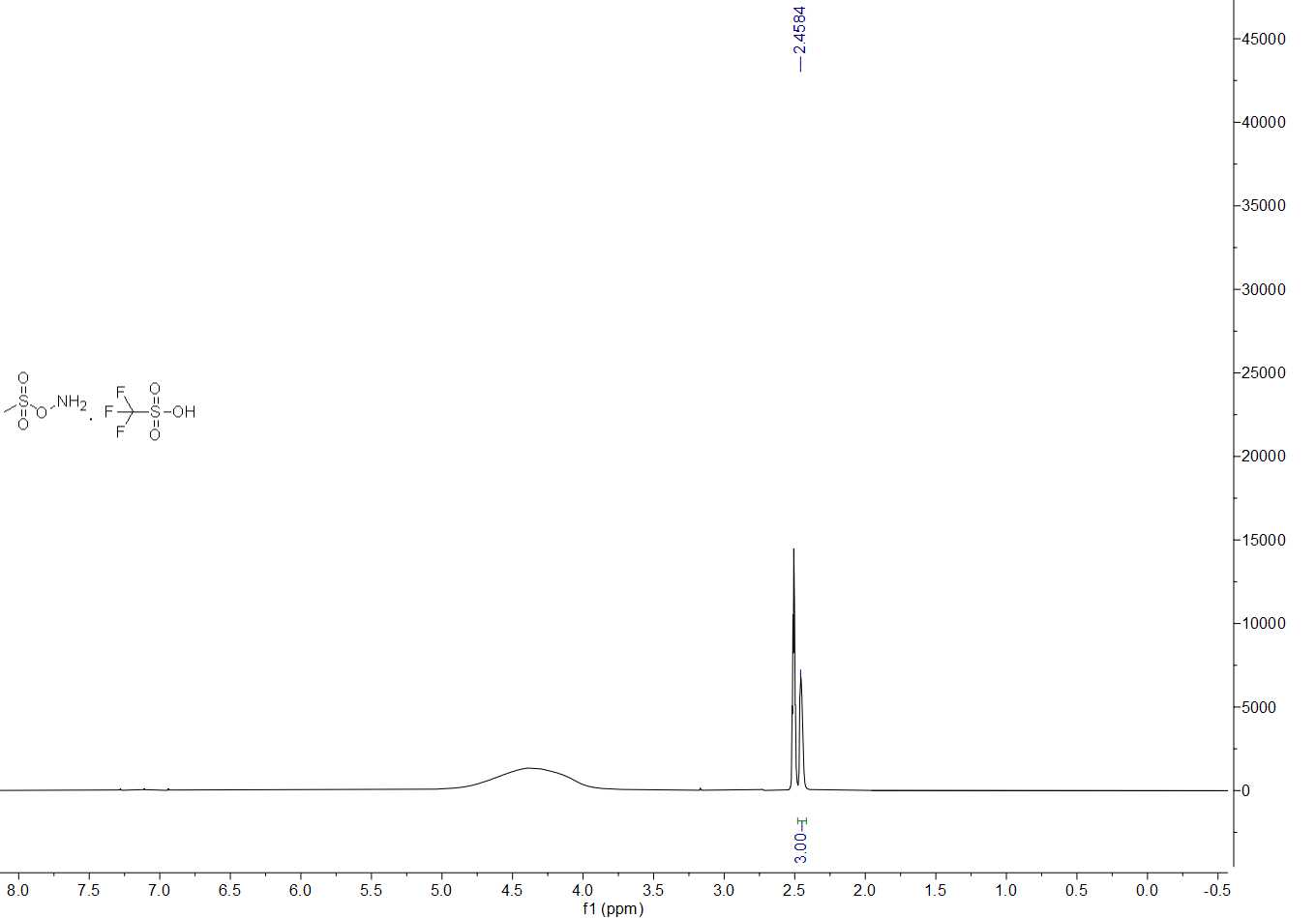


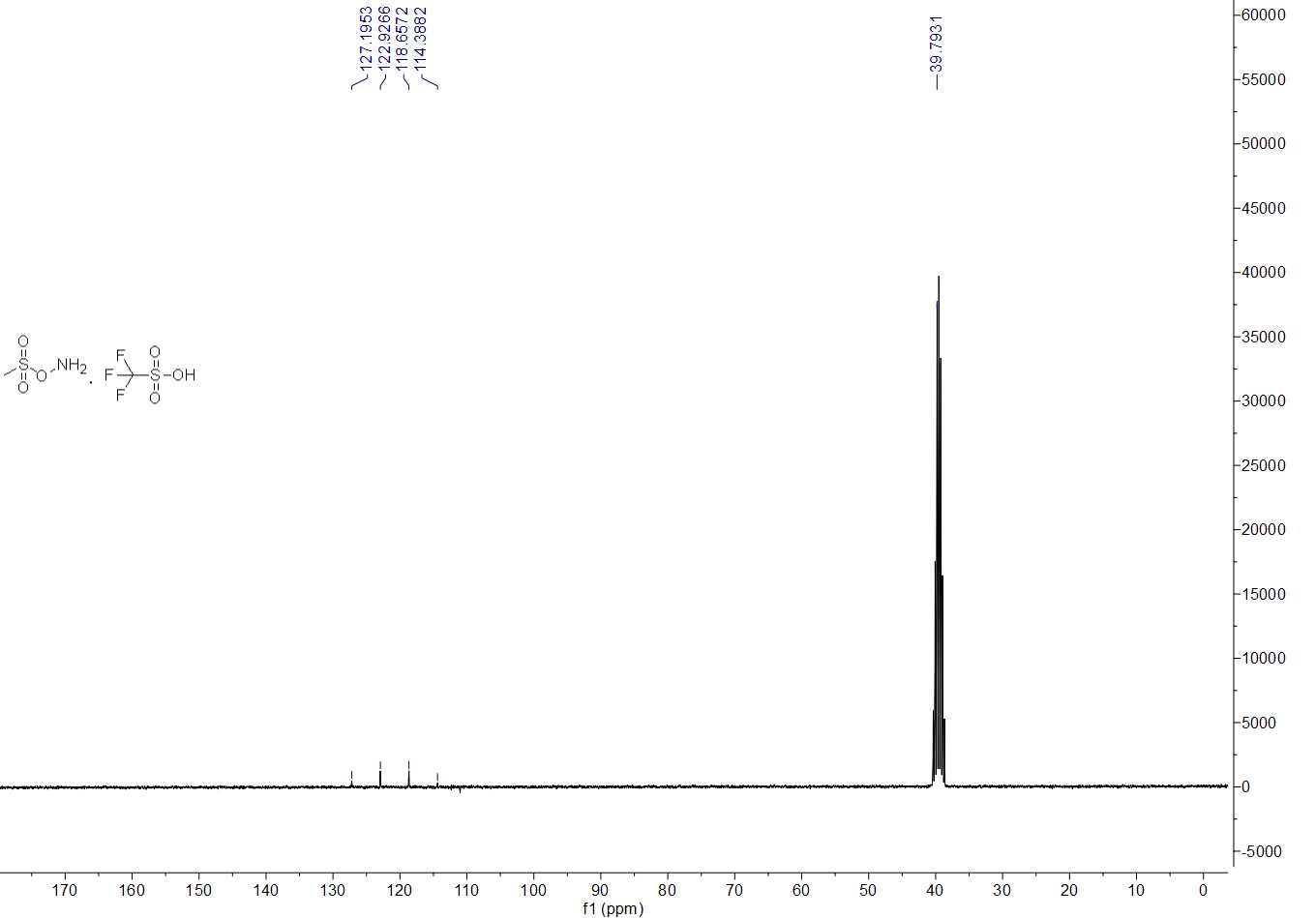


**Supplementary Fig 21.** ^1^H (upper part) and ^13^C NMR (lower part) of **5t.**


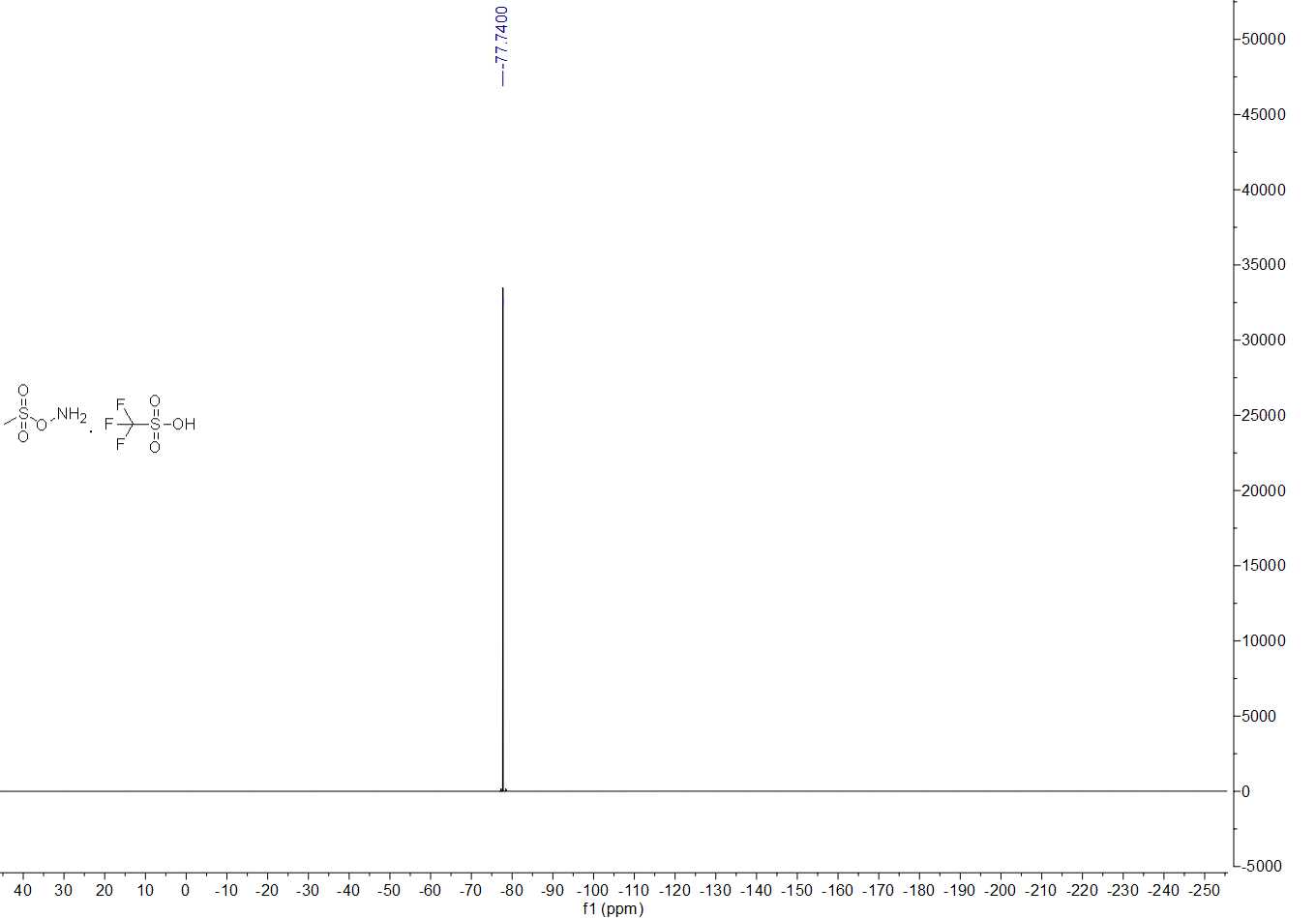


**Supplementary Fig 22.** ^19^F NMR of **5t**.


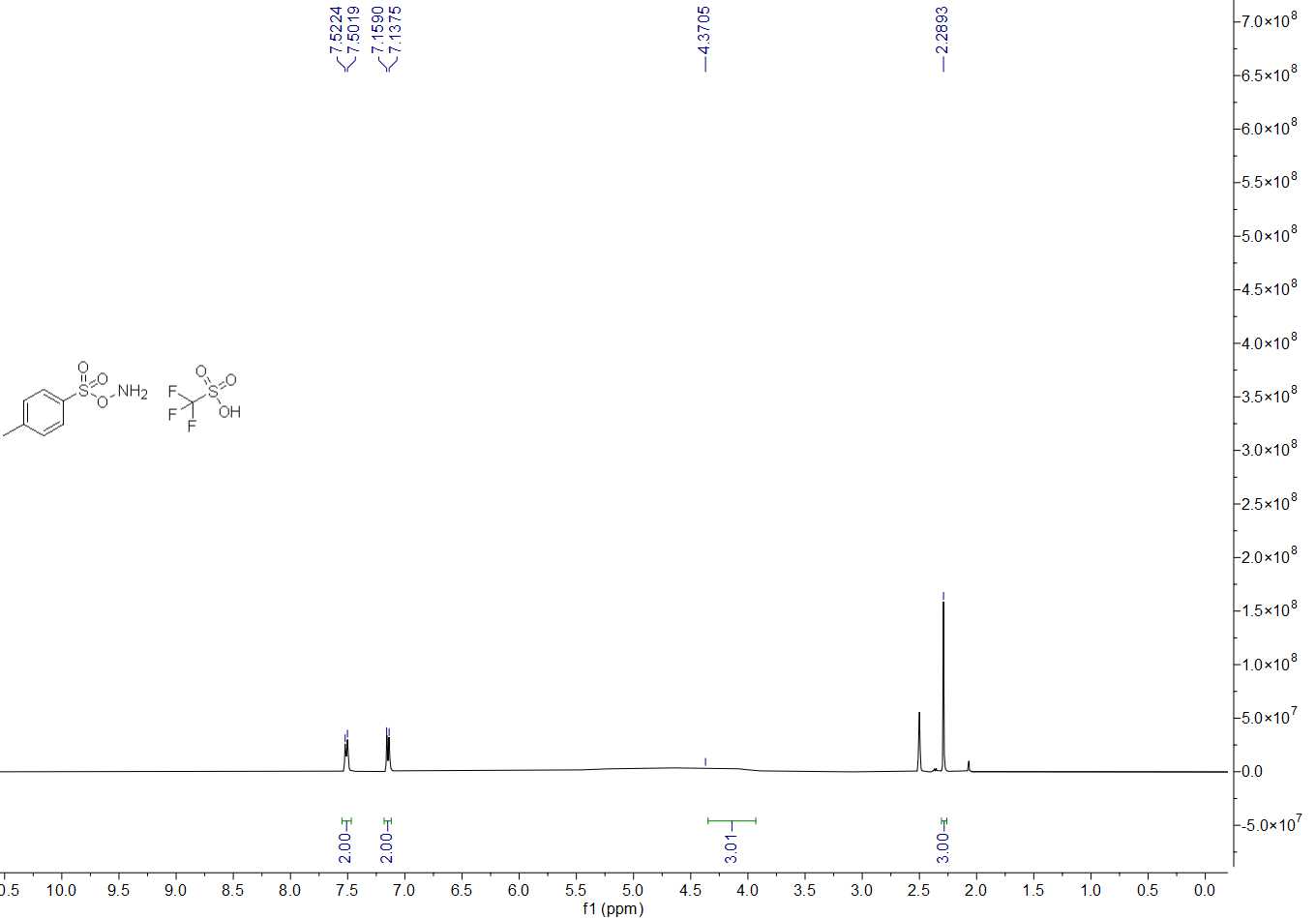


**Supplementary Fig 23.** ^1^H NMR of **5u**.


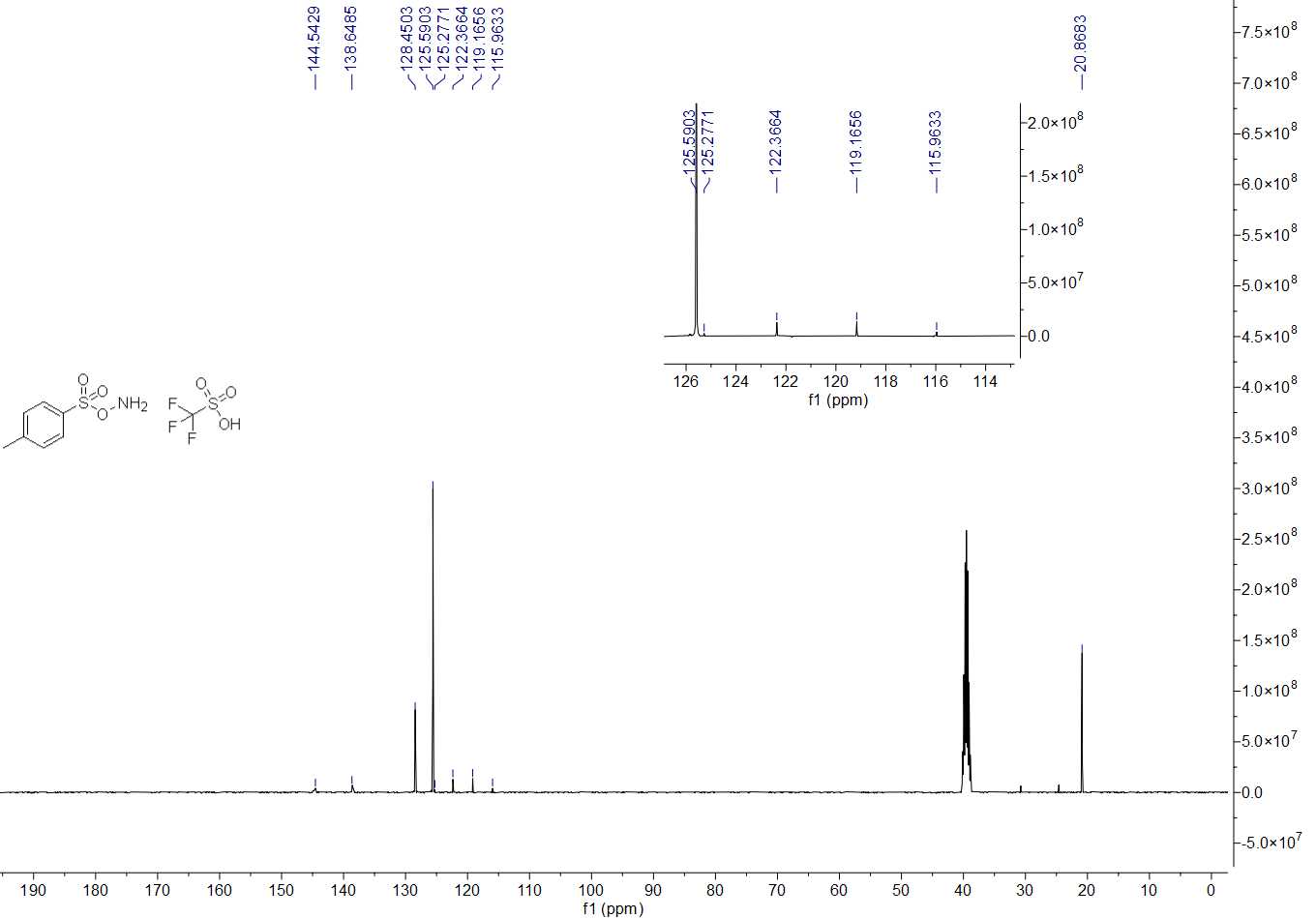


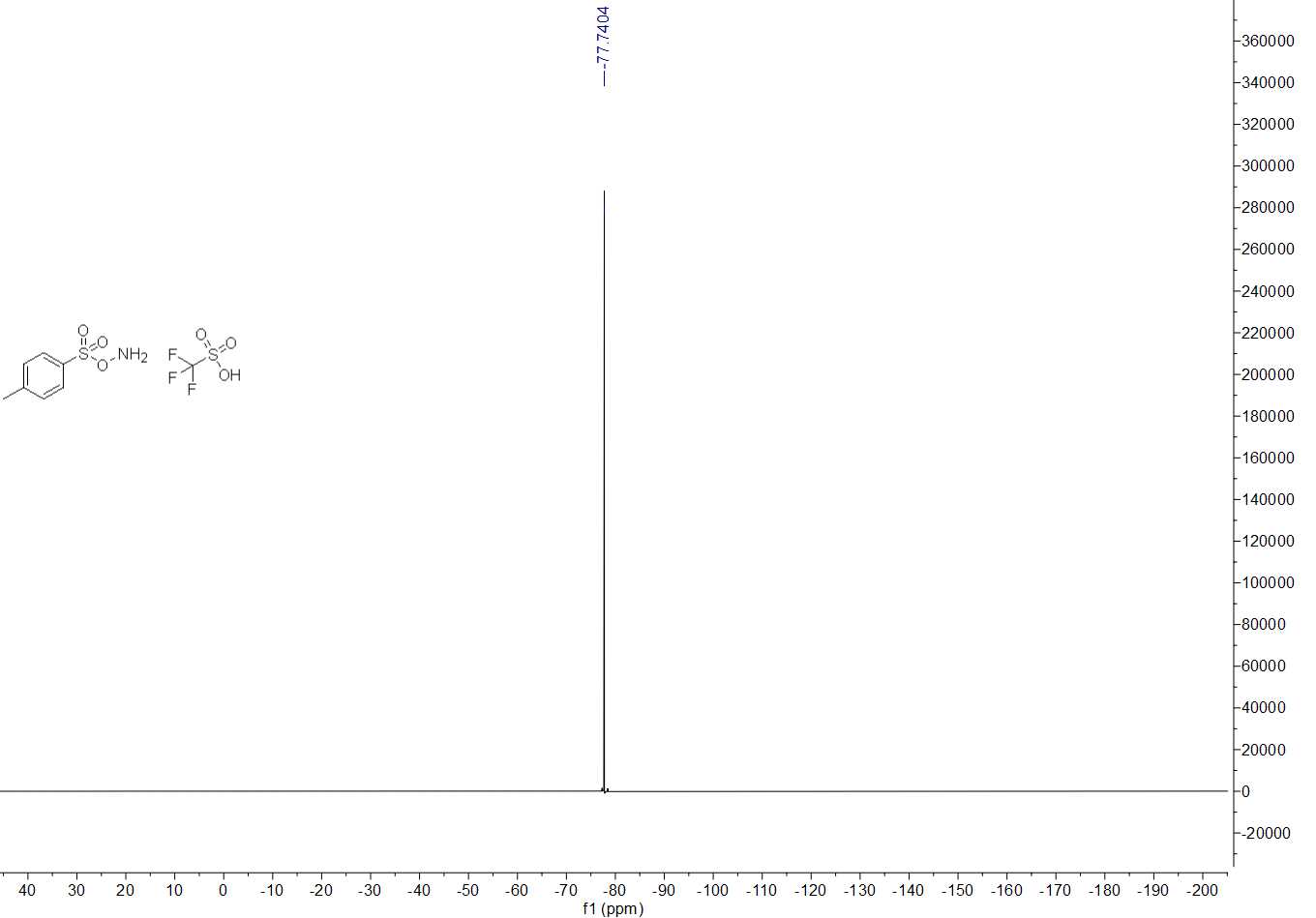


**Supplementary Fig 24.** ^13^C NMR (upper part) and ^19^F NMR (lower part) of **5u**.


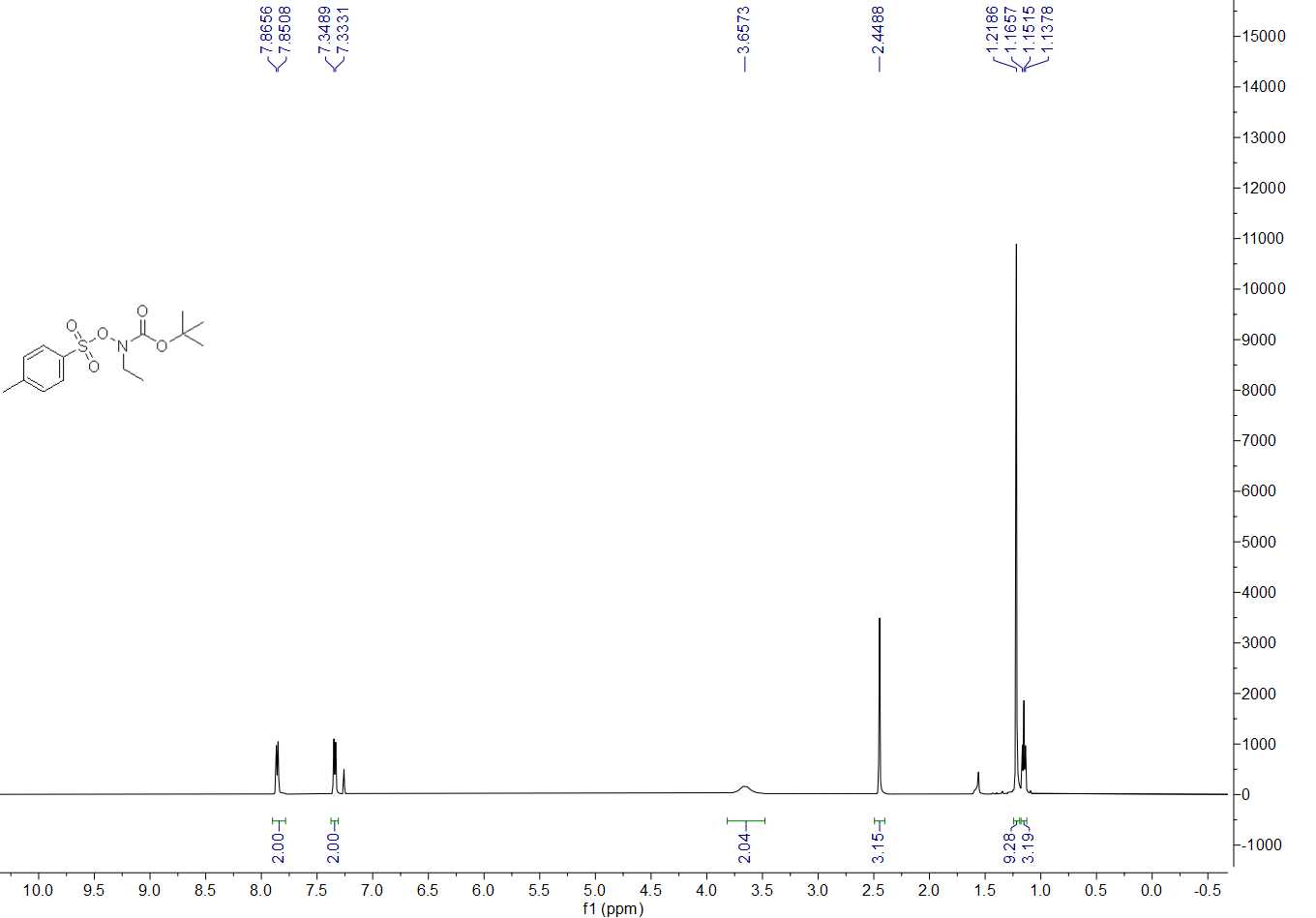


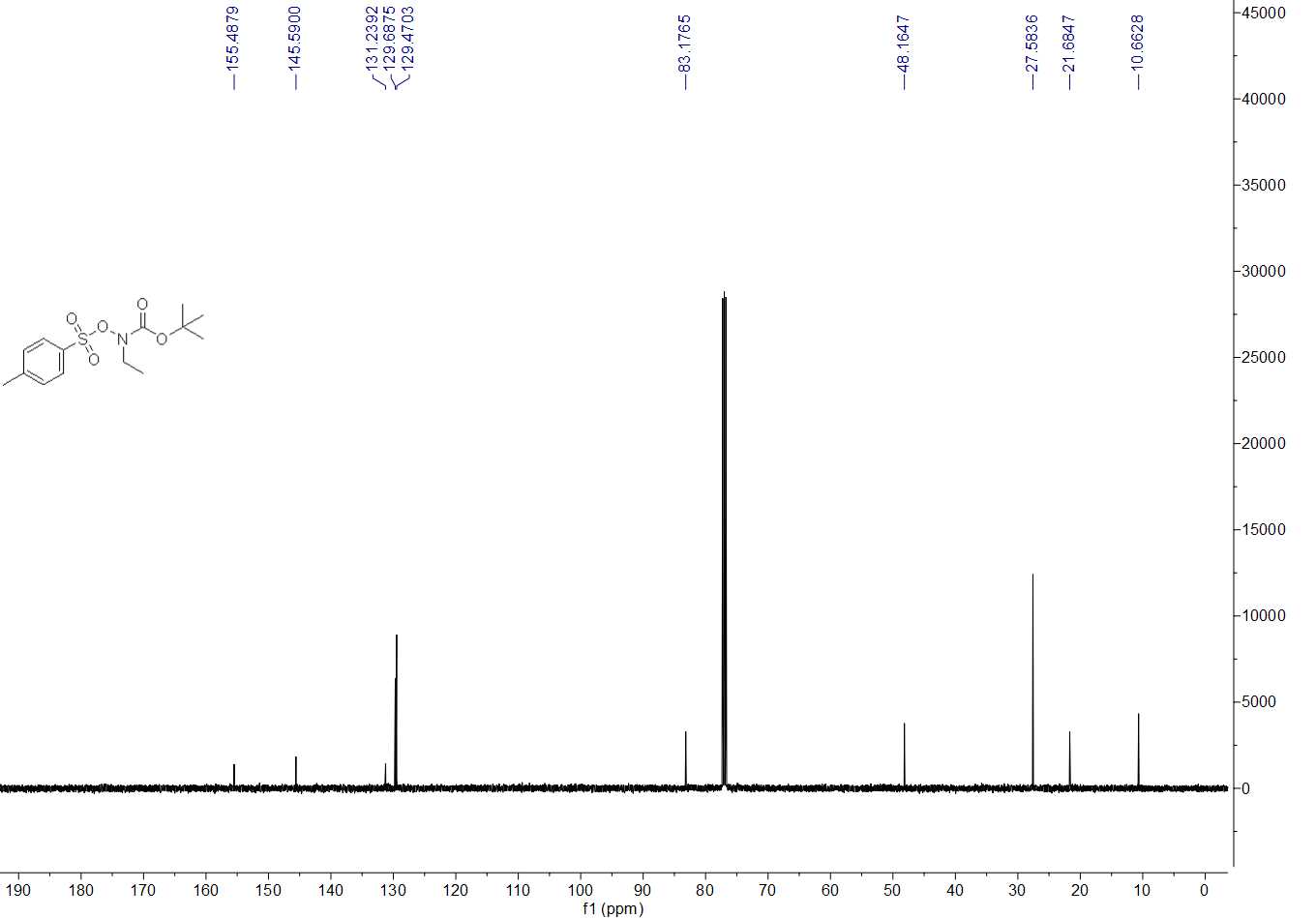


**Supplementary Fig 25.** ^1^H (upper part) and ^13^C NMR (lower part) of *tert*-butyl ethyl(tosyloxy)carbamate.


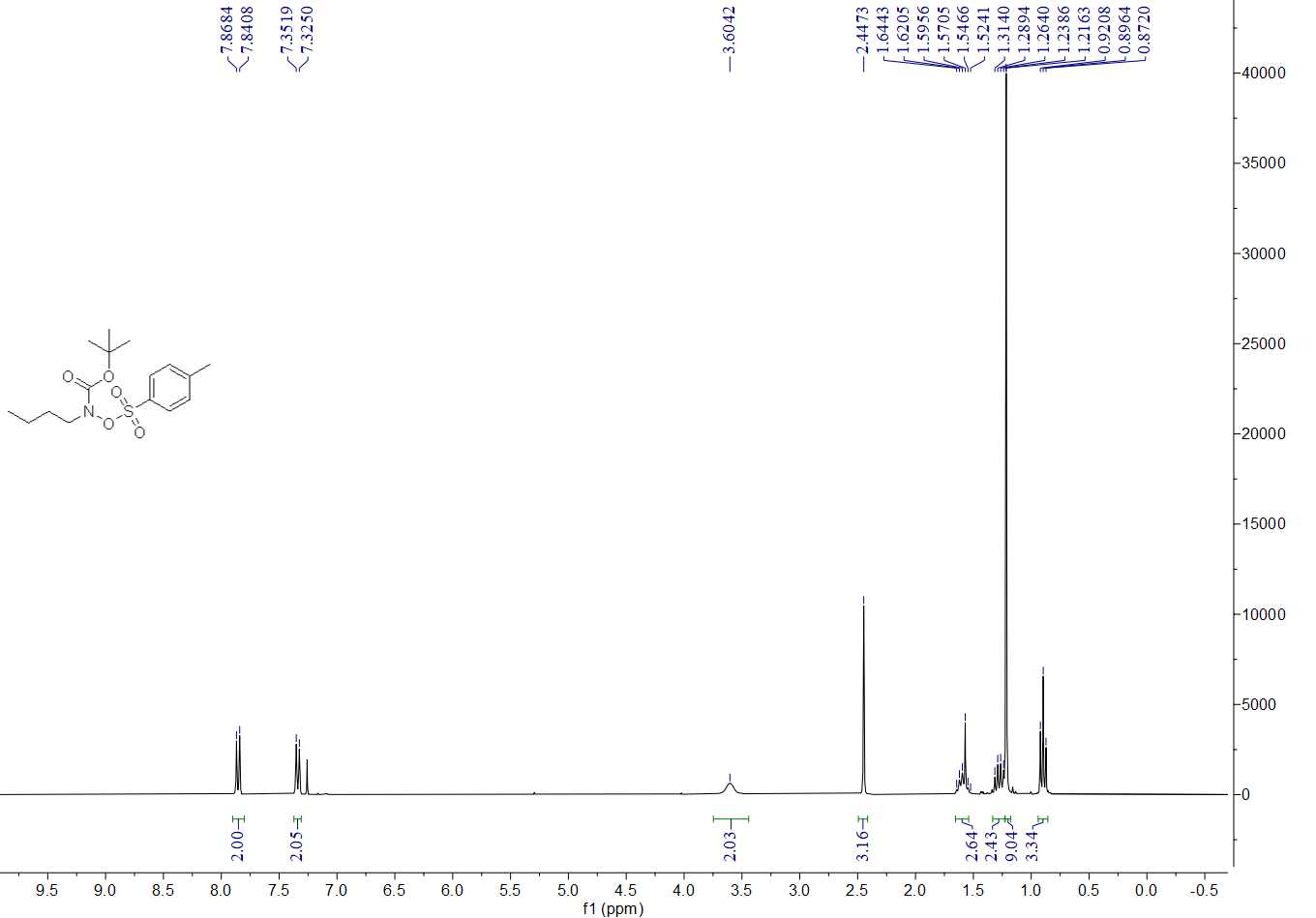


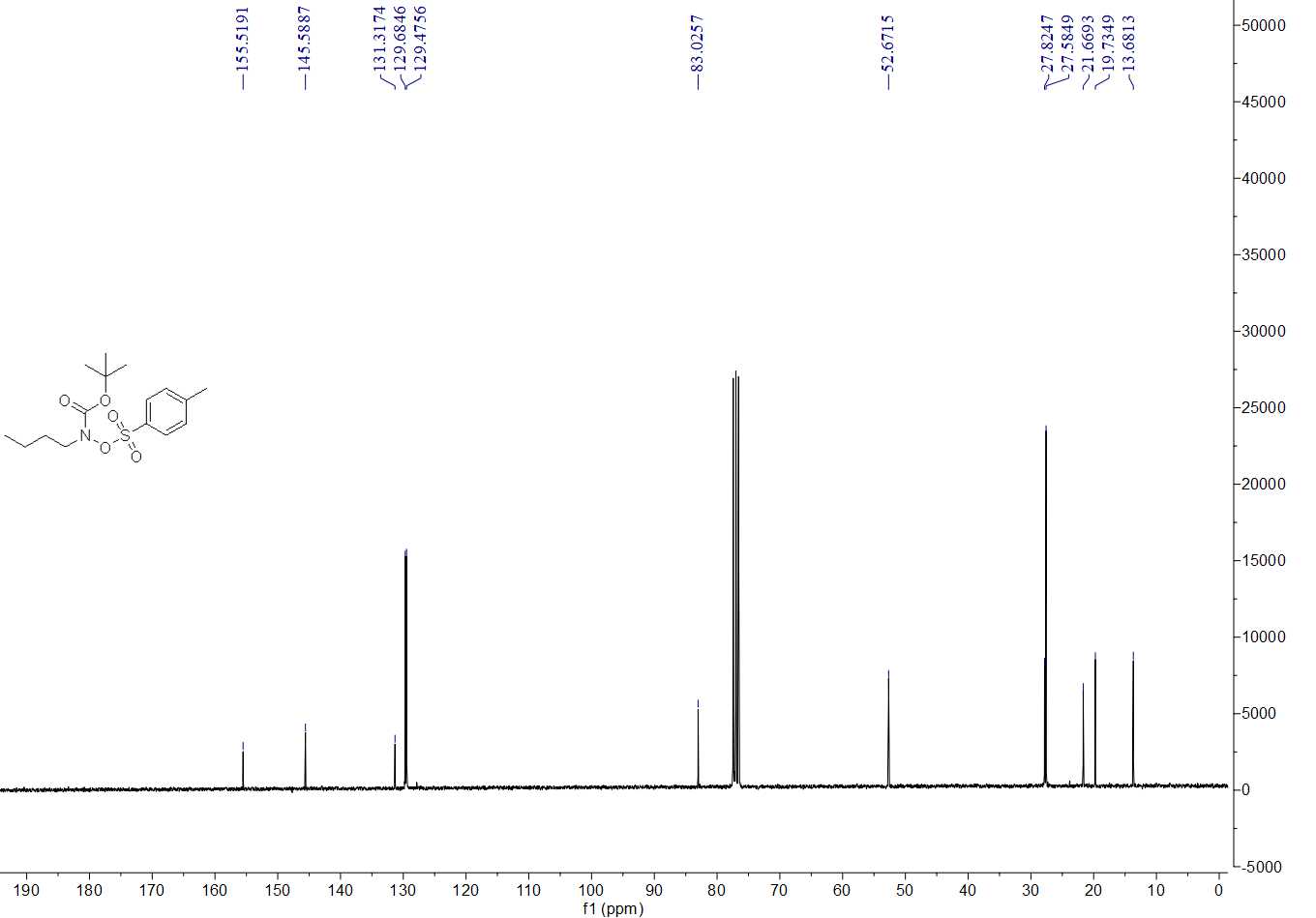


**Supplementary Fig 26.** ^1^H (upper part) and ^13^C NMR (lower part) of *tert*-butyl butyl(tosyloxy)carbamate.


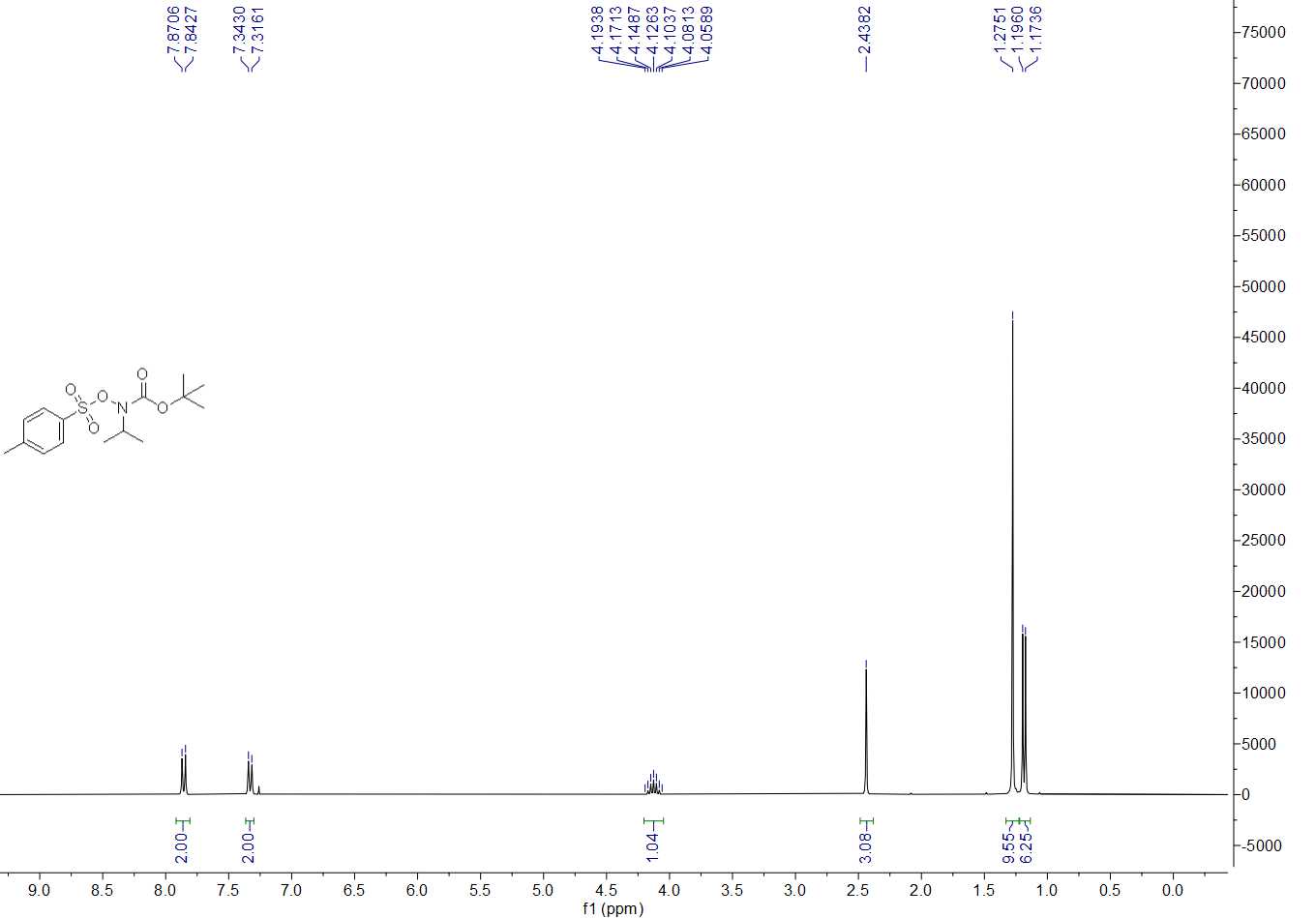


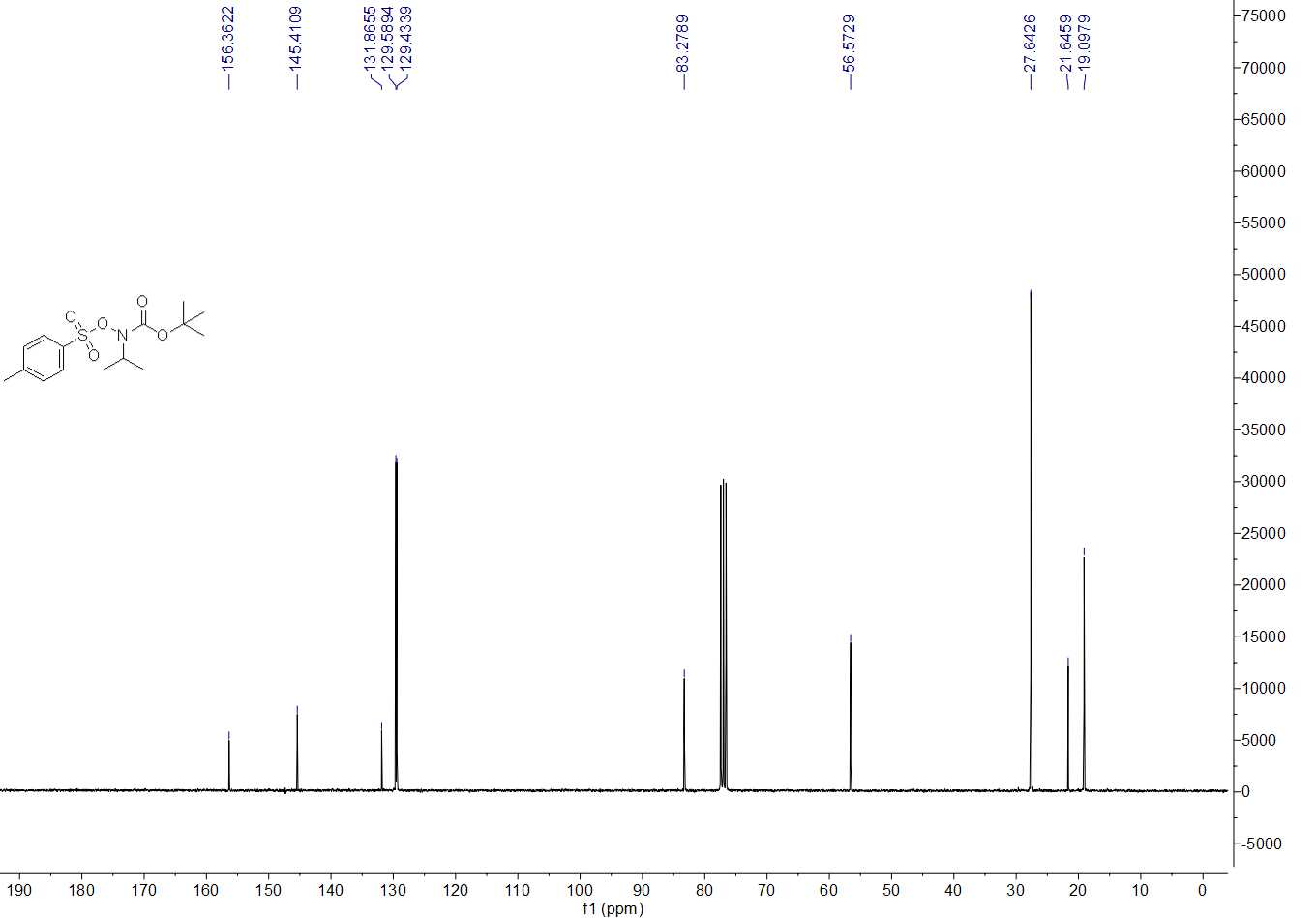


**Supplementary Fig 27.** ^1^H (upper part) and ^13^C NMR (lower part) of *tert*-butyl isopropyl(tosyloxy)carbamate.


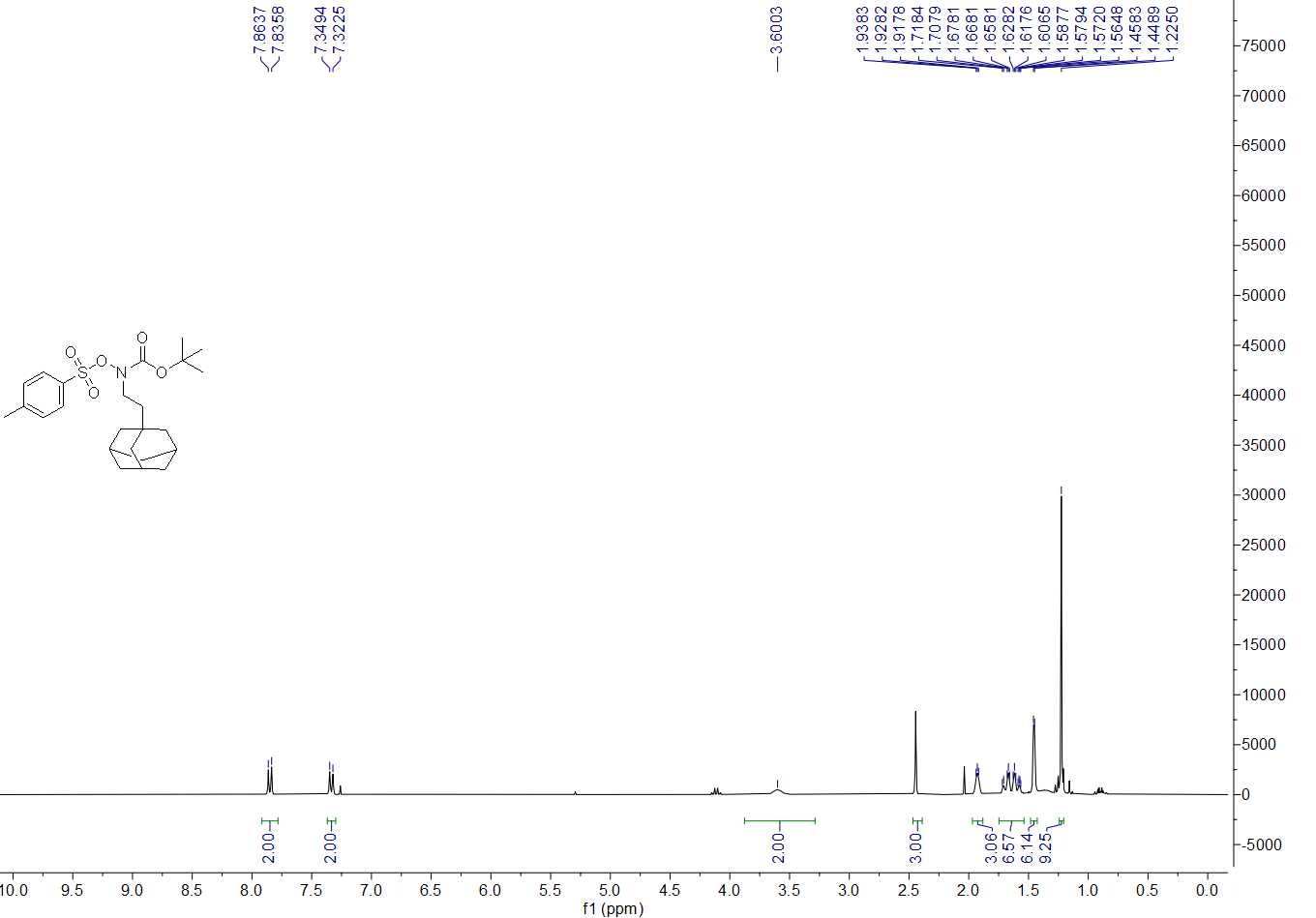


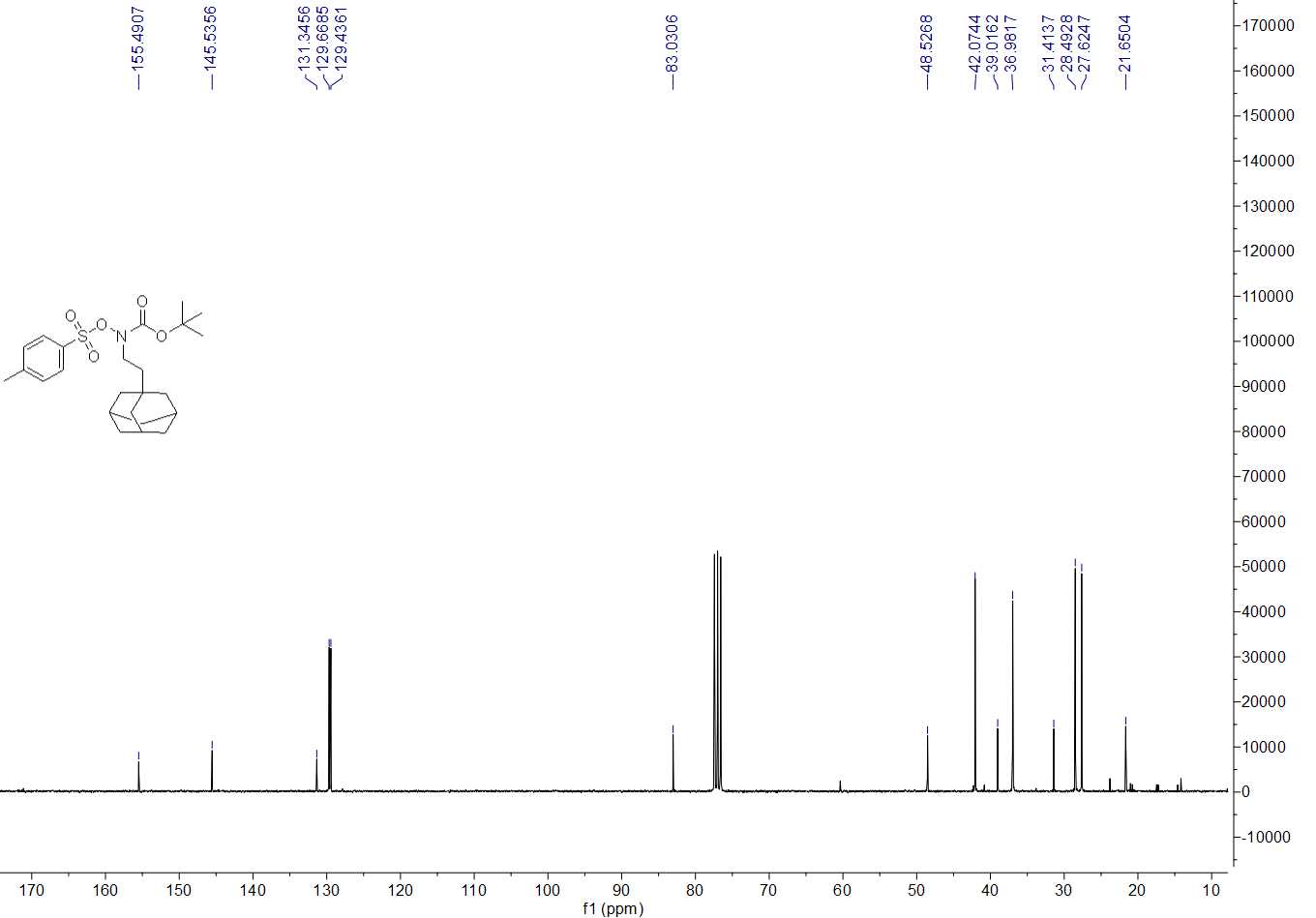


**Supplementary Fig 28.** ^1^H (upper part) and ^13^C NMR (lower part) of *tert*-butyl (2-(adamantan-1-yl)ethyl)(tosyloxy)carbamate.


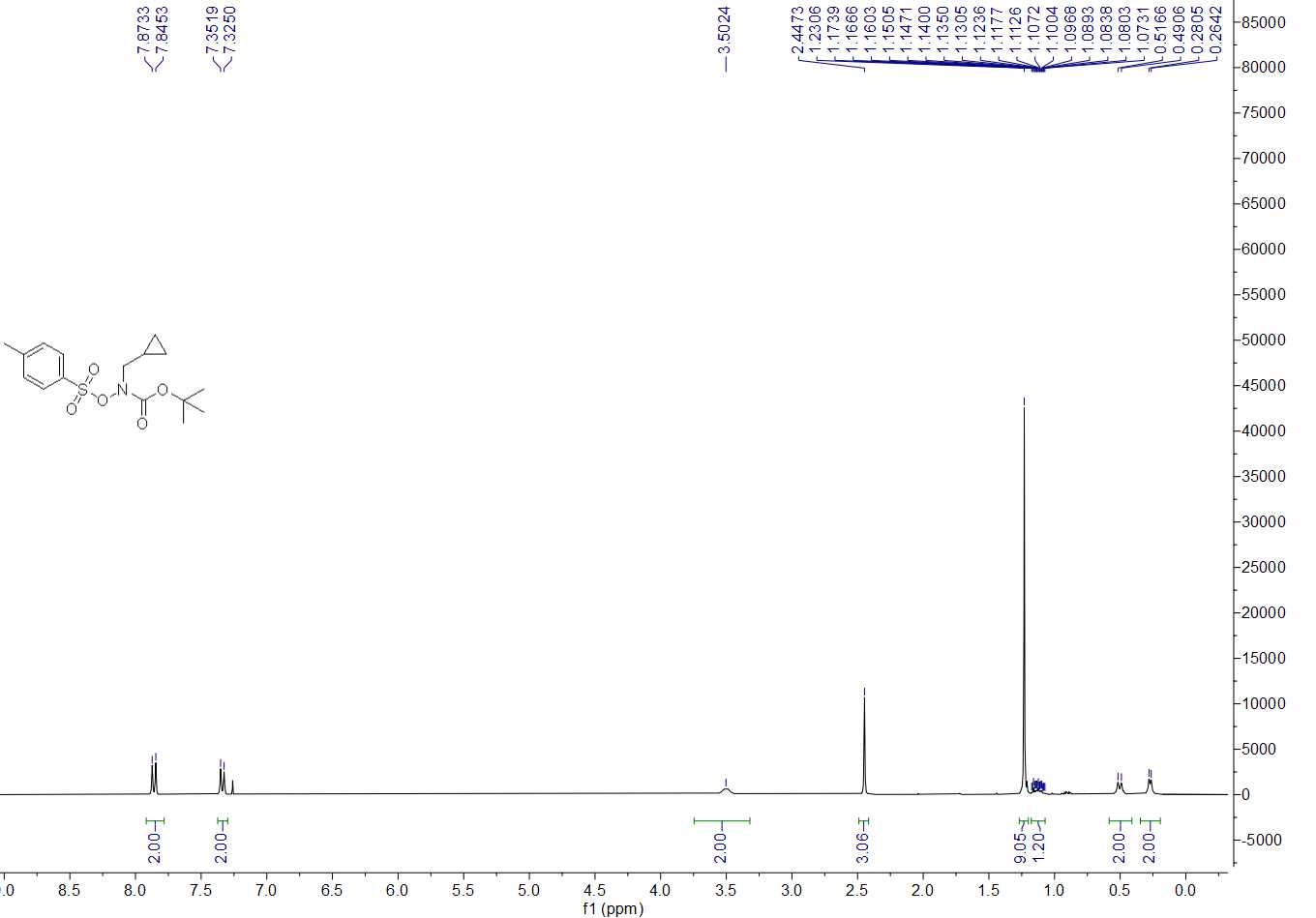


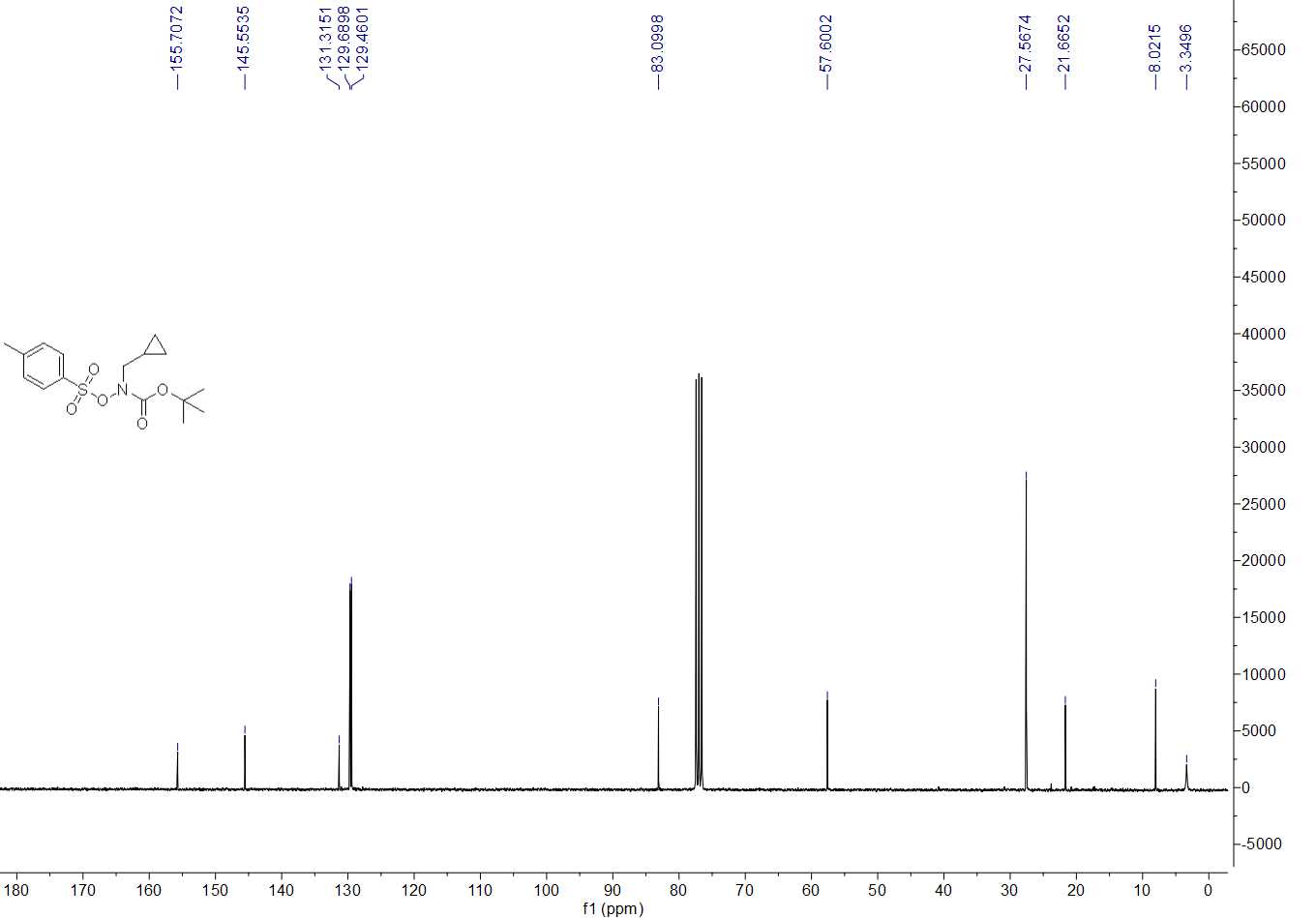


**Supplementary Fig 29.** ^1^H (upper part) and ^13^C NMR (lower part) of *tert*-butyl (cyclopropylmethyl)(tosyloxy)carbamate.


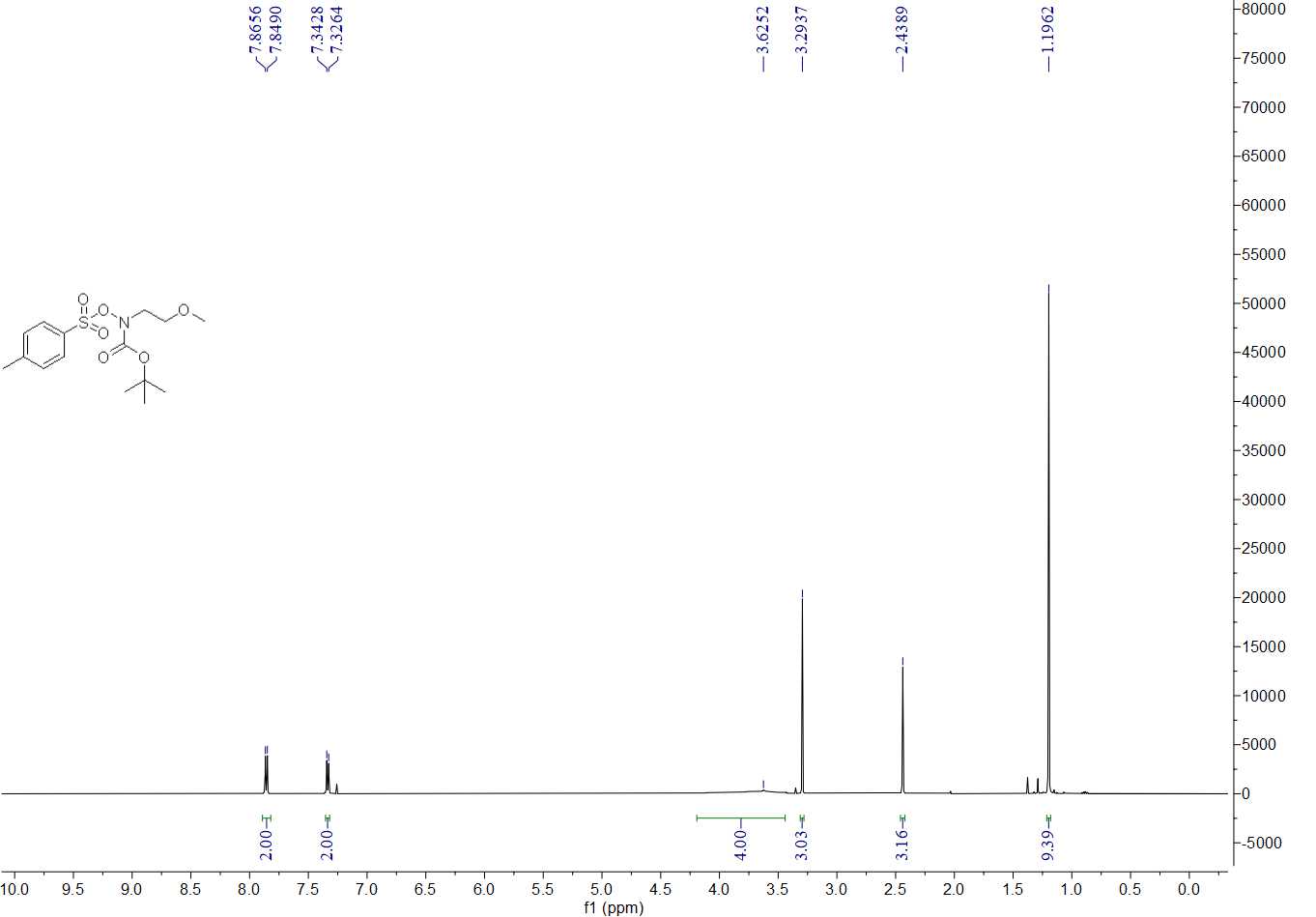


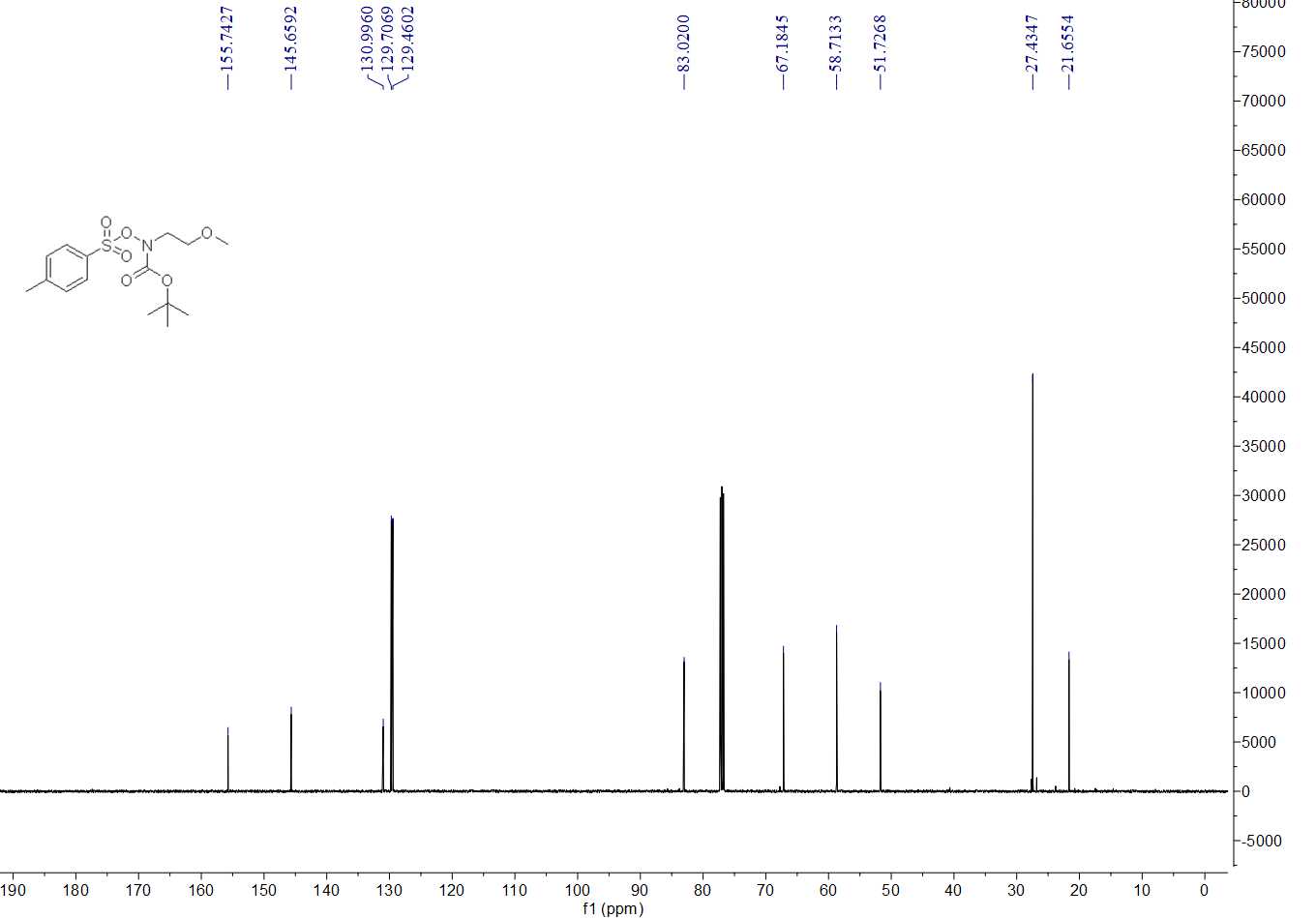


**Supplementary Fig 30.** ^1^H (upper part) and ^13^C NMR (lower part) of *tert*-butyl(2-methoxyethyl) (tosyloxy)carbamate.


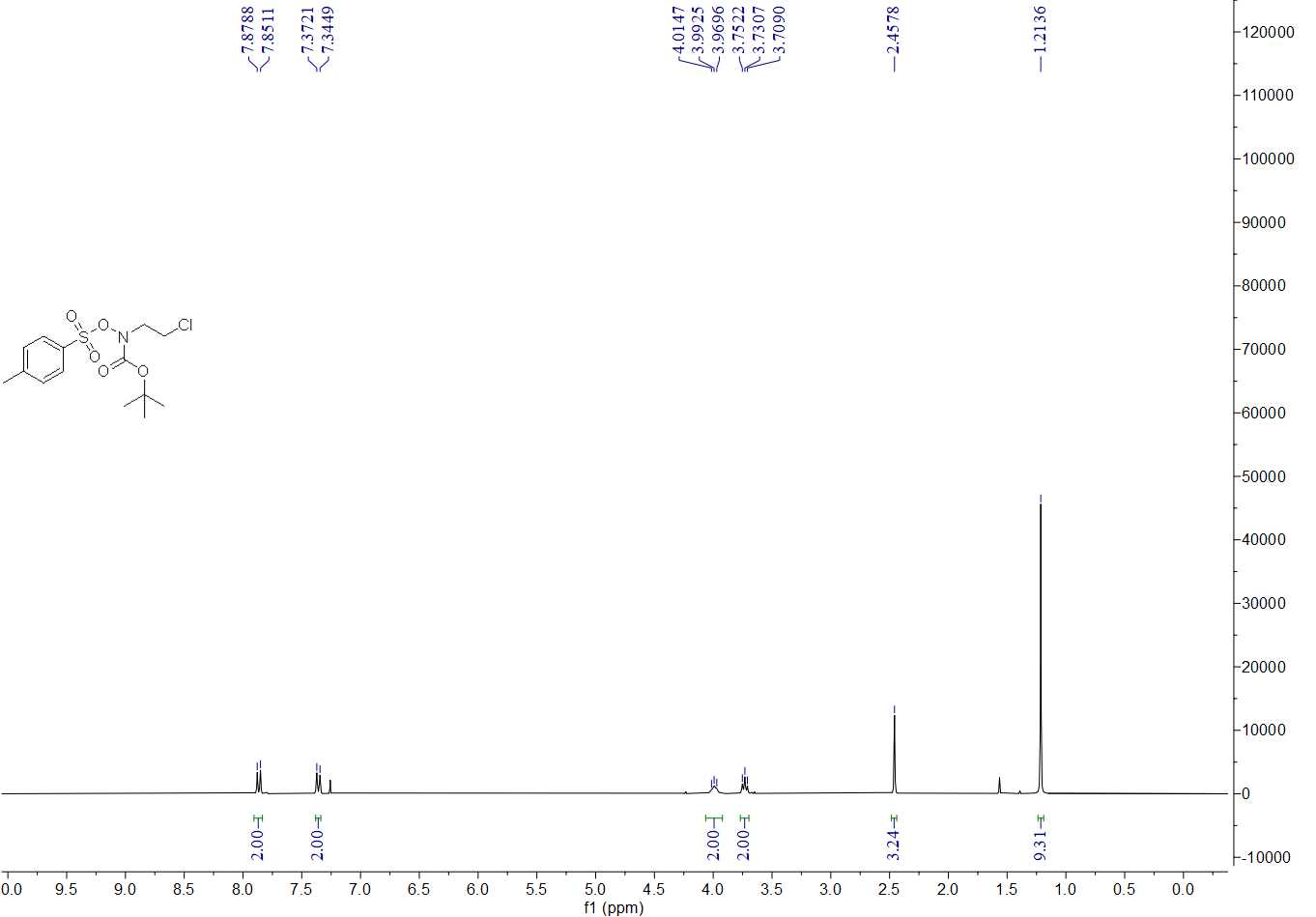


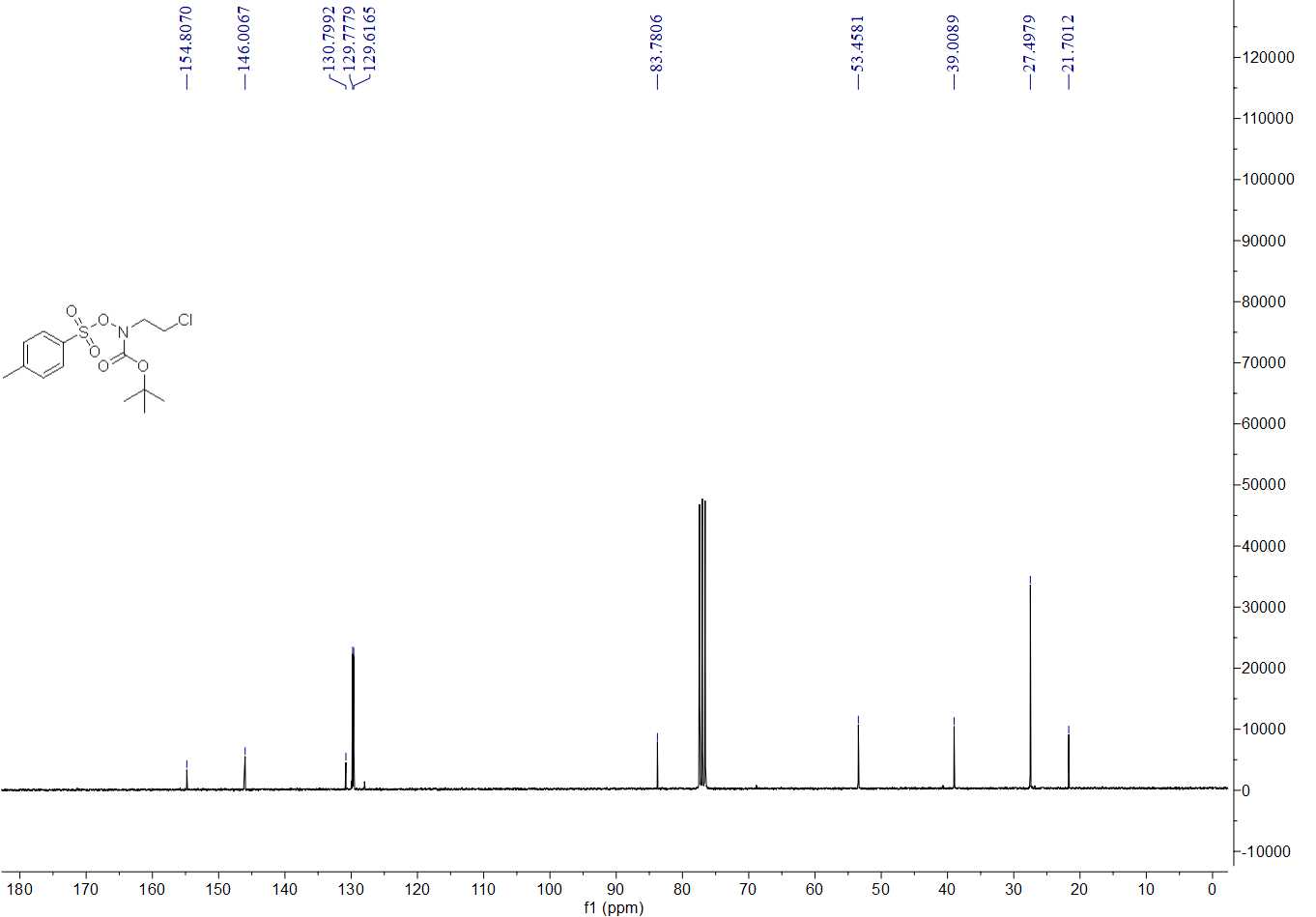


**Supplementary Fig 31.** ^1^H (upper part) and ^13^C NMR (lower part) of *tert*-butyl (2-chloroethyl)(tosyloxy)carbamate.


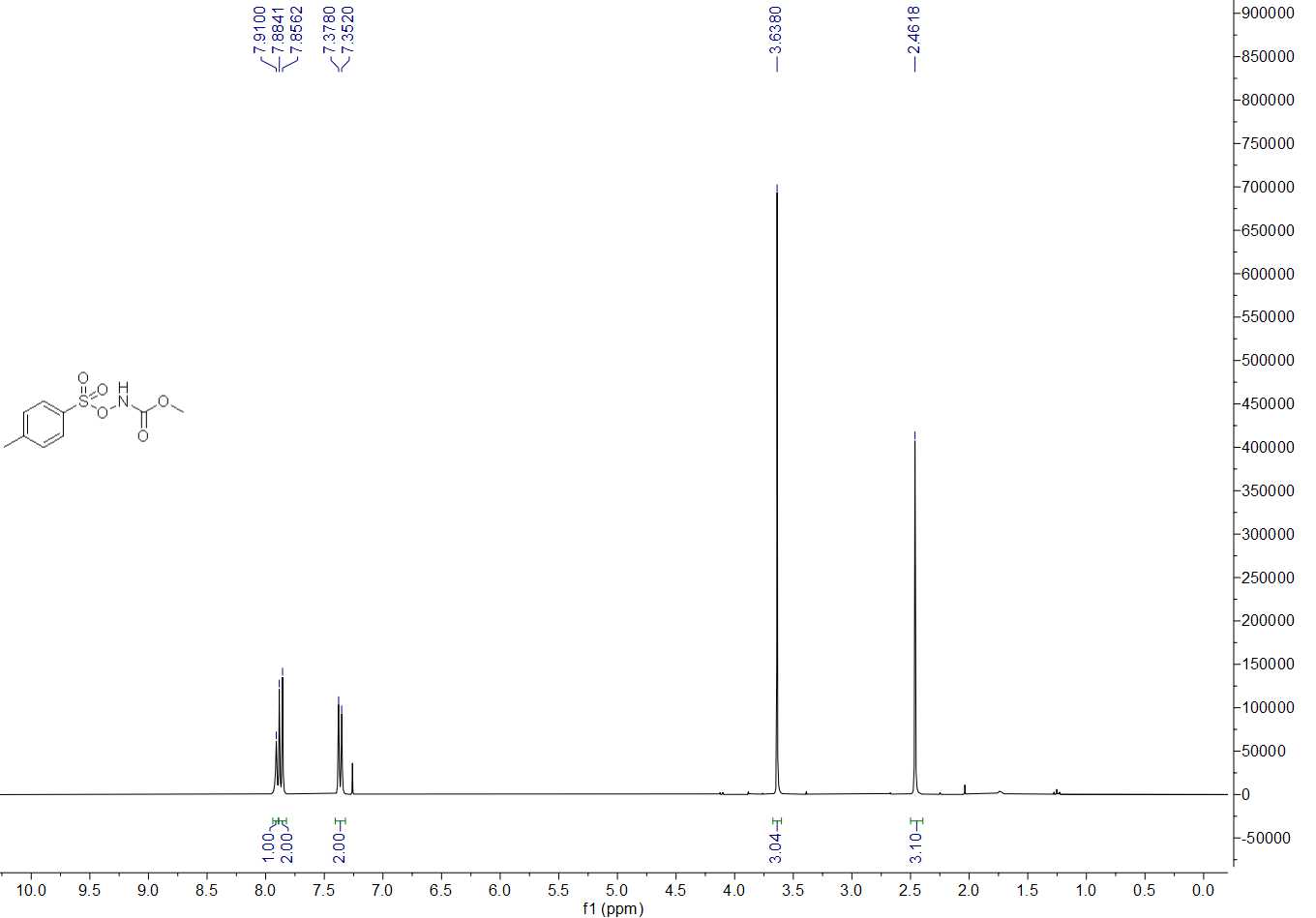


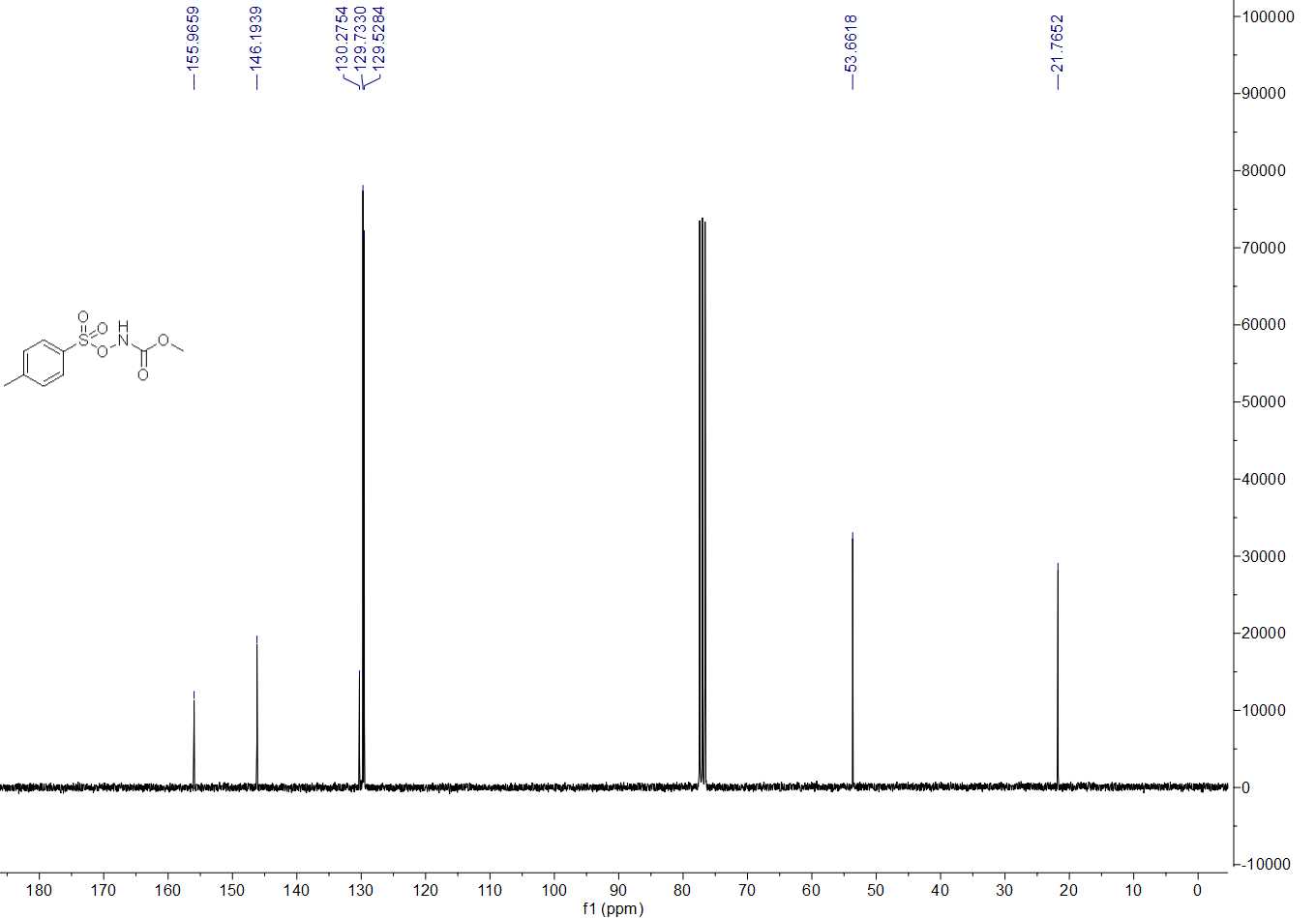


**Supplementary Fig 32.** ^1^H (upper part) and ^13^C NMR (lower part) of methyl (tosyloxy)carbamate.


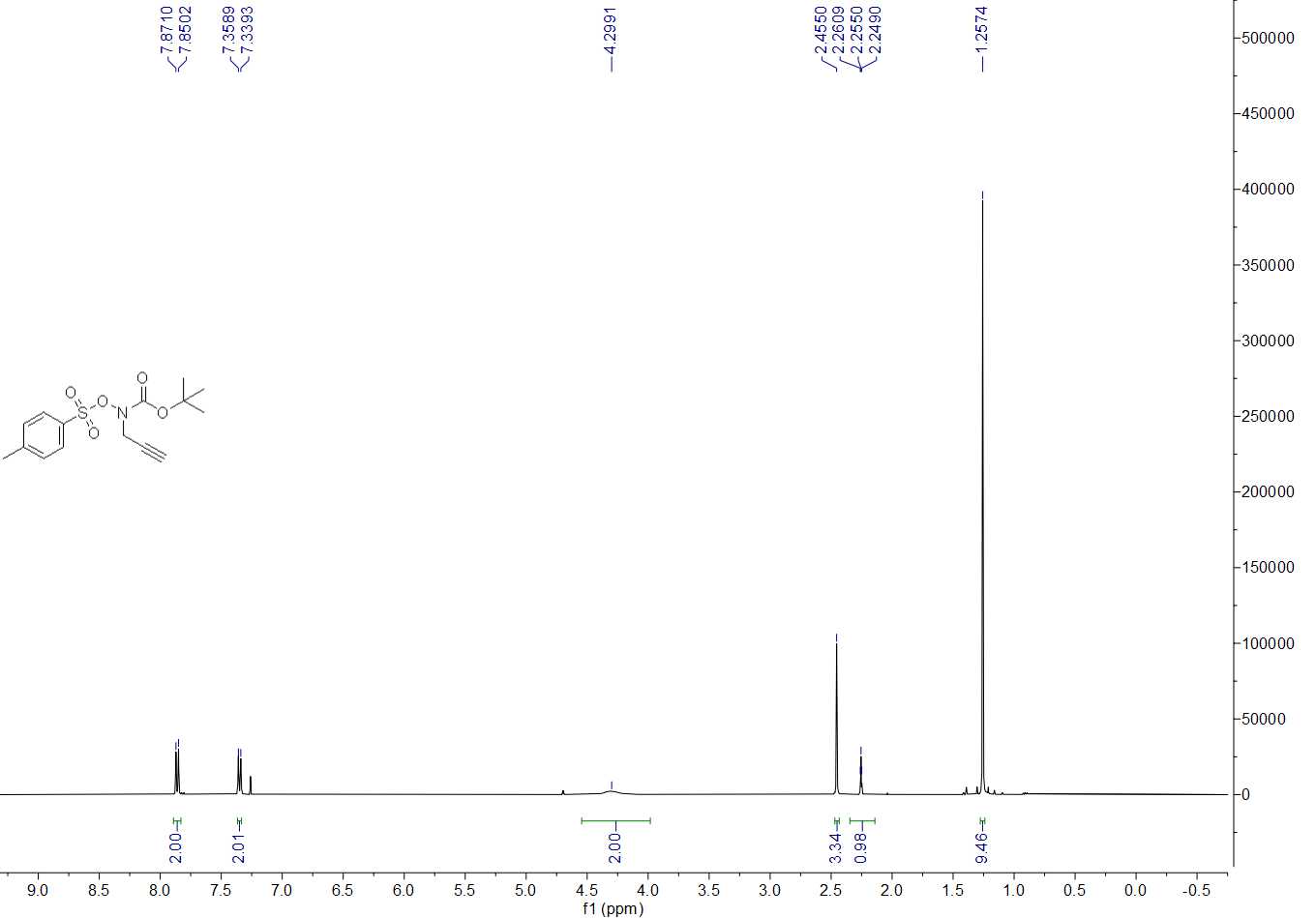


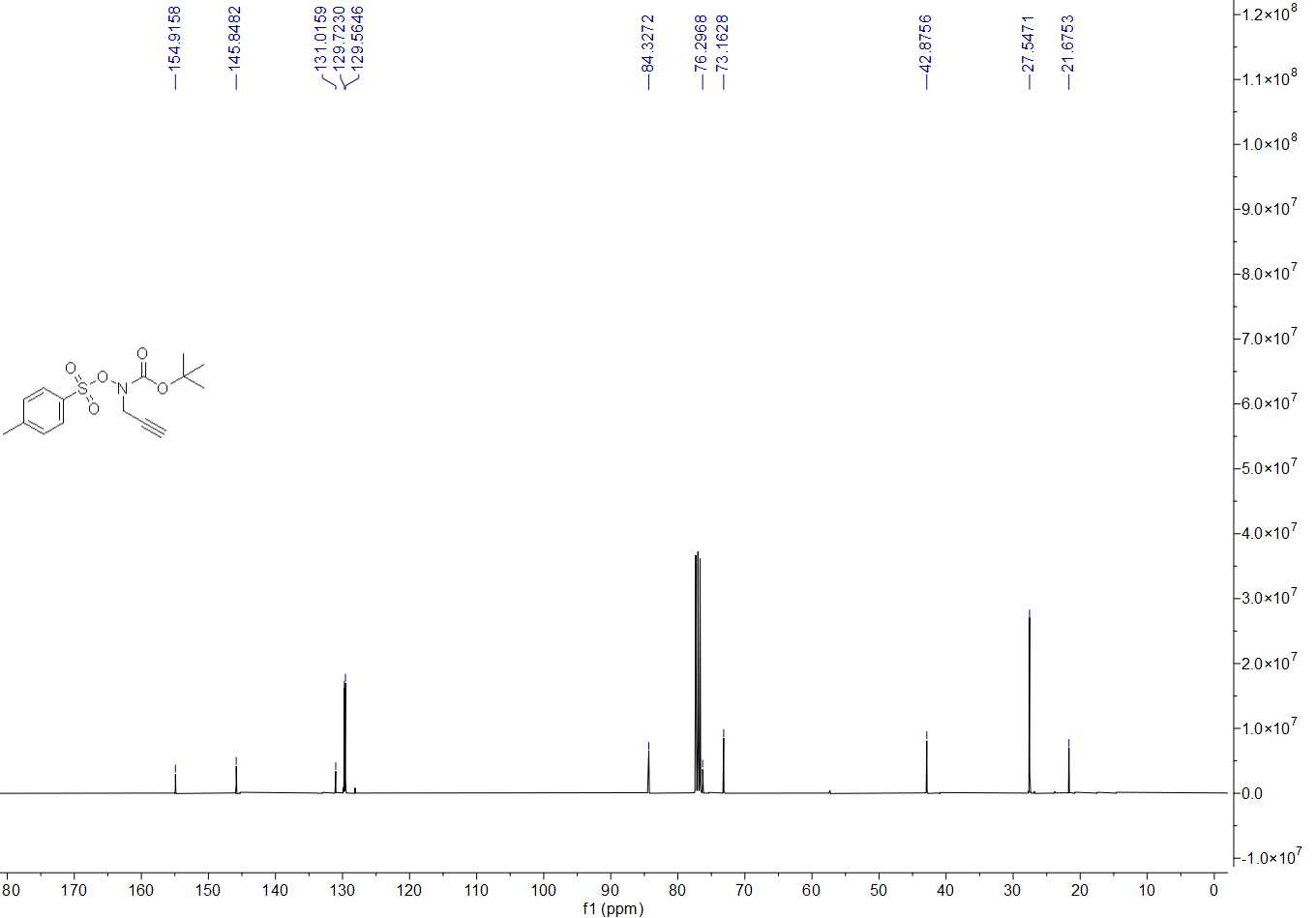


**Supplementary Fig 33.** ^1^H (upper part) and ^13^C NMR (lower part) of *tert*-butyl prop-2-yn-1-yl(tosyloxy)carbamate.


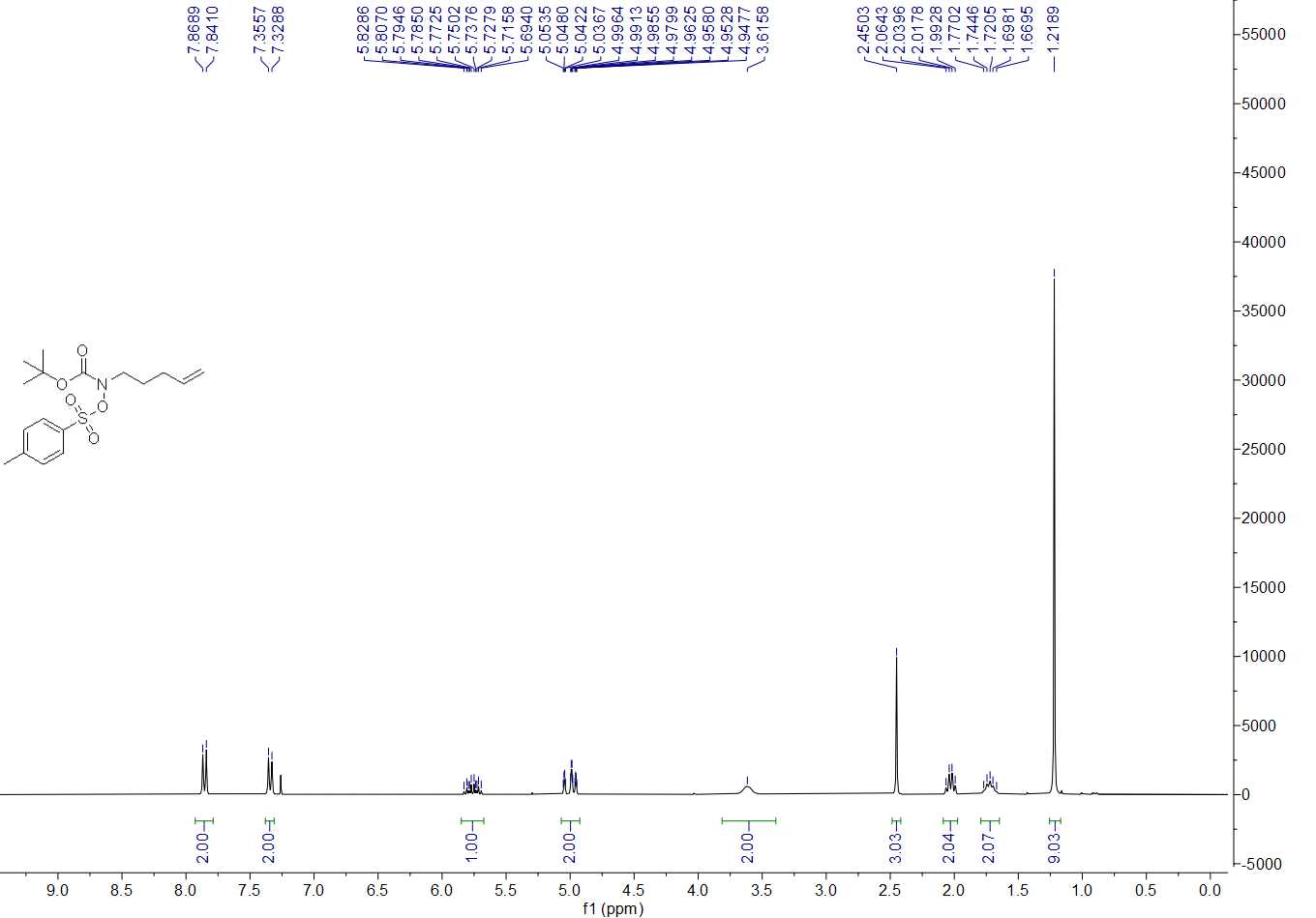


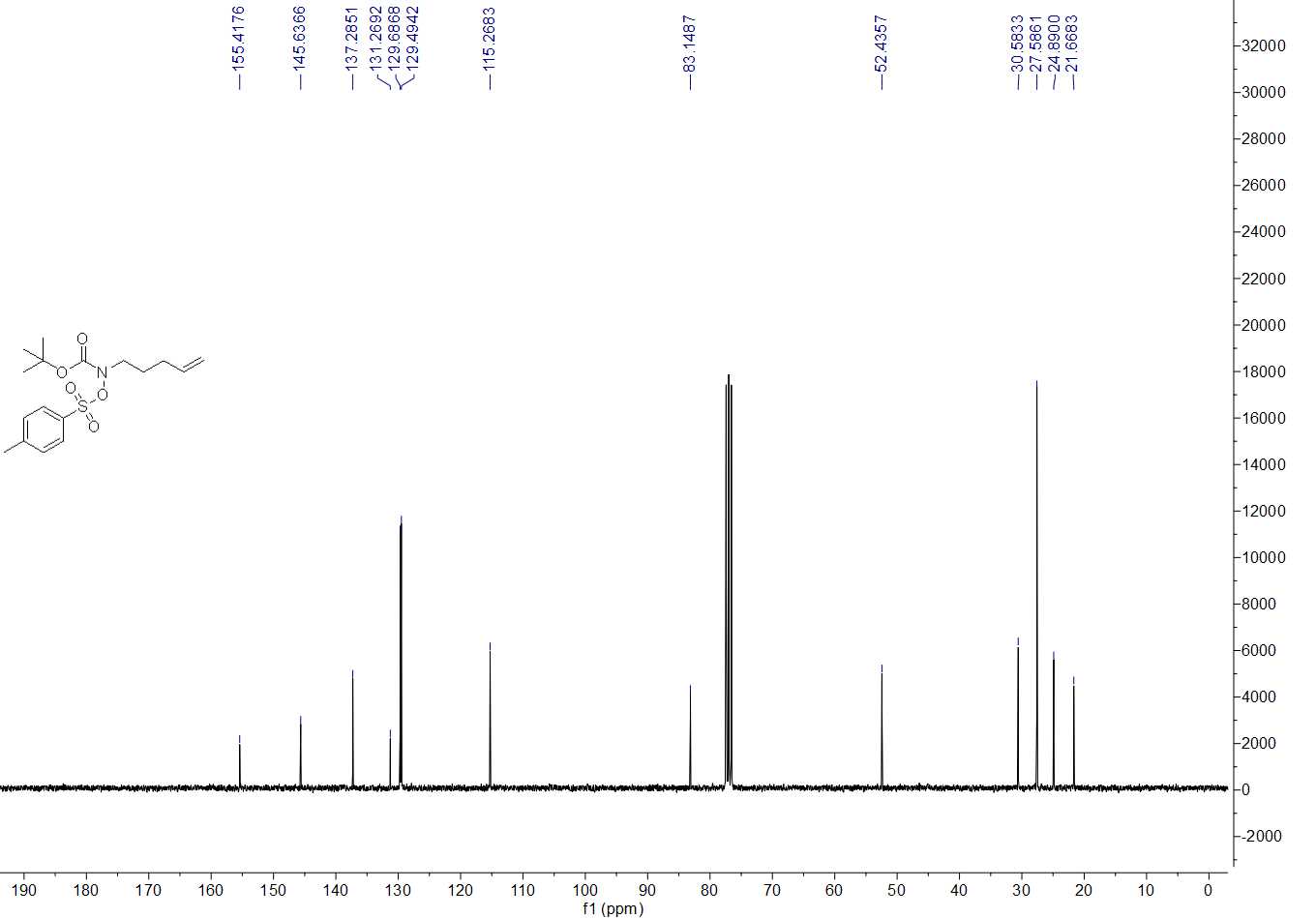


**Supplementary Fig 34.** ^1^H (upper part) and ^13^C NMR (lower part) of *tert*-butyl pent-4-en-1-yl(tosyloxy)carbamate.


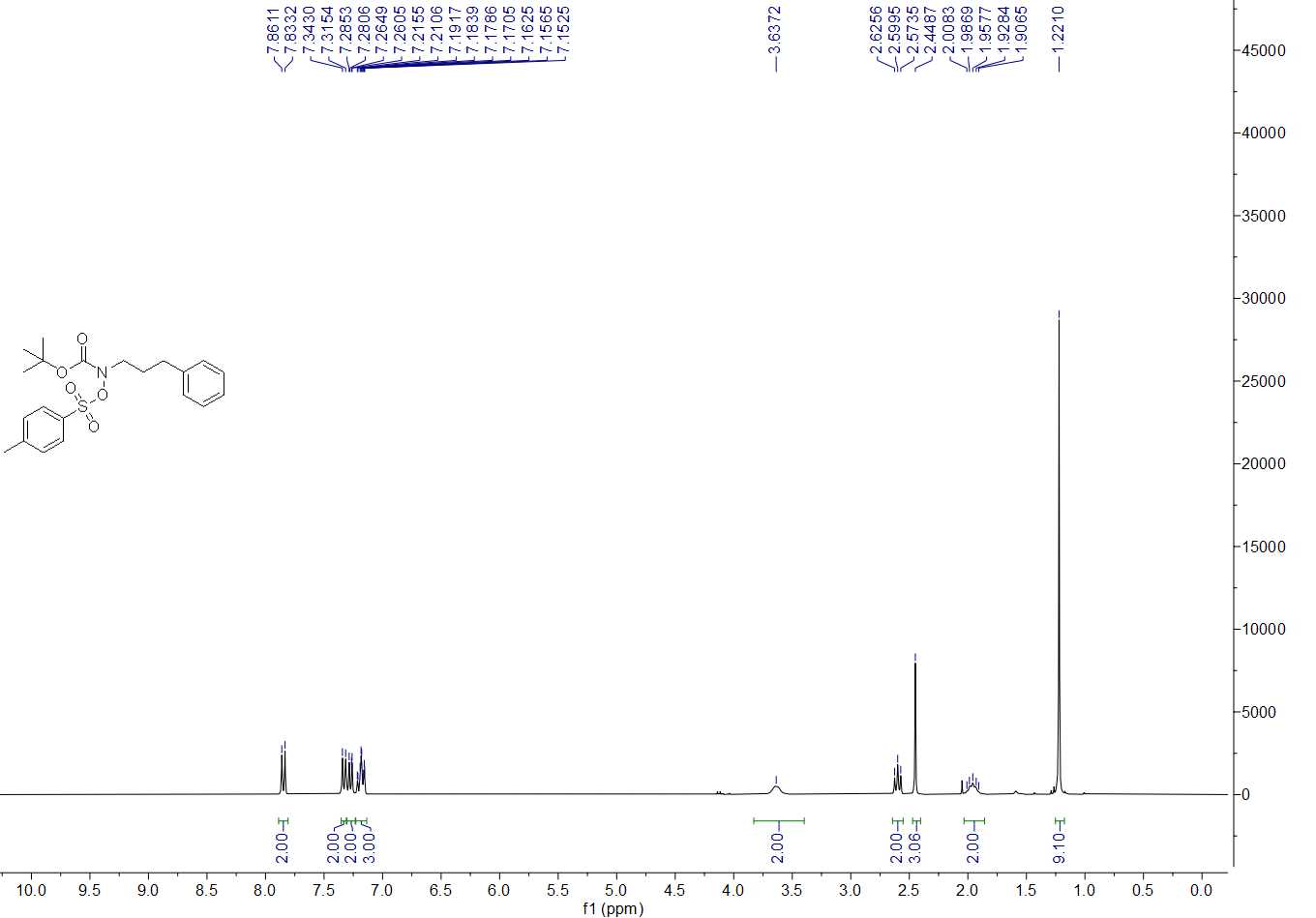


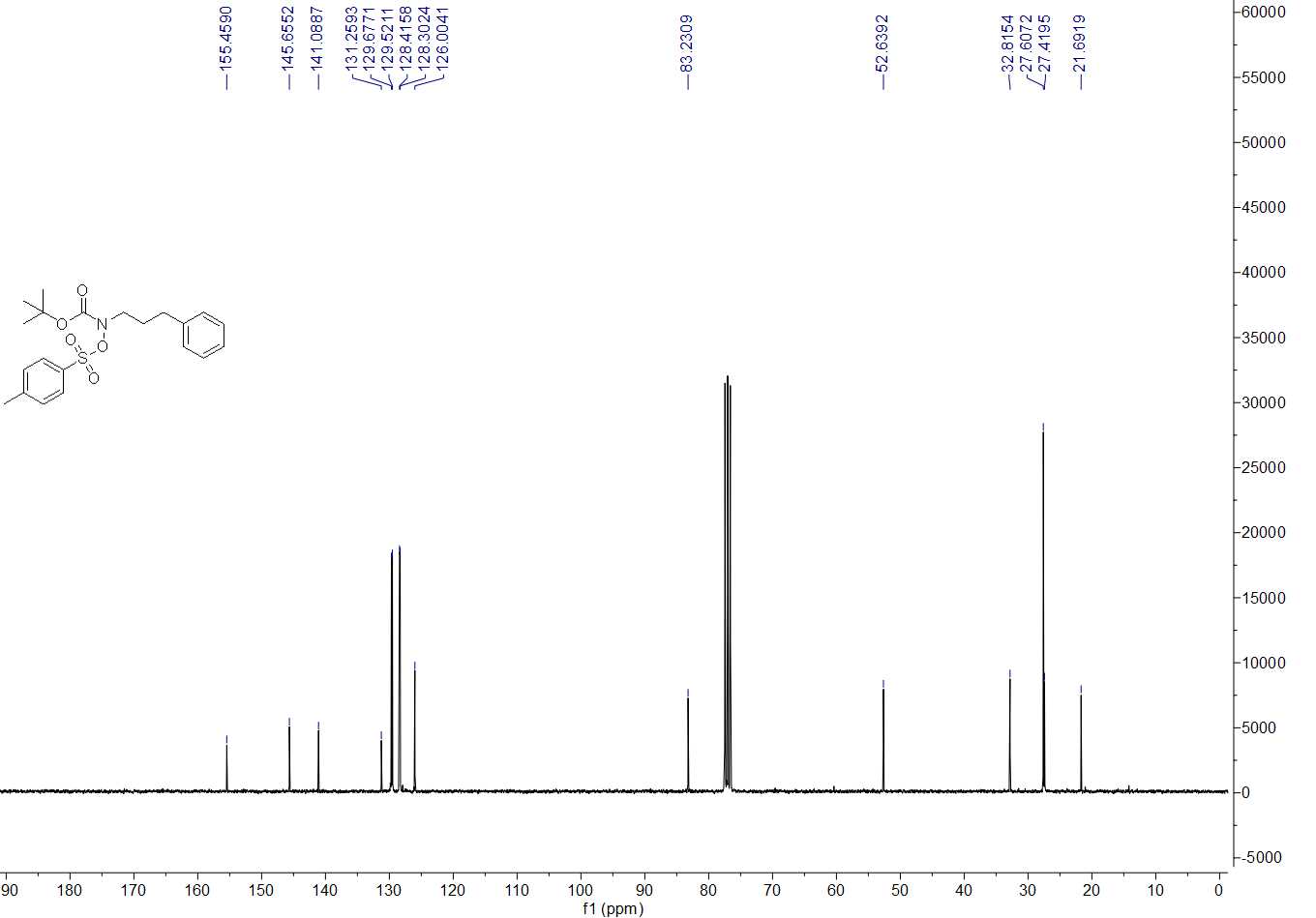


**Supplementary Fig 35.** ^1^H (upper part) and ^13^C NMR (lower part) of *tert*-butyl (3-phenylpropyl)(tosyloxy)carbamate.


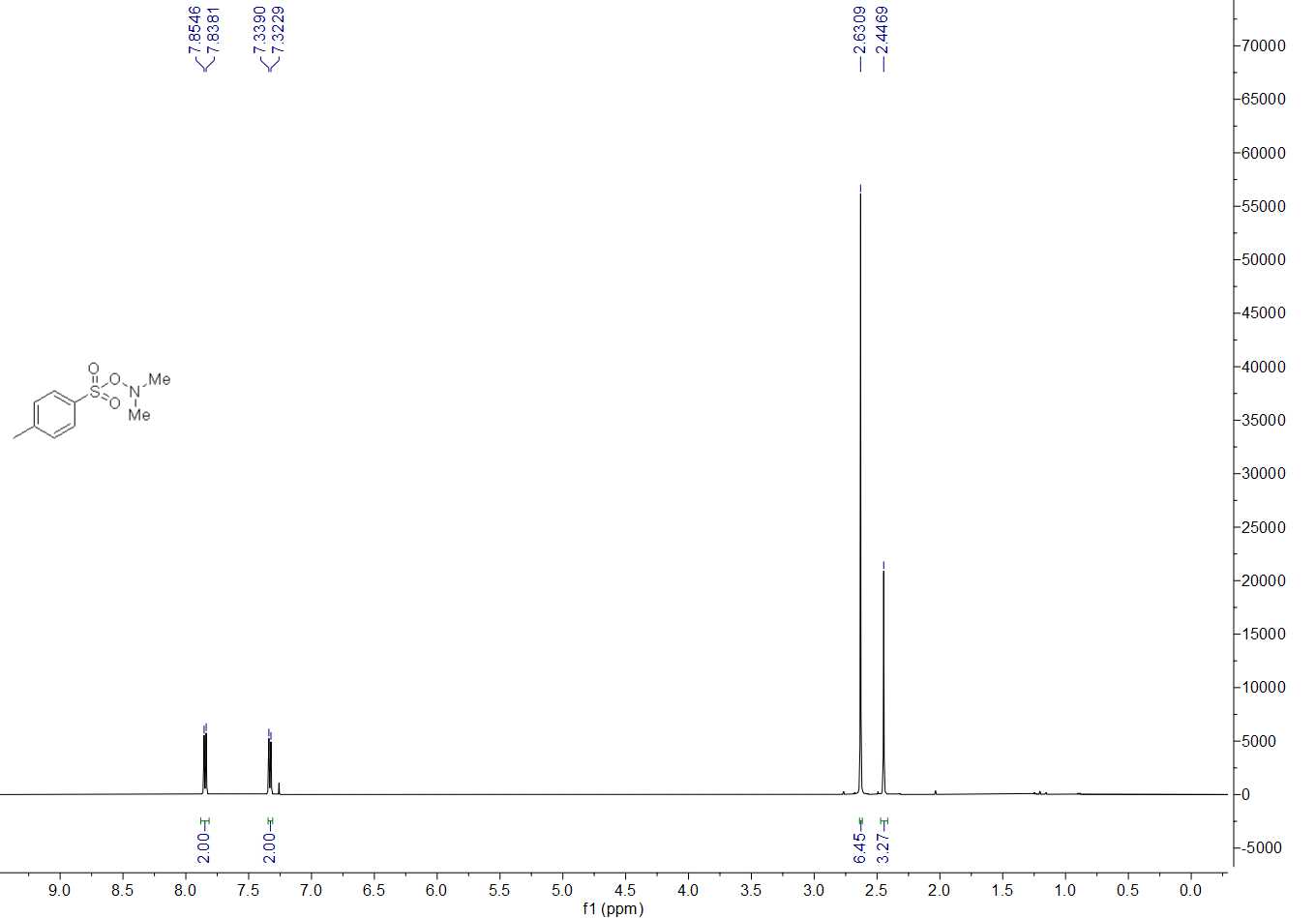


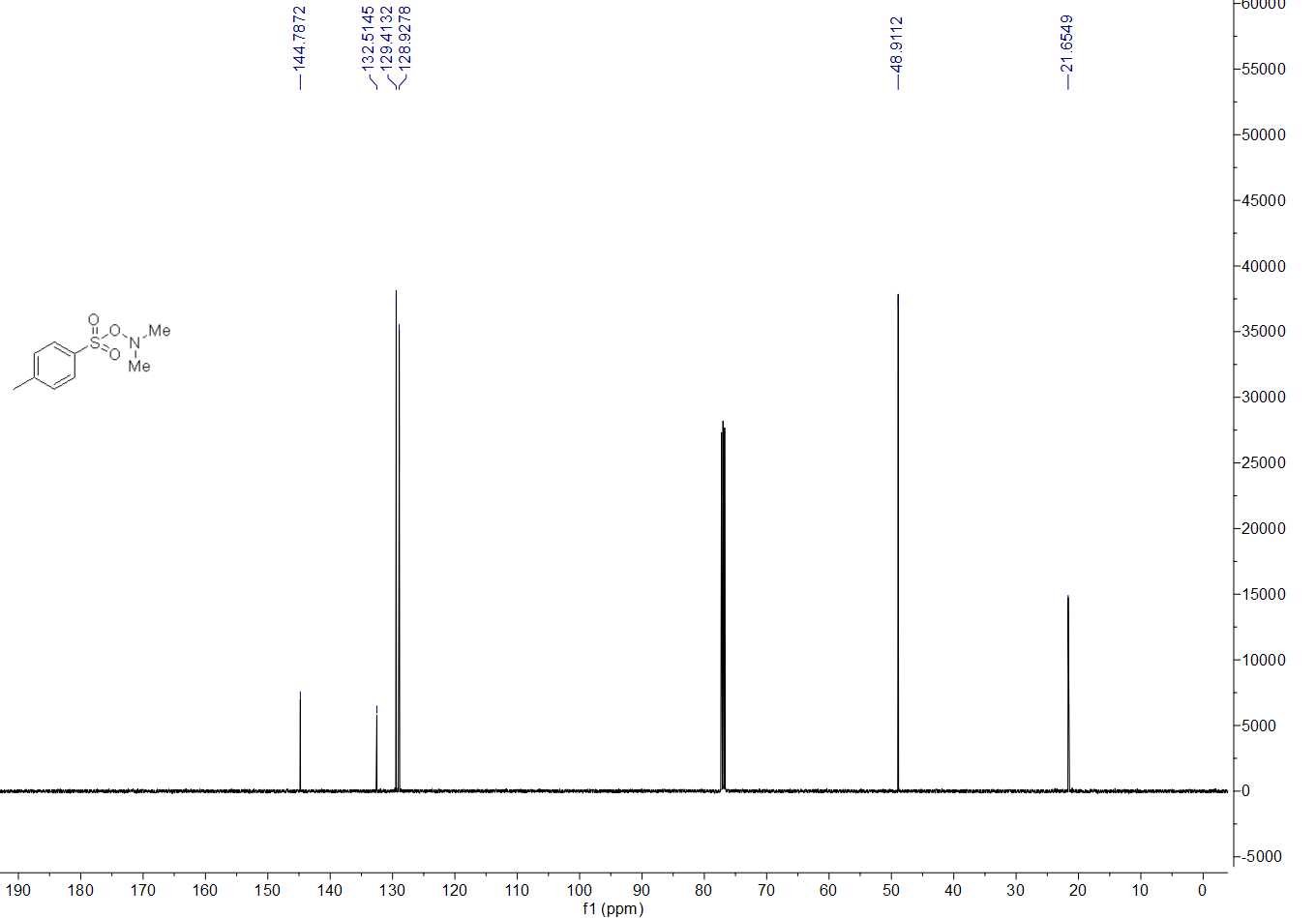


**Supplementary Fig 36.** ^1^H (upper part) and ^13^C NMR (lower part) of *N*, *N*-dimethyl-*O*-tosylhydroxylamine.


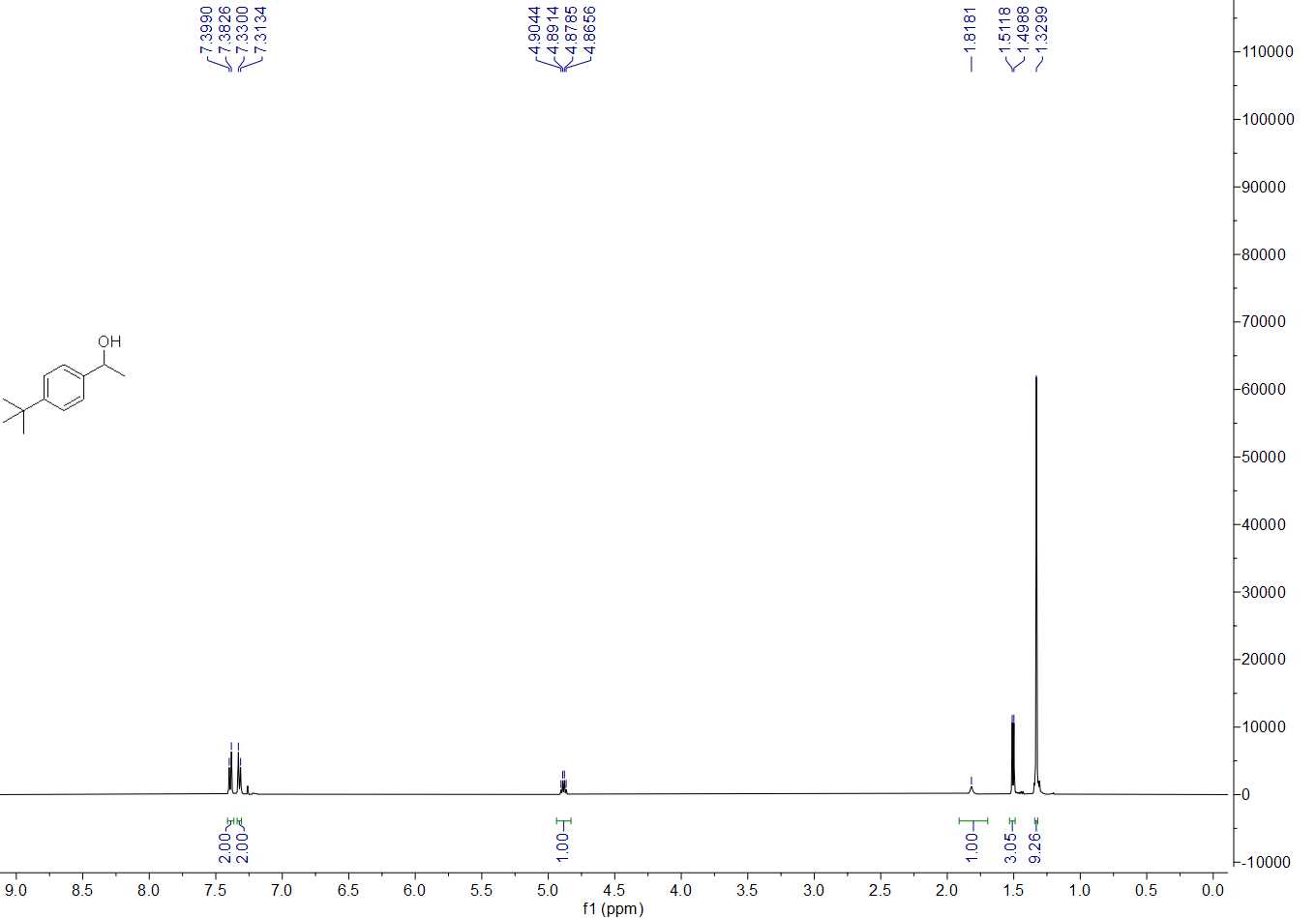


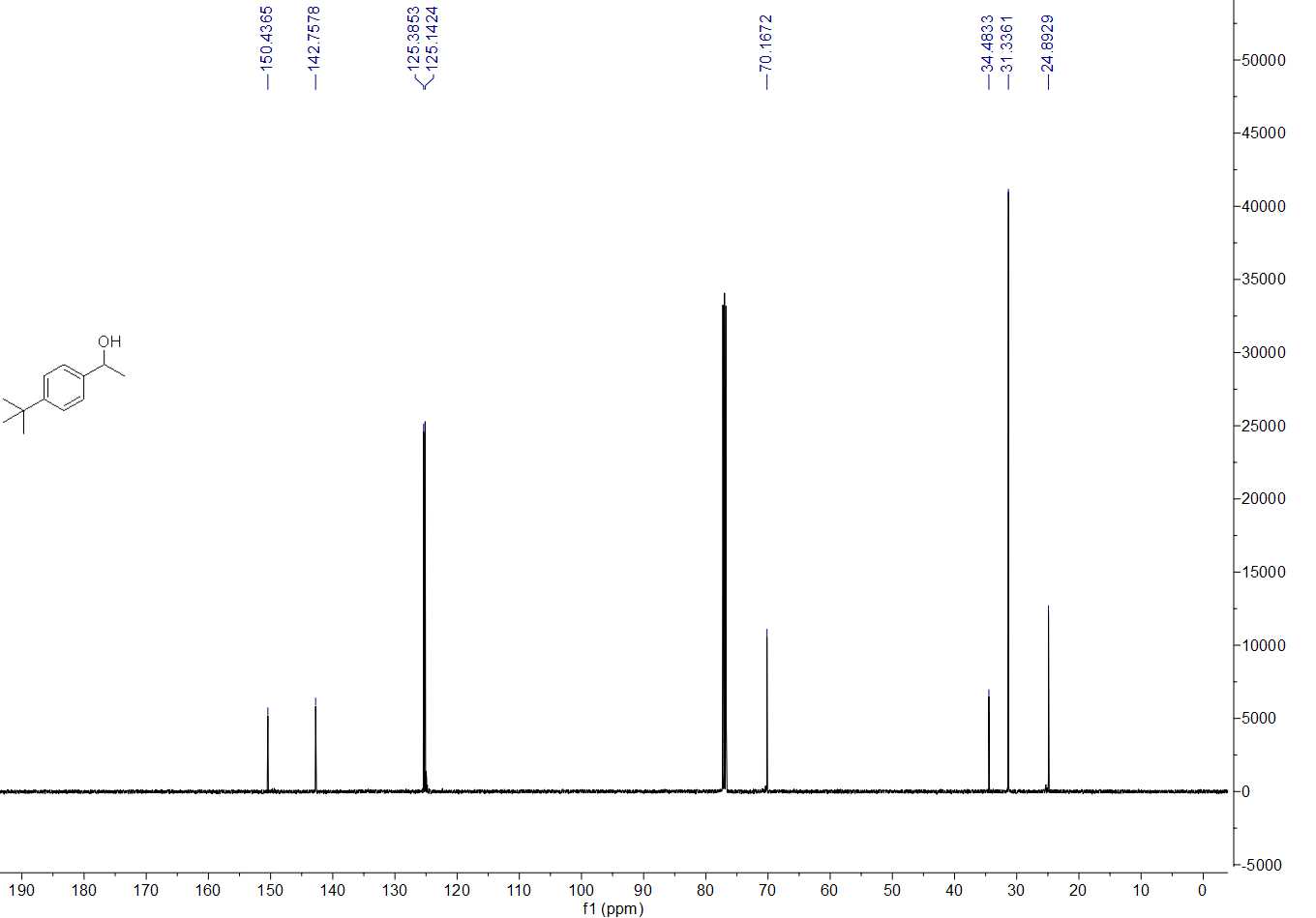


**Supplementary Fig 37.** ^1^H (upper part) and ^13^C NMR (lower part) of **1d**.


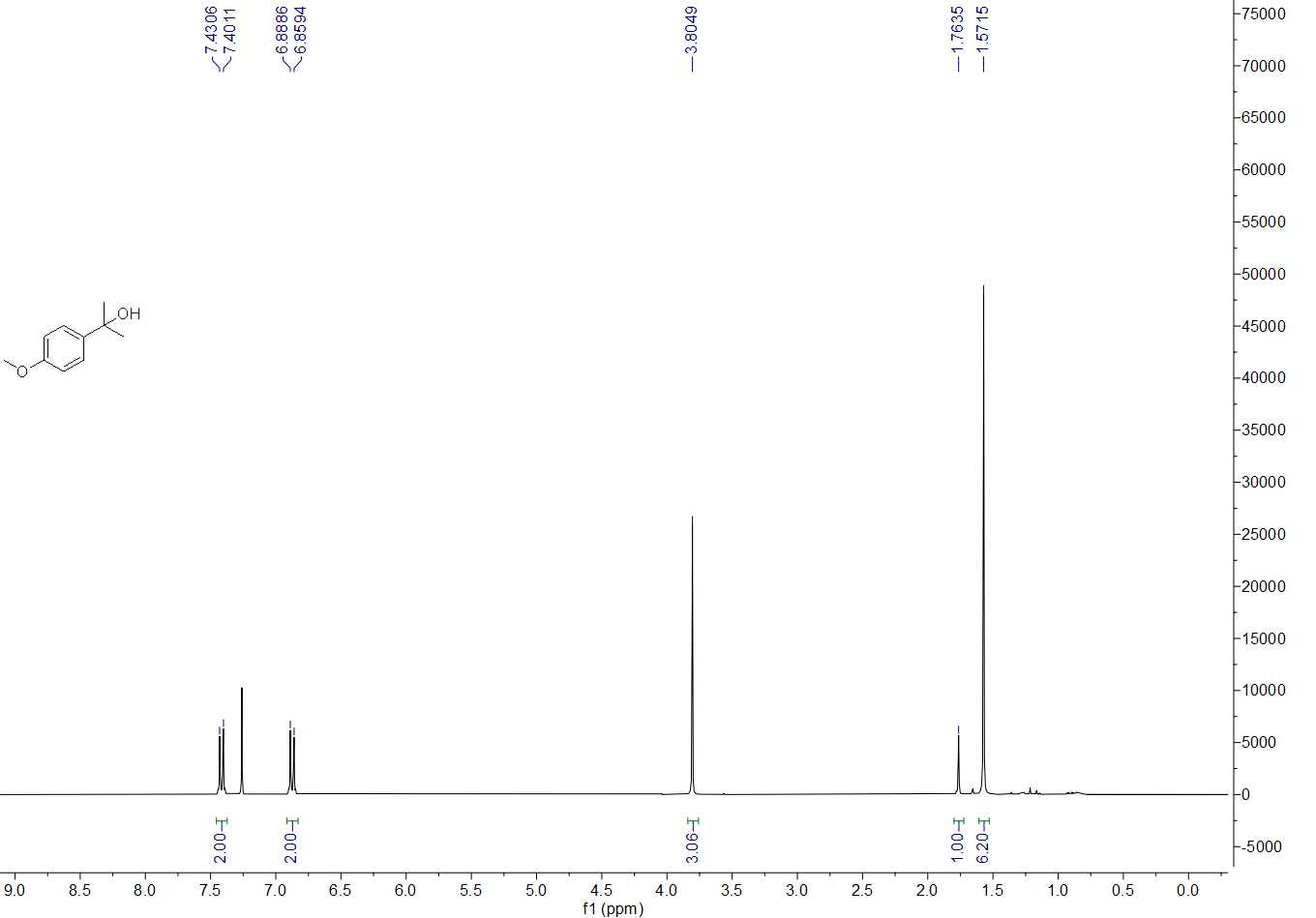


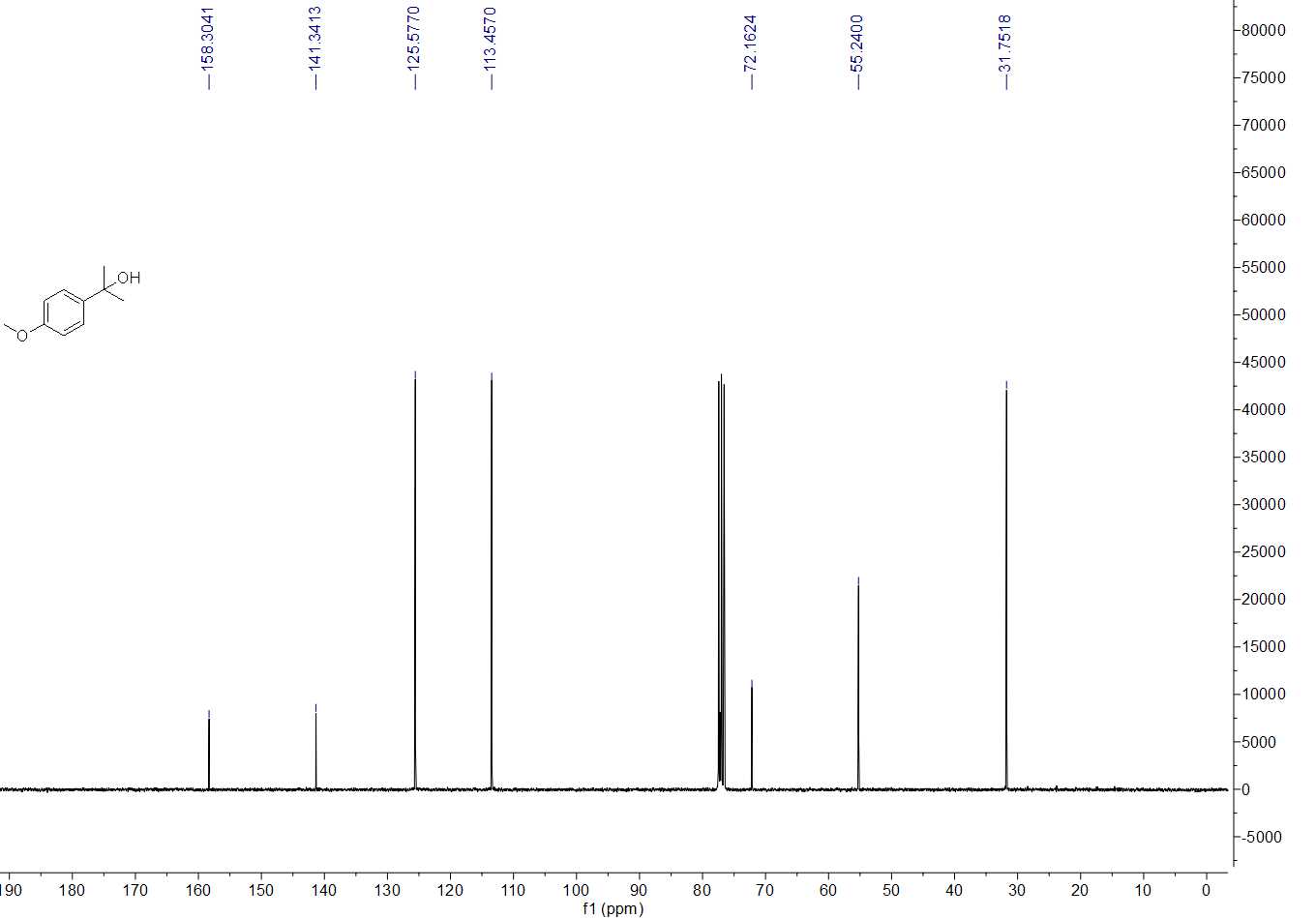


**Supplementary Fig 38.** ^1^H (upper part) and ^13^C NMR (lower part) of **1e**.


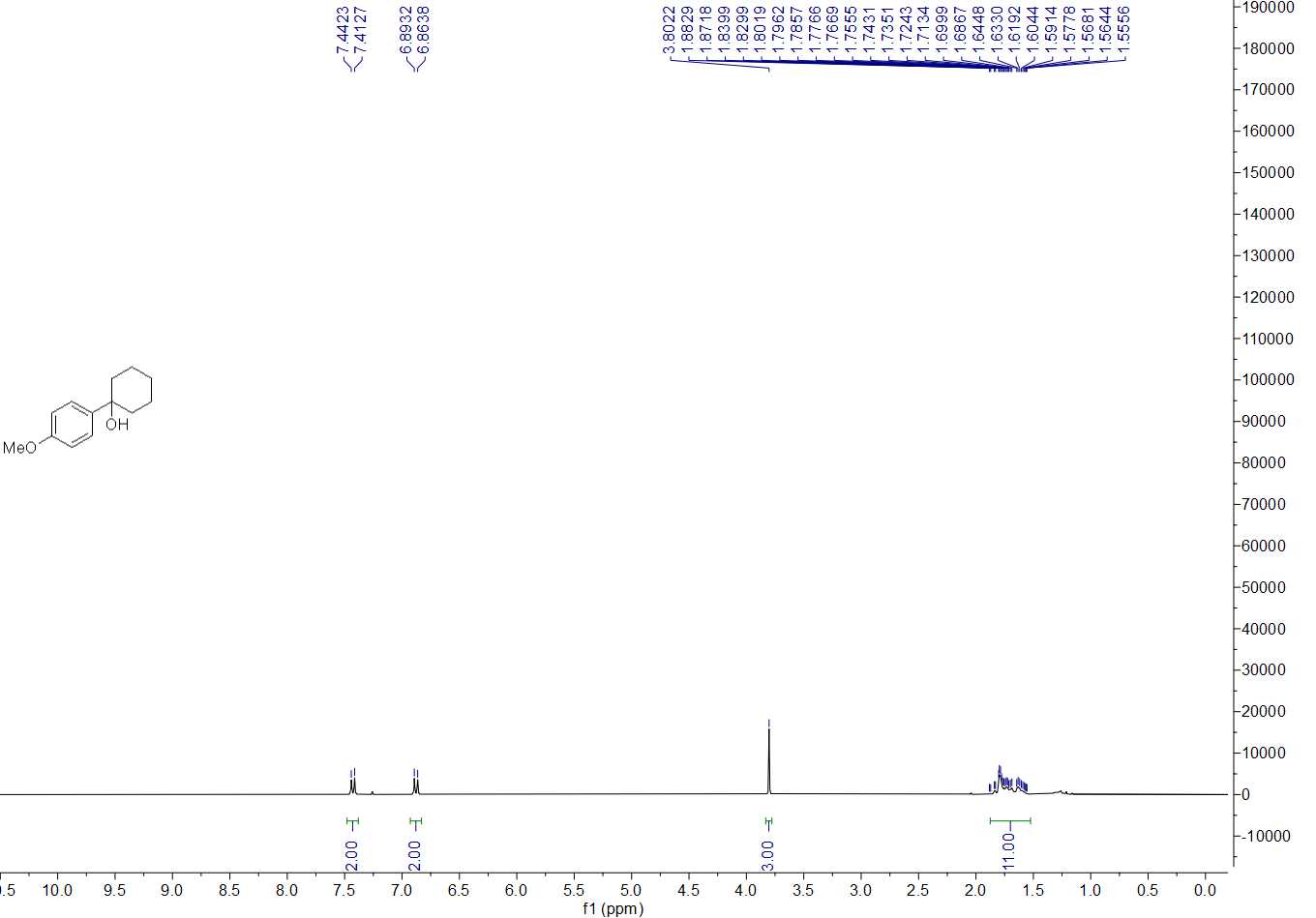


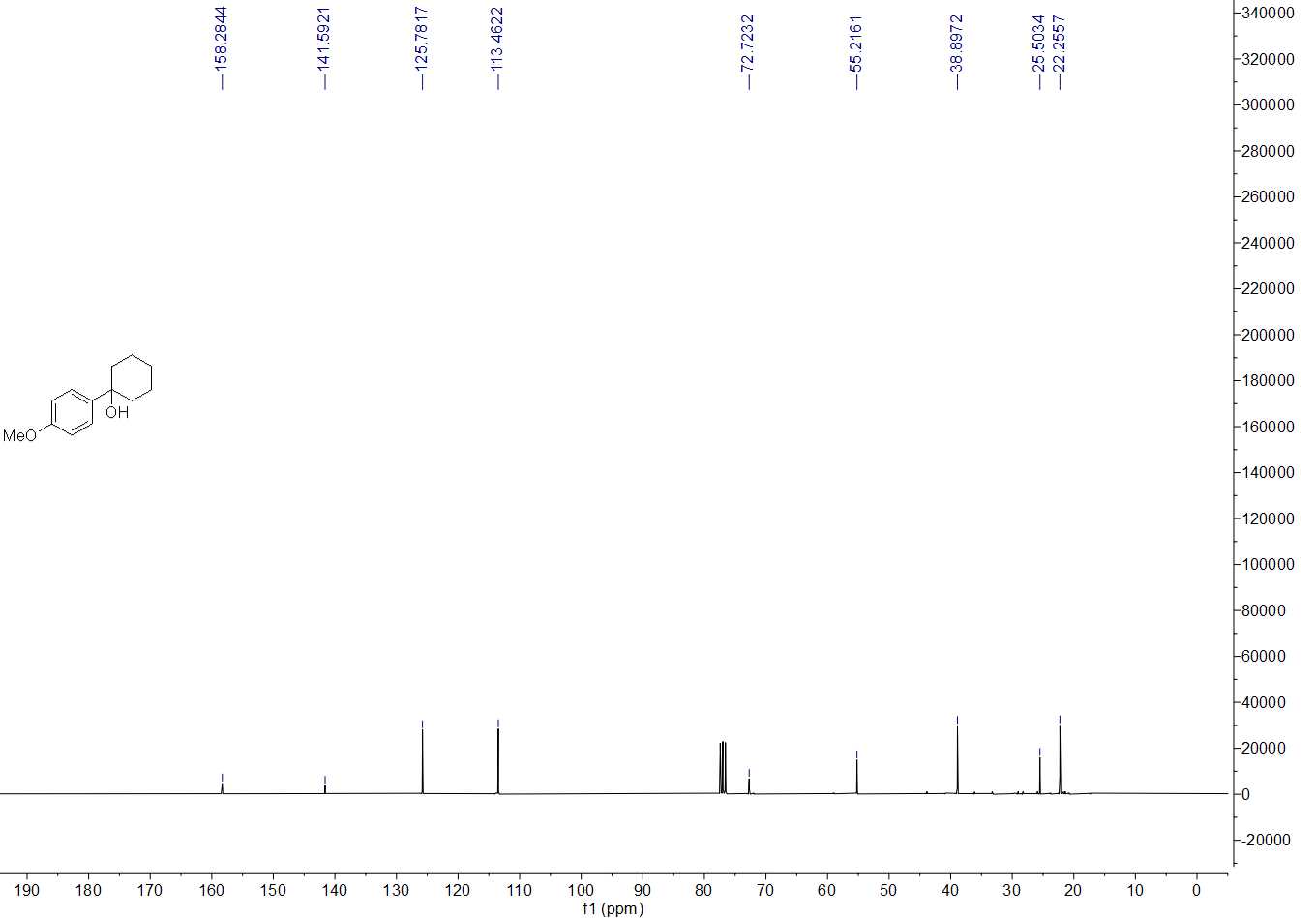


**Supplementary Fig 39.** ^1^H (upper part) and ^13^C NMR (lower part) of **1f**.


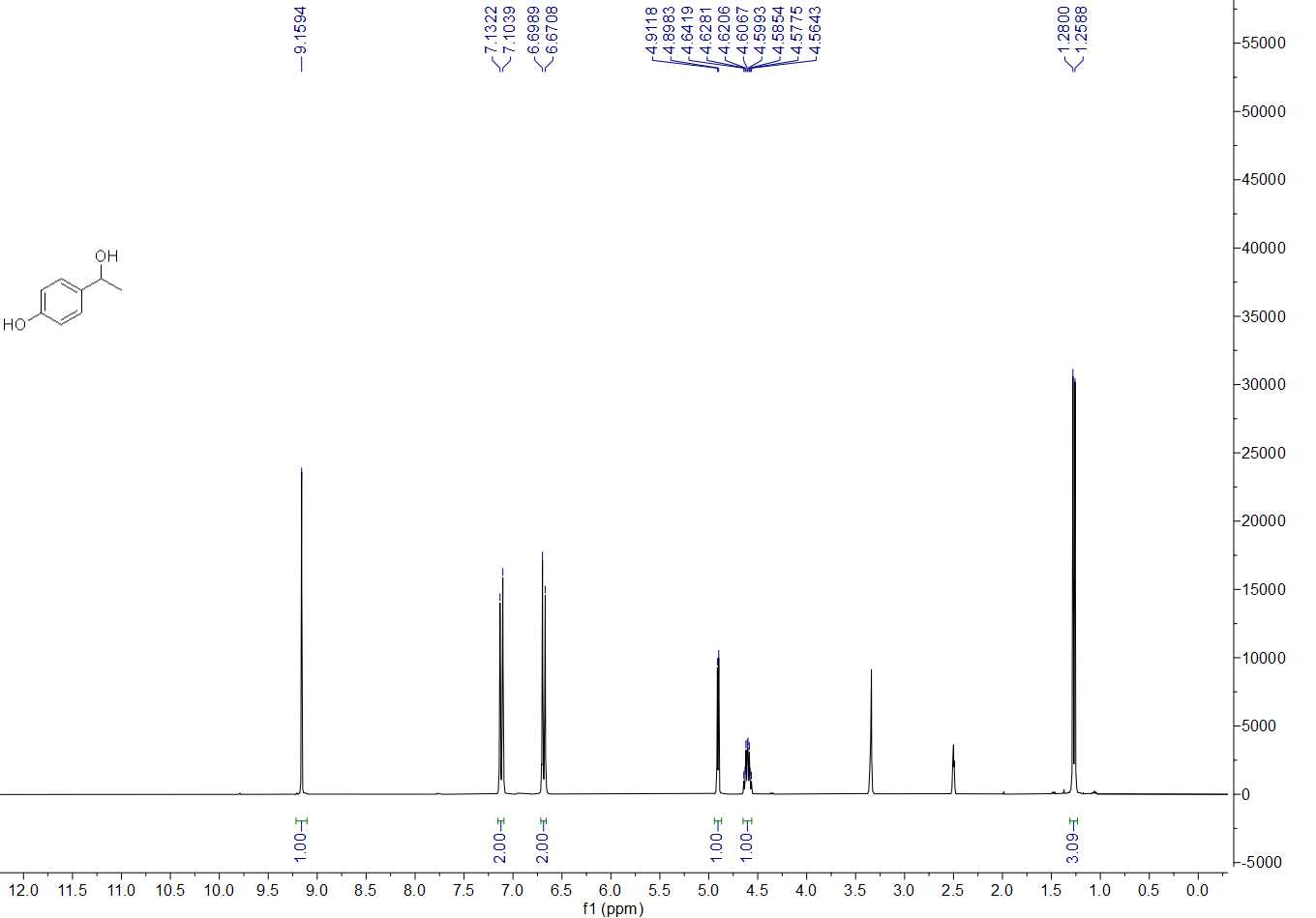


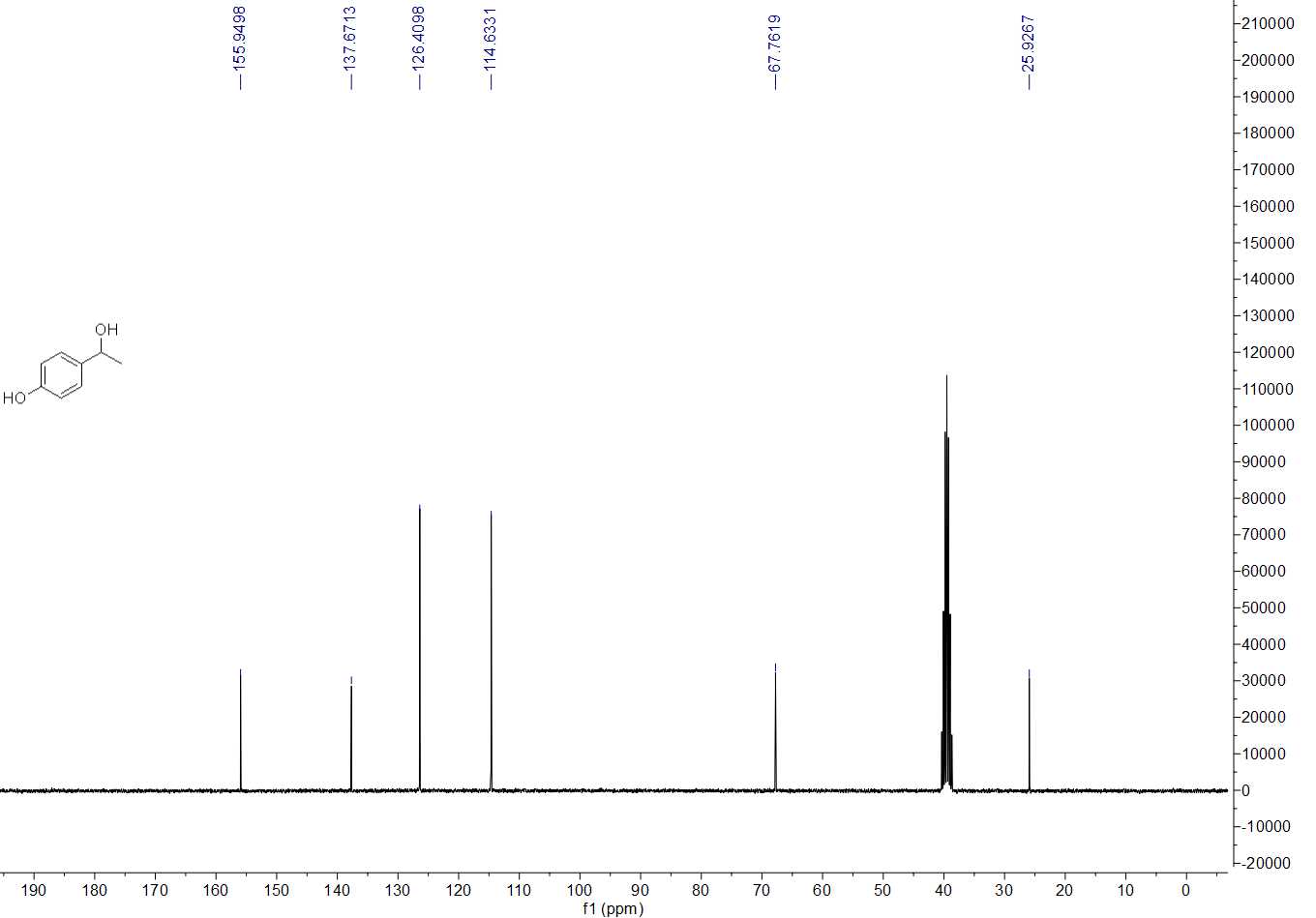


**Supplementary Fig 40.** ^1^H (upper part) and ^13^C NMR (lower part) of **1g.**


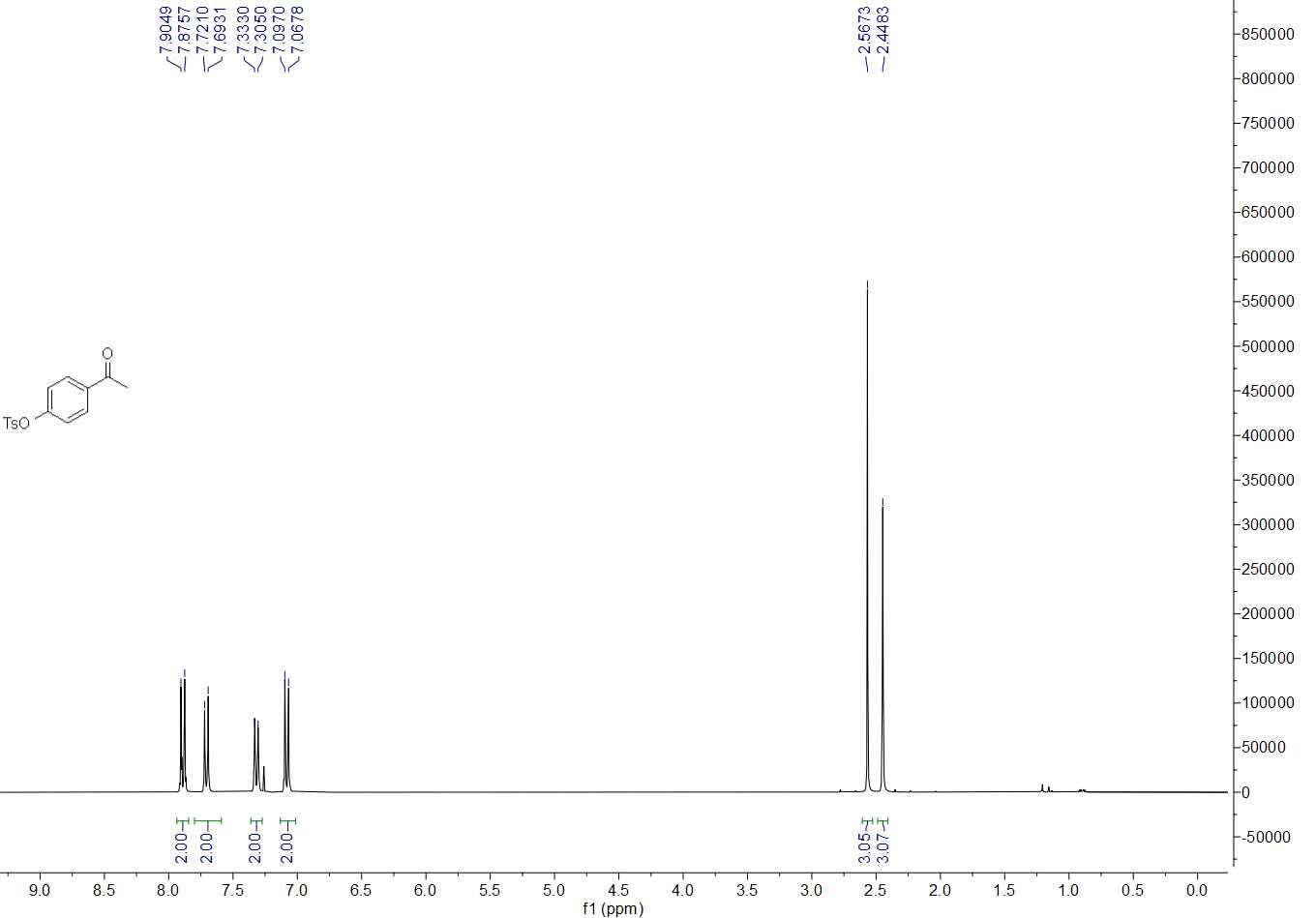


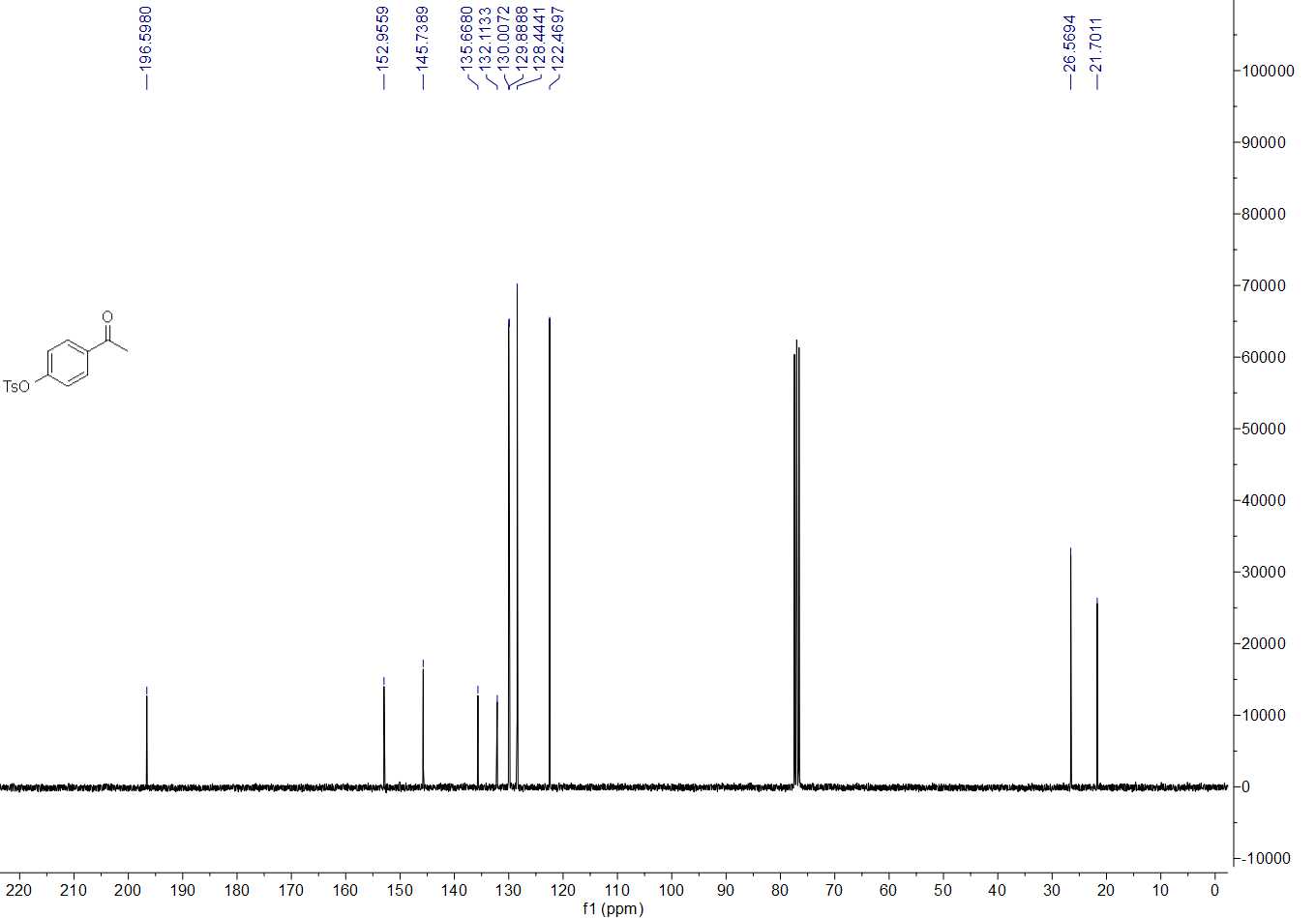


**Supplementary Fig 41.** ^1^H (upper part) and ^13^C NMR (lower part) of 4-Acetylphenyl 4-methylbenzenesulfonate.


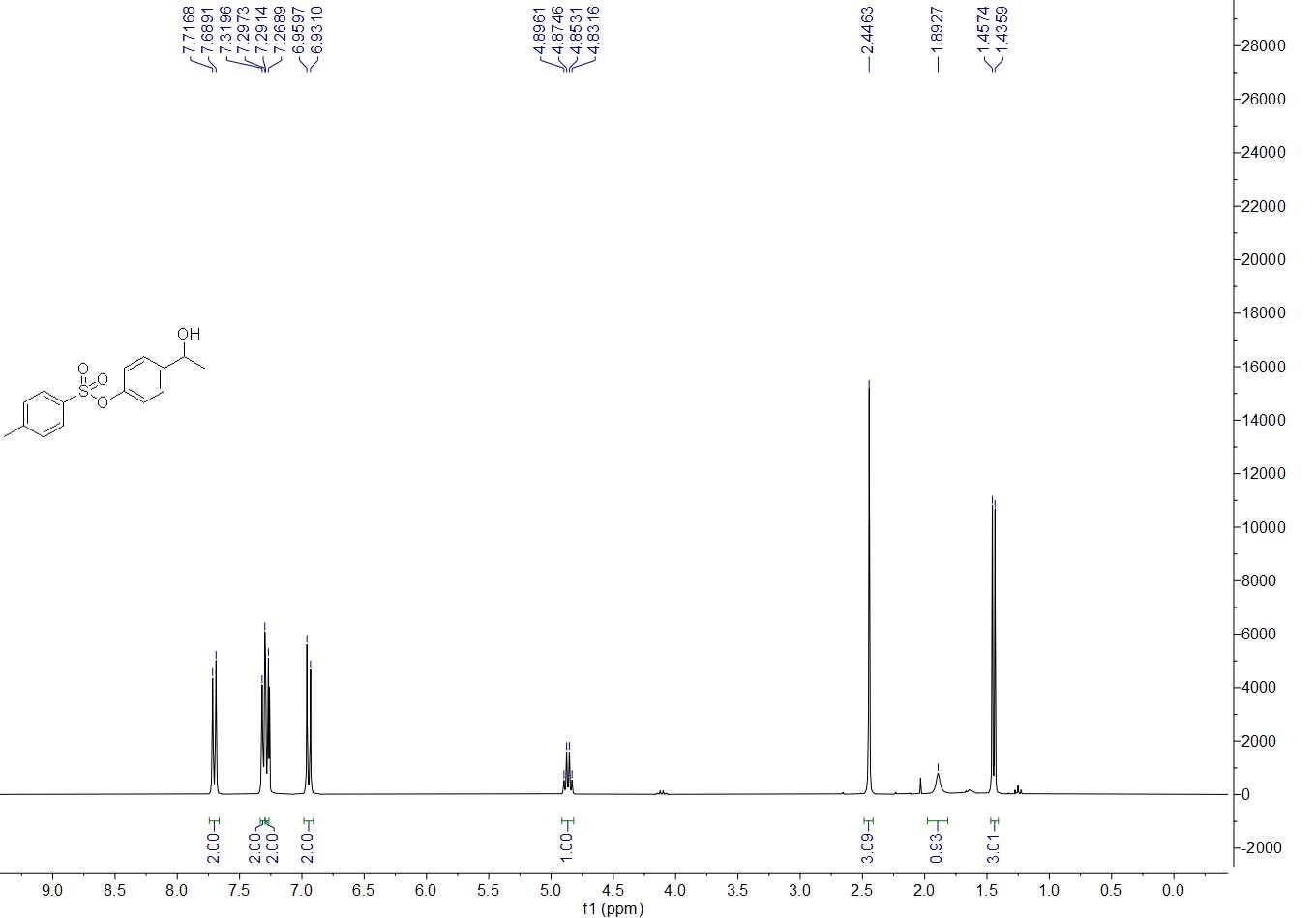


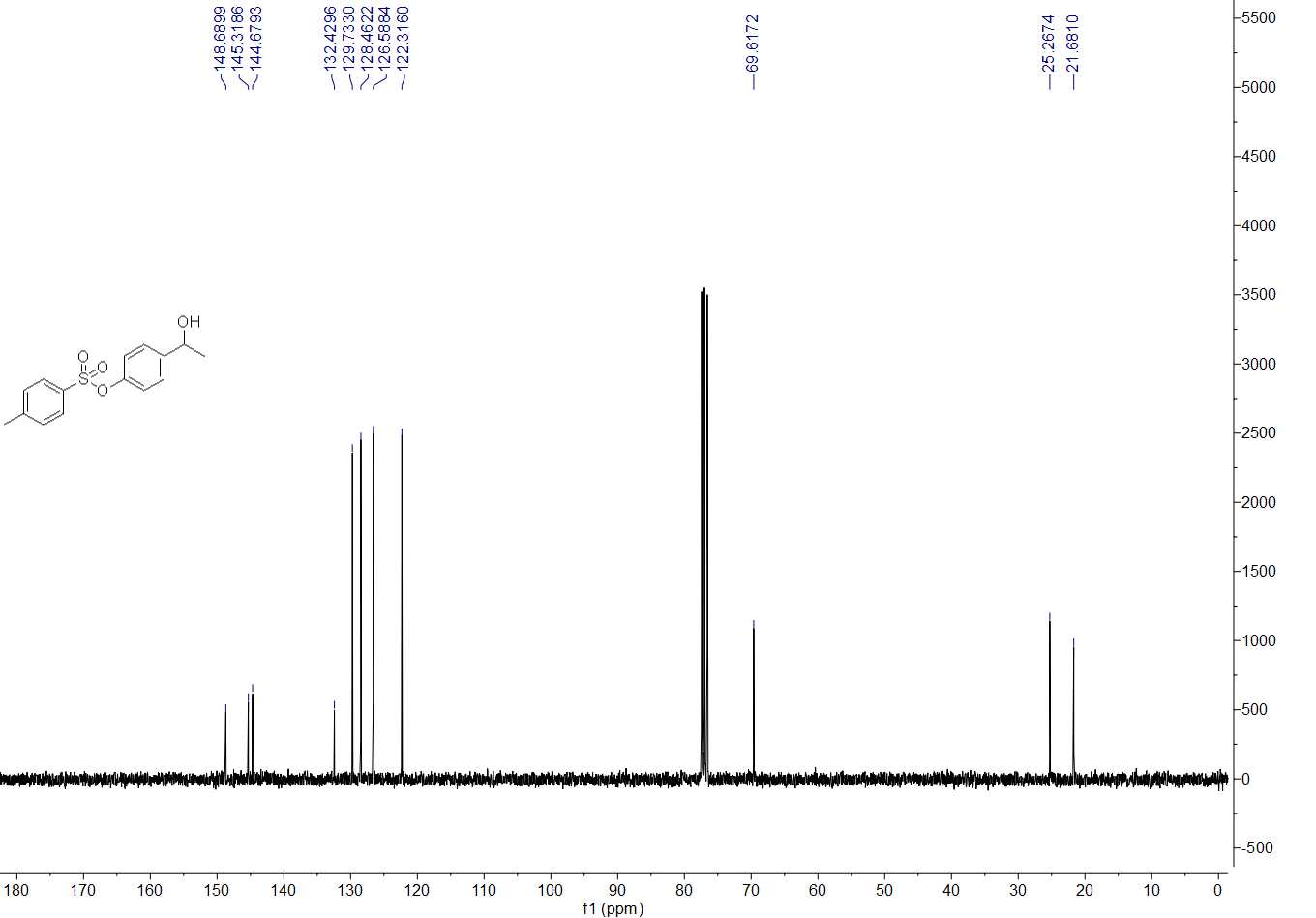


**Supplementary Fig 42.** ^1^H (upper part) and ^13^C NMR (lower part) of **1i**.


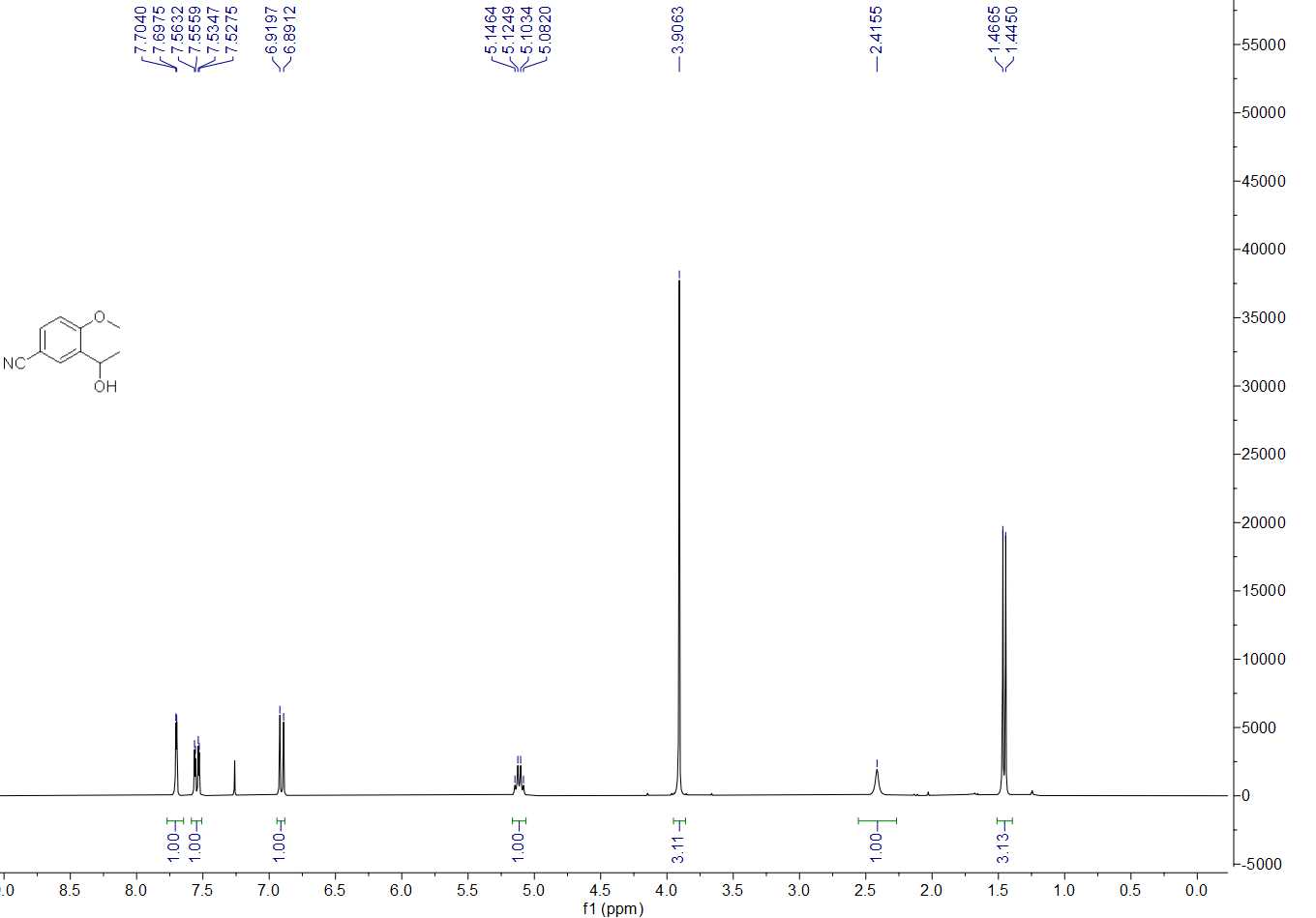


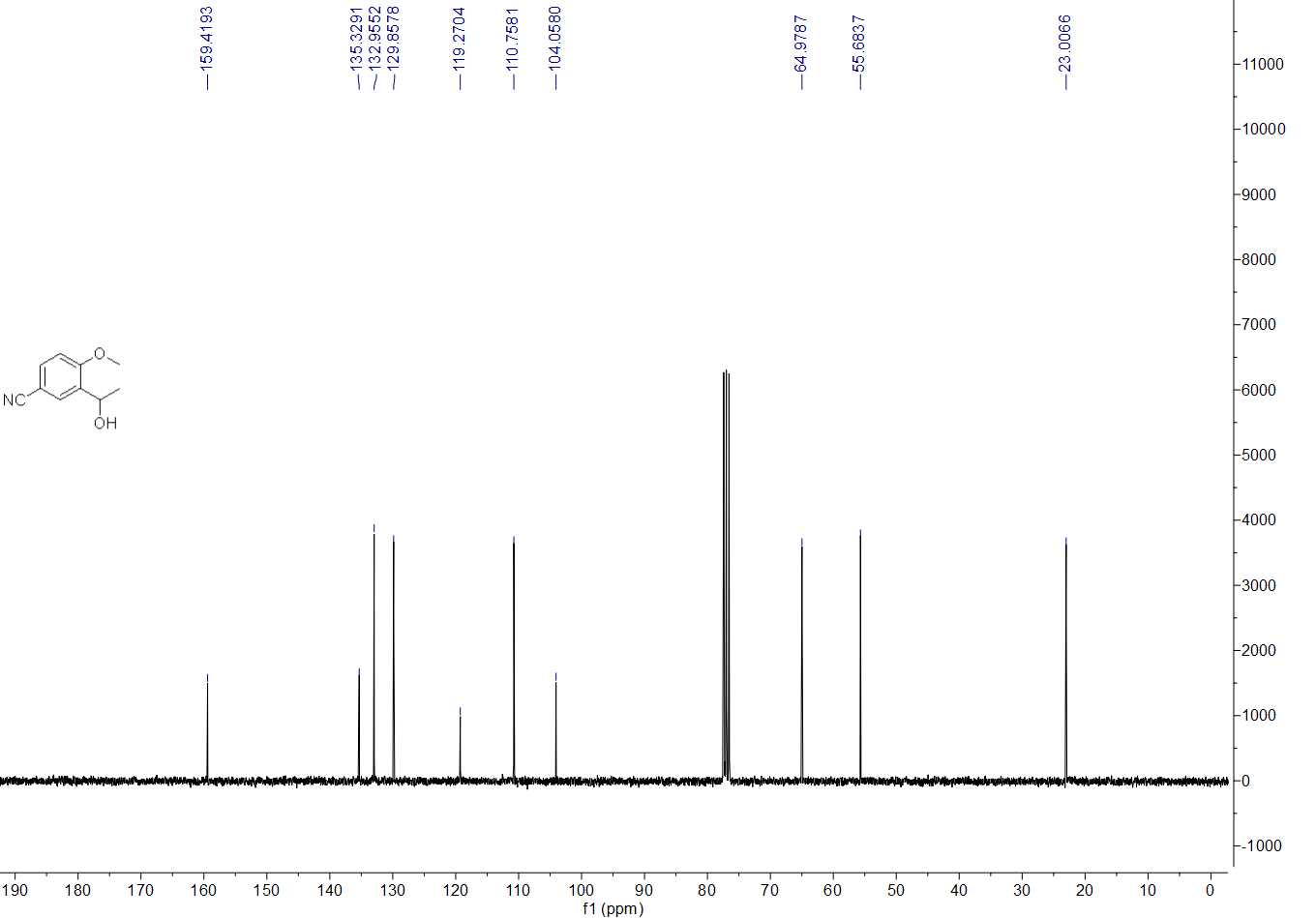


**Supplementary Fig 43.** ^1^H (upper part) and ^13^C NMR (lower part) of **1k**.


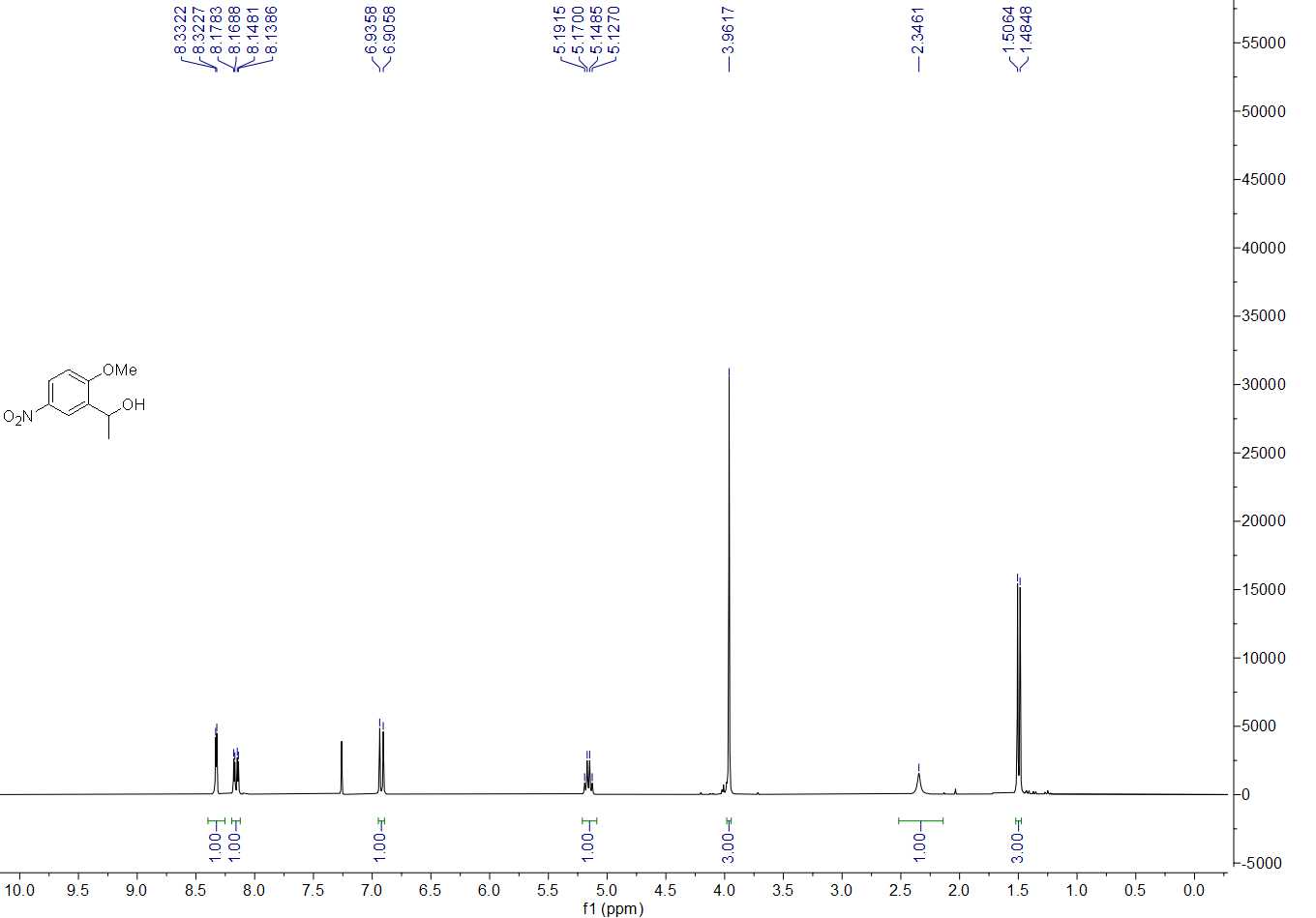


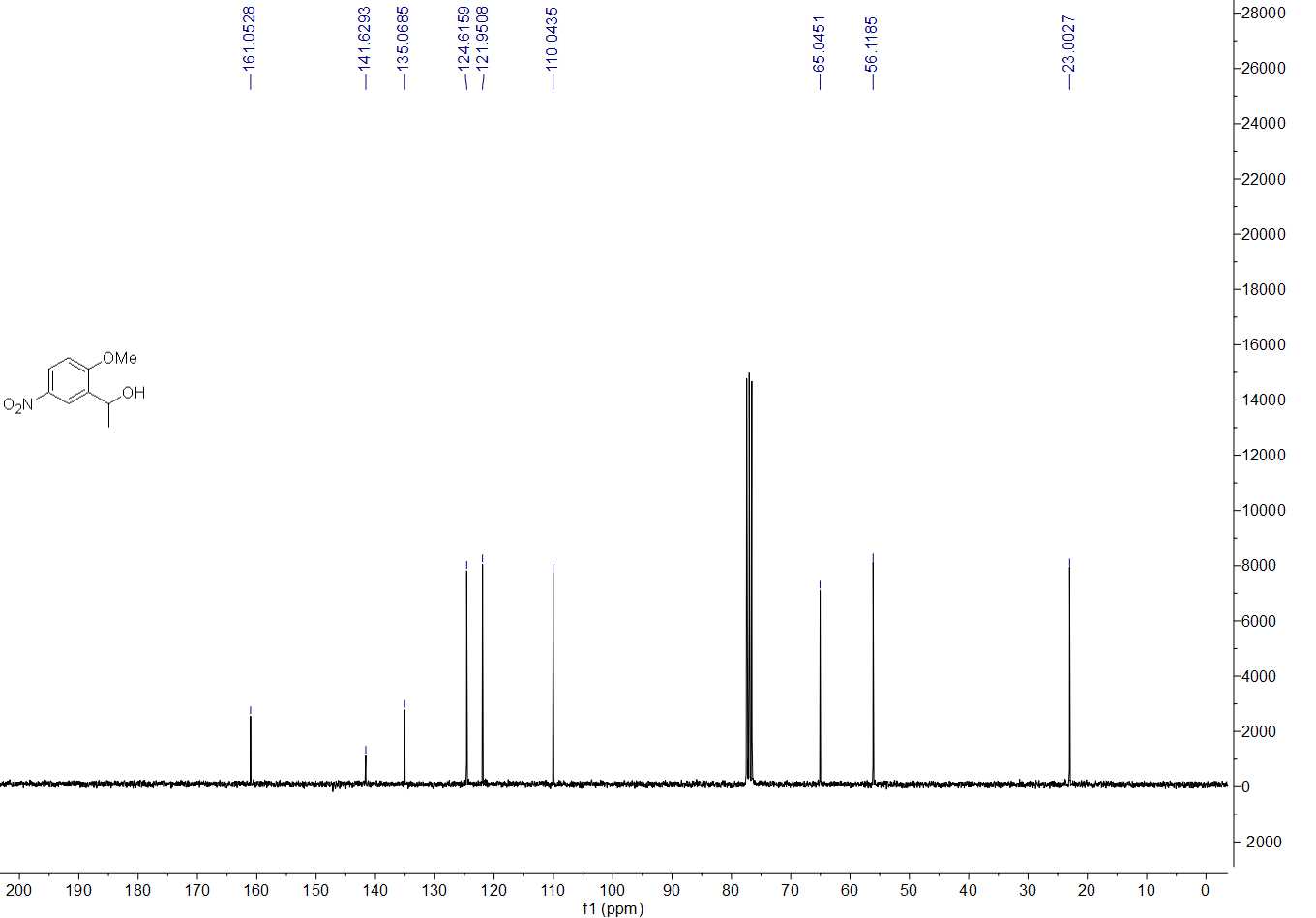


**Supplementary Fig 44.** ^1^H (upper part) and ^13^C NMR (lower part) of **1l**.


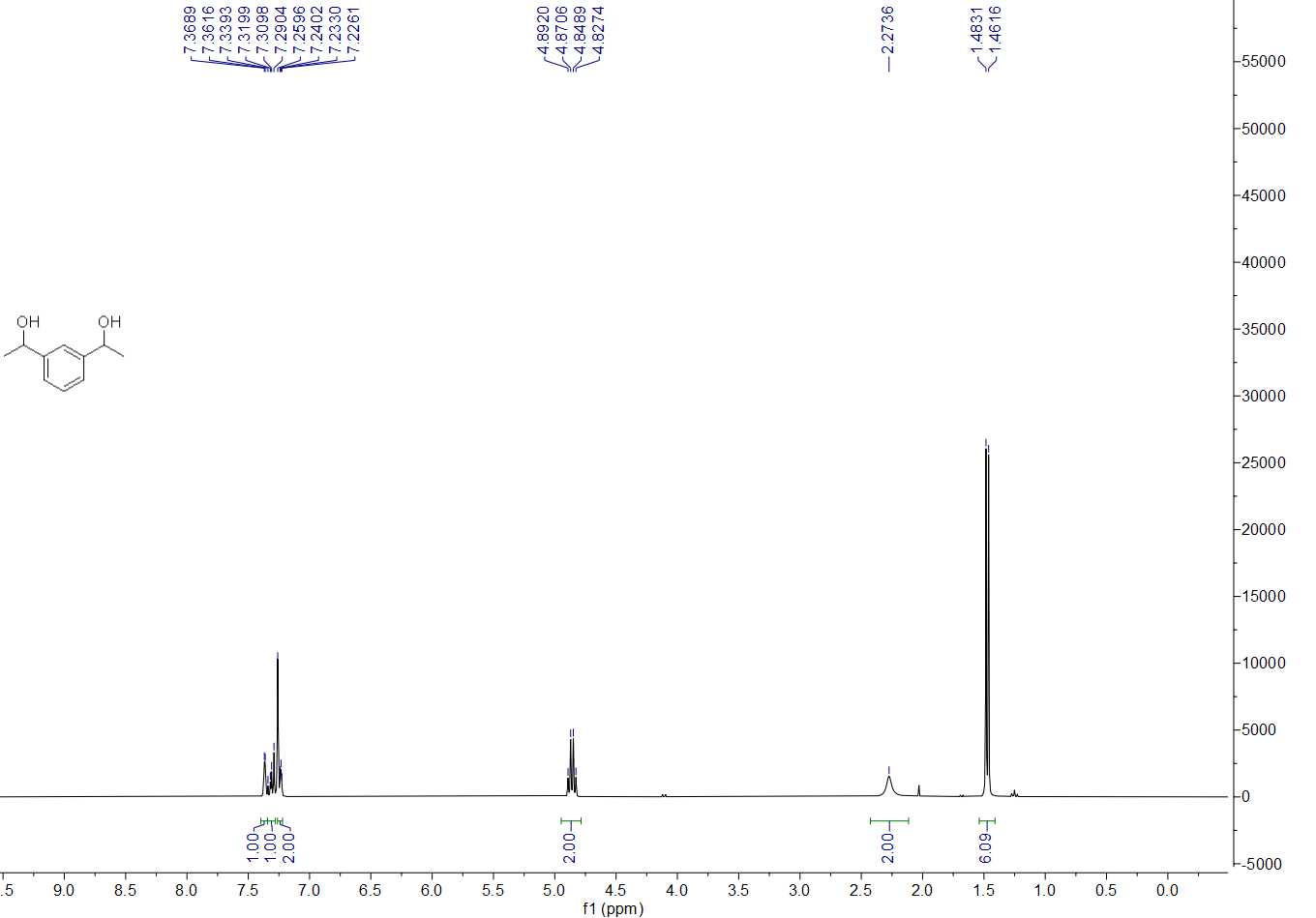


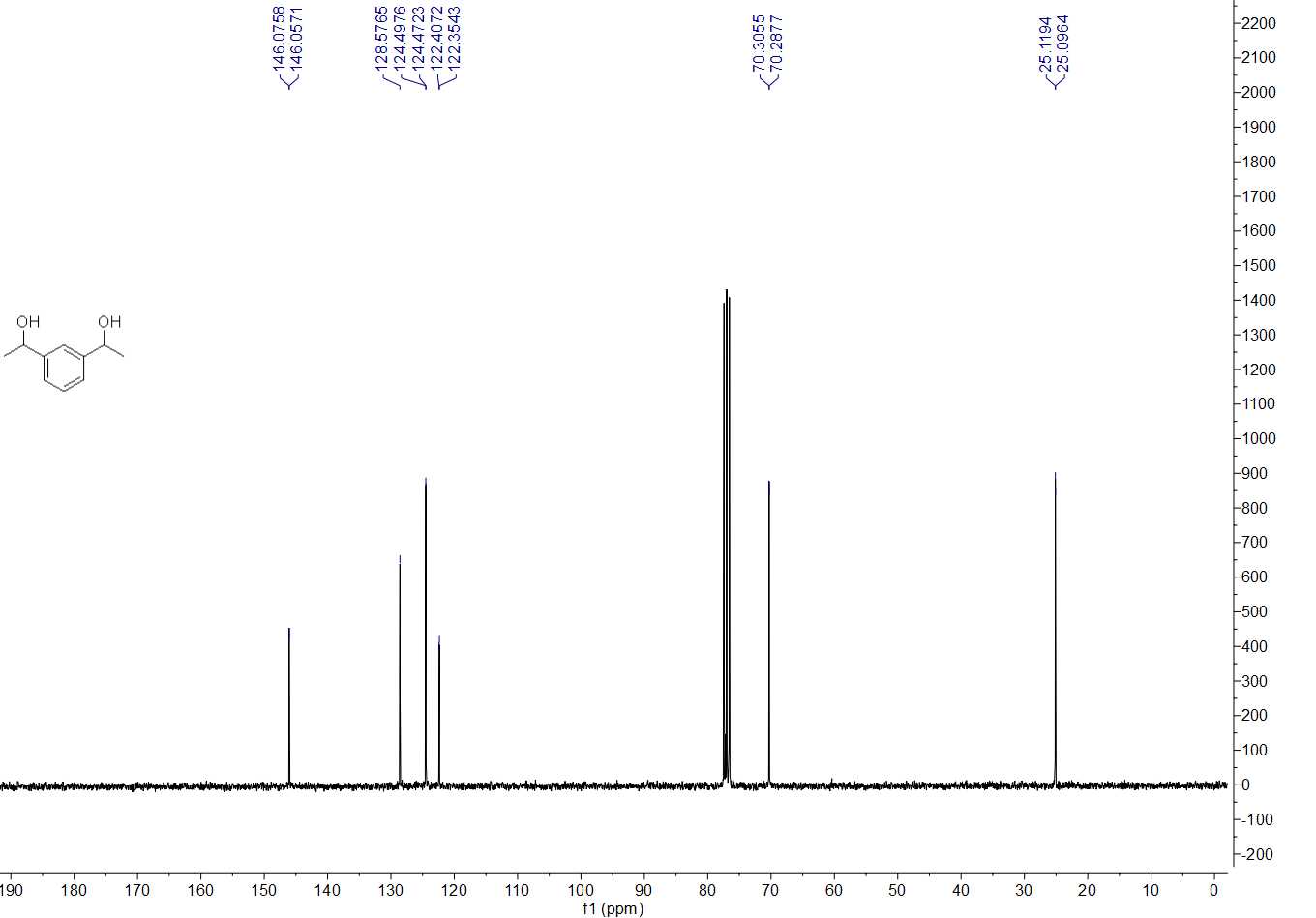


**Supplementary Fig 45.** ^1^H (upper part) and ^13^C NMR (lower part) of **1m**.


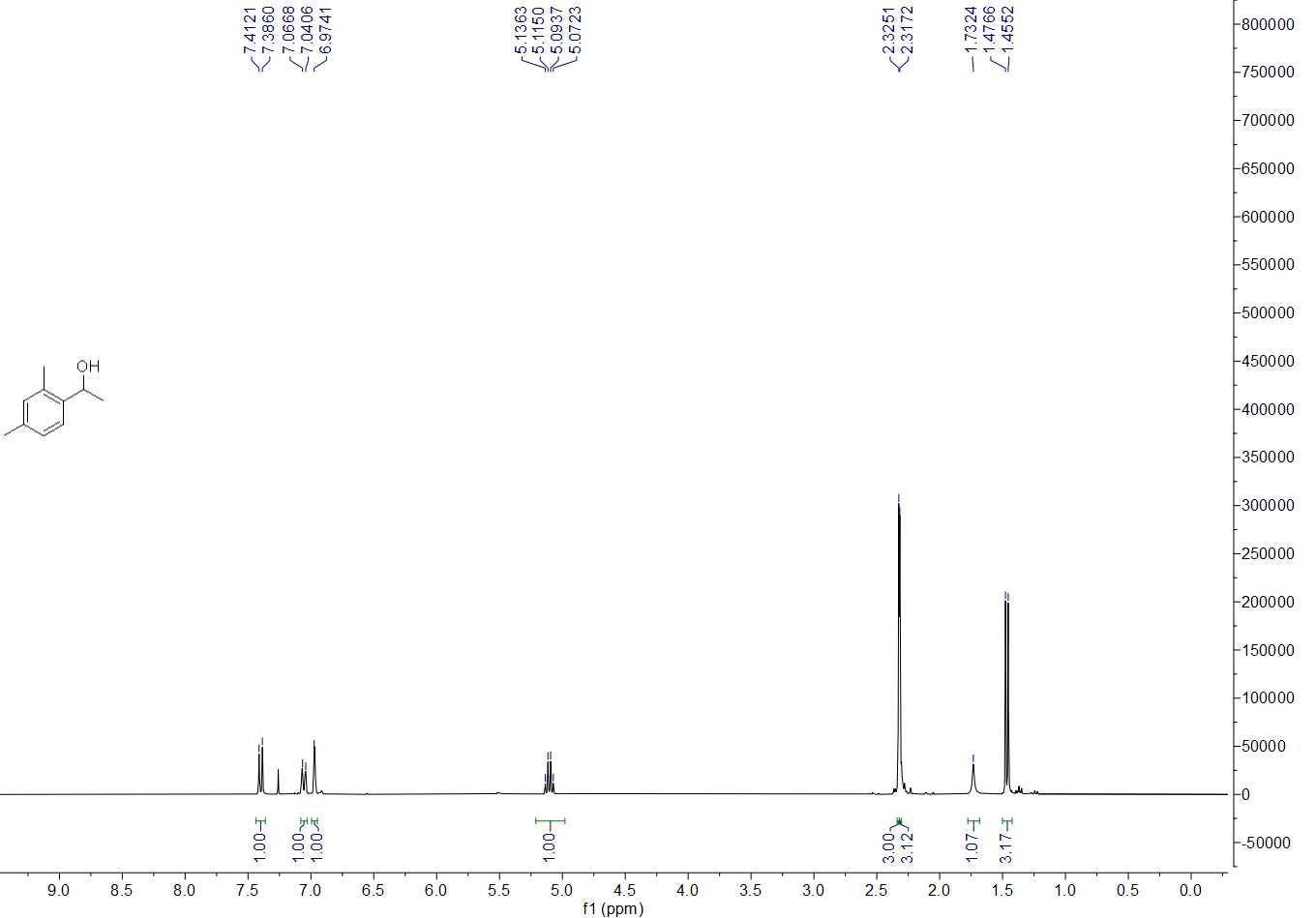


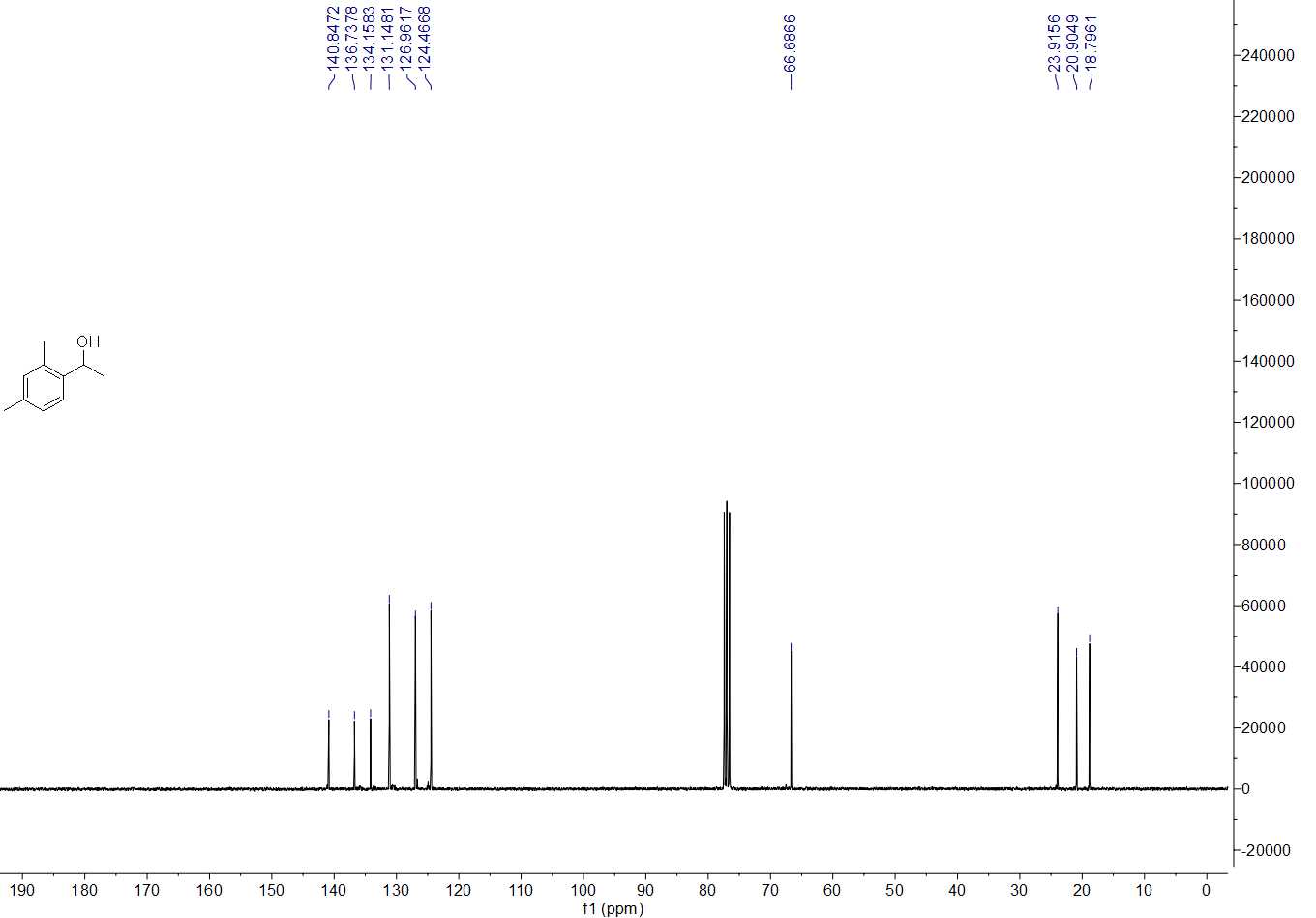


**Supplementary Fig 46.** ^1^H (upper part) and ^13^C NMR (lower part) of **1n**.


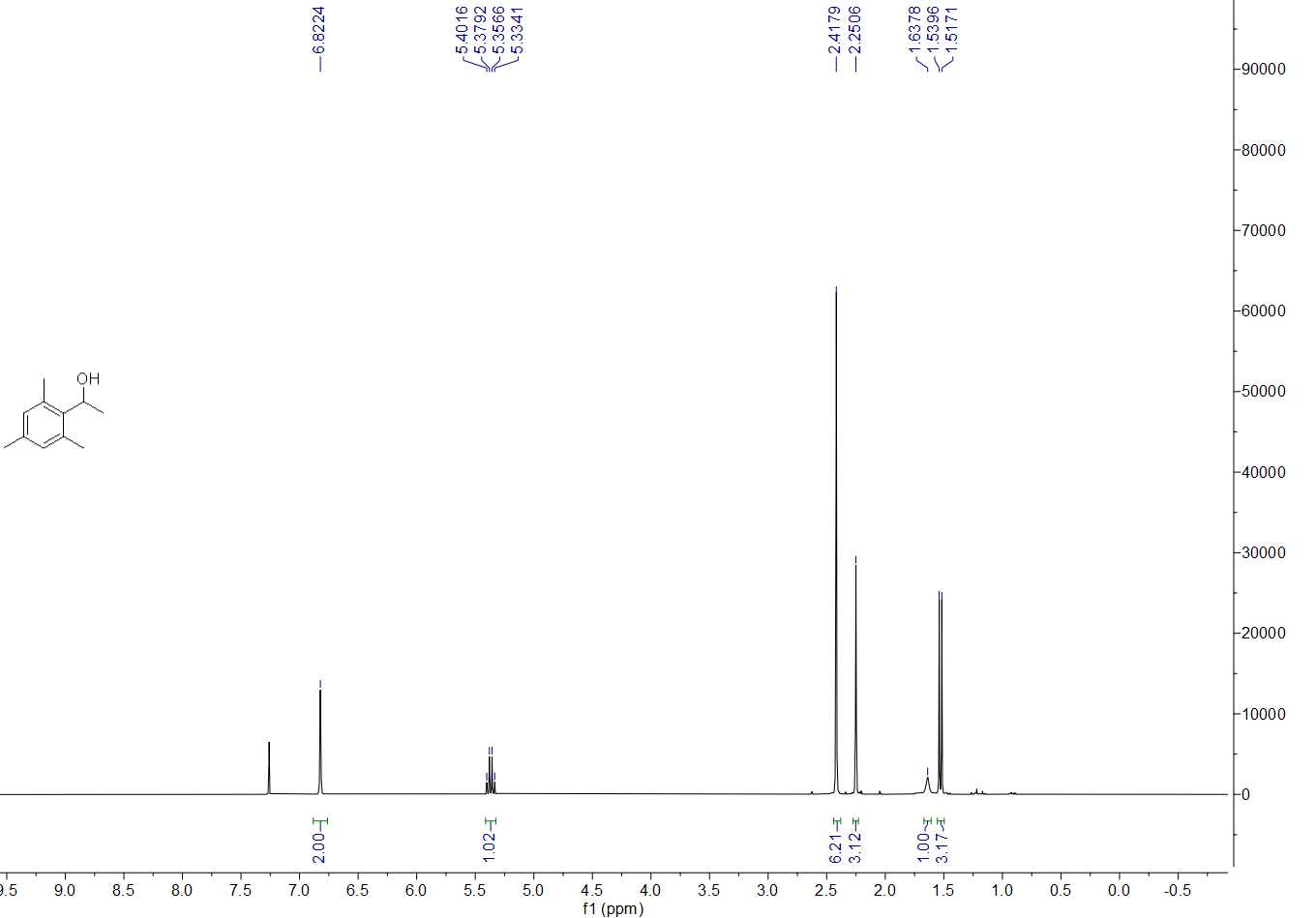


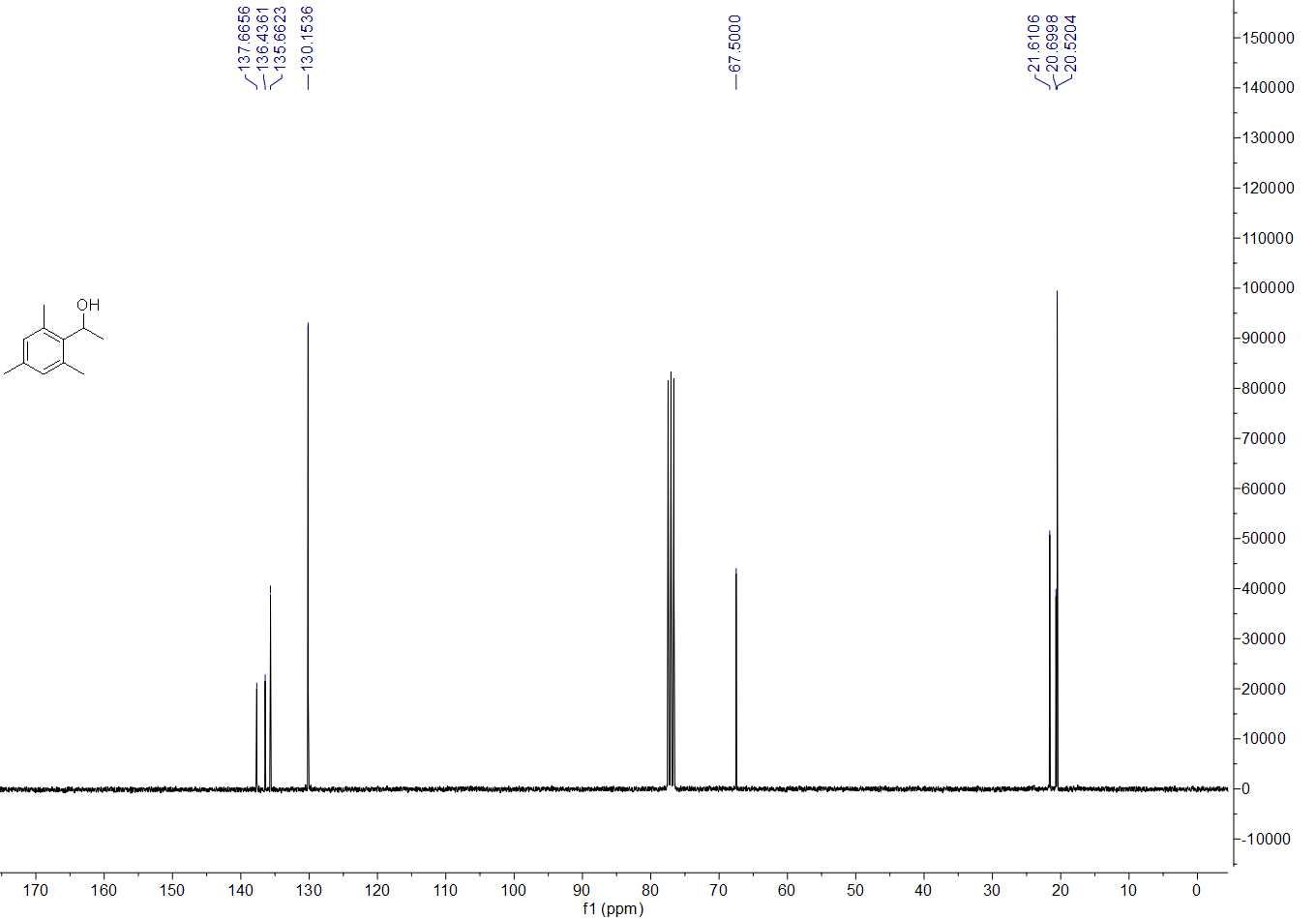


**Supplementary Fig 47.** ^1^H (upper part) and ^13^C NMR (lower part) of **1o**.


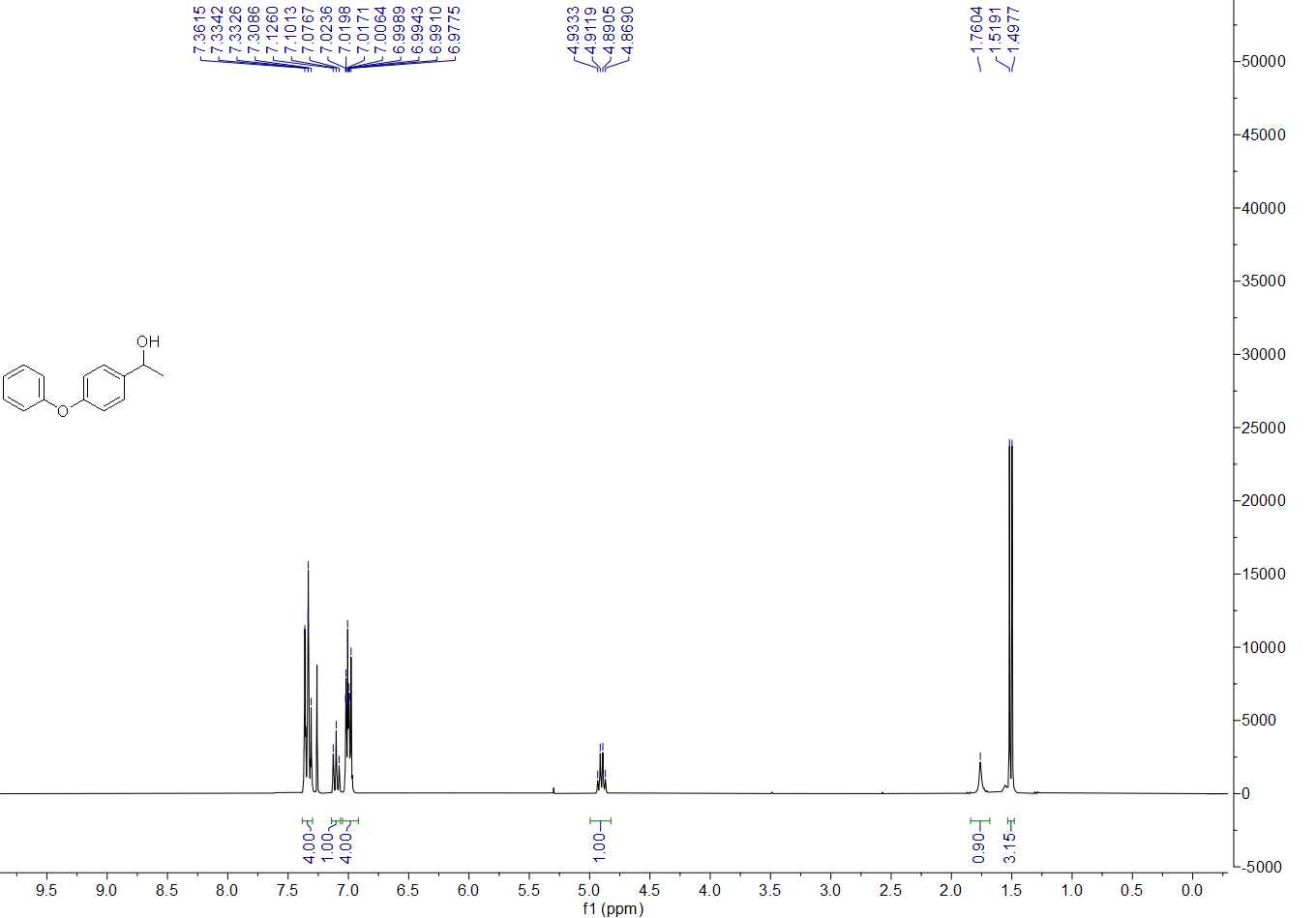


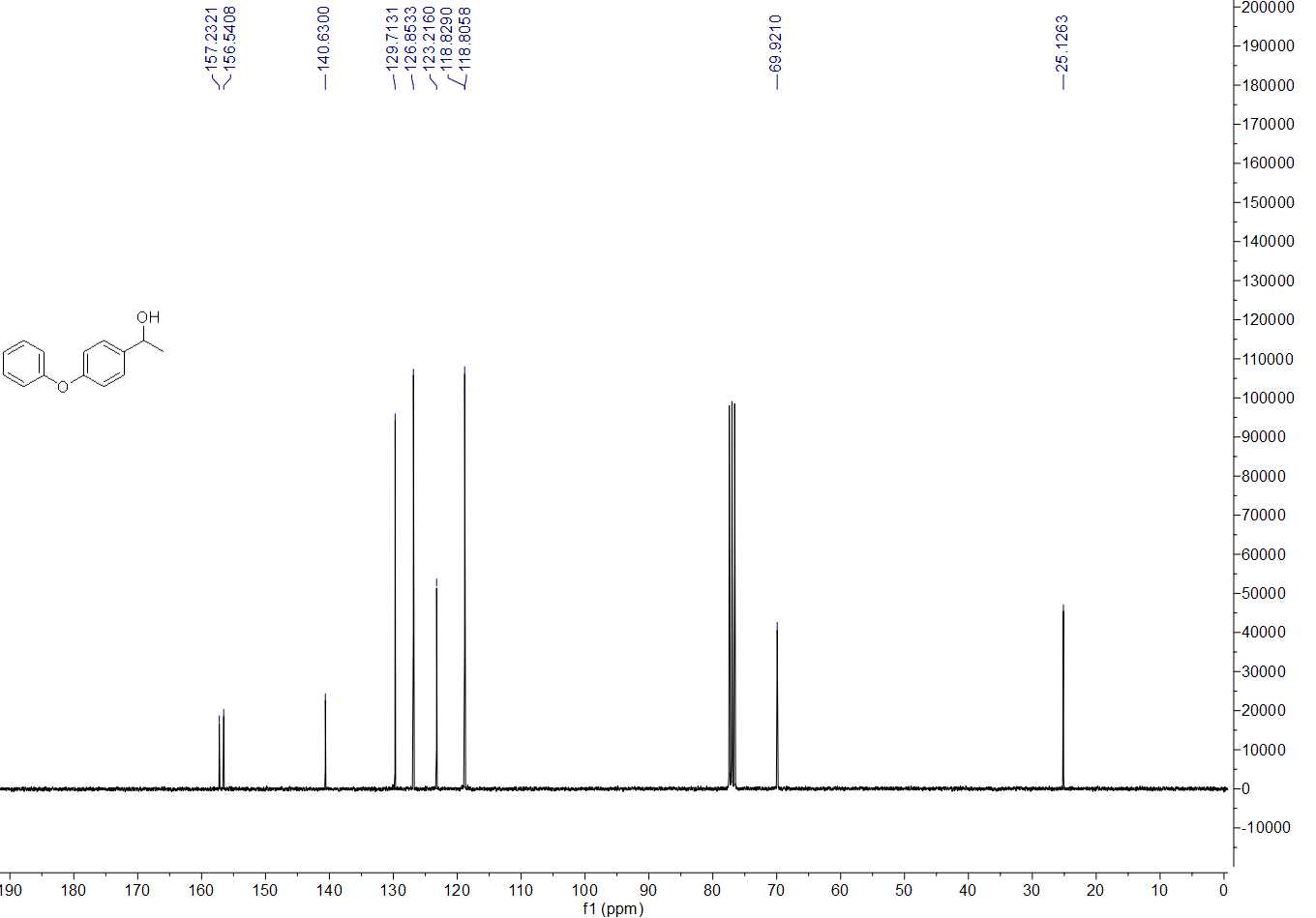


**Supplementary Fig 48.** ^1^H (upper part) and ^13^C NMR (lower part) of **1q**.


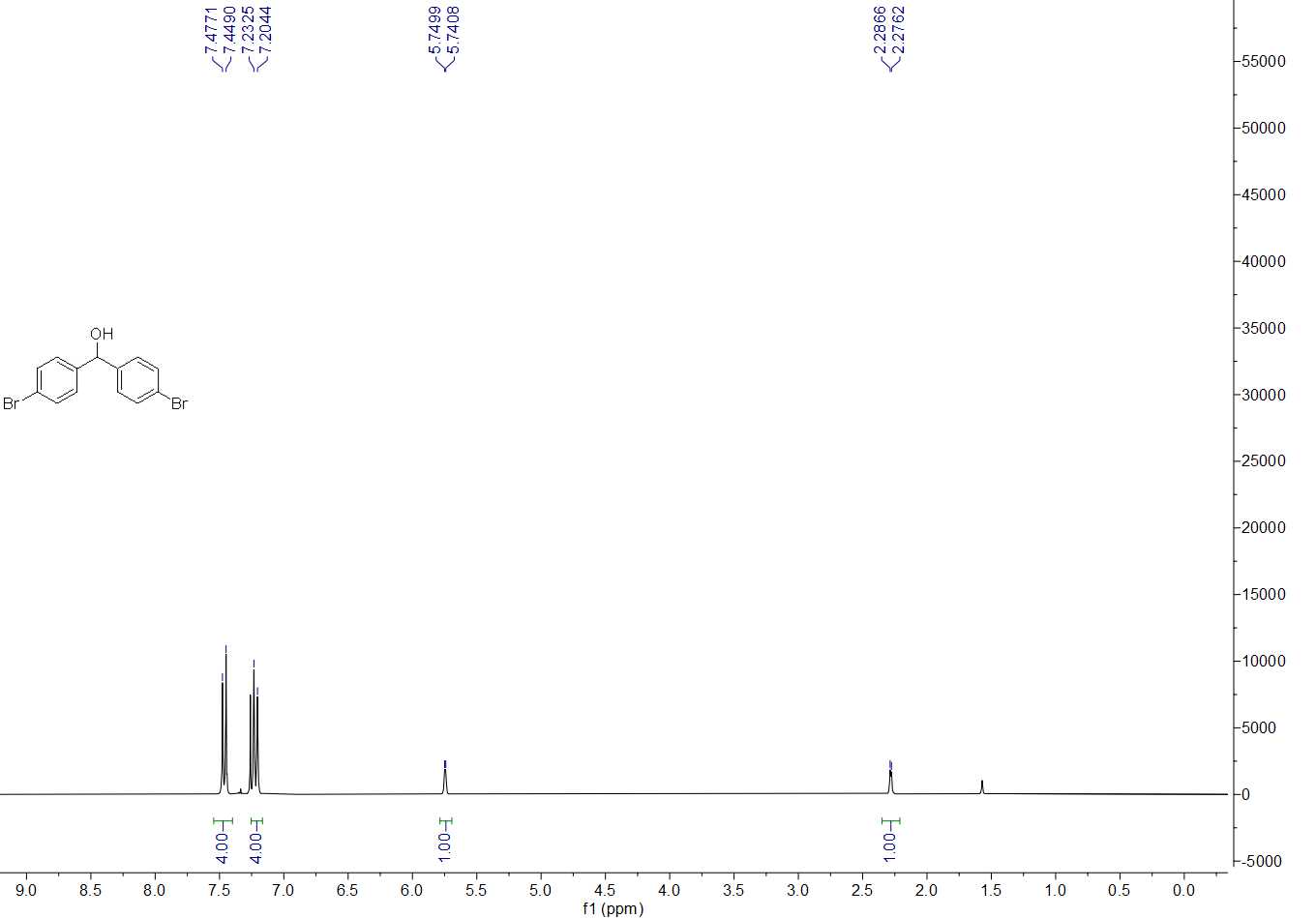


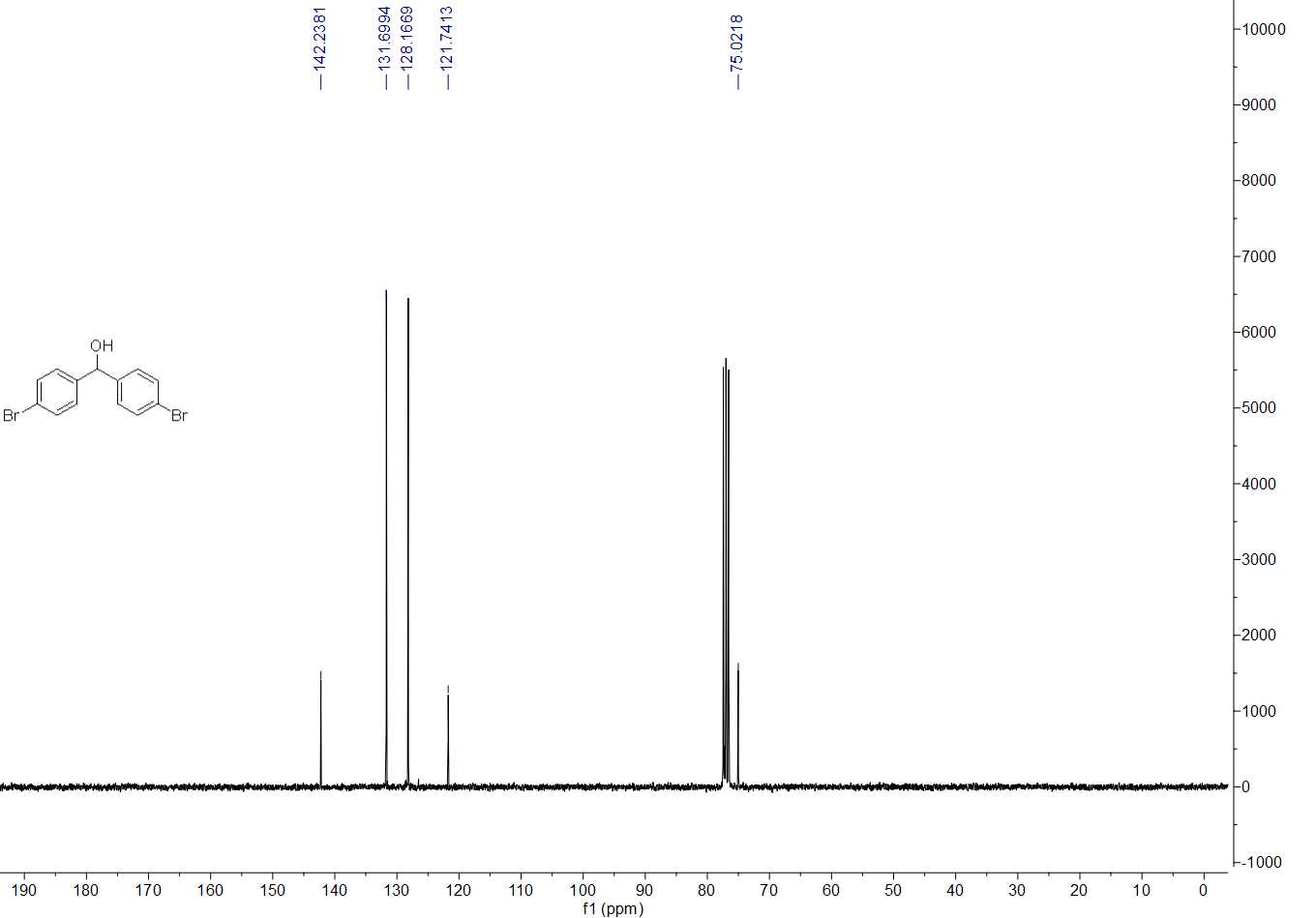


**Supplementary Fig 49.** ^1^H (upper part) and ^13^C NMR (lower part) of **1s**.


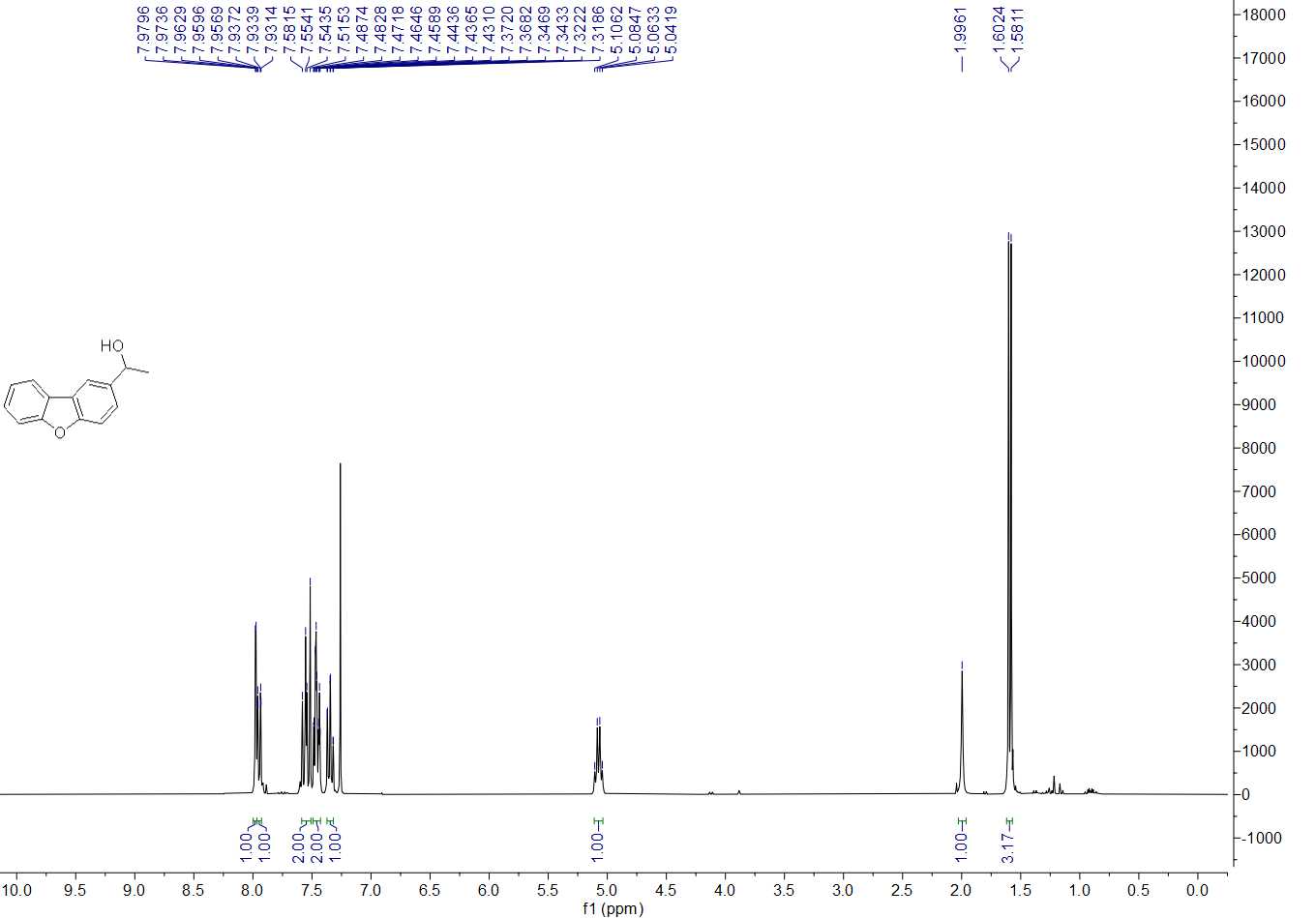


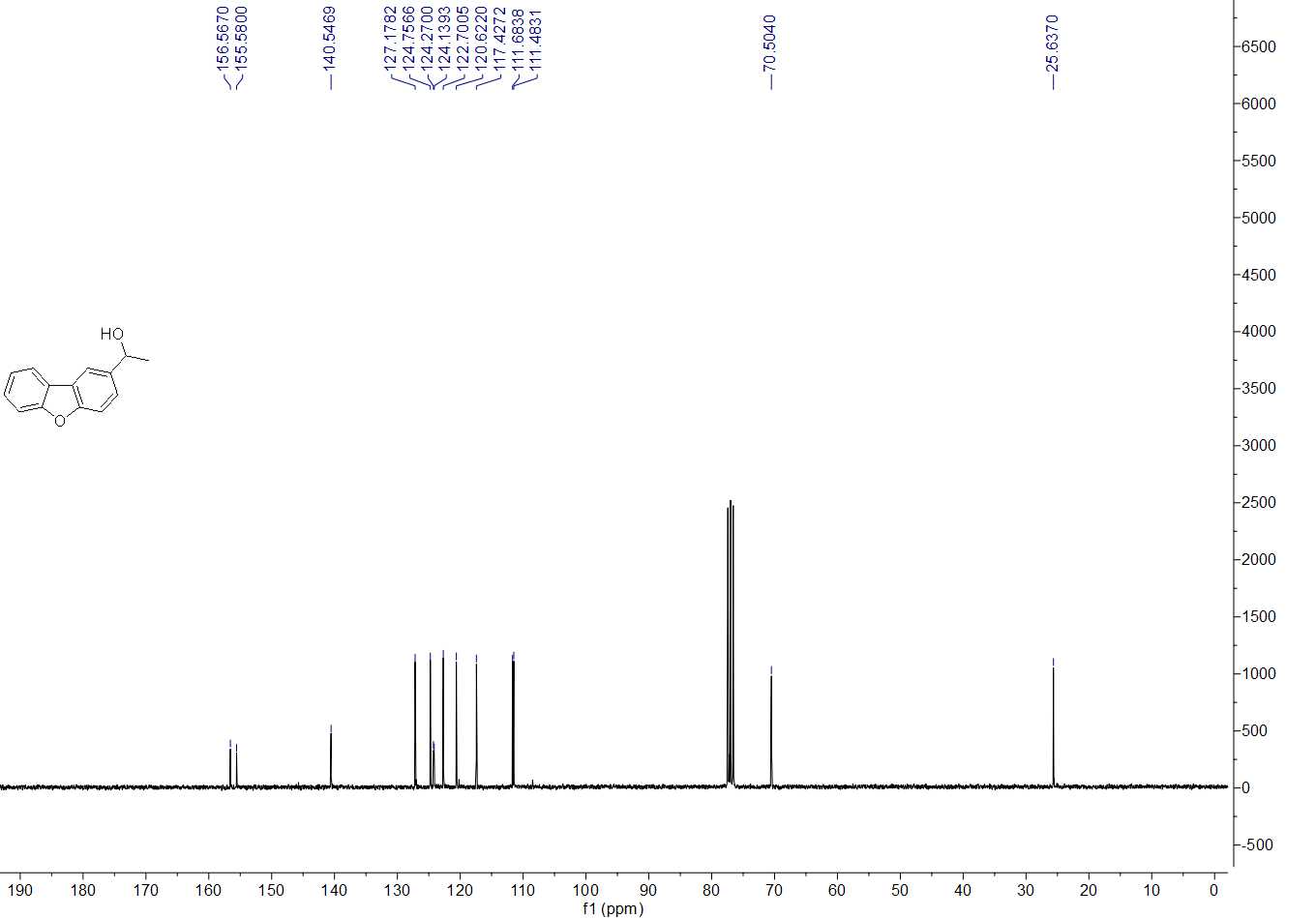


**Supplementary Fig 50.** ^1^H (upper part) and ^13^C NMR (lower part) of **1t**.


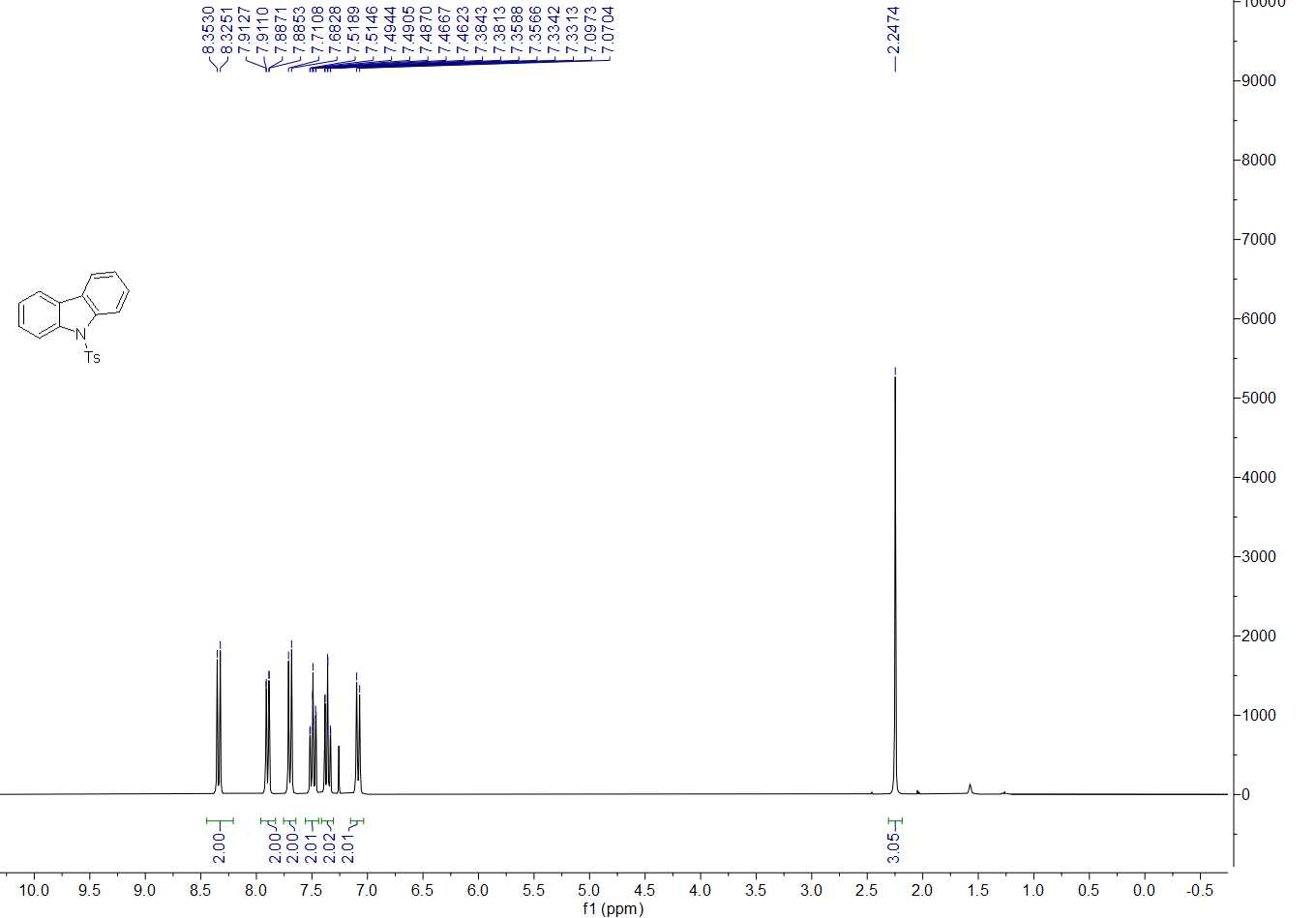


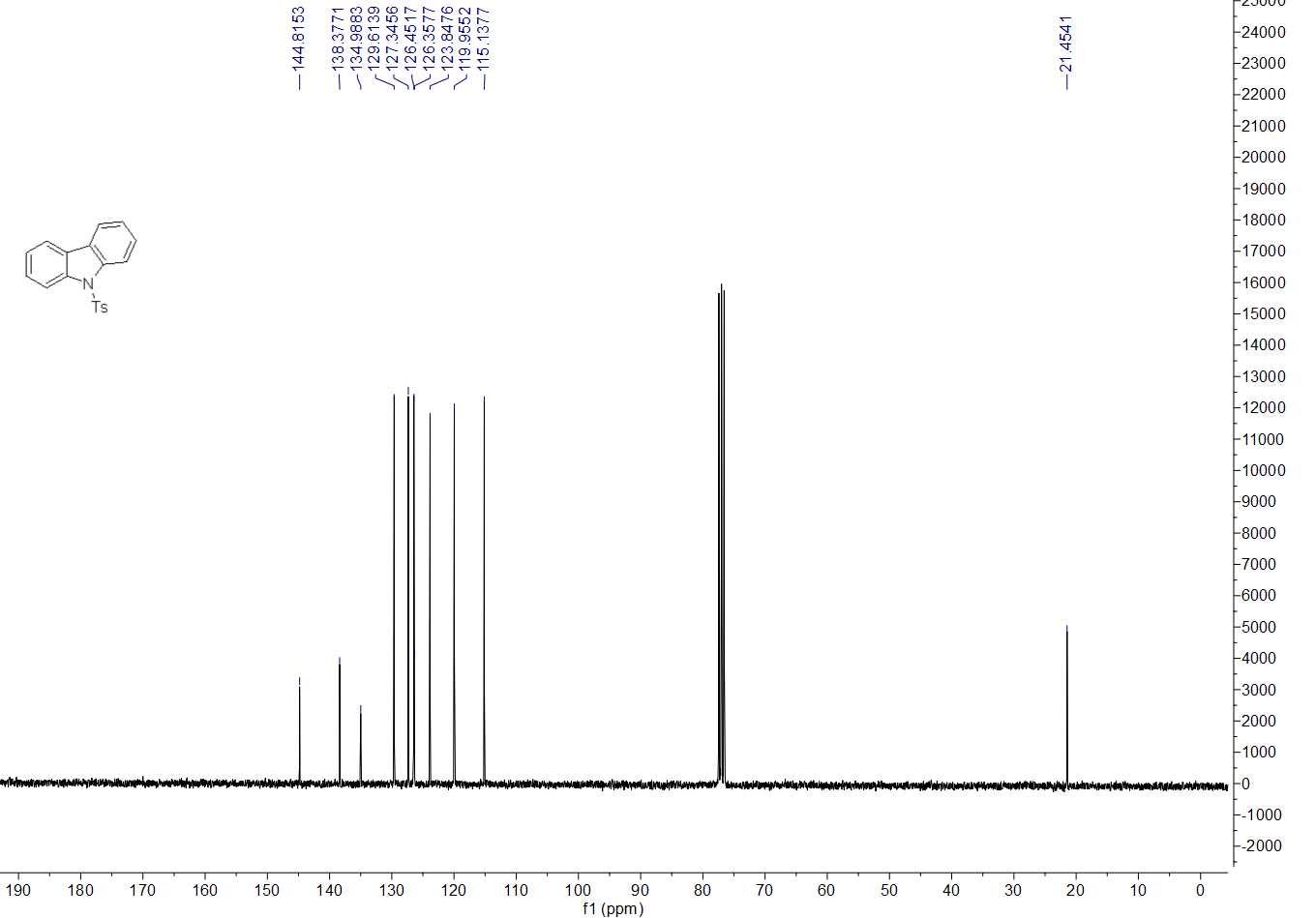


**Supplementary Fig 51.** ^1^H (upper part) and ^13^C NMR (lower part) of 9-tosyl-9*H*-carbazole.


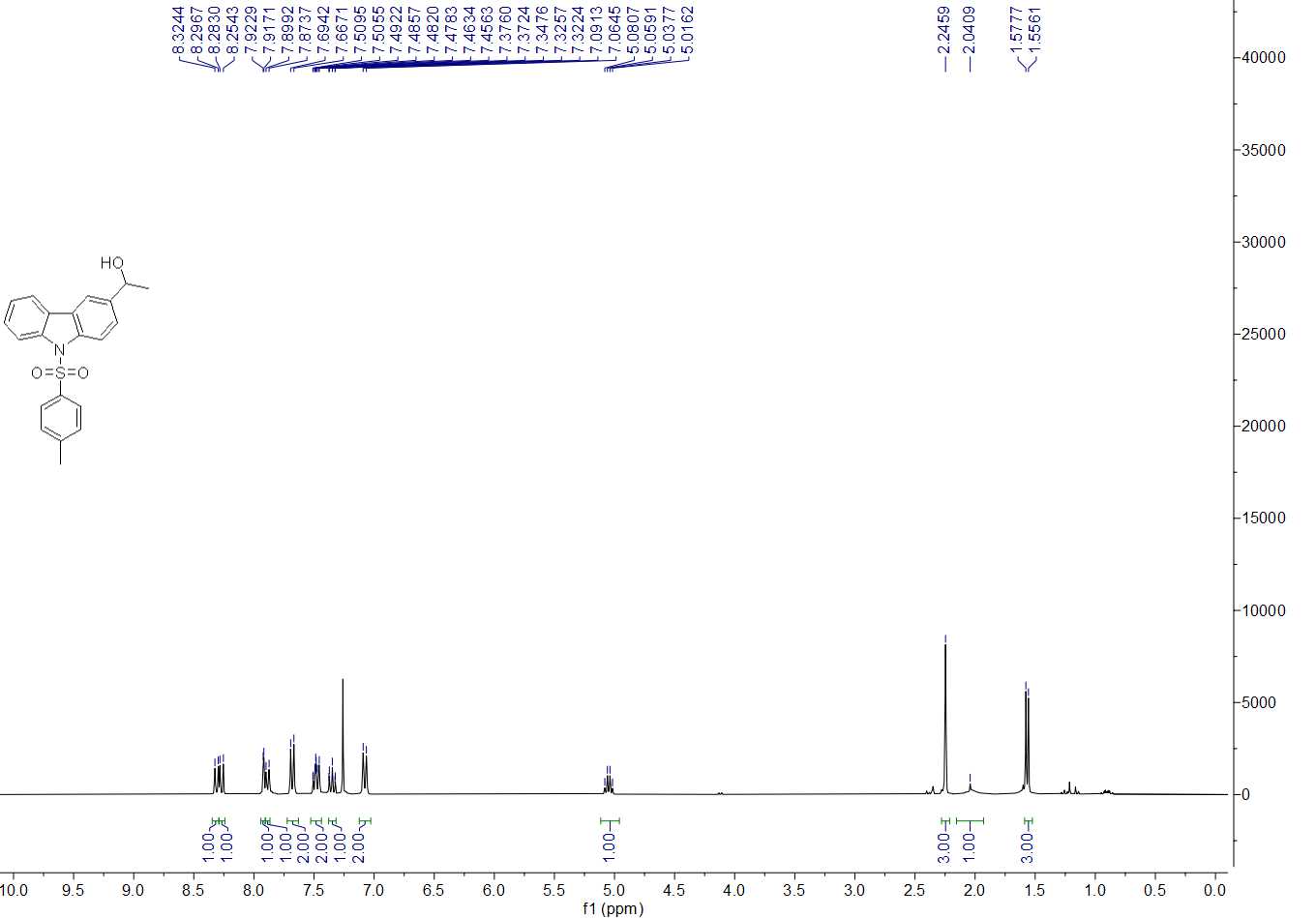


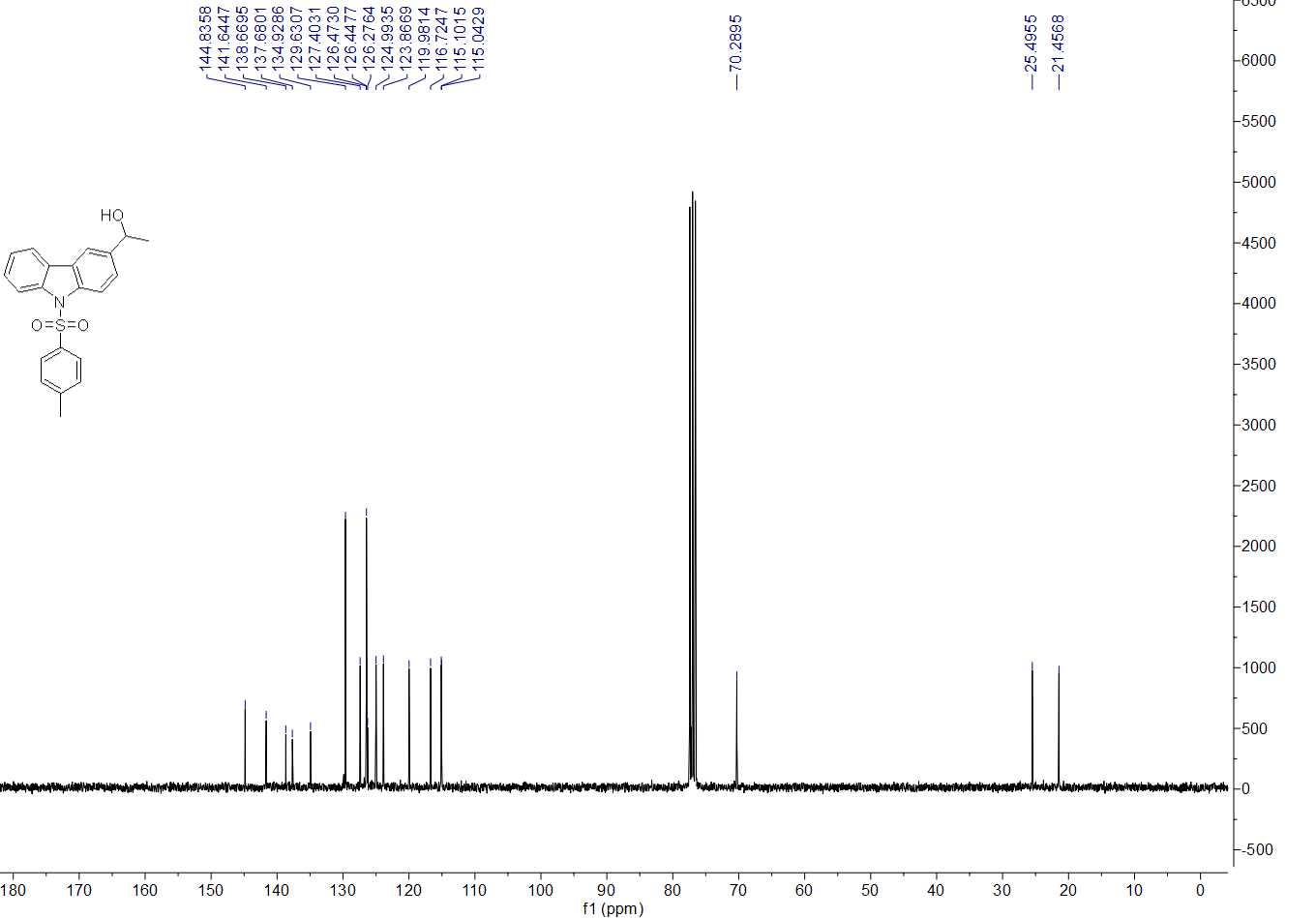


**Supplementary Fig 52.** ^1^H (upper part) and ^13^C NMR (lower part) of **1u**.


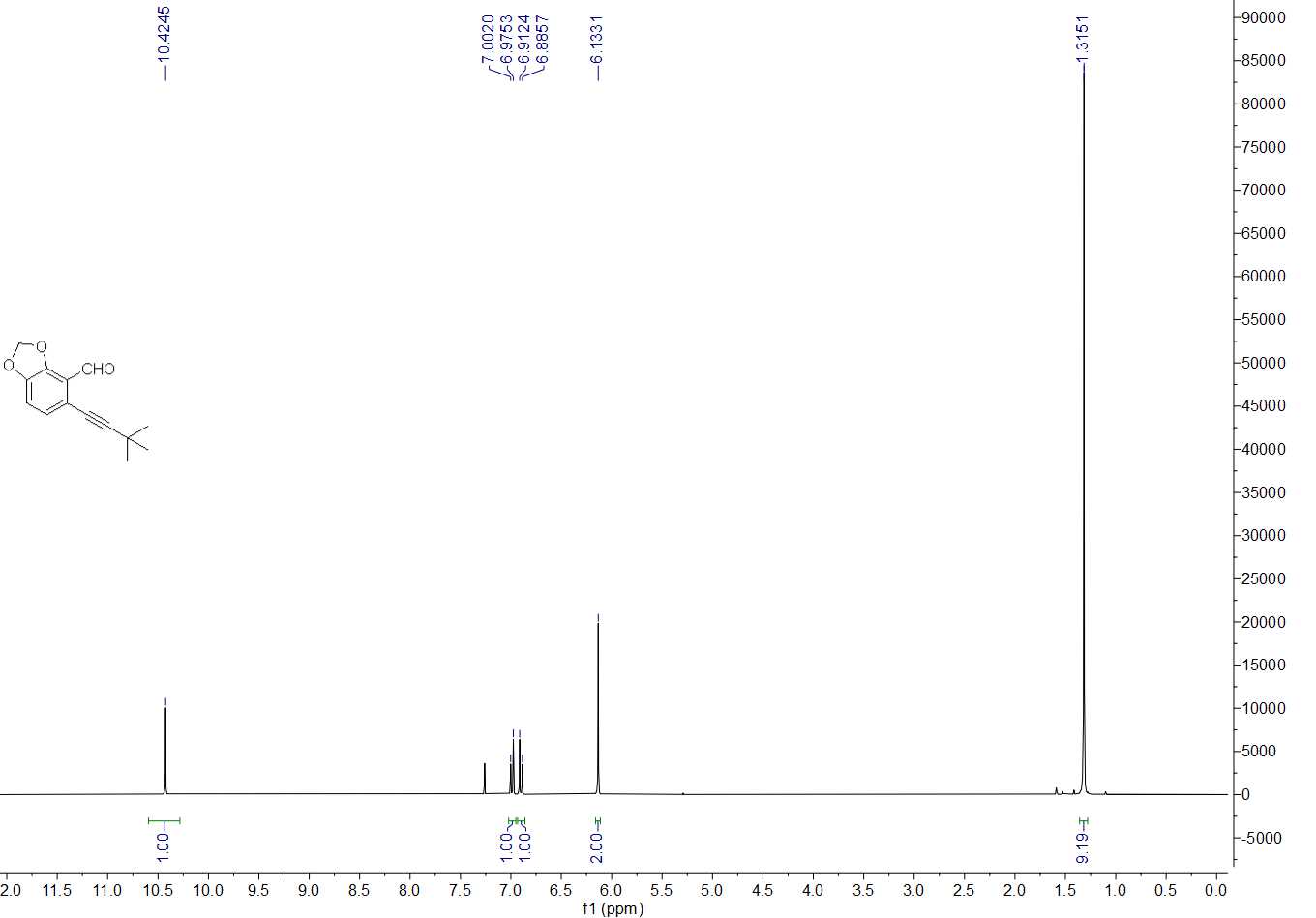


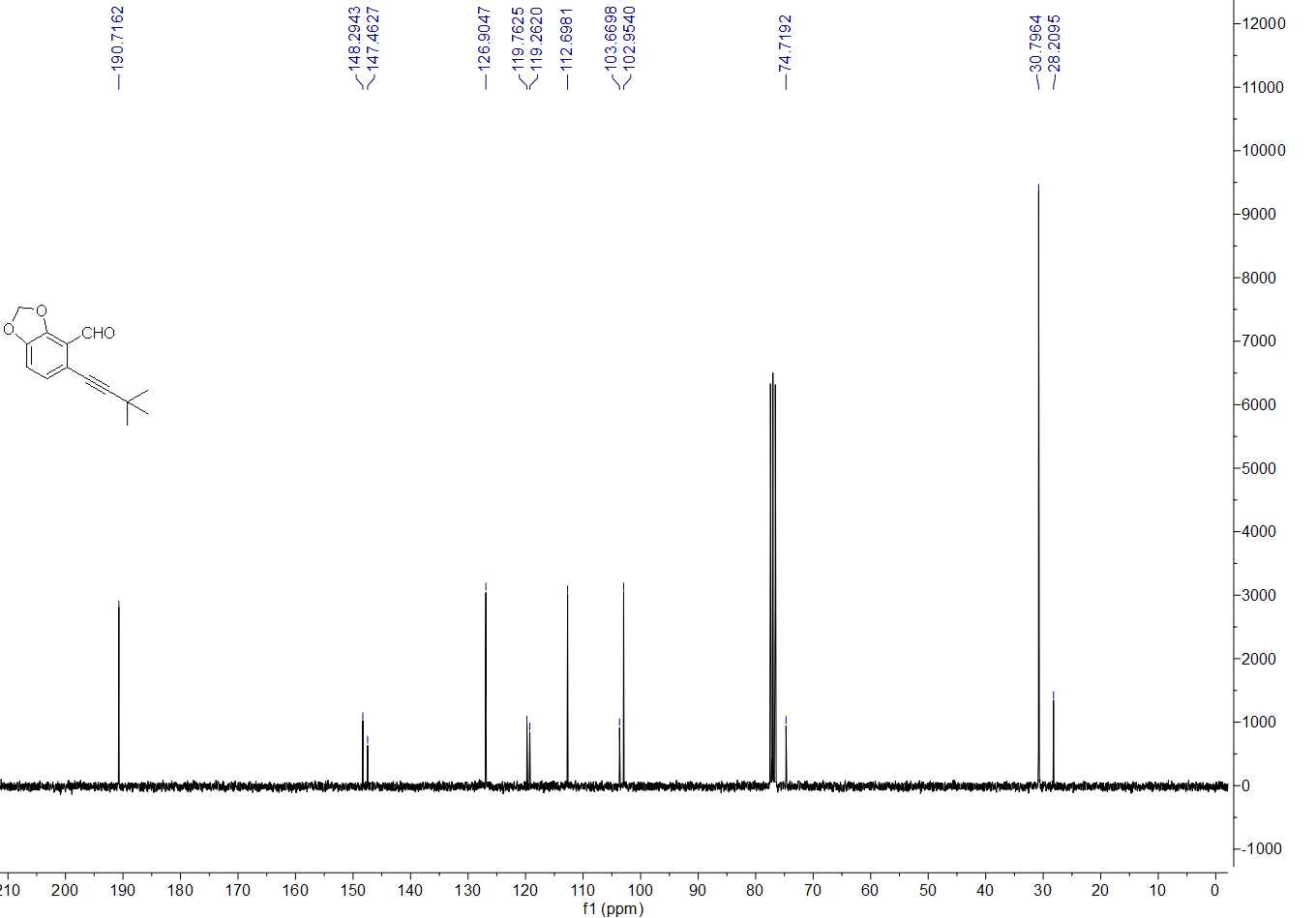


**Supplementary Fig 53.** ^1^H (upper part) and ^13^C NMR (lower part) of 5-(3,3-dimethylbut-1-yn-1-yl)benzo[*d*][1,3]dioxole-4-carbaldehyde.


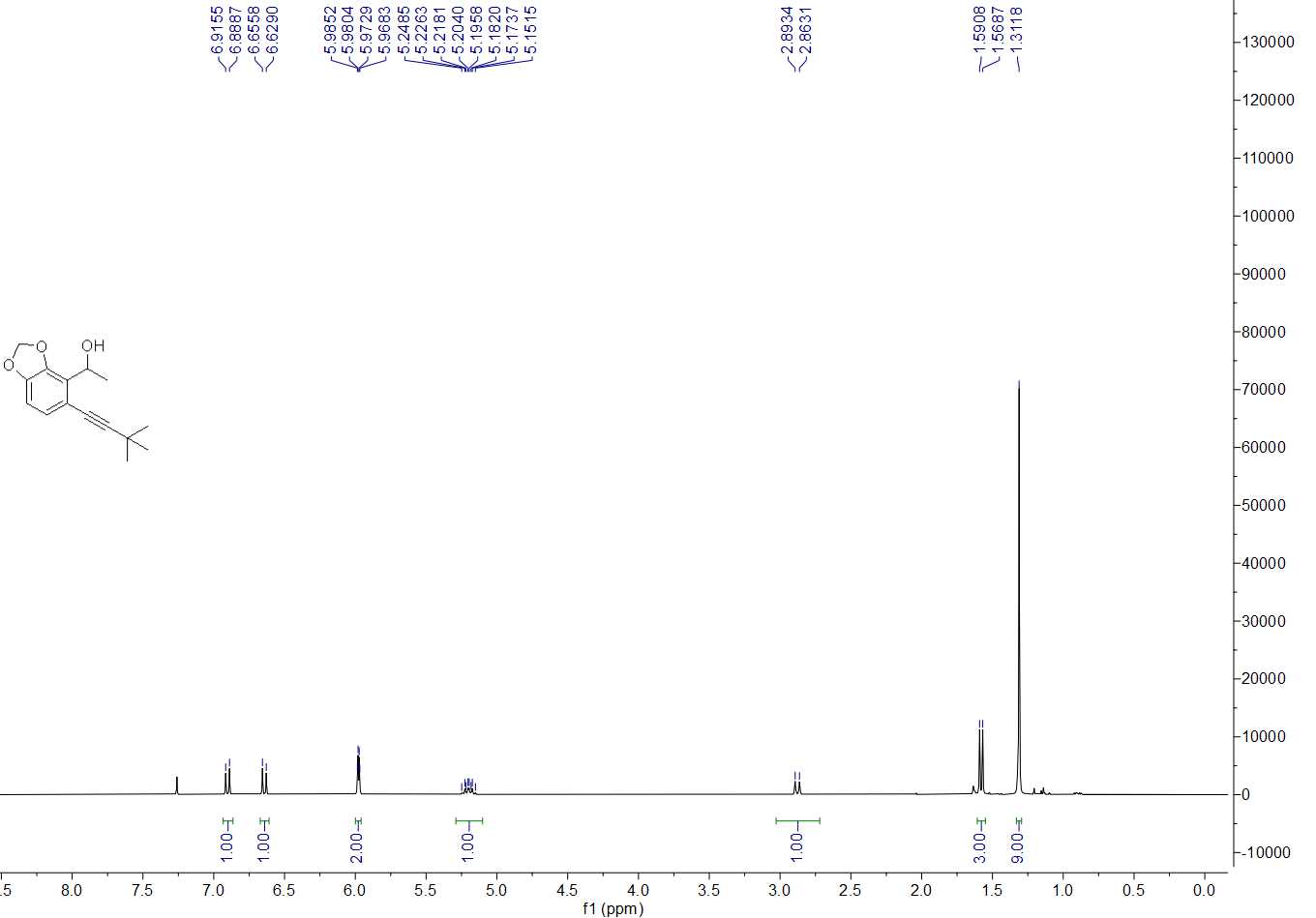


**Supplementary Fig 54.** ^1^H (upper part) and ^13^C NMR (lower part) of **1v**.

**Supplementary Fig 55.** ^1^H (upper part) and ^13^C NMR (lower part) of **1w**.

**Supplementary Fig 56.** ^1^H (upper part) and ^13^C NMR (lower part) of 1-tosylindoline**.**

**Supplementary Fig 57.** ^1^H (upper part) and ^13^C NMR (lower part) of **1x.**

**Supplementary Fig 58.** ^1^H (upper part) and ^13^C NMR (lower part) of **1y.**

**Supplementary Fig 59.** ^1^H (upper part) and ^13^C NMR (lower part) of **1z.**

**Supplementary Fig 60.** ^1^H (upper part) and ^13^C NMR (lower part) of methyl 5-(2,5-dimethyl phenoxy)-2,2-dimethyl pentanoate**.**

**Supplementary Fig 61.** ^1^H (upper part) and ^13^C NMR (lower part) of **1aa.**

**Supplementary Fig 62.** ^1^H (upper part) and ^13^C NMR (lower part) of methyl (*S*)-2-(6-methoxynaphthalen-2-yl)propanoate.

**Supplementary Fig 63.** ^1^H (upper part) and ^13^C NMR (lower part) of **1ab**.

**Supplementary Fig 64.** ^1^H (upper part) and ^13^C NMR (lower part) of methyl 2-(4-(4-chlorobenzoyl)phenoxy)-2-methylpropanoate**.**

**Supplementary Fig 65.** ^1^H (upper part) and ^13^C NMR (lower part) of **1ac.**

**Supplementary Fig 66.** ^1^H (upper part) and ^13^C NMR (lower part) of 1-ethyl 3-(3,4-dimethoxyphenyl)-3-hydroxy-2-(2-methoxyphenoxy)propanoate**.**

**Supplementary Fig 67.** ^1^H (upper part) and ^13^C NMR (lower part) of **1ad.**

**Supplementary Fig 68.** ^1^H (upper part) and ^13^C NMR (lower part) of (8*R*,9*S*,13*S*,14*S*)-13-Methyl-17-oxo-7,8,9,11,12,13,14,15,16,17-decahydro-6*H*-cyclopenta[*a*]phenanthren-3-yl trifluoromethanesulfonate.

**Supplementary Fig 69.** ^19^F NMR of (8*R*,9*S*,13*S*,14*S*)-13-Methyl-17-oxo-7,8,9,11,12,13,14,15,16,17-decahydro-6*H*-cyclopenta[*a*]phenanthren-3-yl trifluoromethanesulfonate.

**Supplementary Fig 70.** ^1^H NMR of (8*R*,9*S*,13*S*,14*S*)-3-acetyl-13-methyl-6,7,8,9,11,12,13,14,15,16-decahydro-17*H*-cyclopenta[*a*]phenanthren-17-one.

**Supplementary Fig 71.** ^13^C NMR (lower part) of (8*R*,9*S*,13*S*,14*S*)-3-acetyl-13-methyl-6,7,8,9,11,12,13,14,15,16-decahydro-17*H*-cyclopenta[*a*]phenanthren-17-one.

**Supplementary Fig 72.** ^1^H NMR (lower part) of **1ae**.

**Supplementary Fig 73.** ^13^C NMR (lower part) of **1ae**.

**Supplementary Fig 74.** ^1^H NMR (lower part) of **1af**.

**Supplementary Fig 75.** ^1^H (upper part) and ^13^C NMR (lower part) of **1ag**.

**Supplementary Fig 76.** ^1^H (upper part) and ^13^C NMR (lower part) of **1ah**.

**Supplementary Fig 77.** ^1^H (upper part) and ^13^C NMR (lower part) of **1ak**.

**Supplementary Fig 78.** ^1^H (upper part) and ^13^C NMR (lower part) of **1al**.

**Supplementary Fig 79.** ^1^H (upper part) and ^13^C NMR (lower part) of **1am**.

**Supplementary Fig 80.** ^1^H (upper part) and ^13^C NMR (lower part) of **1an**.

**Supplementary Fig 81.** ^1^H (upper part) and ^13^C NMR (lower part) of **1ao**.

**Supplementary Fig 82.** ^1^H (upper part) and ^13^C NMR (lower part) of **1ap**.

**Supplementary Fig 83.** ^1^H (upper part) and ^13^C NMR (lower part) of methyl 4-phenylbutanoate

**Supplementary Fig 84.** ^1^H (upper part) and ^13^C NMR (lower part) of **1aq**.

**Supplementary Fig 85.** ^1^H (upper part) and ^13^C NMR (lower part) of **3a**.

**Supplementary Fig 86.** ^1^H (upper part) and ^13^C NMR (lower part) of **3b**.

**Supplementary Fig 87.** ^1^H (upper part) and ^13^C NMR (lower part) of **3c**.

**Supplementary Fig 88.** ^1^H (upper part) and ^13^C NMR (lower part) of **3d**.

**Supplementary Fig 89.** ^1^H (upper part) and ^13^C NMR (lower part) of **3g**.

**Supplementary Fig 90.** ^1^H (upper part) and ^13^C NMR (lower part) of **3h.**

**Supplementary Fig 91.** ^1^H (upper part) and ^13^C NMR (lower part) of **3i**.

**Supplementary Fig 92.** ^1^H (upper part) and ^13^C NMR (lower part) of **3j**.

**Supplementary Fig 93.** ^1^H (upper part) and ^13^C NMR (lower part) of **3k**.

**Supplementary Fig 94.** ^1^H (upper part) and ^13^C NMR (lower part) of **3l**.

**Supplementary Fig 95.** ^1^H (upper part) and ^13^C NMR (lower part) of **3m**.

**Supplementary Fig 96.** ^1^H (upper part) and ^13^C NMR (lower part) of **3n**.

**Supplementary Fig 97.** ^1^H (upper part) and ^13^C NMR (lower part) of **3o**.

**Supplementary Fig 98.** ^1^H (upper part) and ^13^C NMR (lower part) of **3p**.

**Supplementary Fig 99.** ^1^H (upper part) and ^13^C NMR (lower part) of **3q**.

**Supplementary Fig 100.** ^1^H (upper part) and ^13^C NMR (lower part) of **3r**.

**Supplementary Fig 101.** ^1^H (upper part) and ^13^C NMR (lower part) of **3s**.

**Supplementary Fig 102.** ^1^H (upper part) and ^13^C NMR (lower part) of **3s´**.

**Supplementary Fig 103.** ^1^H (upper part) and ^13^C NMR (lower part) of **3t**.

**Supplementary Fig 104.** ^1^H (upper part) and ^13^C NMR (lower part) of **3u**.

**Supplementary Fig 105.** ^1^H (upper part) and ^13^C NMR (lower part) of **3v.**

**Supplementary Fig 106.** ^1^H (upper part) and ^13^C NMR (lower part) of **3w**.

**Supplementary Fig 107.** ^1^H (upper part) and ^13^C NMR (lower part) of **3x**.

**Supplementary Fig 108.** ^1^H (upper part) and ^13^C NMR (lower part) of **3y**.

**Supplementary Fig 109.** ^1^H (upper part) and ^13^C NMR (lower part) of **3z**.

**Supplementary Fig 110.** ^1^H (upper part) and ^13^C NMR (lower part) of **3z´**.

**Supplementary Fig 111.** ^19^F NMR of **3z´**.

**Supplementary Fig 112.** ^1^H (upper part) and ^13^C NMR (lower part) of **3aa**.

**Supplementary Fig 113.** ^1^H (upper part) and ^13^C NMR (lower part) of **3ab**.

**Supplementary Fig 114.** ^1^H (upper part) and ^13^C NMR (lower part) of **3ac**.

**Supplementary Fig 115.** ^1^H (upper part) and ^13^C NMR (lower part) of **3ac´**.

**Supplementary Fig 116.** ^1^H (upper part) and ^13^C NMR (lower part) of **3ad**.

**Supplementary Fig 117.** ^1^H (upper part) and ^13^C NMR (lower part) of **3ae**.

**Supplementary Fig 118.** ^1^H (upper part) and ^13^C NMR (lower part) of **3ag**.

**Supplementary Fig 119.** ^1^H (upper part) and ^13^C NMR (lower part) of **4c**.

**Supplementary Fig 120.** ^1^H (upper part) and ^13^C NMR (lower part) of **4d**.

**Supplementary Fig 121.** ^1^H (upper part) and ^13^C NMR (lower part) of **4e**.

**Supplementary Fig 122.** ^1^H (upper part) and ^13^C NMR (lower part) of **4f**.

**Supplementary Fig 123.** ^1^H (upper part) and ^13^C NMR (lower part) of **4g**.

**Supplementary Fig 124.** ^1^H (upper part) and ^13^C NMR (lower part) of **4h**.

**Supplementary Fig 125.** ^1^H (upper part) and ^13^C NMR (lower part) of **4i**.

**Supplementary Fig 126.** ^1^H (upper part) and ^13^C NMR (lower part) of **4j**.

**Supplementary Fig 127.** ^1^H (upper part) and ^13^C NMR (lower part) of **4k**.

**Supplementary Fig 128.** ^1^H (upper part) and ^13^C NMR (lower part) of **4l**.

**Supplementary Fig 129.** ^1^H (upper part) and ^13^C NMR (lower part) of **4m**.

**Supplementary Fig 130.** ^1^H (upper part) and ^13^C NMR (lower part) of **6a**.

**Supplementary Fig 131.** ^1^H (upper part) and ^13^C NMR (lower part) of **6d**.

**Supplementary Fig 132.** ^1^H (upper part) and ^13^C NMR (lower part) of **6ai**.

**Supplementary Fig 133.** ^1^H (upper part) and ^13^C NMR (lower part) of **6aj**.

**Supplementary Fig 134.** ^19^F NMR of **6aj**.

**Supplementary Fig 135.** ^1^H (upper part) and ^13^C NMR (lower part) of **6ak**.

**Supplementary Fig 136.** ^1^H (upper part) and ^13^C NMR (lower part) of **6n**.

**Supplementary Fig 137.** ^1^H (upper part) and ^13^C NMR (lower part) of **6al**.

**Supplementary Fig 138.** ^1^H (upper part) and ^13^C NMR (lower part) of **6o**.

**Supplementary Fig 139.** ^1^H (upper part) and ^13^C NMR (lower part) of **6p**.

**Supplementary Fig 140.** ^1^H (upper part) and ^13^C NMR (lower part) of **6q**.

**Supplementary Fig 141.** ^1^H (upper part) and ^13^C NMR (lower part) of **6r**.

**Supplementary Fig 142.** ^1^H (upper part) and ^13^C NMR (lower part) of **6s**.

**Supplementary Fig 143.** ^1^H (upper part) and ^13^C NMR (lower part) of **6t**.

**Supplementary Fig 144.** ^1^H (upper part) and ^13^C NMR (lower part) of **6w**.

**Supplementary Fig 145.** ^1^H (upper part) and ^13^C NMR (lower part) of **6x**.

**Supplementary Fig 146.** ^1^H (upper part) and ^13^C NMR (lower part) of **6am**.

**Supplementary Fig 147.** ^1^H (upper part) and ^13^C NMR (lower part) of **6an**.

**Supplementary Fig 148.** ^1^H (upper part) and ^13^C NMR (lower part) of **6ah**.

**Supplementary Fig 149.** ^1^H (upper part) and ^13^C NMR (lower part) of **6y**.

**Supplementary Fig 150.** ^1^H (upper part) and ^13^C NMR (lower part) of **6aa**.

**Supplementary Fig 151.** ^1^H (upper part) and ^13^C NMR (lower part) of **6ab**.

**Supplementary Fig 152.** ^1^H (upper part) and ^13^C NMR (lower part) of **6ac**.

**Supplementary Fig 153.** ^1^H (upper part) and ^13^C NMR (lower part) of **6ae**.

**Supplementary Fig 154.** ^1^H (upper part) and ^13^C NMR (lower part) of **7**.

**Supplementary Fig 155.** ^1^H (upper part) and ^13^C NMR (lower part) of **8**.

**Supplementary Fig 156.** ^1^H (upper part) and ^13^C NMR (lower part) of **9**.

**Supplementary Fig 157.** ^1^H (upper part) and ^13^C NMR (lower part) of **10**.

**Supplementary Fig 158.** ^1^H (upper part) and ^13^C NMR (lower part) of **11**.

**Supplementary Fig 159.** ^1^H (upper part) and ^13^C NMR (lower part) of **12**.

**Supplementary Fig 160.** ^1^H (upper part) and ^13^C NMR (lower part) of **13**.

**Supplementary Fig 161.** ^1^H (upper part) and ^13^C NMR (lower part) of **14**.

**Supplementary Fig 162.** ^1^H (upper part) and ^13^C NMR (lower part) of **15**.

**Supplementary Fig 163.** ^1^H (upper part) and ^13^C NMR (lower part) of 2,6-di-*tert*-butyl-4-methyl-4-(methylamino)cyclohexa-2,5-dien-1-one.

**Supplementary Fig 164.** ^1^H (upper part) and ^13^C NMR (lower part) of **16.**

**Supplementary Fig 165.** ^1^H (upper part) and ^13^C NMR (lower part) of **17.**

**Supplementary Fig 166.** ^19^F NMR of **17**.

**Supplementary Fig 167.** ^1^H NMR of **19**.

**Supplementary Fig 168.** ^13^C NMR of **19**.

**3. Supplementary References**

1 Wang, T. *et al.* A metal‐free direct arene C−H amination. *Adv. Synth. Catal.*, (2021).

2 Masruri, Willis, A. C. & McLeod, M. D. Osmium-catalyzed vicinal oxyamination of alkenes by N-(4-toluenesulfonyloxy)carbamates. *J. Org. Chem.* **77**, 8480-8491, (2012).

3 Shi, Z. *et al.* Development of a practical synthesis of a p38 kinase inhibitor via a safe and robust amination. *Org. Process Res. Dev.* **16**, 1618-1625, (2012).

4 Kitowski, A. *et al.* Oxidative activation of C-S bonds with an electropositive nitrogen promoter enables orthogonal glycosylation of alkyl over phenyl thioglycosides. *Org. Lett.* **19**, 5490-5493, (2017).

5 Makai, S., Falk, E. & Morandi, B. Direct synthesis of unprotected 2-azidoamines from alkenes via an iron-catalyzed difunctionalization reaction. *J. Am. Chem. Soc.* **142**, 21548-21555, (2020).

6 Liu, J. *et al.* Fe-catalyzed amination of (hetero)arenes with a redox-active aminating reagent under mild conditions. *Chem.-Eur. J.* **23**, 563-567, (2017).

7 Tamura, Y. *et al.* O-Arenesulfonyl-N-alkylhydroxylamines as aminating reagents. *Chem Pharm Bull* **30**, 1221-1224, (1982).

8 Farndon, J. J., Ma, X. & Bower, J. F. Transition metal free C-N bond forming dearomatizations and aryl C-H aminations by in situ release of a hydroxylamine-based aminating agent. *J. Am. Chem. Soc.* **139**, 14005-14008, (2017).

9 Paudyal, M. P. *et al.* Dirhodium-catalyzed C-H arene amination using hydroxylamines. *Science* **353**, 1144-1147, (2016).

10 Luo, R. *et al.* An efficient hydration and tandem transfer hydrogenation of alkynes for the synthesis of alcohol in water. *Synthesis* **52**, 3439-3445, (2020).

11 Yamashita, Y. *et al.* Catalytic direct-type addition reactions of alkylarenes with imines and alkenes. *Angew. Chem. Int. Ed.* **57**, 6896-6900, (2018).

12 Xiang, J. *et al.* Hindered dialkyl ether synthesis with electrogenerated carbocations. *Nature* **573**, 398-402, (2019).

13 Mandal, T., Jana, S. & Dash, J. Zinc-mediated efficient and selective reduction of carbonyl compounds. *Eur. J. Org. Chem.* **2017**, 4972-4983, (2017).

14 Duczynski, J. *et al.* The synthesis and catalytic activity of new mixed NHC-phosphite nickel(0) complexes. *Organometallics* **39**, 105-115, (2019).

15 Qu, P., Kuepfert, M., Jockusch, S. & Weck, M. Compartmentalized nanoreactors for one-pot redox-driven transformations. *ACS Catal.* **9**, 2701-2706, (2019).

16 Dong, X., Weickgenannt, A. & Oestreich, M. Broad-spectrum kinetic resolution of alcohols enabled by Cu-H-catalysed dehydrogenative coupling with hydrosilanes. *Nat. Commun.* **8**, 15547, (2017).

17 Hashimoto, T., Ishimaru, T., Shiota, K. & Yamaguchi, Y. Bottleable NiCl_2_(dppe) as a catalyst for the Markovnikov-selective hydroboration of styrenes with bis(pinacolato)diboron. *Chem. Commun.* **56**, 11701-11704, (2020).

18 Polidano, K. *et al.* Exploring tandem ruthenium-catalyzed hydrogen transfer and S_N_Ar Chemistry. *Org. Lett.* **19**, 6716-6719, (2017).

19 Padmanaban, M., Biju, A. T. & Glorius, F. N-heterocyclic carbene-catalyzed cross-coupling of aromatic aldehydes with activated alkyl halides. *Org. Lett.* **13**, 98-101, (2011).

20 Patpi, S. R. *et al.* Design, synthesis, and structure-activity correlations of novel dibenzo[b,d]furan, dibenzo[b,d]thiophene, and N-methylcarbazole clubbed 1,2,3-triazoles as potent inhibitors of Mycobacterium tuberculosis. *J. Med. Chem.* **55**, 3911-3922, (2012).

21 Hashmi, A. S. *et al.* Simple gold-catalyzed synthesis of benzofulvenes--gem-diaurated species as "instant dual-activation" precatalysts. *Angew. Chem. Int. Ed.* **51**, 4456-4460, (2012).

22 Berthier, D., Herrmann, A., Paret, N. & Ouali, L. Microcapsules and uses thereof. WO 2013/079435A l (2013).

23 Zhou, S.-L., Guo, L.-N. & Duan, X.-H. Copper-catalyzed regioselective cross-dehydrogenative coupling of coumarins with benzylic Csp^3^-H bonds. *Eur. J. Org. Chem.* **2014**, 8094-8100, (2014).

24 Liu, J. *et al.* From alkylarenes to anilines via site-directed carbon-carbon amination. *Nat. Chem.* **11**, 71-77, (2019).

25 Bomon, J. *et al.* Bronsted acid catalyzed tandem defunctionalization of biorenewable ferulic acid and derivates into bio-catechol. *Angew. Chem. Int. Ed.* **59**, 3063-3068, (2020).

26 Hofmann, L. E. *et al.* Sequential cleavage of lignin systems by nitrogen monoxide and hydrazine. *Adv. Synth. Catal.* **362**, 1485-1489, (2020).

27 Tang, M. *et al.* Carbosulfenylation of alkenes with organozinc reagents and dimethyl(methylthio)sulfonium trifluoromethanesulfonate. *Org. Lett.* **22**, 9729-9734, (2020).

28 Xia, Y., Wang, J. & Dong, G. Suzuki-Miyaura coupling of simple ketones via activation of unstrained carbon-carbon bonds. *J. Am. Chem. Soc.* **140**, 5347-5351, (2018).

29 Liu, L. *et al.* Intra- and intermolecular Fe-catalyzed dicarbofunctionalization of vinyl cyclopropanes. *Chem. Sci.* **11**, 3146-3151, (2020).

30 Liu, W. *et al.* A General regioselective synthesis of alcohols by cobalt-catalyzed hydrogenation of epoxides. *Angew. Chem. Int. Ed.* **59**, 11321-11324, (2020).

31 Li, G. X. *et al.* A unified photoredox-catalysis strategy for C(sp(3))-H hydroxylation and amidation using hypervalent iodine. *Chem. Sci.* **8**, 7180-7185, (2017).

32 Mashima, K. *et al.* Asymmetric Hydrogenation of Heteroaromatic Ketones and Cyclic and Acyclic Enones Mediated by Cu(I)-Chiral Diphosphine Catalysts. *Synlett* **2009**, 3143-3146, (2009).

33 Clive, D. L. & Kang, S. Synthesis of biaryls by intramolecular radical transfer in phosphinates. *J. Org. Chem.* **66**, 6083-6091, (2001).

34 Lafleur, K. *et al.* Optimization of inhibitors of the tyrosine kinase EphB4. 2. Cellular potency improvement and binding mode validation by X-ray crystallography. *J. Med. Chem.* **56**, 84-96, (2013).

35 Liu, X. G. *et al.* Decarboxylative negishi coupling of redox-active aliphatic esters by cobalt catalysis. *Angew. Chem. Int. Ed.* **57**, 13096-13100, (2018).

36 Tokumaru, T. & Nakata, K. InCl_3_-promoted intramolecular decarboxylative etherification of benzylic carbonates. *Tetrahedron Lett.* **56**, 2336-2339, (2015).

37 Zhang, C. *et al.* Base-catalyzed selective esterification of alcohols with unactivated esters. *Org. Biomol. Chem.* **16**, 8467-8471, (2018).

38 Li, C. *et al.* Transition-metal-free stereospecific cross-coupling with alkenylboronic acids as nucleophiles. *J. Am. Chem. Soc.* **138**, 10774-10777, (2016).

39 Falk, E., Gasser, V. C. M. & Morandi, B. Synthesis of N-alkyl anilines from arenes via iron-promoted aromatic C-H amination. *Org. Lett.* **23**, 1422-1426, (2021).

40 Blanco, V. *et al.* A switchable [2]rotaxane asymmetric organocatalyst that utilizes an acyclic chiral secondary amine. *J. Am. Chem. Soc.* **136**, 4905-4908, (2014).

41 Zhang, S., Ibrahim, J. J. & Yang, Y. A pincer ligand enabled ruthenium catalyzed highly selective N-monomethylation of nitroarenes with methanol as the C1 source. *Org. Chem. Front.* **6**, 2726-2731, (2019).

42 Seo, H. *et al.* Selective N-monomethylation of primary anilines with dimethyl carbonate in continuous flow. *Tetrahedron* **74**, 3124-3128, (2018).

43 Botla, V., Barreddi, C., Daggupati, R. V. & Malapaka, C. Base-oxidant promoted metal-free N-demethylation of arylamines. *J. Chem. Sci.* **128**, 1469-1473, (2016).

44 Tan, H. *et al.* N-Heterocyclic carbene catalyzed ester synthesis from organic halides through incorporation of oxygen atoms from air. *Angew. Chem. Int. Ed.* **60**, 2140-2144, (2021).

45 Aman, H. *et al.* Catalyst/additive free oxidation of benzyl bromides to benzaldehydes. *ChemistrySelect* **5**, 15015-15019, (2020).

46 Sang, D. *et al.* Anchimerically assisted selective cleavage of acid-labile aryl alkyl ethers by aluminum triiodide and N,N-dimethylformamide dimethyl acetal. *J. Org. Chem.* **85**, 6429-6440, (2020).

47 Ge, X. *et al.* Facile synthesis of hydrochar supported copper nanocatalyst for Ullmann C N coupling reaction in water. *Mol. Catal.* **484**, (2020).

48 Hoang, V. H. *et al.* Discovery of conformationally restricted human glutaminyl cyclase inhibitors as potent anti-alzheimer's agents by structure-based design. *J. Med. Chem.* **62**, 8011-8027, (2019).

49 Panchenko, S. P. *et al.* Arylation of adamantanamines: VIII. Optimization of the catalytic system for copper-catalyzed arylation of adamantane-containing amines. *Russ. J. Organ. Chem.* **53**, 1497-1504, (2017).

50 Wei, Y., Zhao, C., Xuan, Q. & Song, Q. An expedient and novel strategy for reductive amination by employing H_2_O as both a hydrogen source and solventviaB_2_(OH)_4_/H_2_O systems. *Org. Chem. Front.* **4**, 2291-2295, (2017).

51 Zhang, Q. *et al.* Calcium carbide as a dehydrating agent for the synthesis of carbamates, glycerol carbonate, and cyclic carbonates from carbon dioxide. *Green Chem.* **22**, 4231-4239, (2020).

52 Chou, C. H. *et al.* Synthesis and photophysical characterization of 2,3-dihydroquinolin-4-imines: new fluorophores with color-tailored emission. *Chem.-Eur. J.* **24**, 1112-1120, (2018).

53 Brown, T. J. *et al.* Use of (cyclopentadienone)iron tricarbonyl complexes for C-N bond formation reactions between amines and alcohols. *J. Org. Chem.* **82**, 10489-10503, (2017).

54 Arachchige, P. T. K., Lee, H. & Yi, C. S. Synthesis of symmetric and unsymmetric secondary amines from the ligand-promoted ruthenium-catalyzed deaminative coupling reaction of primary amines. *J. Org. Chem.* **83**, 4932-4947, (2018).

55 Gao, H. *et al.* Rapid heteroatom transfer to arylmetals utilizing multifunctional reagent scaffolds. *Nat. Chem.* **9**, 681-688, (2017).

56 Jiang, S. *et al.* A new ligand for copper-catalyzed amination of aryl halides to primary(hetero)aryl amines. *Tetrahedron Lett.* **61**, (2020).

57 Harris, M. C., Huang, X. & Buchwald, S. L. Improved functional group compatibility in the palladium-catalyzed synthesis of aryl amines. *Org. Lett.* **4**, 2885-2888, (2002).

58 See, Y. Y. & Sanford, M. S. C-H amination of arenes with hydroxylamine. *Org. Lett.* **22**, 2931-2934, (2020).

59 Qiu, Z. *et al.* Direct conversion of phenols into primary anilines with hydrazine catalyzed by palladium. *Chem. Sci.* **10**, 4775-4781, (2019).

60 Henry, M. C., Senn, H. M. & Sutherland, A. Synthesis of functionalized indolines and dihydrobenzofurans by iron and copper catalyzed aryl C-N and C-O bond formation. *J. Org. Chem.* **84**, 346-364, (2019).

61 Green, R. A. & Hartwig, J. F. Nickel-catalyzed amination of aryl chlorides with ammonia or ammonium salts. *Angew. Chem. Int. Ed.* **54**, 3768-3772, (2015).

62 De la Fuente-Olvera, A. A., Suárez-Castillo, O. R. & Mendoza-Espinosa, D. Synthesis and catalytic applications of palladium(II) complexes supported by hydroxyl-functionalized triazolylidenes. *Eur. J. Inorg. Chem.* **2019**, 4879-4886, (2019).

63 S, U. D. *et al.* A photochemical dehydrogenative strategy for aniline synthesis. *Nature* **584**, 75-81, (2020).

64 Mosley, C. A. *et al.* Synthesis, structural activity-relationships, and biological evaluation of novel amide-based allosteric binding site antagonists in NR1A/NR2B N-methyl-D-aspartate receptors. *Bioorg. Med. Chem.* **17**, 6463-6480, (2009).

65 Wei, Y., Xuan, Q., Zhou, Y. & Song, Q. Reductive N-alkylation of primary and secondary amines using carboxylic acids and borazane under mild conditions. *Org. Chem. Front.* **5**, 3510-3514, (2018).

66 Llopis, N. & Baeza, A. Oxidation of electron-rich arenes using HFIP-UHP system. *J. Org. Chem.* **85**, 6159-6164, (2020).

67 Phuc Tran, D. *et al.* Metal- and base-free synthesis of aryl bromides from arylhydrazines. *Tetrahedron Lett.* **61**, (2020).

68 Cheng, Q.-Q. *et al.* Organocatalytic nitrogen transfer to unactivated olefins via transient oxaziridines. *Nat. Catal.* **3**, 386-392, (2020).
